# Central-to-Axial-to-Central Chirality Transfer in the Au(I)-Catalyzed Cycloisomerization of Propargyl Vinyl Ethers to Cyclopentadienes

**DOI:** 10.1021/acs.joc.5c00433

**Published:** 2025-05-13

**Authors:** Dina Scarpi, Giovanni Turchi, Matteo Fazzini, Lucilla Favero, Ernesto G. Occhiato

**Affiliations:** † Dipartimento di Chimica “U. Schiff”, 9300 Università degli Studi di Firenze, Via della Lastruccia 13, Sesto Fiorentino, Florence 50019, Italy; ‡ Dipartimento di Farmacia, 9310 Università degli Studi di Pisa, Via Bonanno 33, Pisa 56126, Italy

## Abstract

An easy approach to the enantioselective synthesis of five-, six-, and seven-membered ring-fused cyclopentadienes (85–99% ee) is based on the Au­(I)-catalyzed cycloisomerization of enantiomerically pure or enriched propargyl vinyl ethers, which occurs with complete central-to-axial-to-central chirality transfer. DFT calculations show that the formation of a nonplanar σ-Au­(I)-pentadienyl cation intermediate having a helical configuration, which quickly cyclizes to form the target cyclopentadiene, accounts for the lack of erosion of the initial optical purity. From a synthetic point of view, when the cyclopentadienes are subjected to a quick 1,5-*H* shift and cannot be isolated as pure regioisomers, they can be trapped *in situ* by suitable dienophiles during or immediately after the gold­(I)-catalyzed cycloisomerization to form more complex polycyclic compounds. The synthesis of an enantiomerically pure α-tertiary amine was realized to demonstrate the usefulness of this approach.

## Graphical abstract

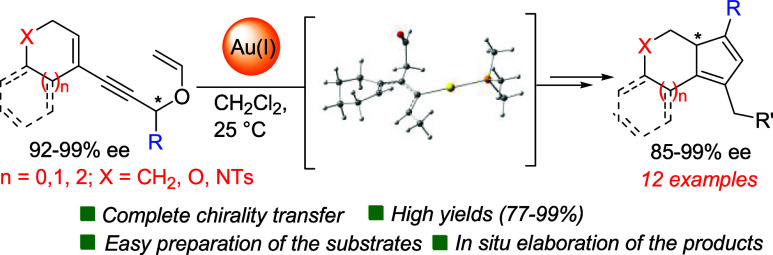

## Introduction

The chirality transfer from nonracemic substrates to enantioenriched products is a powerful synthetic tool for the synthesis of more complex, C­(sp^3^)-rich compounds, including carbo- and heterocyclic natural products.^1,2^ Gold catalysis has quickly gained momentum in chirality transfer thanks to the ability of Au­(I) to activate both alkynes and alkenes in inter- and intramolecular processes.^3^ Gagosz first^4^ and then Malacria^5,6a^ reported efficient center-to-axis-to-center chirality transfer in the Au­(I)-catalyzed cycloisomerization of suitably substituted propargyl acetates **1** and **3** (Scheme 1a,b), occurring via the initial 1,3-shift of the acetyloxy group to form chiral allenes, which provided complex polycyclic compounds **2** and **4** with almost intact optical purity.

**Scheme 1:**
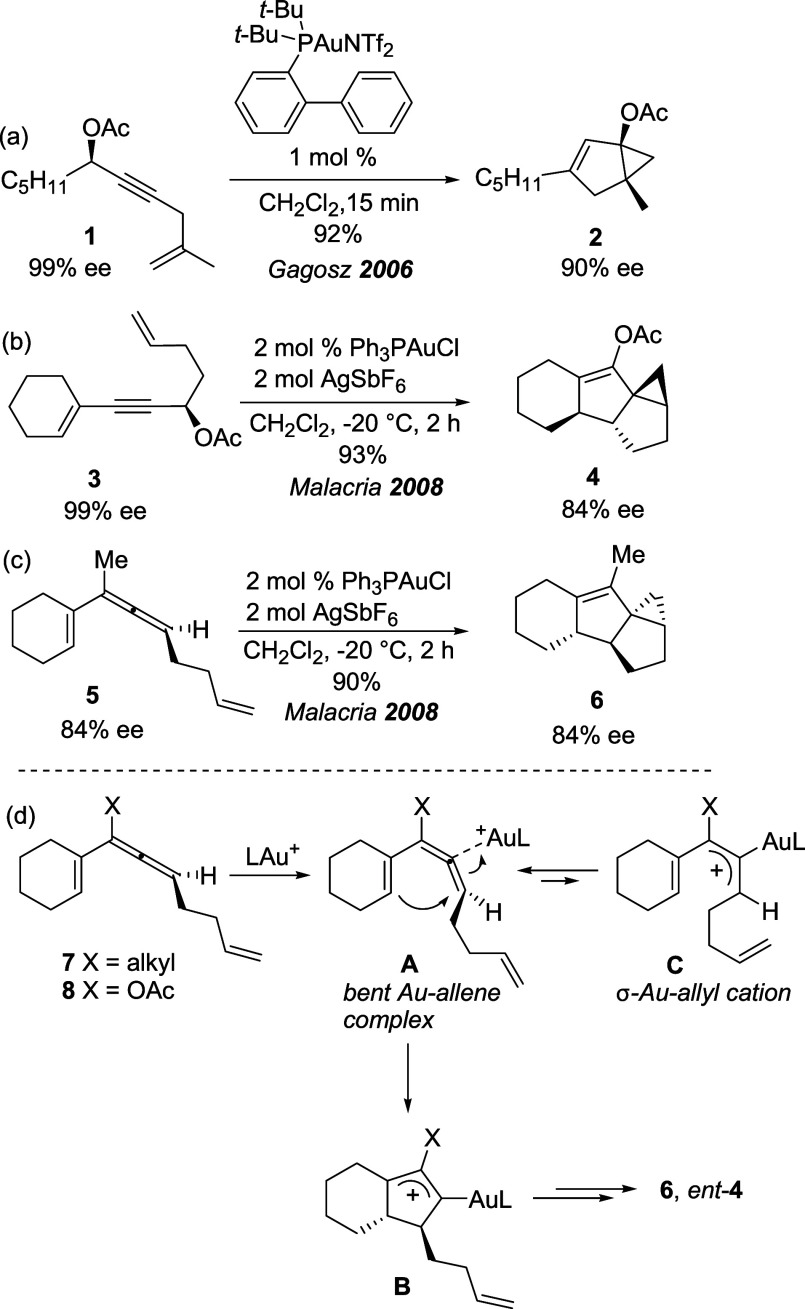
Previous Studies on Chirality Transfer in Au­(I)-Catalyzed Cycloisomerization

With enyne **3**, the initial 1,3-acyloxy shift is followed by a Nazarov-type cyclization^7^ involving the allene intermediate, which forms a cationic cyclopentenylidene gold species in turn undergoing the eventual attack by the pendant olefin.^5,6a^ The chirality transfer observed in tandem processes queuing the 1,3-acyloxy migration and a Nazarov reaction was later exploited by Carreira^8^ and Zhang^9^ in the synthesis of complex, sp^3^-rich pentannulated compounds, and cyclopentadienyl esters, respectively.^10,11^

The axis-to-center chirality transfer from isolated chiral allenes to the final products was demonstrated by Malacria with enantioenriched allene **5** (Scheme 1c), which, under Au­(I) catalysis, provided polycyclic compound **6** without erosion of the initial optical purity.^6a^ DFT calculations suggested that chiral allenes such as **7** and **8** form η1-coordinated “bent” allene-Au­(I) complexes **A** (Scheme 1d), where gold is covalently linked to the central carbon of the allenic moiety, which are able to transmit chirality to **B** in the next Nazarov-like cyclization because of their helical configuration.^6a^ Crucial to that is the ability of the “bent” allene complexes to retain the chirality of the starting material as racemization of these allenes could quickly take place through the equilibrium with planar σ-Au­(I)-allyl cation complexes **C**.^6^

We have recently reported on the tandem Au­(I)-catalyzed cycloisomerization of propargyl vinyl ethers **9** to aldehydes **10** occurring via a stepwise Claisen rearrangement to form Au­(I)-allene complexes **E**, which then cyclize to eventually produce the final cyclopentadienes **10** in excellent yields (Scheme 2).^12,13^

**Scheme 2:**
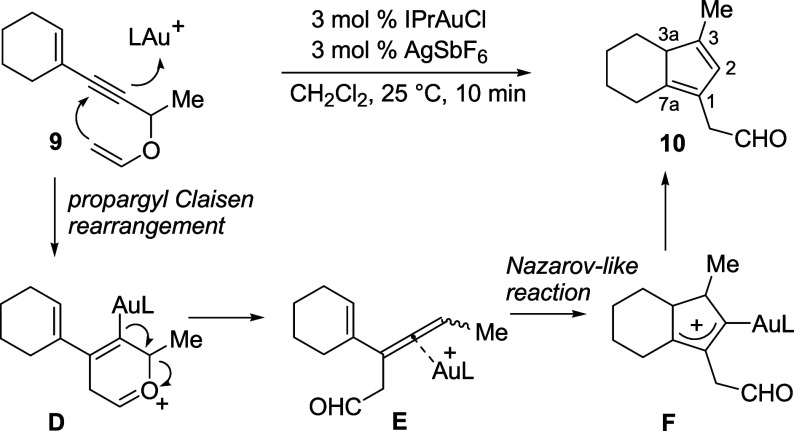
Cycloisomerization of Propargyl Vinyl Ethers Leading to Cyclopentadienes

Since center-to-axis chirality transfer to allenes has been demonstrated by Toste in the Claisen rearrangement of simple propargyl vinyl ethers,^14^ we were eager to evaluate whether the center-to-axis-to-center chirality transfer observed with propargyl acetates^8,9^ could occur with the same efficiency in the Au­(I)-catalyzed cycloisomerization of nonracemic propargyl vinyl ethers **9** as well, in which the final step creating the stereocenters in **F** is a Nazarov-like reaction (as in the cycloisomerization of propargyl esters). To have complete chirality transfer, allene intermediate **E** must be sufficiently reactive to avoid its partial or complete racemization through σ-bond rotation in a planar σ-Au­(I)-allyl cation complex (analogous to **C**) under the conditions of the tandem process. Encouragingly, we had previously found that cyclization of trisubstituted Au­(I)-allene complexes **E** is very fast,^12^ as we were never able to observe or isolate the allene intermediates in the cycloisomerization of propargyl vinyl ethers, if not when the latter bore an aromatic ring instead of the cyclohexenyl ring.^15^ If successful, this approach would allow for facile access to enantiopure, functionalized cyclopentadienes, which are important reactants in organic synthesis, especially as partners in Diels–Alder reactions^16,17^ and scaffolds for transition metal cyclopentadienyl (Cp) complexes,^18^ but are difficult to synthesize owing to their instability induced by Diels–Alder dimerization and 1,5-sigmatropic hydrogen migration.^19^

## Results and Discussion

Enantioenriched substrates **14** used for this study were prepared by the initial Sonogashira coupling of vinyl triflates **11** with either enantiopure or racemic alcohols **12** (Scheme 3) as reported.^12c^ In the latter case, enzymatic kinetic resolution of the Sonogashira coupling products was exploited to obtain enantioenriched enynyl alcohols **13** for the next vinylation^12b^ (see Supporting Information), which eventually provided the requisite propargyl vinyl ethers **14** in 48–75% yield.

**Scheme 3:**
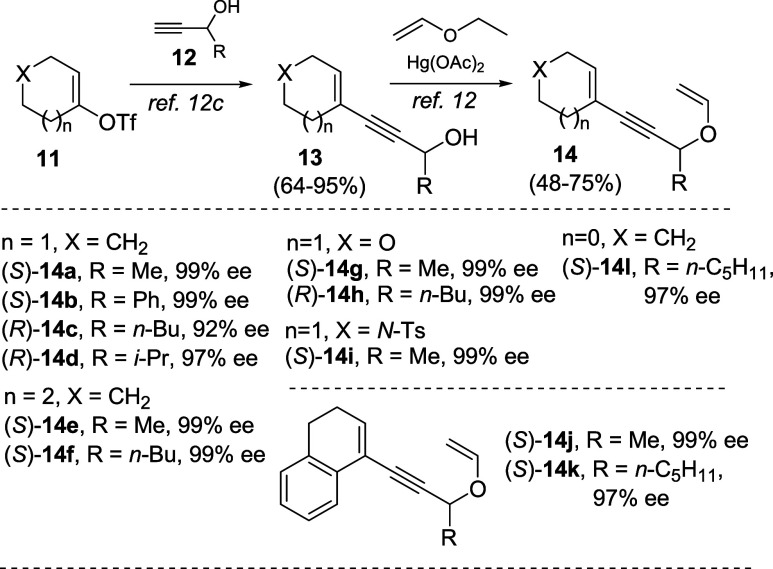
Synthesis of Enantiopure and Enantioenriched Propargyl Vinyl Ethers **14a–l**

A screening of gold­(I) catalysts was carried out for the cycloisomerization of compound (*S*)-**14a** (99% ee) in dichloromethane as the solvent (Table 1). To avoid migration of the double bond to the exocyclic position, the aldehyde group was reduced *in situ* by NaBH_4_ after the disappearance of the starting material (by TLC monitoring).

**Table 1:**
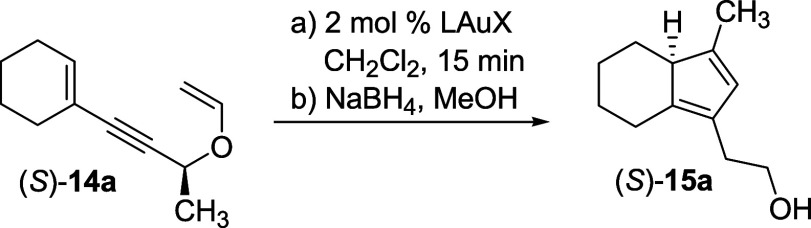
Screening of Au­(I) Catalysts in the Cycloisomerization of **14a**

Entry	Catalyst^a^	*T* (°C)	Yield (%)^b^	ee (%)^c^
1	*t*-Bu_3_PAuNTf_2_	25	83	96
2	*t*-Bu_3_PAuNTf_2_	0	85	96
3	*t*-Bu_3_PAuNTf_2_	–20	81	96
4	*t*-Bu_3_PAuCl/AgNTf_2_	25	59	96
5	*t*-Bu_3_PAuCl/AgBF_4_	25	40	86
6	*t*-Bu_3_PAuCl/AgSbF_6_	25	76	96
7	*t*-Bu_3_PAuCl/AgOTf	25	32	95
8	*t*-Bu_3_PAuCl/NaBARF	25	69	95
9^d^	IPrAuNTf_2_	25	94	95^e^
10	IPrAuBF_4_	25	86	96
11	Ph_3_PAuCl/AgNTf_2_	25	36	68
12	Ph_3_PAuCl/AgSbF_6_	25	46	70
13	[2,4-di-*t*-BuC_6_H_3_O]_3_PAuCl/AgSbF_6_	25	45	92
14	[(C_6_F_5_)_3_P]AuCl/AgSbF_6_	25	74	92
15	[(Ph_3_PAu)_3_O]BF_4_	25	0	-
16	AgSbF_6_	25	32	96

a Commercially preformed catalysts or prepared by mixing the silver salt (2 mol %) and the gold chloride (2 mol %) in CH_2_Cl_2_ before the addition of the substrate. IPr = 1,3-bis­(diisopropylphenyl)­imidazol-2-ylidene. NaBARF = Sodium tetrakis­[3,5-bis­(trifluoromethyl)­phenyl]­borate.

b After chromatography on silica gel.

c Determined by chiral GLC on a β-DEX 120 column after hydrogenation of **15a** on a 10% Pd/C catalyst in MeOH (see Supporting Information).

d Reaction completed in 30 min.

e A slightly higher ee (98%) for (*S*)-**15a** was found by chiral HPLC on a Lux 5μ Amylose-1 column after reaction with *N*-phenylmaleimide (see Figure 1 and Supporting Information).

We were glad to see that the reaction was very fast in most cases, being complete in less than 15 min, and occurred with almost complete center-to-axis-to-center transfer of chirality, as the erosion of the optical purity of the starting material was minimal.

Preformed complexes with electron-rich ligands (*t*-Bu_3_PAuNTf_2_, IPrAuNTf_2_, and IPrAuBF_4_) provided the final product (entries 1–3, 9–10) with high isolated yields (>85%) and ee’s (95–96%), whereas with Au­(I) complexes obtained by mixing gold­(I) and silver­(I) salts, the yields were, in general, moderate to low. Among the silver salts used to provide the noncoordinating anion, AgSbF_6_ was comparatively superior in terms of both yields and enantiomeric excesses (e.g., compare entry 6 with entries 5 and 7–8). Finally, the ee of the product did not change with the temperature (entries 1–3). In these three experiments, the cycloisomerization was equally fast (less than 15 min) and provided (*S*)-**15a** in 96% ee. Interestingly, AgSbF_6_ alone was able to catalyze the reaction with almost complete chirality transfer; however, the yield after chromatography was very low, demonstrating the need of Au­(I) for an effective catalysis.

The (*S*)-absolute configuration of **15a** was determined by reacting the isolated diene with commercial enantiopure (*R*)-(+)-*N*-(1-phenylethyl)­maleimide (Scheme 4), which provided, with complete facial selectivity, cycloadduct **16** as a 3:1 mixture of *endo* and *exo* stereoisomers.^20^ Crystals of the major *endo* isomer, suitable for X-ray analysis, were obtained by the slow evaporation of a diethyl ether solution.

**Scheme 4:**
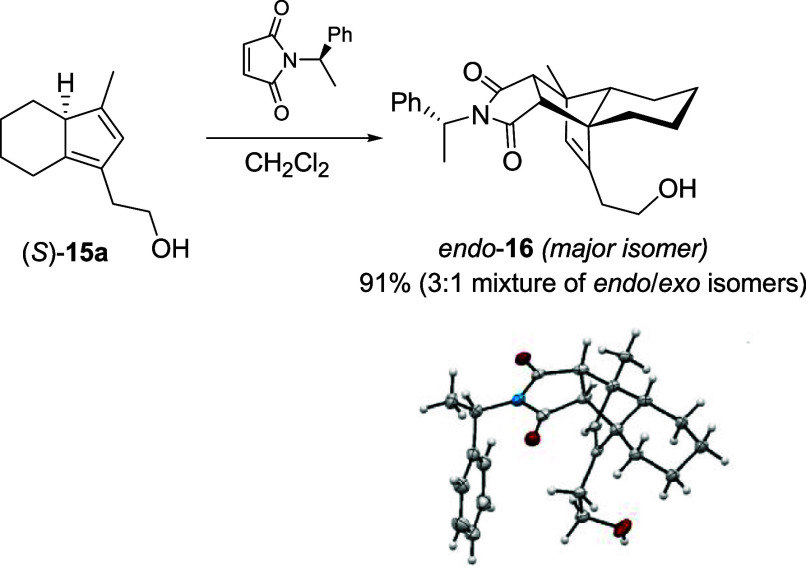
Determination of the Absolute Configuration of (*S*)-**15a**

Of the preformed catalysts initially screened, we used commercial IPrAuBF_4_ to evaluate the scope of the reaction with various R substituents and ring types on **14** (Table 2).

**Table 2:**
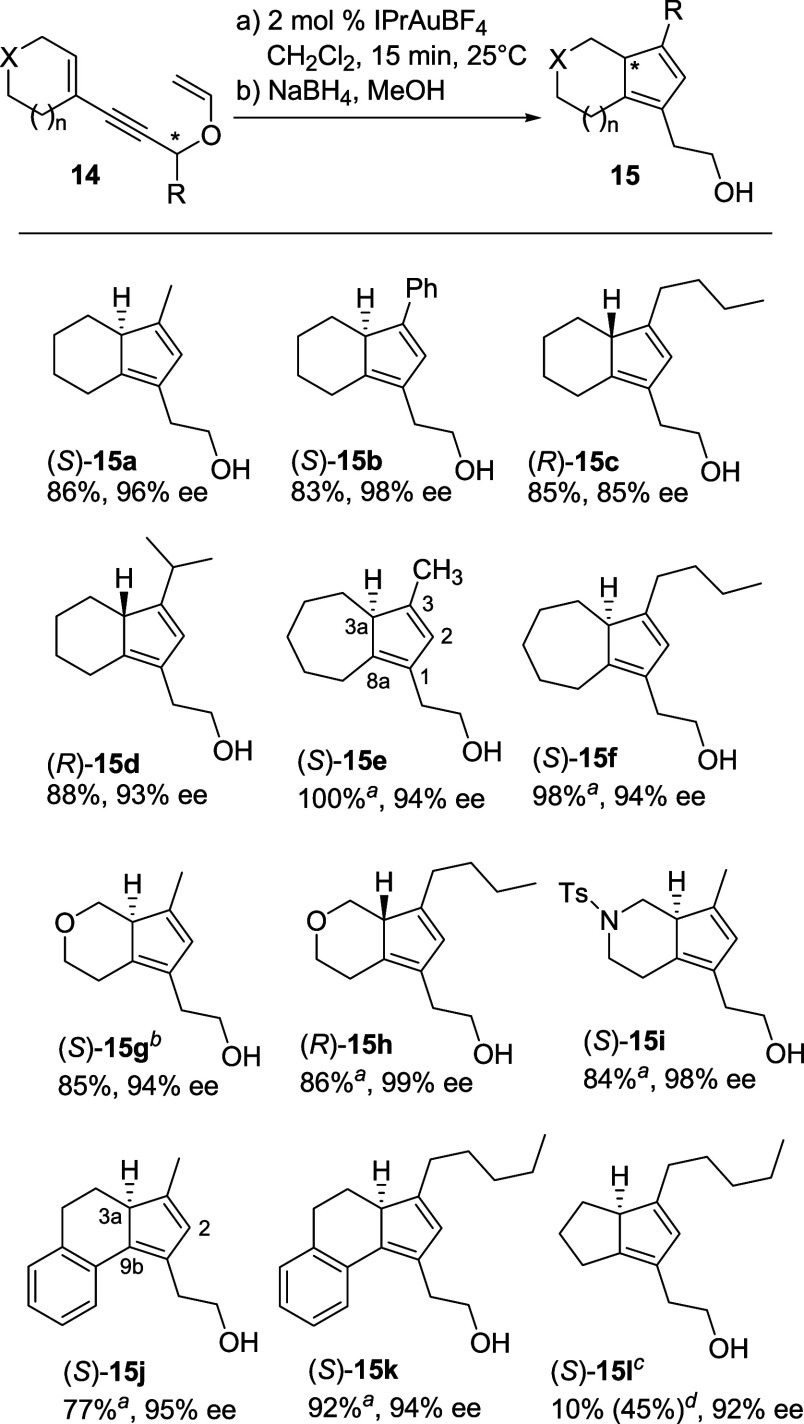
Synthesis of Enantiopure and Enantioenriched Ring-Fused Cyclopentadienes **15a–l**

a Yield calculated on the crude reaction mixture.

b In a 10:1 mixture with a minor regioisomer.

c 2:1 mixture of regioisomers.

d Calculated based on the yield of its cycloaddition product.

The ee of the products was determined by chiral HPLC after trapping the dienes with *N*-phenylmaleimide, either before or after their isolation, to form cycloaddition products **18** (see Figure 1 and Supporting Information). As we have already shown with racemic substrates,^12c^ this reaction is highly facial- and endo-selective and allows one to obtain stable compounds for HPLC analysis. For the ee determination, all reactions were carried out on racemic starting material as well.

**Figure 1:**
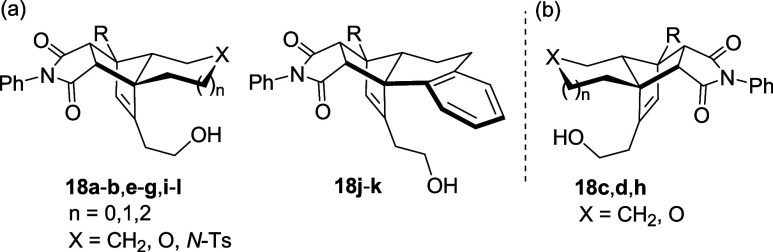
Cycloaddition products **18a**–**l** prepared for ee determination by HPLC analysis. (a) Products derived from substrates (*S*)-**14**; (b) products derived from substrates (*R*)-**14**.

With a single exception, cycloisomerization provided the dienes in excellent yields (77–99%) and took place with no or just a small erosion of the initial enantiomeric excess. While six-membered ring-fused cyclopentadienes (*S*)-**15a**-**b** and (*R*)-**15c**-**d** were regioisomerically stable and could be isolated in high yield by chromatography, in the case of products (*S*)-**15e**-**f**, having a fused seven-membered ring, we noted a high propensity to double-bond isomerization inside the five-membered ring both when chromatographed on silica gel and when analyzed by NMR in CDCl_3_. Notwithstanding, quite clean ^1^H NMR spectra of the crude reaction mixtures were obtained in CD_3_OD, and therefore, these compounds were characterized as such (**15e** and **15f** as 25:1 and 10:1 mixtures of regioisomers, respectively)^21^ before subjecting them to cycloaddition with *N*-phenylmaleimide for the ee determination.

Double-bond isomerization to the ring-fused position in products **15** was observed when a heteroatom was present in the six-membered ring. The reaction of **14g**-**h,** with an O atom in the ring, indeed provided, after work-up, the corresponding products **15g**-**h** as inseparable 10:1 and 4:1 mixtures, respectively, with minor regioisomers **17g**-**h** (Scheme 5).^22^ Similarly, with an N atom in the six-membered ring, a 3:1 mixture of regioisomers (*S*)-**15i** and **17i** ^22^ was obtained when we tried to isolate the cyclopentadiene after the cycloisomerization and reduction by NaBH_4_ (Scheme 6). TLC control of this reaction after reduction, but before work-up, revealed that a single spot was present, while spots were two after work-up. Thus, isomerization must have occurred during the latter process and not during any of the preceding steps. We discovered, in fact, that by changing the work-up, i.e., adding water before rotary evaporation of the methanol and extraction of the product with dichloromethane, the isomerization of both dienes (*R*)-**15h** and (*S*)-**15i** did not occur at all and the crude products were obtained as single isomers and in sufficiently pure form to be fully characterized.

**Scheme 5:**
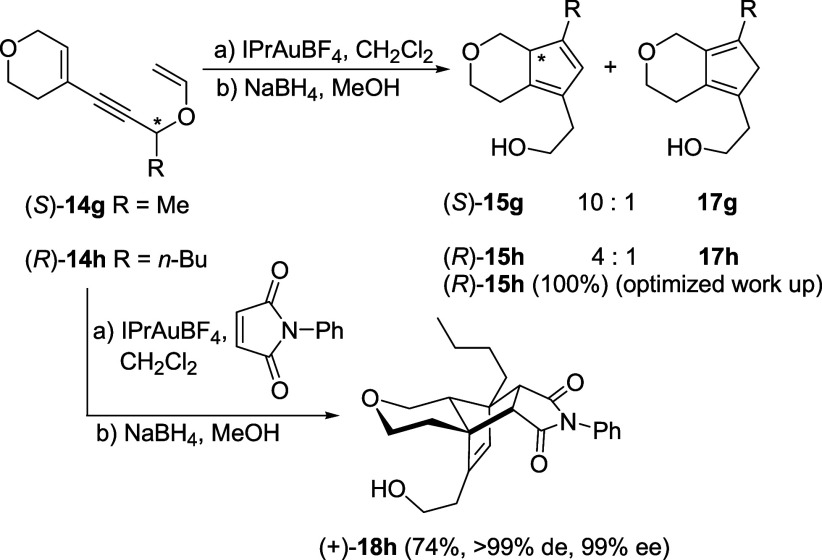
Cycloisomerization of Propargyl Vinyl Ether (*S*)-**14g** and (*R*)-**14h**

**Scheme 6:**
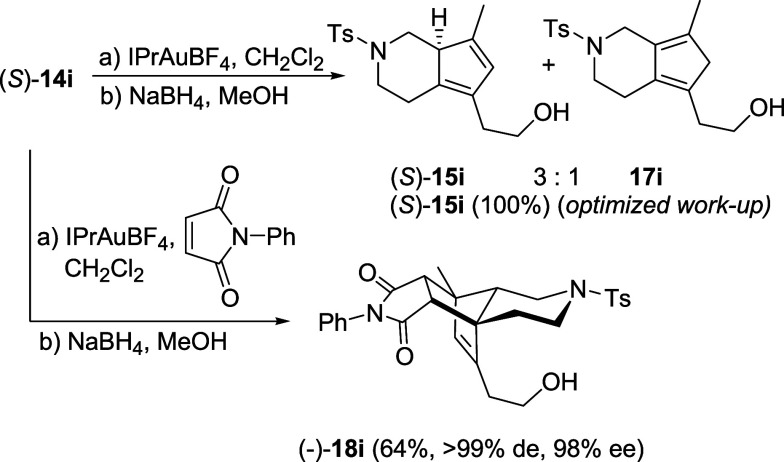
Cycloisomerization of Propargyl Vinyl Ether (*S*)-**14i**

To further prove this, with substrates (*R*)-**14h** and (*S*)-**14i** (Schemes 5 and 6), we repeated the reaction in the presence of *N*-phenylmaleimide to trap the cyclopentadienyl intermediates once formed, before the reduction of the carbonyl group and work-up, and, gratifyingly, we obtained in both cases cycloadducts **18h** and **18i** as single, almost enantiopure isomers in good yields (74% and 64%, respectively). This confirms that the cycloisomerization is regioselective in terms of double-bond position in the five-membered ring and that, also with heterocyclic substrates **14g**–**i**, the reaction occurs with complete chirality transfer.

Optimized work-up provided regioisomerically pure crude products also in the cases of **15j**-**k** (77–92% yield), which were obtained in the cycloisomerization of α-tetralone-derived propargyl vinyl ethers **14j**-**k**. The gold­(I)-catalyzed cycloisomerization of these substrates was repeated in the presence of *N*-phenylmaleimide to obtain cycloadducts **18j** and **18k** (in 88% and 84% yield, respectively), which were necessary for the ee determination by chiral HPLC. In both cases, the facial selectivity was lower than usual (dr 5:1 and 4:1, respectively, by ^1^H NMR before chromatographic purification of the major diastereomer, see Supporting Information),^23^ likely because of the diminished difference in the steric hindrance of the two diene faces in **15j**-**k**. The 1,2-dihydronaphthalene ring in substrates **15j**-**k** had instead no influence on the chirality transfer, which was almost complete also in these cases (95% and 94% ee, respectively).

More difficult was the isolation, even after optimized work-up, of regioisomerically pure or enriched tetrahydropentalene **15l**. The 1,5-*H* sigmatropic rearrangement within such a structure to form more stable isomers is known to occur very easily,^24^ and, in fact, the cycloisomerization (which occurred in 30 min) of **14l** provided a complex reaction mixture from which, after chromatography, it was possible to isolate an enriched fraction of two regioisomers of **15l** (in a 2:1 ratio) in 10% yield. In any case, by carrying out the cycloisomerization in the presence of *N*-phenylmaleimide an almost isomerically pure cycloadduct **18l** was obtained, albeit in quite low yield (45%), which allowed us to determine an ee for **15l** of 92% and thus to assess that, also in such a case, the initial chirality is conserved in the final product.

DFT calculations^25^ (Scheme 7), carried out using Me_3_P as the gold­(I) ligand, showed that the propargyl Claisen rearrangement of gold-complex **I** with (*S*) absolute configuration to give gold­(I)-allene complex *R*_*a*_-**IIIa** was the rate-limiting step of the whole process (Δ*G* ^‡^ = 58 kJ/mol). In this complex, cationic gold preferentially coordinates with the proximal double bond of the allene moiety (see Supporting Information). The subsequent electrocyclization to give bicyclic cation **V** is much faster, which explains why we never detected intermediate allenes in these reactions. Interestingly, the energy barrier for racemization of allene complex **IIIa** (to *ent*-**IIIa**) through a planar transition state (**TS-rac**) is much higher (76.5 kJ/mol) than that of its electrocyclization (41.8 kJ/mol) to cation **V** that occurs through a nonplanar pentadienyl cation **IV** having a helical P absolute configuration, with a C(1)–C(2)–C(4)–C(5) angle of 47.10°. The geometrical parameters of this cation match those reported for “bent” allenes by Gandon et al.,^6a,26^ although with our calculations, the C(1)–C(2)–C(3) angle (120.73°) is smaller, being therefore closer to that of an sp^2^-hybridized central carbon atom [C(2)].

**Scheme 7:**
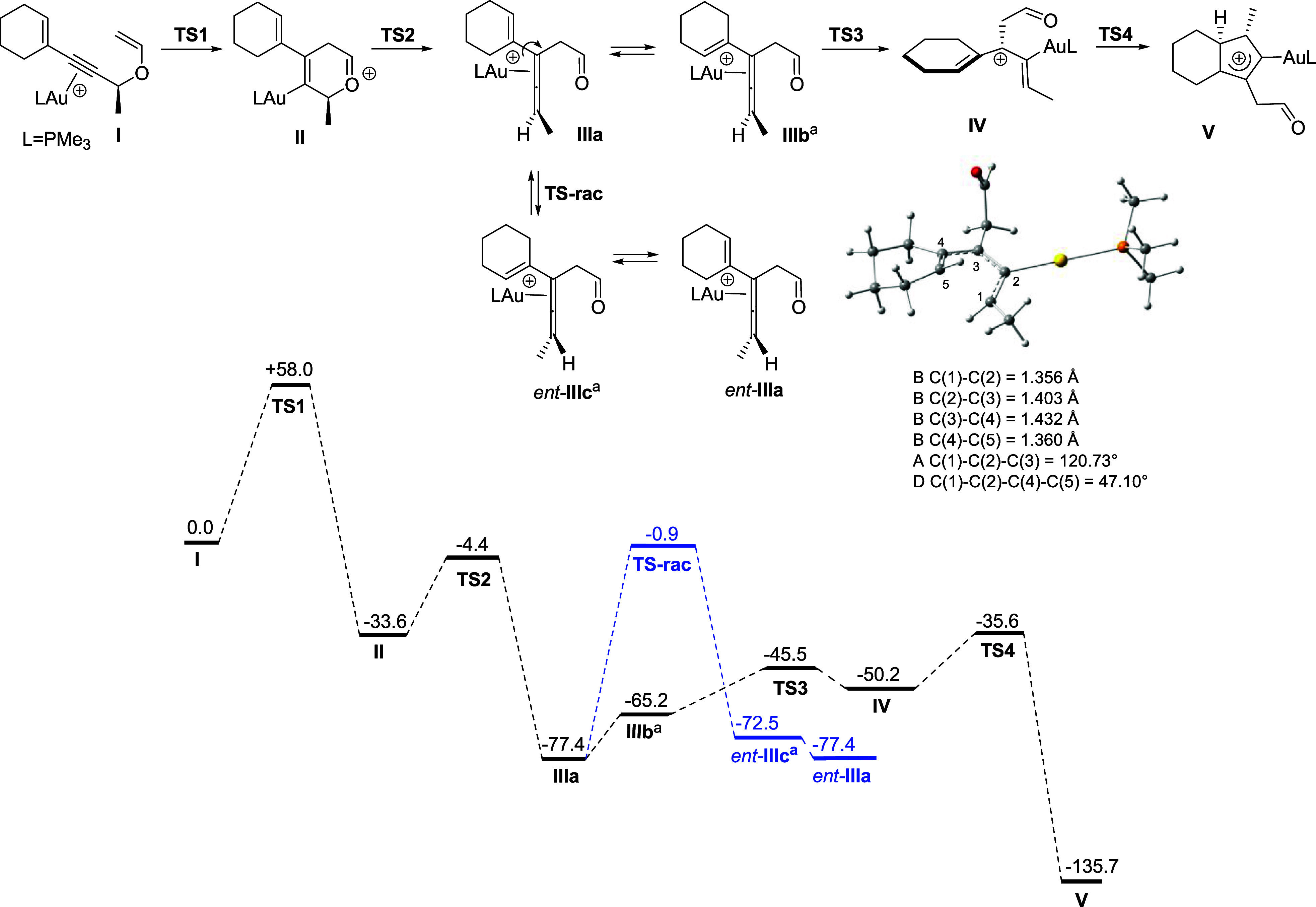
Computed Reaction Profile for the Cycloisomerization Process^a^

a Conformer obtained by IRC calculation (see Supporting Information). Gibbs energies are in kJ/mol.

Given its P configuration, the formation of the new C(5)–C(1) bond in the ring closure to **V** takes place, thereby generating a new stereogenic center at the ring junction with *S* absolute configuration, in accordance with our experimental data. This process occurs without erosion of the initial optical purity as the electrocyclization step of cation **IV**, which retains the stereochemical information of the substrate, is much faster than the racemization of allene **IIIa** as it results from our DFT calculations.^27^

To demonstrate the usefulness of the Au­(I)-catalyzed cycloisomerization of propargyl vinyl ethers in the synthesis of more complex enantiopure compounds, we prepared *N*-protected α-tertiary amine (*S*)-(+)-**22** (Scheme 8). α-Tertiary amines, i.e., amines bearing an α-tetrasubstituted stereocenter, are common in biologically active natural products and small molecules and still represent a synthetic challenge.^28^ According to a strategy we have reported for its racemic counterpart,^12b^ (*S*)-(+)-**22** was obtained in good overall yield (40% over five steps) and ee (97%)^29^ by trapping with DEAD (diethyl azodicarboxylate) the cyclopentadiene intermediate generated from **14a**, followed by reduction, acid-catalyzed ring opening, and N–N cleavage.

**Scheme 8:**
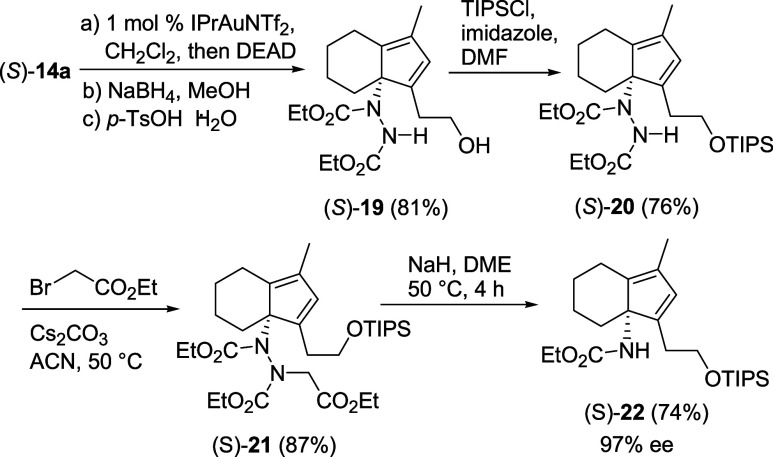
Synthesis of Enantiopure *N*-Protected α-Tertiary Amine (*S*)-**22**

## Conclusions

In conclusion, the Au­(I)-catalyzed cycloisomerization of enantiomerically pure propargyl vinyl ethers occurs with complete central-to-axial-to-central chirality transfer through the formation of a nonplanar pentadienyl cation (based on DFT calculations) having a helical configuration, which quickly cyclizes to form the target cyclopentadienes. Racemization of the allene intermediate is avoided because of the very fast 4π-electrocyclization of this nonplanar carbocation. Some of the enantiomerically pure five-, six-, and seven-membered ring-fused cyclopentadienes we prepared are prone to a 1,5-*H* shift to generate regioisomers, but in the context of a synthetic application, this can be circumvented by trapping them *in situ* during or immediately after the gold­(I)-catalyzed cycloisomerization to form more complex target products. The synthesis of an enantiomerically pure α-tertiary amine by a hetero-Diels–Alder/ring-opening process involving one of the cyclopentadienes was realized to demonstrate the usefulness of this approach.

## Experimental Section

### General Information

Anhydrous solvents were prepared according to standard techniques. Commercially available reagents were used without further purification. Melting points were recorded on a Büchi B-540 apparatus and are uncorrected. Optical rotations were determined with a JASCO DIP-370 instrument. Chromatographic separations were performed under pressure on silica gel (Merck 70-230 mesh) by using flash column techniques; *R*_f_ values refer to TLC carried out on 0.25 mm silica gel plates (F_254_) with the same eluent as indicated for column chromatography. ^1^H NMR (200 or 400 MHz) and ^13^C NMR (100.4 MHz) spectra were recorded either on Varian Inova (400 MHz) or Mercury (200 or 400 MHz) spectrometers in the specified deuterated solvent at 25 °C. Solvent reference lines were set at 7.26 (CDCl_3_) and 3.31 (CD_3_OD) in ^1^H NMR spectra and at 77.00 (CDCl_3_), 49.00 (CD_3_OD), and 206.26 (acetone-*d*_6_) in the ^13^C NMR spectra, respectively. Mass spectra were recorded either by direct inlet of a 20 ppm solution in CH_3_OH on an LCQ Fleet Ion Trap LC/MS system (Thermo Fisher Scientific) with an electrospray ionization (ESI) interface in the positive ion mode or by electron ionization (EI) at 70 eV on a Shimadzu GC/MS-QP2020NX instrument equipped with a SH-Rxi-5 ms Shimadzu column. Microanalyses were carried out with a Thermo Scientific FlashSmart Elemental Analyzer CHNS/O. HRMS analyses were performed under conditions of ESI-MS through direct infusion of a 10 ppm solution in 90/10 MeOH/H_2_O containing 0.1% formic acid in an LTQ Orbitrap mass spectrometer (Thermo Scientific). HPLC analyses were carried out with a Dionex UltiMate 3000 HPLC system equipped either with a Lux 5 μm Amylose-1 or with a Lux 5 μm Cellulose-4 column, 250 × 4.60 mm, and eluting at a 0.5 mL/min flow rate with the reported eluent in isocratic conditions. GLC analyses were carried out on a Shimadzu GC2014 instrument equipped with a Supelco β DEX 120, 30 m × 0.25 mm, 0.25 μm film column. 1-Cyclohexenyl trifluoromethanesulfonate (**11a**) is commercially available; 1-cycloheptenyl trifluoromethanesulfonate (**11b**), 4-trifluoromethanesulfonate-3,6-dihydro-2*H*-pyran (**11c**), 4-trifluoromethanesulfonate-1-tosyl-1,2,3,6-tetrahydropyridine (**11d**), 4-trifluoromethanesulfonate-1,2-dihydronaphthalene (**11e**), and 1-cyclopentenyl trifluoromethanesulfonate (**11f**) were prepared as reported;^12c^ compounds **11b**,^30^ **11c**,^31^ **11d**,^30^ **11e**,^30^ and **11f** ^30^ are known.

#### General Procedure for the Synthesis of Propargyl Vinyl Ethers **14a–l**

A 3:1 (v/v) solution of anhydrous THF/Et_3_N (6.6 mL) was added to a round-bottomed flask containing the suitable vinyl trifluoromethanesulfonate (**11**, 1 mmol). The alkynol (**12**) (1.0 equiv), CuI (3.2 mol %), and (Ph_3_P)_2_PdCl_2_ (1.6 mol %) were then added under a nitrogen atmosphere, and the reaction mixture was stirred at room temperature for 3 h. Water (15 mL) was then added, and the product was extracted with Et_2_O (3 × 7 mL). The combined organic extracts were washed with brine (15 mL) and dried in anhydrous K_2_CO_3_. After filtration and evaporation of the solvent, the crude reaction mixture was purified by flash chromatography, affording intermediate enynyl alcohol **13**, which was used immediately in the next step. In a screw-cap vial, Hg­(OAc)_2_ (45 mol %) was added in one portion to a solution of enynyl alcohol **13** (1 mmol) in ethyl vinyl ether (6.6 mL, 0.15 M) under a nitrogen atmosphere, and the reaction mixture was heated at 50 °C (external, oil bath) for 18–20 h. The mixture was then cooled to room temperature, and a saturated aqueous solution of Na_2_CO_3_ (7 mL) was added. The product was extracted with Et_2_O (3 × 7 mL), and the combined organic extracts were dried over anhydrous K_2_CO_3_. After filtration and evaporation of the solvent, the crude reaction mixture was purified by flash column chromatography to give pure **14**, which was stored at 4 °C as a solution in the eluent containing 1% Et_3_N. The solution of **14** in the eluent was concentrated and dried under a *vacuum* just prior to use.

##### (*S*)-(−)-1-(3-Vinyloxybut-1-ynyl)­cyclohexene (**14a**)

Sonogashira coupling of **11a** (525 μL, 3.0 mmol) and (*S*)-3-butyn-2-ol (235 μL, 3.0 mmol; ee, 99%), followed by purification by flash chromatography (*n*-hexane/EtOAc, 3:1 + 1% Et_3_N; *R*_f_ = 0.32), afforded the enynyl alcohol intermediate (*S*)-**13a** (374 mg, 83%), which was used immediately in the next step. ^1^H NMR (200 MHz, CDCl_3_) δ (ppm): 6.12–6.08 (m, 1H), 4.69–4.59 (m, 1H), 2.13–2.05 (m, 4H), 1.82–1.72 (m, 1H), 1.67–1.52 (m, 6H), 1.46 (d, *J* = 6.6 Hz, 3H). Vinylation of (*S*)-**13a** (374 mg, 2.5 mmol) afforded (*S*)-**14a**, which was purified by flash chromatography (*n*-hexane +1% Et_3_N; *R*_f_ = 0.37). Pure (*S*)-**14a** was obtained as a pale yellow oil (250 mg, 57%). ee, 99%. [α]_D_^24^ −136.0 (*c* 1.0, CHCl_3_). ^1^H NMR (400 MHz, CDCl_3_) δ (ppm): 6.44 (dd, *J* = 14.0, 6.4 Hz, 1H), 6.13–6.10 (m, 1H), 4.65 (q, *J* = 6.8 Hz, 1H), 4.40 (dd, *J* = 14.0, 1.6 Hz, 1H), 4.11 (dd, *J* = 6.4, 1.6 Hz, 1H), 2.14–2.04 (m, 4H), 1.65–1.53 (m, 4H), 1.51 (d, *J* = 6.8 Hz, 3H). Spectroscopical data were identical to those reported for the corresponding racemic compound.^12a^

##### (*S*)-(−)-(3-Cyclohex-1-enyl-1-vinyloxyprop-2-ynyl)­benzene (**14b**)

Sonogashira coupling of **11a** (242 μL, 1.4 mmol) and (*R*)-1-phenyl-2-propyn-1-ol (182 mg, 1.4 mmol; ee, 99%), followed by purification via flash chromatography (*n*-hexane/EtOAc, 3:1 + 1% Et_3_N; *R*_f_ = 0.39), afforded the enynyl alcohol intermediate (*S*)-**13b** (223 mg, 75%), which was used immediately in the next step. ^1^H NMR (400 MHz, CDCl_3_) δ (ppm): 7.58–7.54 (m, 2H), 7.41–7.30 (m, 3H), 6.18–6.16 (m, 1H), 5.58 (d, *J* = 6.0 Hz, 1H), 2.18–2.06 (m, 5H), 1.67–1.56 (m, 5H). Vinylation of (*S*)-**13b** (223 mg, 1.05 mmol) afforded (*S*)-**14b**, which was purified by flash chromatography (*n*-hexane +1% Et_3_N; *R*_f_ = 0.08). Pure (*S*)-**14b** was obtained as a pale yellow oil (120 mg, 48%). ee, 99%. [α]_D_^24^ −50.1 (*c* 1.0, CHCl_3_). ^1^H NMR (400 MHz, CDCl_3_) δ (ppm): 7.54–7.51 (m, 2H), 7.41–7.32 (m, 3H), 6.52 (dd, *J* = 14.0, 6.4 Hz, 1H), 6.20–6.17 (m, 1H), 5.63 (s, 1H), 4.51 (dd, *J* = 14.0, 2.0 Hz, 1H), 4.17 (dd, *J* = 6.4, 2.0 Hz, 1H), 2.17–2.08 (m, 4H), 1.67–1.55 (m, 4H). Spectroscopical data were identical to those reported for the corresponding racemic compound.^12b^

##### (*R*)-(+)-1-(3-Vinyloxyhept-1-ynyl)­cyclohexene (**14c**)

Sonogashira coupling of **11a** (176 μL, 1.0 mmol) and (*±*)-1-heptyn-2-ol (132 μL, 1.0 mmol), followed by purification via flash chromatography (*n*-hexane/EtOAc, 4:1 + 1% Et_3_N; *R*_f_ = 0.53), afforded the enynyl alcohol intermediate (162 mg, 84%), which was used as the substrate for the enzymatic kinetic resolution with CAL-B (see Supporting Information). ^1^H NMR (400 MHz, CDCl_3_) δ (ppm): 6.14–6.02 (m, 1H), 4.46 (t, *J* = 6.6 Hz, 1H), 2.16–2.02 (m, 4H), 1.90 (br s, OH), 1.80–1.66 (m, 2H), 1.66–1.50 (m, 4H), 1.49–1.29 (m, 4H), 0.91 (t, *J* = 7.2 Hz, 3H). Vinylation of (*R*)-**13c** (67 mg, 0.29 mmol; ee, 92%) afforded (*R*)-**14c**, which was purified by flash chromatography (*n*-hexane +1% Et_3_N; *R*_f_ = 0.25). Pure (*R*)-**14c** was obtained as a pale yellow oil (40 mg, 68%). ee, 92%. [α]_D_^24^ +83.9 (*c* 1.0, CHCl_3_). ^1^H NMR (400 MHz, CDCl_3_) δ (ppm): 6.45 (dd, *J* = 14.2, 6.7 Hz, 1H), 6.15–6.06 (m, 1H), 4.51 (t, *J* = 6.6 Hz, 1H), 4.40 (dd, *J* = 14.2, 1.8 Hz, 1H), 4.10 (dd, *J* = 6.7, 1.8 Hz, 1H), 2.17–2.00 (m, 4H), 1.89–1.68 (m, 2H), 1.68–1.51 (m, 4H), 1.50–1.29 (m, 4H), 0.91 (t, *J* = 7.2 Hz, 3H). Spectroscopical data were identical to those reported for the corresponding racemic compound.^12a^

##### (*R*)-(+)-1-(4-Methyl-3-vinyloxypent-1-ynyl)­cyclohexene (**14d**)

Sonogashira coupling of **11a** (351 μL, 2.0 mmol) and (*±*)-4-methyl-1-pentyn-3-ol (211 μL, 2.0 mmol), followed by purification via flash chromatography (*n*-hexane/EtOAc, 6:1 + 1% Et_3_N; *R*_f_ = 0.36), afforded the enynyl alcohol intermediate **13d** (335 mg, 94%), which was used as the substrate for the enzymatic kinetic resolution with CAL-B (see Supporting Information). ^1^H NMR (200 MHz, CDCl_3_) δ (ppm): 6.12–6.08 (m, 1H), 4.30–4.25 (m, 1H), 2.13–2.04 (m, 4H), 1.96–1.79 (m, 1H), 1.69–1.51 (m, 4H), 1.01 (d, *J* = 6.6 Hz, 3H), 0.99 (d, *J* = 6.8 Hz, 3H). Vinylation of (*R*)-**13d** (87 mg, 0.49 mmol; ee, 97%) afforded (*R*)-**14d**, which was purified by flash chromatography (*n*-hexane/EtOAc, 60:1 + 1% Et_3_N; *R*_f_ = 0.58). Pure (*R*)-**14d** was obtained as a pale yellow oil (65 mg, 65%). ee, 97%. [α]_D_^23^ +42.7 (*c* 1.0, CHCl_3_). ^1^H NMR (400 MHz, CDCl_3_) δ (ppm): 6.45 (dd, *J* = 14.0, 6.8 Hz, 1H), 6.13–6.10 (m, 1H), 4.40 (dd, *J* = 14.0, 1.6 Hz, 1H), 4.30 (d, *J* = 5.6 Hz, 1H), 4.09 (dd, *J* = 6.8, 1.6 Hz, 1H), 2.14–2.05 (m, 4H), 2.05–1.96 (m, 1H), 1.66–1.54 (m, 4H), 1.03 (d, *J* = 6.8 Hz, 3H), 1.01 (d, *J* = 6.4 Hz, 3H). Spectroscopical data were identical to those reported for the corresponding racemic compound.^12^c

##### (*S*)-(−)-1-(3-Vinyloxybut-1-ynyl)­cycloheptene (**14e**)

Sonogashira coupling of **11b** (208 mg, 0.85 mmol) and (*S*)-3-butyn-2-ol (67 μL, 0.85 mmol; ee, 99%), followed by purification via flash chromatography (*n*-hexane/EtOAc, 5:1 + 1% Et_3_N; *R*_f_ = 0.33), afforded the enynyl alcohol intermediate (*S*)-**13e** (135 mg, 88%), which was used immediately in the next step. ^1^H NMR (400 MHz, CDCl_3_) δ (ppm): 6.27 (t, *J* = 6.7 Hz, 1H), 4.63 (q, *J* = 6.2 Hz, 1H), 2.35–2.27 (m, 2H), 2.19–2.14 (m, 2H), 1.84 (br s, OH), 1.77–1.69 (m, 2H), 1.59–1.47 (m, 4H), 1.45 (d, *J* = 6.2 Hz, 3H). Vinylation of (*S*)-**13e** (125 mg, 0.76 mmol) afforded (*S*)-**14e**, which was purified by flash chromatography (*n*-hexane +1% Et_3_N; *R*_f_ = 0.42). Pure (*S*)-**14e** was obtained as a pale yellow oil (92 mg, 64%). ee, 99%. [α]_D_^24^ −58.6 (*c* 1.0, CHCl_3_). ^1^H NMR (400 MHz, CDCl_3_) δ (ppm): 6.43 (dd, *J* = 14.2, 6.7 Hz, 1H), 6.28 (t, *J* = 6.7 Hz, 1H), 4.64 (q, *J* = 6.6 Hz, 1H), 4.40 (dd, *J* = 14.2, 1.7 Hz, 1H), 4.10 (dd, *J* = 6.7, 1.7 Hz, 1H), 2.37–2.23 (m, 2H), 2.19–2.14 (m, 2H), 1.77–1.67 (m, 2H), 1.62–1.44 (m, 4H), 1.49 (d, *J* = 6.6 Hz, 3H). Spectroscopical data were identical to those reported for the corresponding racemic compound.^12a^

##### (*S*)-(−)-1-(3-Vinyloxyhept-1-ynyl)­cycloheptene (**14f**)

Sonogashira coupling of **11b** (254 mg, 1.04 mmol) and (*S*)-1-heptyn-3-ol (117 mg, 1.04 mmol; ee, 99%), followed by purification by flash chromatography (*n*-hexane/EtOAc, 9:1 + 1% Et_3_N; *R*_f_ = 0.28), afforded the enynyl alcohol intermediate (*S*)-**13f** (193 mg, 90%), which was used immediately in the next step. ^1^H NMR (400 MHz, CDCl_3_) δ (ppm): 6.28 (t, *J* = 6.8 Hz, 1H), 4.47 (t, *J* = 6.8 Hz, 1H), 2.33–2.30 (m, 2H), 2.20–2.15 (m, 2H), 1.76–1.67 (m, 4H), 1.58–1.47 (m, 4H), 1.45–1.31 (m, 4H), 0.92 (t, *J* = 7.2 Hz, 3H). Vinylation of (*S*)-**13f** (193 mg, 0.94 mmol) afforded (*S*)-**14f**, which was purified by flash chromatography (*n*-hexane/EtOAc, 2:1 + 1% Et_3_N; *R*_f_ = 0.54). Pure (*S*)-**14f** was obtained as a pale yellow oil (163 mg, 75%). ee, 99%; [α]_D_^25^ −66.3 (*c* 1.0, CHCl_3_). ^1^H NMR (400 MHz, CDCl_3_) δ (ppm): 6.44 (dd, *J* = 14.0, 6.4 Hz, 1H), 6.29 (t, *J* = 6.8 Hz, 1H), 4.51 (t, *J* = 6.8 Hz, 1H), 4.40 (dd, *J* = 14.0, 1.6 Hz, 1H), 4.09 (dd, *J* = 6.4, 1.6 Hz, 1H), 2.32–2.29 (m, 2H), 2.19–2.15 (m, 2H), 1.84–1.70 (m, 4H), 1.58–1.40 (m, 6H), 1.39–1.30 (m, 2H), 0.91 (t, *J* = 7.2 Hz, 3H). Spectroscopical data were identical to those reported for the corresponding racemic compound.^12^c

##### (*S*)-(−)-4-(3-Vinyloxybut-1-ynyl)-3,6-dihydro-2*H*-pyran (**14g**)

Sonogashira coupling of **11c** (186 mg, 0.81 mmol) and (*S*)-3-butyn-2-ol (64 μL, 0.81 mmol; ee, 99%), followed by purification by flash chromatography (*n*-hexane/EtOAc, 3:1 + 1% Et_3_N; *R*_f_ = 0.24), afforded the enynyl alcohol intermediate (*S*)-**13g** (98 mg, 79%), which was used immediately in the next step. ^1^H NMR (200 MHz, CDCl_3_) δ (ppm): 6.09–6.03 (m, 1H), 4.65 (q, *J* = 6.0 Hz, 1H), 4.18 (q, *J* = 2.8 Hz, 2H), 3.77 (t, *J* = 5.6 Hz, 2H), 2.27–2.18 (m, 2H), 1.79 (d, *J* = 5.2 Hz, 1H), 1.47 (d, *J* = 6.6 Hz, 3H). Vinylation of (*S*)-**13g** (93 mg, 0.61 mmol) afforded (*S*)-**14g**, which was purified by flash chromatography (*n*-hexane +1% Et_3_N; *R*_f_ = 0.18). Pure (S)-**14g** was obtained as a pale yellow oil (68 mg, 63%). ee, 99%. [α]_D_^24^ −116.1 (*c* 1.0, CHCl_3_). ^1^H NMR (400 MHz, CDCl_3_) δ (ppm): 6.43 (dd, *J* = 14.4, 6.8 Hz, 1H), 6.08–6.07 (m, 1H), 4.66 (q, *J* = 6.4 Hz, 1H), 4.41 (dd, *J* = 14.4, 2.0 Hz, 1H), 4.19–4.16 (m, 2H), 4.12 (dd, *J* = 6.8, 2.0 Hz, 1H), 3.76 (t, *J* = 5.2 Hz, 2H), 2.24–2.19 (m, 2H), 1.52 (d, *J* = 6.4 Hz, 3H). ^13^C­{^1^H} NMR (100.4 MHz, CDCl_3_) δ (ppm): 149.5, 133.0, 117.8, 89.7, 86.9, 85.3, 65.3, 65.0, 63.8, 28.9, 21.8. GC-MS (EI) *m/z* (%): 178 ([M]^+^, 1), 135 (75), 91 (100), 79 (57). HRMS (ESI Orbitrap) *m/z*: [M + H]^+^ calcd for C_11_H_15_O_2_: 179.1072. Found: 179.1068.

##### (*R*)-(+)-4-(3-Vinyloxyhept-1-ynyl)-3,6-dihydro-2*H*-pyran (**14h**)

Sonogashira coupling of **11c** (280 mg, 1.2 mmol) and (*R*)-1-heptyn-3-ol (135 mg, 1.2 mmol; ee, 99%), followed by purification by flash chromatography (*n*-hexane/EtOAc, 3:1 + 1% Et_3_N; *R*_f_ = 0.24), afforded the enynyl alcohol intermediate (*R*)-**13h** (161 mg, 69%), which was used immediately in the next step. ^1^H NMR (400 MHz, CDCl_3_) δ (ppm): 6.07–6.04 (m, 1H), 4.48 (q, *J* = 6.4 Hz, 1H), 4.19 (q, *J* = 2.8 Hz, 2H), 3.78 (t, *J* = 5.6 Hz, 2H), 2.25–2.20 (m, 2H), 1.83 (d, *J* = 5.2 Hz, 1H), 1.75–1.68 (m, 2H), 1.48–1.31 (m, 4H), 0.92 (t, *J* = 7.2 Hz, 3H). Vinylation of (*R*)-**13h** (161 mg, 0.83 mmol) afforded (*R*)-**14h**, which was purified by flash chromatography (Et_2_O/*n*-hexane, 1:20 + 1% Et_3_N, 1:20; *R*_f_ = 0.21). Pure (*R*)-**14h** was obtained as a colorless oil (107 mg, 59%). ee, 99%. [α]_D_^25^ +64.3 (*c* 1.0, CHCl_3_). ^1^H NMR (400 MHz, CDCl_3_) δ (ppm): 6.43 (dd, *J* = 14.4, 6.8 Hz, 1H), 6.09–6.05 (m, 1H), 4.51 (t, *J* = 6.8 Hz, 1H), 4.40 (dd, *J* = 14.4, 2.0 Hz, 1H), 4.19–4.16 (m, 2H), 4.11 (dd, *J* = 6.8, 2.0 Hz, 1H), 3.77 (t, *J* = 5.2 Hz, 2H), 2.24–2.20 (m, 2H), 1.87–1.72 (m, 2H), 1.48–1.31 (m, 4H), 0.91 (t, *J* = 7.2 Hz, 3H). ^13^C­{^1^H} NMR (100.4 MHz, CDCl_3_) δ (ppm): 149.8, 132.9, 117.8, 89.5, 86.2, 86.0, 69.2, 65.3, 63.8, 35.1, 29.0, 27.2, 22.3, 14.0. GC-MS (EI) *m/z* (%): 218 ([M]^+^, 1), 177 (3), 105 (47), 91 (100), 77 (84). Anal. Calcd for C_14_H_20_O_2_: C, 76.33; H, 9.15. Found: C, 75.86; H, 9.06.

##### (*S*)-(−)-1-Tosyl-4-(3-vinyloxybut-1-ynyl)-1,2,3,6-tetrahydropyridine (**14i**)

Sonogashira coupling of **11d** (763 mg, 1.98 mmol) and (*S*)-3-butyn-2-ol (155 μL, 1.98 mmol; ee, 99%), followed by purification via flash chromatography (*n*-hexane/EtOAc, 2:1 + 1% Et_3_N; *R*_f_ = 0.13), afforded the enynyl alcohol intermediate (*S*)-**13i** (441 mg, 73%), which was used immediately in the next step. ^1^H NMR (200 MHz, CDCl_3_) δ (ppm): 7.66 (d, *J* = 8.2 Hz, 2H), 7.32 (d, *J* = 8.2 Hz, 2H), 5.96–5.90 (m, 1H), 4.62 (q, *J* = 6.6 Hz, 1H), 3.64 (q, *J* = 3.0 Hz, 2H), 3.17 (t, *J* = 5.6 Hz, 2H), 2.43 (s, 3H), 2.35–2.26 (m, 2H), 1.44 (d, *J* = 6.6 Hz, 3H). Vinylation of (*S*)-**13i** (355 mg, 1.16 mmol) afforded (*S*)-**14i**, which was purified by flash chromatography (EtOAc/*n*-hexane, 1:6 + 1% Et_3_N; *R*_f_ = 0.39). Pure (*S*)-**14i** was obtained as a thick, colorless oil (238 mg, 62%). ee, 99%. [α]_D_^24^ −58.9 (*c* 1.04, CHCl_3_). ^1^H NMR (400 MHz, CDCl_3_) δ (ppm): 7.65 (d, *J* = 8.4 Hz, 2H), 7.32 (d, *J* = 8.4 Hz, 2H), 6.38 (dd, *J* = 14.4, 6.8 Hz, 1H), 5.97–5.92 (m, 1H), 4.62 (q, *J* = 6.8 Hz, 1H), 4.37 (dd, *J* = 14.4, 2.0 Hz, 1H), 4.10 (dd, *J* = 6.8, 2.0 Hz, 1H), 3.64–3.61 (m, 2H), 3.16 (t, *J* = 5.6 Hz, 2H), 2.42 (s, 3H), 2.33–2.27 (m, 2H), 1.48 (d, *J* = 6.4 Hz, 3H). ^13^C­{^1^H} NMR (100.4 MHz, CDCl_3_) δ (ppm): 149.5, 143.7, 133.0, 129.7 (2C), 129.2, 127.6 (2C), 118.6, 89.7, 87.6, 84.8, 64.9, 44.9, 42.3, 29.2, 21.7, 21.5. MS (ESI) *m/z* (%): 684 ([2M + Na]^+^, 100), 354 ([M + Na]^+^, 67). Anal. Calcd for C_18_H_21_NO_3_S: C, 65.23; H, 6.39; N, 4.23; S, 9.67. Found: C, 64.94; H, 6.49; N, 4.07; S, 9.63.

##### (*S*)-(−)-4-(3-Vinyloxybut-1-ynyl)-1,2-dihydronaphtalene (**14j**)

Sonogashira coupling of **11e** (248 mg, 0.89 mmol) and (*S*)-3-butyn-2-ol (105 μL, 1.34 mmol, 1.5 equiv; ee, 99%), followed by purification by flash chromatography (*n*-hexane/EtOAc, 5:1 + 1% Et_3_N; *R*_f_ = 0.27), afforded the enynyl alcohol intermediate (*S*)-**13j** (156 mg, 88%), which was used immediately in the next step. ^1^H NMR (400 MHz, CDCl_3_) δ (ppm): 7.53 (d, *J* = 7.6 Hz, 1H), 7.25–7.20 (m, 1H), 7.20–7.16 (m, 1H), 7.11 (d, *J* = 7.2 Hz, 1H), 6.46 (t, *J* = 4.8 Hz, 1H), 4.81–4.74 (m, 1H), 2.79 (t, *J* = 8.0 Hz, 2H), 2.41–2.36 (m, 2H), 1.89 (d, *J* = 5.2 Hz, 1H), 1.57 (d, *J* = 6.4 Hz, 3H). Vinylation of (*S*)-**13j** (152 mg, 0.76 mmol) afforded (*S*)-**14j**, which was purified by flash chromatography (EtOAc/*n*-hexane, 1:10 + 1% Et_3_N; *R*_f_ = 0.65). Pure (*S*)-**14j** was obtained as a pale yellow oil (105 mg, 61%). ee, 99%. [α]_D_^20^ −92.0 (*c* 0.74, CHCl_3_). ^1^H NMR (400 MHz, CDCl_3_) δ (ppm): 7.52 (d, *J* = 7.6 Hz, 1H), 7.25–7.16 (m, 2H), 7.11 (d, *J* = 7.2 Hz, 1H), 6.52 (dd, *J* = 14.4, 6.8 Hz, 1H), 6.47 (t, *J* = 4.8 Hz, 1H), 4.78 (q, *J* = 6.8 Hz, 1H), 4.49 (dd, *J* = 14.4, 2.0 Hz, 1H), 4.18 (dd, *J* = 6.8, 2.0 Hz, 1H), 2.79 (t, *J* = 8.0 Hz, 2H), 2.41–2.36 (m, 2H), 1.62 (d, *J* = 6.8 Hz, 3H). ^13^C­{^1^H} NMR (100.4 MHz, CDCl_3_) δ (ppm): 149.7, 136.1, 134.9, 132.4, 127.7, 127.3, 126.6, 124.9, 121.0, 89.7, 88.8, 83.5, 65.0, 27.0, 23.5, 22.0. GC-MS (EI) *m/z* (%): 224 ([M]^+^, 47), 195 (35), 167 (100), 165 (98), 153 (46), 115 (35). Anal. Calcd for C_16_H_16_O: C, 85.68; H, 7.19. Found: C, 85.92; H, 6.97.

##### (*S*)-(−)-3-(3-Vinyloxyoct-1-ynyl)-1,2-dihydronaphtalene (**14k**)

Sonogashira coupling of **11e** (252 mg, 0.91 mmol) and (*S*)-1-octyn-3-ol (198 μL, 1.36 mmol, 1.5 equiv; ee, 97%), followed by purification by flash chromatography (*n*-hexane/EtOAc, 10:1 + 1% Et_3_N; *R*_f_ = 0.20), afforded (*S*)-**13k** (220 mg, 95%), which was used immediately in the next step. ^1^H NMR (400 MHz, CDCl_3_) δ (ppm): 7.53 (d, *J* = 7.6 Hz, 1H), 7.25–7.15 (m, 2H), 7.13–7.09 (m, 1H), 6.45 (t, *J* = 4.8 Hz, 1H), 4.61 (t, *J* = 6.8 Hz, 1H), 2.80 (t, *J* = 8.0 Hz, 2H), 2.41–2.36 (m, 2H), 1.84–1.78 (m, 2H), 1.57–1.49 (m, 2H), 1.38–1.32 (m, 4H), 0.91 (t, *J* = 7.2 Hz, 3H). Vinylation of (*S*)-**13k** (209 mg, 0.82 mmol) afforded (*S*)-**14k**, which was purified by flash chromatography (EtOAc/*n*-hexane, 1:10 + 1% Et_3_N; *R*_f_ = 0.66). Pure (*S*)-**14k** was obtained as a pale, colorless oil (163 mg, 71%). ee, 97%. [α]_D_^20^ −62.1 (*c* 0.88, CHCl_3_). ^1^H NMR (400 MHz, CDCl_3_) δ (ppm): 7.54 (d, *J* = 7.6 Hz, 1H), 7.26–7.17 (m, 2H), 7.11 (d, *J* = 6.8 Hz, 1H), 6.53 (dd, *J* = 14.4, 6.8 Hz, 1H), 6.48 (t, *J* = 4.8 Hz, 1H), 4.66 (t, *J* = 6.8 Hz, 1H), 4.50 (dd, *J* = 14.4, 2.0 Hz, 1H), 4.18 (dd, *J* = 6.8, 2.0 Hz, 1H), 2.80 (t, *J* = 8.0 Hz, 2H), 2.42–2.36 (m, 2H), 1.95–1.85 (m, 2H), 1.61–1.51 (m, 2H), 1.40–1.33 (m, 4H), 0.93 (t, *J* = 6.8 Hz, 3H). ^13^C­{^1^H} NMR (100.4 MHz, CDCl_3_) δ (ppm): 149.9, 136.0, 134.9, 132.4, 127.6, 127.3, 126.6, 125.0, 121.1, 89.5, 88.1, 84.2, 69.3, 35.5, 31.4, 27.0, 24.9, 23.6, 22.5, 14.0. GC-MS (EI) *m/z* (%): 280 ([M]^+^, 47), 209 (50), 181 (91), 167 (100), 115 (45). Anal. Calcd for C_20_H_24_O: C, 85.67; H, 8.63. Found: C, 85.39; H, 8.64.

##### (*S*)-(−)-1-(3-Vinyloxyoct-1-ynyl)­cyclopentene (**14l**)

Sonogashira coupling of **11f** (432 mg, 2.0 mmol) and (*S*)-1-octyn-3-ol (292 μL, 2.0 mmol; ee, 97%), followed by purification via flash chromatography (*n*-hexane/EtOAc, 10:1 + 1% Et_3_N; *R*_f_ = 0.20), afforded (*S*)-**13l** (245 mg, 64%), which was used immediately in the next step. ^1^H NMR (400 MHz, CDCl_3_) δ (ppm): 6.05–6.02 (m, 1H), 4.50 (t, *J* = 6.8 Hz, 1H), 2.47–2.39 (m, 4H), 1.93–1.85 (m, 2H), 1.75–1.67 (m, 2H), 1.49–1.41 (m, 2H), 1.35–1.27 (m, 4H), 0.89 (t, *J* = 6.8 Hz, 3H). Vinylation of (*S*)-**13l** (245 mg, 1.27 mmol) afforded (*S*)-**14l**, which was purified by flash chromatography (EtOAc/*n*-hexane, 1:10 + 1% Et_3_N; *R*_f_ = 0.76). Pure (*S*)-**14l** was obtained as a colorless oil (178 mg, 64%). ee, 97%. [α]_D_^18^ −70.5 (*c* 0.94, CHCl_3_). ^1^H NMR (400 MHz, CDCl_3_) δ (ppm): 6.45 (dd, *J* = 14.0, 6.8 Hz, 1H), 6.07–6.05 (m, 1H), 4.53 (t, *J* = 6.4 Hz, 1H), 4.41 (dd, *J* = 14.0, 2.0 Hz, 1H), 4.11 (dd, *J* = 6.4, 2.0 Hz, 1H), 2.64–2.38 (m, 4H), 1.93–1.85 (m, 2H), 1.83–1.74 (m, 2H), 1.50–1.43 (m, 2H), 1.33–1.29 (m, 4H), 0.89 (t, *J* = 7.2 Hz, 3H). ^13^C­{^1^H} NMR (100.4 MHz, CDCl_3_) δ (ppm): 149.8, 138.5, 123.7, 89.4, 88.2, 83.6, 69.4, 36.3, 35.4, 33.2, 31.4, 24.8, 23.2, 22.5, 14.0. GC-MS (EI) *m/z* (%): 218 ([M]^+^, 1), 161 (14), 147 (17), 119 (29), 105 (57), 91 (100). Anal. Calcd for C_15_H_22_O: C, 82.52; H, 10.16. Found: C, 82.31; H, 9.95.

#### General Procedure for the Propargyl Claisen Rearrangement/Nazarov Cyclization Reaction

The solution of propargyl vinyl ether **14** in the eluent containing Et_3_N was concentrated and dried under *vacuum* for 30 min just prior to use. To a solution of the commercially available gold­(I) complex IPrAuBF_4_ (2 mol %) in DCM (3 mL) stirred at 25 °C under a nitrogen atmosphere was added a solution of propargyl vinyl ether **14** (0.3 mmol) in DCM (3 mL; final concentration 0.05 M), and the reaction mixture was stirred at 25 °C until complete consumption of the starting material (TLC monitoring, usually 15 min). The mixture was diluted with MeOH (12 mL), and NaBH_4_ (12 mg, 0.3 mmol) was immediately added. After 10 min, the reduction was completed. *Work-up A*. The solvent was then evaporated, water was added to the residue (10 mL), and the product was extracted with DCM (3 × 10 mL). The combined organic extracts were washed with brine (20 mL) and dried over anhydrous Na_2_SO_4_. After filtration and evaporation of the solvent, the crude oil was purified by flash chromatography to afford the corresponding alcohol **15**. *Work-up B*. Water (5 mL) was added to the solution, and the mixture was concentrated under *vacuum* to a small volume. Water was added once more (up to 10 mL), and the product was extracted with DCM (3 × 10 mL). The combined organic extracts were washed with brine (20 mL) and dried over anhydrous Na_2_SO_4_. After filtration and evaporation of the solvent, the crude oil was purified by flash chromatography to afford the corresponding alcohol **15**.

##### (*S*)-(−)-2-(3-Methyl-4,5,6,7-tetrahydro-3a*H*-inden-1-yl)­ethanol (**15a**)

Prepared following the General procedure and Work-up A, starting from (*S*)-**14a** (237 mg, 1.34 mmol, ee, 99%) and obtaining pure (*S*)-**15a** (206 mg, 1.16 mmol, 86%) after purification by flash chromatography (eluent: *n*-hexane/EtOAc, 4:1 + 1% Et_3_N; *R*_f_ = 0.36). ee, 96%. [α]_D_^23^ −41.3 (*c* 1.0, CHCl_3_). ^1^H NMR (400 MHz, CDCl_3_) δ (ppm): 5.93 (s, 1H), 3.70–3.65 (m, 2H), 2.68–2.62 (m, 1H), 2.52–2.43 (m, 3H), 2.31–2.23 (m, 1H), 2.07 (td, *J* = 13.2, 5.2 Hz, 1H), 1.96–1.93 (m, 1H), 1.92 (s, 3H), 1.83–1.72 (m, 1H), 1.47 (br s, 1H), 1.46–1.35 (m, 1H), 1.12–1.00 (m, 1H), 0.71 (qd, *J* = 12.8, 3.2 Hz, 1H). Spectroscopical data identical to those reported for the corresponding racemic compound.^12a^

##### (*S*)-(−)-2-(3-Phenyl-4,5,6,7-tetrahydro-3a*H*-inden-1-yl)­ethanol (**15b**)

Prepared following the General procedure and Work-up A, starting from (*S*)-**14b** (81 mg, 0.34 mmol, ee, 99%) and obtaining pure (*S*)-**15b** (68 mg, 83%) after purification by flash chromatography (eluent: *n*-hexane/EtOAc, 4:1 + 1% Et_3_N; *R*_f_ = 0.15). ee, 98%. [α]_D_^17^ −50.5 (*c* 1.0, CHCl_3_). ^1^H NMR (400 MHz, CDCl_3_) δ (ppm): 7.45–7.39 (m, 2H), 7.36–7.29 (m, 2H), 7.21–7.16 (m, 1H), 6.71 (s, 1H), 3.77 (t, *J* = 6.4 Hz, 2H), 3.15 (dd, *J* = 12.4, 5.6 Hz, 1H), 2.81–2.76 (m, 1H), 2.65–2.57 (m, 2H), 2.43–2.38 (m, 1H), 2.21 (td, *J* = 13.2, 5.2 Hz, 1H), 2.08–1.99 (m, 1H), 1.85–1.78 (m, 1H), 1.71 (br s, 1H, OH), 1.53 (qt, *J* = 13.2, 3.2 Hz, 1H), 1.18 (qt, *J* = 13.2, 4.0 Hz, 1H), 0.83 (qd, *J* = 12.8, 3.6 Hz, 1H). ^13^C­{^1^H} NMR (100.4 MHz, CDCl_3_) δ (ppm): 148.7, 147.9, 135.4, 130.9, 128.8, 128.5 (2C), 126.1, 125.4 (2C), 62.1, 52.2, 33.8, 30.2, 29.6, 26.3, 25.7. GC-MS (EI) *m/z* (%): 240 ([M]^+^, 26), 209 (66), 167 (39), 115 (39), 91 (100). Anal. Calcd for C_17_H_20_O: C, 84.96; H, 8.25. Found: C, 84.90; H, 8.39.

##### (*R*)-(+)-2-(3-Butyl-4,5,6,7-tetrahydro-3a*H*-inden-1-yl)­ethanol (**15c**)

Prepared following the General procedure and Work-up A, starting from (*R*)-**14c** (43 mg, 0.20 mmol, ee, 92%) and obtaining pure (*R*)-**15c** (37 mg, 85%) after purification by flash chromatography (eluent: *n*-hexane/EtOAc, 4:1 + 1% Et_3_N; *R*_f_ = 0.36). ee, 85%. [α]_D_^22^ +31.8 (*c* 1.0, CHCl_3_). ^1^H NMR (400 MHz, CDCl_3_) δ (ppm): 5.93 (s, 1H), 3.69 (t, *J* = 6.4 Hz, 2H), 2.70–2.63 (m, 1H), 2.58–2.46 (m, 3H), 2.33–2.19 (m, 3H), 2.08 (td, *J* = 13.4, 5.0 Hz, 1H), 2.00–1.88 (m, 1H), 1.85–1.73 (m, 1H), 1.57–1.27 (m, 5H + OH), 1.15–0.98 (m, 1H), 0.91 (t, *J* = 7.3 Hz, 3H), 0.73 (qd, *J* = 12.7, 3.3 Hz, 1H). Spectroscopical data were identical to those reported for the corresponding racemic compound.^12a^

##### (*R*)-(+)-2-(3-Isopropyl-4,5,6,7-tetrahydro-3a*H*-inden-1-yl)­ethanol (**15d**)

Prepared following the General procedure and Work-up A, starting from (*R*)-**14d** (61 mg, 0.30 mmol, ee, 97%) and obtaining pure (*R*)-**15d** (54 mg, 88%) after purification by flash chromatography (EtOAc/*n*-hexane, 1:4 + 1% Et_3_N; *R*_f_ = 0.32). ee, 93%. Colorless oil. [α]_D_^25^ +44.7 (*c* 1.0, CHCl_3_). ^1^H NMR (400 MHz, CDCl_3_) δ (ppm): 5.92 (s, 1H), 3.72–3.67 (m, 2H), 2.68–2.61 (m, 2H), 2.58–2.47 (m, 1H), 2.50 (t, *J* = 6.8 Hz, 2H), 2.31–2.25 (m, 1H), 2.07 (td, *J* = 13.2, 4.8 Hz, 1H), 1.97–1.89 (m, 1H), 1.82–1.74 (m, 1H), 1.49 (m, 1H, OH), 1.41 (qt, *J* = 13.2, 3.2 Hz, 1H), 1.14 (d, *J* = 6.8 Hz, 3H), 1.11–1.00 (m, 1H), 1.07 (d, *J* = 7.2 Hz, 3H), 0.75 (qd, *J* = 12.8, 3.2 Hz, 1H). ^13^C­{^1^H} NMR (100.4 MHz, CDCl_3_) δ (ppm): 158.2, 144.7, 129.5, 124.6, 61.9, 53.1, 32.0, 30.3, 29.1, 27.5, 25.9, 25.7, 24.2, 21.3. GC-MS (EI) *m/z* (%): 206 ([M]^+^, 17), 175 (76), 147 (24), 119 (51), 105 (65), 91 (100). Anal. Calcd for C_14_H_22_O: C, 81.50; H, 10.75. Found: C, 81.36; H, 10.64.

##### (*S*)-(−)-2-(3-Methyl-3a,4,5,6,7,8-hexahydroazulen-1-yl)­ethanol (**15e**)

Prepared following the General procedure and Work-up A, starting from (*S*)-**14e** (67 mg, 0.35 mmol, ee, 99%) and obtaining crude (*S*)-**15e** (68 mg, quantitative), as a colorless oil, which was used as such in the cycloaddition reaction for enantiomeric excess determination. ee, 94%. [α]_D_^21^ −39.1 (*c* 1.0, CH_3_OH). ^1^H NMR (400 MHz, CD_3_OD) (crude reaction mixture) δ (ppm): 5.89 (t, *J* = 1.6 Hz, 1H), 3.59 (t, *J* = 7.2 Hz, 2H), 2.74–2.70 (m, 1H), 2.61–2.39 (m, 4H), 2.08–2.02 (m, 1H), 1.91–1.84 (m, 4H), 1.80–1.67 (m, 2H), 1.67–1.47 (m, 2H), 1.38–1.28 (m, 2H), 1.05–0.95 (m, 1H). Spectroscopical data were identical to those reported for the corresponding racemic compound.^12a^

##### (*S*)-(−)-2-(3-Butyl-3a,4,5,6,7,8-hexahydroazulen-1-yl)­ethanol (**15f**)

Prepared following the General procedure and Work-up A, starting from (*S*)-**14f** (56 mg, 0.24 mmol, ee, 99%) and obtaining crude (*S*)-**15f** (55 mg, 98%), as a colorless oil, in a sufficiently pure form to be used for the spectroscopic characterization and in the cycloaddition reaction for enantiomeric excess determination. ee, 94%. [α]_D_^25^ −30.2 (*c* 1.0, CH_3_OH). ^1^H NMR (400 MHz, CD_3_OD) (95:5 mixture of regioisomers, major isomer reported) δ (ppm): 5.91 (s, 1H), 3.60 (t, *J* = 7.2 Hz, 2H), 2.82–2.76 (m, 1H), 2.62–2.47 (m, 2H), 2.46–2.39 (m, 2H), 2.36–2.13 (m, 3H), 2.06–1.98 (m, 1H), 1.92–1.82 (m, 1H), 1.81–1.67 (m, 2H), 1.67–1.29 (m, 6H), 1.05–0.96 (m, 1H), 0.93 (t, *J* = 7.2 Hz, 3H), 0.95–0.89 (m, 1H). ^13^C­{^1^H} NMR (100.4 MHz, CD_3_OD) (mixture of regioisomers, major isomer reported) δ (ppm): 150.9, 146.7, 134.9, 128.3, 62.3, 57.8, 33.0, 32.5, 32.0, 31.4, 31.0, 29.5, 29.4, 29.1, 23.7, 14.4. MS (ESI) *m/z* (%): 235 ([M + 1]^+^, 100). HRMS (ESI Orbitrap) *m/z*: [M + H]^+^ calcd for C_16_H_27_O: 235.2062. Found: 235.2054.

##### (*S*)-(−)-2-(7-Methyl-1,3,4,7a-tetrahydrocyclopenta­[c]­pyran-5-yl)­ethanol (**15g**)

Prepared following the General procedure and Work-up A, starting from (*S*)-**14g** (55 mg, 0.31 mmol, ee, 99%) and obtaining pure (*S*)-**15g** (47 mg, 85%) as a colorless oil after purification by flash chromatography (EtOAc/*n*-hexane, 1:3 + 1% Et_3_N; *R*_f_ = 0.28), as a 10:1 mixture of regioisomers. ee, 94%. [α]_D_^25^ −32.5 (*c* 1.0, CHCl_3_). ^1^H NMR (400 MHz, CD_3_OD) (10:1 mixture of regioisomers, major isomer reported) δ (ppm): 6.09 (s, 1H), 4.46 (dd, *J* = 10.0, 6.8 Hz, 1H), 4.10 (dd, *J* = 10.4, 5.6 Hz, 1H), 3.60 (td, *J* = 7.2, 1.2 Hz, 2H), 3.00–2.94 (m, 1H), 2.84–2.76 (m, 1H), 2.65–2.59 (m, 2H), 2.51–2.36 (m, 3H), 1.90 (s, 3H). ^13^C­{^1^H} NMR (100.4 MHz, CD_3_OD) (mixture of regioisomers, major isomer reported) δ (ppm): 143.3, 139.5, 134.2, 132.0, 74.6, 70.5, 62.6, 56.3, 31.3, 28.7, 14.5. GC-MS (EI) *m/z* (%): 180 ([M]^+^, 18), 149 (33), 119 (100), 105 (28), 91 (60). Anal. Calcd for C_11_H_16_O_2_: C, 73.30; H, 8.95. Found: C, 73.50; H, 8.76.

##### (*R*)-(+)-2-(7-Butyl-1,3,4,7a-tetrahydrocyclopenta­[c]­pyran-5-yl)­ethanol (**15h**)

Prepared following the General procedure and Work-up B, starting from (*R*)-**14h** (56 mg, 0.26 mmol, ee, 99%) and obtaining crude (*R*)-**15h** (50 mg, 86%), as a colorless oil, that was fully characterized without further purification to avoid double-bond isomerization in the cyclopentadienyl moiety. ESI-MS and HRMS analyses were performed on a purified sample (eluent: *n*-hexane/EtOAc, 3:1 + 1% Et_3_N; *R*_f_ = 0.32). ee, 99%. [α]_D_^25^ +24.5 (*c* 1.0, CHCl_3_). ^1^H NMR (400 MHz, CD_3_OD) (crude reaction mixture) δ (ppm): 6.10 (s, 1H), 4.45 (dd, *J* = 10.4, 6.8 Hz, 1H), 4.10 (dd, *J* = 10.4, 6.0 Hz, 1H), 3.61 (td, *J* = 7.2, 1.2 Hz, 2H), 3.00–2.93 (m, 1H), 2.87 (dd, *J* = 10.8, 6.8 Hz, 1H), 2.67–2.58 (m, 2H), 2.49 (t, *J* = 7.2 Hz, 2H), 2.46–2.38 (m, 1H), 2.26–2.21 (m, 2H), 1.54–1.43 (m, 2H), 1.38–1.31 (m, 2H), 0.93 (t, *J* = 7.2 Hz, 3H). ^13^C­{^1^H} NMR (100.4 MHz, CD_3_OD) (crude reaction mixture) δ (ppm): 148.4, 139.4, 134.0, 131.0, 74.6, 70.4, 62.6, 55.2, 32.6, 31.3, 29.9, 28.7, 23.6, 14.3. MS (ESI) *m/z* (%): 245 ([M + Na]^+^, 100), 223 ([M + 1]^+^, 71). HRMS (ESI Orbitrap) *m/z*: [M + H]^+^ calcd for C_14_H_23_O_2_: 223.1698. Found: 223.1692.

##### (*S*)-(−)-2-(7-Methyl-2tosyl-2,3,4,7a-tetrahydro-1*H*-[2]­pyrindin-5-yl)­ethanol (**15i**)

Prepared following the General procedure and Work-up B, starting from (*S*)-**14i** (78 mg, 0.24 mmol, ee, 99%) and obtaining crude (*S*)-**15i** (67 mg, 84%), as a pale yellow oil, that was fully characterized without further purification to avoid isomerization of the cyclopentadienyl moiety. ESI-MS and HRMS analyses were performed on a purified sample (eluent: EtOAc/*n*-hexane, 1:1 + 1% Et_3_N; *R*_f_ = 0.38). ee, 98%. [α]_D_^23^ −57.7 (*c* 1.0, CH_3_OH). ^1^H NMR (400 MHz, CD_3_OD) δ (ppm): 7.67 (d, *J* = 8.4 Hz, 2H), 7.38 (d, *J* = 8.0 Hz, 2H), 6.00 (s, 1H), 4.28 (ddd, *J* = 10.8, 6.4, 1.6 Hz, 1H), 3.98–3.94 (m, 1H), 3.56–3.50 (m, 2H), 2.75 (dd, *J* = 11.6, 6.0 Hz, 1H), 2.66–2.60 (m, 1H), 2.41 (s, 3H), 2.45–2.40 (m, 1H), 2.38 (t, *J* = 6.8 Hz, 2H), 2.08 (td, *J* = 11.6, 3.6 Hz, 1H), 1.91 (s, 3H), 1.62 (t, *J* = 11.2 Hz, 1H). ^13^C­{^1^H} NMR (100.4 MHz, CD_3_OD) δ (ppm): 145.2, 143.9, 139.2, 135.6, 135.4, 131.9, 130.8 (2C), 128.5 (2C), 62.3, 54.4, 52.3, 49.6, 31.2, 26.5, 21.5, 14.2. MS (ESI) *m/z* (%): 689 ([2M + Na]^+^, 94), 356 ([M + Na]^+^, 100). HRMS (ESI Orbitrap) *m/z*: [M + H]^+^ calcd for C_18_H_24_NO_3_S: 334.1477. Found: 334.1472.

##### (*S*)-2-(3-Methyl-4,5-dihydro-3a*H*-cyclopenta­[a]­naphthalen-1-yl)-ethanol (**15j**)

Prepared following the General procedure and Work-up B, starting from (*S*)-**14j** (45 mg, 0.20 mmol, ee, 99%) and obtaining crude (*S*)-**15j** (35 mg, 77%), as a colorless oil, that was fully characterized without further purification to avoid isomerization of the cyclopentadienyl moiety. ESI-MS and HRMS analyses were performed on a purified sample (eluent: EtOAc/*n*-hexane, 1:5; *R*_f_ = 0.17). ee, 95%. [α]_D_^20^ +31.5 (*c* 1.0, CH_3_OH). ^1^H NMR (400 MHz, CDCl_3_) δ (ppm): 7.61 (d, *J* = 7.2 Hz, 1H), 7.21–7.09 (m, 3H), 6.16 (s, 1H), 3.96–3.89 (m, 2H), 3.09–2.88 (m, 3H), 2.82–2.75 (m, 1H), 2.42–2.36 (m, 1H), 2.05 (s, 3H), 1.65 (br s, 1H), 1.31–1.16 (m, 2H). ^13^C­{^1^H} NMR (100.4 MHz, CDCl_3_) δ (ppm): 148.0, 140.6, 136.3, 134.9, 133.3, 131.0, 128.8, 125.9, 125.7, 125.1, 62.1, 54.8, 32.1, 30.7, 28.5, 14.1. GC-MS (EI) *m/z* (%): 226 ([M]^+^, 28), 195 (100), 182 (33), 165 (42). HRMS (ESI Orbitrap) *m/z*: [M + H]^+^ calcd for C_16_H_19_O: 227.1436. Found: 227.1432.

##### (*S*)-2-(3-Pentyl-4,5-dihydro-3a*H*-cyclopenta­[a]­naphthalen-1-yl)-ethanol (**15k**)

Prepared following the General procedure and Work-up B, starting from (*S*)-**14k** (82 mg, 0.30 mmol, ee, 97%) and obtaining crude (*S*)-**15k** (78 mg, 92%), as a thick colorless oil, that was fully characterized without further purification to avoid isomerization of the cyclopentadienyl moiety. ESI-MS and HRMS analyses were performed on a purified sample (eluent: EtOAc/*n*-hexane, 1:3; *R*_f_ = 0.44). ee, 94%. [α]_D_^21^ +10.6 (*c* 1.0, CH_3_OH). ^1^H NMR (400 MHz, CD_3_OD) δ (ppm): 7.51 (d, *J* = 7.2 Hz, 1H), 7.14–7.11 (m, 2H), 7.05–7.01 (m, 1H), 6.14–6.12 (m, 1H), 3.79–3.75 (m, 2H), 2.97–2.82 (m, 3H), 2.78–2.71 (m, 1H), 2.37–2.32 (m, 3H), 1.63–1.48 (m, 2H), 1.37–1.33 (m, 4H), 1.23–1.09 (m, 2H), 0.92 (t, *J* = 7.2 Hz, 3H). ^13^C­{^1^H} NMR (100.4 MHz, CD_3_OD) δ (ppm): 153.4, 140.9, 137.5, 136.1, 134.6, 131.4, 129.8, 126.8, 126.6, 126.1, 62.3, 54.9, 33.4, 33.0, 31.8, 30.0, 29.8, 29.6, 23.6, 14.4. GC-MS (EI) *m/z* (%): 282 ([M]^+^, 40), 251 (100), 226 (18), 182 (59), 165 (37). HRMS (ESI Orbitrap) *m/z*: [M + H]^+^ calcd for C_20_H_27_O: 283.2062. Found: 283.2057.

##### (*S*)-2-(3-Pentyl-3a,4,5,6-tetrahydropentalen-1-yl)­ethanol (**15l**)

Prepared following the General procedure and Work-up B, starting from (*S*)-**14l** (95 mg, 0.44 mmol, ee, 97%) and obtaining (*S*)-**15l** (10 mg, 10%) after purification by flash chromatography (eluent: EtOAc/*n*-hexane, 1:10; *R*_f_ = 0.10), as a colorless oil that contained a 2:1 mixture of regioisomers. ee, 92%. ^1^H NMR (400 MHz, CD_3_OD) (2:1 mixture of regioisomers) δ (ppm): 5.98 (s, 1H, minor), 5.91 (s, 1H, major), 3.66 (t, *J* = 7.2 Hz, 2H, minor), 3.62 (t, *J* = 7.2 Hz, 2H, major), 3.13–3.04 (m, 1H), 2.55–2.47 (m, 1H), 2.42–2.36 (m, 2H), 2.33–2.22 (m, 4H), 2.20–2.09 (m, 3H), 2.04–1.91 (m, 2H), 1.53–1.45 (m, 3H), 1.39–1.25 (m, 9H), 0.93–0.88 (m, 3H), 0.85–0.74 (m, 1H). ^13^C­{^1^H} NMR (100.4 MHz, CD_3_OD) (mixture of regioisomers) δ (ppm): 153.0, 151.9, 151.4, 147.1, 136.3, 133.5, 132.7, 131.8, 62.9, 62.6, 62.0, 61.9, 34.8, 33.0, 32.9, 32.8, 32.55, 32.52, 31.5, 30.4, 29.9, 29.0, 28.83, 28.78, 23.63, 23.62, 22.74, 22.71, 14.5, 14.4. GC-MS (EI) *m/z* (%): 220 ([M]^+^, 25), 189 (46), 164 (35), 133 (47), 91 (100). HRMS (ESI Orbitrap) *m/z*: [M + Li]^+^ calcd for C_15_H_24_LiO: 227.1987. Found: 227.1981.

##### (*S*)-(+)-2-[7a-(*N*,*N*’-Diethoxycarbonyl)­hydrazino-3-methyl-5,6,7,7a-tetrahydro-4*H*-Inden-1-yl]-ethanol (**19**)

Compound (*S*)-**19** was prepared according to the procedure reported in the literature.^12^b Starting from (*S*)-**14a** (301 mg, 1.71 mmol, ee, 99%) and using IPrAuNTf_2_ (1 mol %) as the catalyst and DEAD (269 μL, 1 equiv) as the dienophile, crude (*S*)-**19** was obtained after reduction with NaBH_4_. The crude product was dissolved in DCM (34 mL) and treated with a catalytic amount of PTSA·H_2_O. After 5 min, saturated NaHCO_3_ was added and the mixture was vigorously stirred for 5 min. The phases were separated, the aqueous phase was extracted once with DCM (17 mL), and the combined organic extracts were first washed with brine (34 mL) and then dried over Na_2_SO_4_. Pure (*S*)-**19** was obtained as a white foam (488 mg, 1.38 mmol, 81%) after flash chromatography purification (EtOAc/*n*-hexane, 1:1; *R*_f_ = 0.22). To determine the enantiomeric excess of (*S*)-**19**, a small amount of the solution containing intermediate diene (*S*)-**15a** generated by the gold­(I) catalysis was collected before the addition of DEAD and treated with an excess of *N*-phenylmaleimide, followed by NaBH_4_ reduction and purification to obtain a sample of pure cycloadduct **18a** for chiral HPLC analysis. ee, 97%. [α]_D_^20^ +104.6 (*c* 0.9, CHCl_3_). ^1^H NMR (400 MHz, CD_3_OD) (1.4:1 mixture of rotamers) δ (ppm): 5.89 (m, 1H, minor), 5.81 (m, 1H, major), 4.18–4.03 (m, 4H), 3.79–3.71 (m, 2H), 3.19–3.01 (m, 1H), 2.62–2.43 (m, 3H), 2.14–2.05 (m, 1H), 1.93–1.84 (m, 1H), 1.76 (s, 3H), 1.74–1.67 (m, 1H), 1.59–1.44 (m, 2H), 1.26–1.17 (m, 6H), 1.13–1.02 (m, 1H), 0.88–0.76 (m, 1H). Spectroscopical data were identical to those reported for the corresponding racemic compound.^12^b

##### (*S*)-(+)-Triisopropyl-{2-[7a-(*N*,*N*’-diethoxycarbonyl)­hydrazino-3-methyl-5,6,7,7a-tetrahydro-4*H*-inden-1-yl]-ethoxy}-silane (**20**)

Compound (*S*)-**20** was prepared according to the procedure reported in the literature.^12^b Starting from (*S*)-**19** (330 mg, 0.94 mmol), pure (*S*)-**20** (364 mg, 76%) was obtained as a thick colorless oil after chromatographic purification (eluent: *n*-hexane/EtOAc, 8:1; *R*_f_ = 0.22). [α]_D_^22^ +76.2 (*c* 1.0, CHCl_3_). ^1^H NMR (400 MHz, CDCl_3_) (1.5:1 mixture of rotamers) δ (ppm): 6.65 (br s, 1H, NH major), 6.44 (br s, 1H, NH minor), 5.89 (s, 1H, minor), 5.79 (s, 1H, major), 4.21–3.99 (m, 4H), 3.88–3.78 (m, 2H), 3.25 (br m, 1H, major), 2.98 (br m, 1H, minor), 2.61–2.38 (m, 3H), 2.34–2.24 (m, 1H, major), 2.05–1.79 (m, 2H), 1.75 (s, 3H, minor), 1.73 (s, 3H, major), 1.65–1.39 (m, 2H), 1.27–1.13 (m, 6H), 1.11–1.00 (m, 22H), 0.91–0.73 (m, 1H and 1H, minor). Spectroscopical data were identical to those reported for the corresponding racemic compound.^12^b

##### (*S*)-(+)-{*N*,*N*’-diethoxycarbonyl-*N*’-[1-methyl-3-(2-triisopropylsilanyloxyethyl)-4,5,6,7-tetrahydroinden-3a-yl]-hydrazino}-acetic acid ethyl ester (**21**)

Compound (*S*)-**21** was prepared according to the procedure reported in the literature.^12^b Starting from (*S*)-**20** (330 mg, 0.65 mmol), pure (*S*)-**21** (337 mg, 87%) was obtained as a colorless oil after chromatographic purification (eluent: *n*-hexane/EtOAc, 8:1; *R*_f_ = 0.30). [α]_D_^20^ +44.3 (*c* 1.0, CHCl_3_). ^1^H NMR (400 MHz, CDCl_3_) (2:1 mixture of rotamers) δ (ppm): 5.82 (m, 1H, major), 5.80 (m, 1H, minor), 4.26–3.99 (m, 7H), 3.92–3.83 (m, 2H), 3.75 (d, *J* = 16.8 Hz, 1H, minor), 3.59–3.49 (m, 1H, major), 3.40 (d, *J* = 16.4 Hz, 1H, major), 2.91–2.83 (m, 1H, minor), 2.65–2.40 (m, 3H), 2.39–2.22 (m, 1H), 2.20–2.08 (m, 1H, major), 1.96–1.81 (m, 1H and 1H, minor), 1.81–1.67 (m, 1H, major), 1.70 (s, 3H), 1.60–1.51 (m, 1H), 1.29–1.18 (m, 6H), 1.08–1.03 (m, 22H), 0.90–0.84 (m, 1H, minor), 0.81–0.71 (m, 1H, major). Spectroscopical data were identical to those reported for the corresponding racemic compound.^12^b

##### (*S*)-(+)-[1-Methyl-3-(2-triisopropylsilanyloxyethyl)-4,5,6,7-tetrahydroinden-3a-yl]-carbamic acid ethyl ester (**22**)

Compound (*S*)-**22** was prepared according to the procedure reported in the literature.^12^b Starting from (*S*)-**21** (99 mg, 0.17 mmol), pure (*S*)-**22** (52 mg, 74%) was obtained as a colorless oil after chromatographic purification (eluent: *n*-hexane/EtOAc, 10:1; *R*_f_ = 0.34). [α]_D_^22^ +56.5 (*c* 0.92, CHCl_3_). ^1^H NMR (400 MHz, CDCl_3_) δ (ppm): 5.86 (s, 1H), 4.65 (s, 1H), 4.02–3.94 (m, 2H), 3.90–3.80 (m, 2H), 2.60–2.55 (m, 1H), 2.48–2.39 (m, 2H), 2.28–2.21 (m, 1H), 1.99–1.83 (m, 2H), 1.80 (s, 3H), 1.61–1.42 (m, 2H), 1.16 (t, *J* = 7.2 Hz, 3H), 1.13–1.01 (m, 22H), 0.89 (td, *J* = 13.2, 4.4 Hz, 1H). Spectroscopical data were identical to those reported for the corresponding racemic compound.^12^b

## Supplementary Material



## Data Availability

The data underlying this study are available in the published article and its Supporting Information.

## Notes

Additional experimental procedures, ^1^H and ^13^C NMR spectra, GLC and HPLC analyses, X-ray crystallography details, and computational methods (PDF)
