# Supplementary material for: Central-to-Axial-to-Central Chirality Transfer in the Au(I)-Catalyzed Cycloisomerization of Propargyl Vinyl Ethers to Cyclopentadienes
Source: J Org Chem. 2025 May 13;90(20):6743–54. doi: 10.1021/acs.joc.5c00433 (PMC12117571; doi:10.1021/acs.joc.5c00433)
Supplement: Supplementary file 1 [file jo5c00433_si_001.pdf]

Central-to-Axial-to-Central Chirality Transfer in the Au(I)-Catalyzed Cycloisomerization of Propargyl Vinyl Ethers to Cyclopentadienes

Dina Scarpi,[a] Giovanni Turchi,[a] Matteo Fazzini,[a] Lucilla Favero,[b] and Ernesto G. Occhiato[a,*]

[a]*Dipartimento di Chimica "U. Schiff", Università degli Studi di Firenze, Via della Lastruccia 13, 50019, Sesto Fiorentino (FI), Italy. E-mail: ernesto.occhiato@unifi.it*

[b]*Dipartimento di Farmacia, Università degli Studi di Pisa, Via Bonanno 33, 56126, Pisa, Italy*

Supporting Information

Table of contents

1. Experimental procedures S2

1.1. EKR of commercially available racemic alcohols **12b-c** S2-S4

1.2. EKR of alcohols **13c-d** S4-S5

1.3. GLC and HPLC chromatograms of compounds deriving from the EKRs S6-S11

2. Determination of the enantiomeric excess of dienes **15a-l** S12

2.1. Hydrogenation of compound (*S*)-**15a** and chiral GLC analysis S12

2.2. Reaction of dienes **15a-l** with *N*-phenylmaleimide: synthesis of compounds **18a-l** S13-S18

2.3. HPLC chromatograms of racemic and chiral compounds **18a-l** S19-S31

3. Determination of the absolute configuration of diene **15a** S32

3.1. Synthesis of compound **16** S32

3.2. Crystal structure determination of compound **16** S33-S34

3.3. ORTEP drawing of compound **16** S35

4. Copies of ^{1}H and ^{13}C NMR spectra S36-S106

5. Supplementary material for DFT calculation S107

5.1. Cartesian coordinates of the optimized structures S110-S123

1. Experimental procedures

General information. Anhydrous solvents were prepared according to the standard techniques. Commercially available reagents were used without further purification. Melting points were recorded on a Büchi B-540 apparatus and are uncorrected. Chromatographic separations were performed under pressure on silica gel (Merck 70-230 mesh) by using flash column techniques; R_f values refer to TLC carried out on 0.25 mm silica gel plates (F_{254}) with the same eluent as indicated for column chromatography. ^{1}H NMR (200 or 400 MHz) and ^{13}C NMR (100.4 MHz) spectra were recorded either on Varian Inova (400 MHz) or Mercury (200 or 400 MHz) spectrometers in the specified deuterated solvent at 25 °C. Solvent reference lines were set at 7.26 ($CDCl_3$) and 3.31 (CD_3OD) in ^{1}H NMR spectra, and at 77.00 ($CDCl_3$), 49.00 (CD_3OD) and 206.26 (acetone-d6) in the ^{13}C NMR spectra, respectively. Mass spectra were recorded either by direct inlet of a 20 ppm solution in CH_3OH on a LCQ Fleet™ Ion Trap LC/MS system (Thermo Scientific) with electrospray ionization (ESI) interface in the positive ion mode or by electron ionization (EI) at 70 eV on a Shimadzu GC/MS-QP2020NX instrument equipped with a SH-Rxi-5ms Shimadzu column. Microanalyses were carried out with a ThermoScientific FlashSmart Elemental Analyzer CHNS/O. HRMS analyses were performed under conditions of ESI-MS through direct infusion of a 10 ppm solution in 90/10 MeOH/H_2O containing 0.1% formic acid in a LTQ Orbitrap mass spectrometer (Thermo Scientific). HPLC analyses were carried out with a Dionex Ulltimate 3000 HPLC system equipped either with a Lux 5μm Amylose-1 or with a Lux 5μm Cellulose-4 column, 250 x 4.60 mm and eluting at 0.5 mL/min flow rate with the reported eluent in isocratic conditions. GLC analyses were carried out on a Shimadzu GC2014 instrument equipped with a Supelco β DEX™ 120, 30 m × 0.25 mm, 0.25 μm film column.

1.1. EKR of commercially available racemic alcohols 12b-c.

a) EKR of (±)-1-phenyl-2-propyn-1-ol (12b).

Scheme S1.

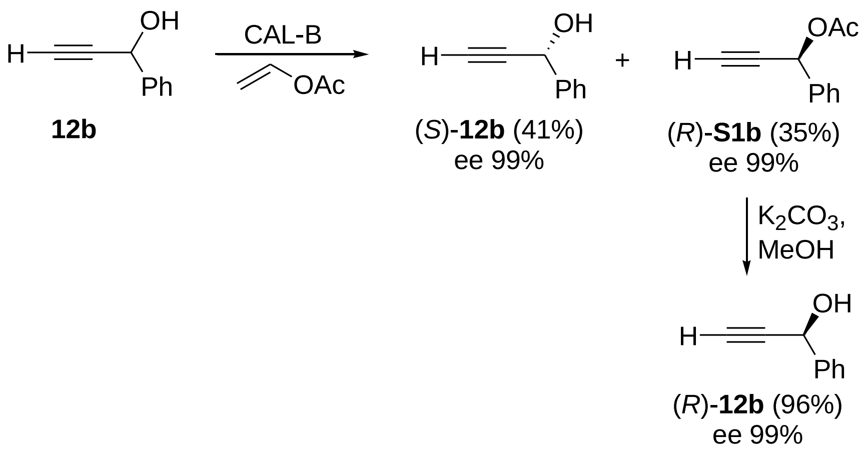

To a solution of alcohol (±)-**12b** (496 μL, 4.1 mmol) in anhydrous toluene (10 mL, 0.4 M) was added CAL-B (Novozyme435, 142 mg, 35 mg/mmol substrate) followed by vinyl acetate (750 μL, 8.2 mmol, 2 equiv.). The mixture was left under slow magnetic stirring in a nitrogen atmosphere at 30 °C and the reaction monitored

by GLC. After 30 minutes the conversion was nearly 50% and the reaction was stopped by filtration over a celite pad. The filtrate was evaporated and the crude purified by flash chromatography (EtOAc/*n*-hexane, 1:4 + 1% Et_3N) to afford alcohol (*S*)-**12b** (218 mg, 41%) and acetate (*R*)-**S1b** (249 mg, 35%) as clear oils. The absolute configuration was determined by comparison of the optical rotation sign with the data reported in the literature.

(*S*)-**12b**: e.e. 99% (by GLC: R_t 12.27 min).

(*R*)-**S1b**: e.e. 99% (by GLC: R_t 11.39 min).

GLC conditions. Racemate (±)-**12b**: 142 °C for 3 min to 146 °C for 2 min at 1 °C/min, then to 160°C for 4 min at 10 °C/min.; 11.94 min (*S*-enantiomer) and 12.25 min (*R*-enantiomer). Racemate (±)-**S1b**: R_t 11.23 min (*R*) and 11.38 min (*S*).

Hydrolysis of (*R*)-S1b. A solution of acetate (*R*)-**S1b** (249 mg, 1.43 mmol) in anhydrous CH_3OH (4.8 mL, 0.3 M) was cooled at 0°C (ice bath) and K_2CO_3 (198 mg, 1.43 mmol) was added in one portion. The ice bath was then removed, and the mixture allowed to stir at room temperature for 30 minutes. Aqueous 1 N HCl (2.8 mL) was added, methanol removed under *vacuum* and the residue suspended in water (5 mL). The product was extracted with EtOAc (3 x 5 mL) and the combined organic extracts were washed with brine and dried over Na_2SO_4. After filtration and evaporation of the solvent, the crude (*R*)-**12b** (182 mg, 96%) was used as such in the Sonogashira coupling (see Experimental Section).

b) EKR of (±)-1-heptyn-3-ol (12c).

Scheme S2.

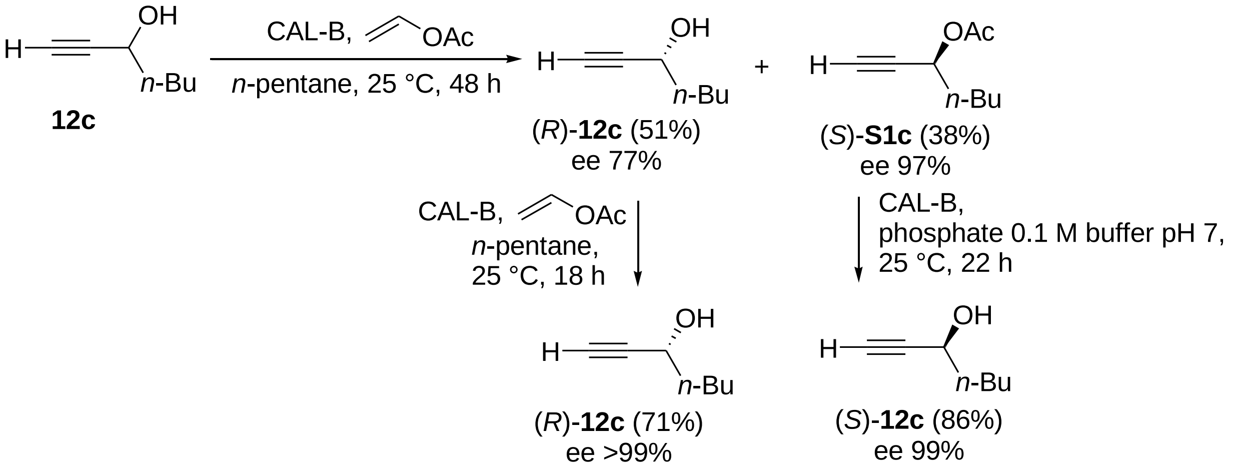

Prepared as reported for **12b**, starting from alcohol (±)-**12c** (528 μL, 4.0 mmol) in anhydrous *n*-pentane (0.5 M) with CAL-B (Novozyme435, 40 mg, 10 mg / mmol substrate) at 25 °C. After 48 h the conversion was 43% and the reaction was stopped by filtration over a celite pad, washing thoroughly the pad with Et_2O (20 mL). The filtrate was evaporated (P ≥ 300 mbar) and the crude purified by flash chromatography, eluting first with Et_2O/*n*-pentane, 1:10 to recover the acetate (*S*)-**S1c** (237 mg, 38%) and then with Et_2O/*n*-pentane, 1:5 to afford alcohol (*R*)-**12c** (227 mg, 51%) as clear oils. The absolute configuration was determined by comparison of the optical rotation sign with the data reported in the literature.

(R)-**12c**: e.e. 77% (by GLC: R_t 8.04 min).

(S)-**S1c**: e.e. 97% (by GLC: R_t 8.51 min).

GLC conditions. Oven temp.: 110 °C for 3 min to 150 °C for 3 min at 4°C/min. Racemate (±)-**12c**: 7.93 min (S-enantiomer) and 8.04 min (R-enantiomer). Racemate (±)-**S1c**: 8.33 min (R-enantiomer) and 8.51 min (S-enantiomer).

Enantioenriched alcohol (R)-**12c** (216 mg, 1.93 mmol, e.e. 77%) was used as substrate for a second EKR, set up under the conditions reported above. The reaction was monitored by GLC and after 18 h no residual (S)-**12c** was detected. The reaction was stopped and the product (R)-**12c** recovered as a clear oil after chromatography (153 mg, 71%, ee 99%).

Enzymatic hydrolysis of (S)-S1c. The acetate (S)-**S1c** (237 mg, 1.54 mmol, e.e. 97%) was suspended in 0.1 M phosphate buffer at pH 7.0 (5.1 mL, 0.3 M) and CAL-B (Novozyme435, 25 mg, 16 mg/mmol substrate) was added in one portion and the mixture was vigorously stirred at room temperature for 22 h (GLC monitoring). The reaction was then stopped by filtration over a thin celite pad, washing thoroughly the pad with Et_2O (25 mL). The aqueous filtrate was extracted with Et_2O (3 x 5 mL) and the combined organic extracts were dried over Na_2SO_4. After filtration and evaporation of the solvent, the crude was purified by flash chromatography and pure alcohol (S)-**12c** was obtained as a clear oil (149 mg, 86%, e.e. 99%).

1.2. EKR of racemic alcohols (±)-13c and (±)-13d.

a) 1-Cyclohex-1-enylhept-1-yn-3-ol (13c).

Scheme S3.

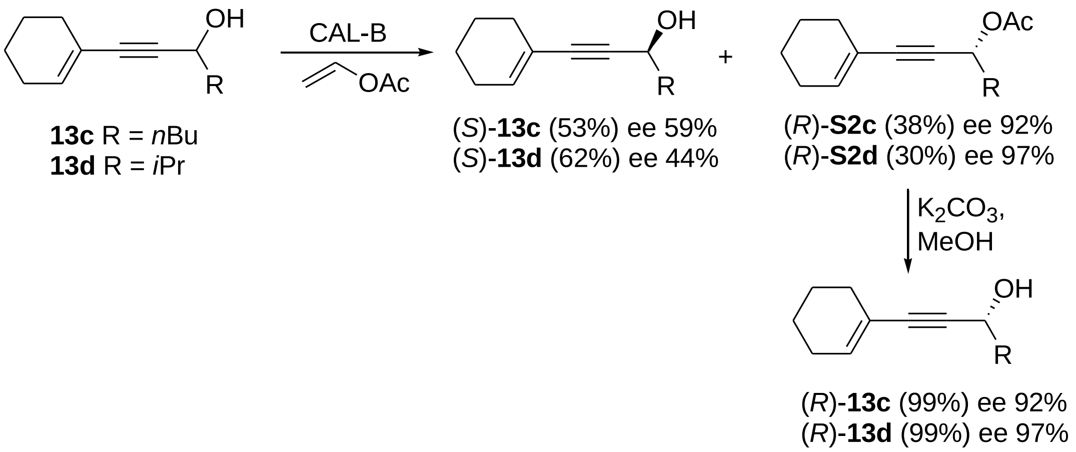

Prepared as reported for **12b**, starting from racemic substrate **13c** (162 mg, 0.84 mmol) in anhydrous n-hexane (0.4 M) with CAL-B (Novozyme435, 93 mg, 110 mg/mmol substrate). The reaction was stopped when the conversion reached 47% (25 minutes) by filtration over a celite pad. The filtrate was evaporated and the crude purified by flash chromatography (EtOAc/n-hexane, 1:4 + 1% Et_3N) to afford alcohol (S)-**13c** (85 mg, 52%) and acetate (R)-**S2c** (71 mg, 38%) as clear oils. The absolute configuration was determined by comparison of the optical rotation sign with the data reported in the literature. For enantiomeric excess determination, only the acetates could be resolved by chiral HPLC.

(R)-**S2c**: e.e. 92% (by HPLC: R_t 8.82 min).

HPLC conditions. Column: Lux 5μm Amylose-1. Eluent: *n*-hexane/IPA, 99.5:0.5 (isocratic conditions). Racemate (±)-**S2c**: R_t 8.27 min (*S*) and 8.67 min (*R*).

The hydrolysis of the so obtained acetate was performed as reported for (*R*)-**S1b**, starting from (*R*)-**S2c** (67 mg, 0.29 mmol) and obtaining crude (*R*)**-13c** (55 mg, quantitative) that was used in the next step without further purification.

b) 1-Cyclohex-1-enyl-4-methyl-pent-1-yn-3-ol (13d).

Prepared as reported for **12b**, starting from racemic substrate **13d** (330 mg, 1.85 mmol) in anhydrous *n*-hexane (0.4 M) with CAL-B (Novozyme435, 120 mg, 70 mg/mmol substrate). The reaction was stopped when the conversion reached 49% (25 hours) by filtration over a celite pad. The filtrate was evaporated and the crude purified by flash chromatography (EtOAc/*n*-hexane, 1:5 + 1% Et_3N) to afford alcohol (*S*)**-13d** (155 mg, 47%) and acetate (*R*)-**S2d** (175 mg, 43%) as clear oils. The absolute configuration was determined by comparison of the optical rotation sign with the data reported in the literature.

(*R*)-**S2d**: e.e. 97% (by HPLC: R_t 11.11 min).

HPLC conditions. Column: Lux 5μm Amylose-1. Eluent: *n*-hexane/IPA, 99.5:0.5 (isocratic conditions). Racemate (±)-**S2c**: R_t 9.53 min (*S*) and 11.02 min (*R*).

The hydrolysis of the so obtained acetate was performed as reported for (*R*)**-S1b**, starting from (*R*)**-S2d** (107 mg, 0.49 mmol) and obtaining crude (*R*)**-13c** (87 mg, quantitative) that was used in the next step without further purification.

1.3. GCL and HPLC analyses of racemic and chiral compounds deriving from EKRs

Analysis Date & Time : 05/03/2024 16.45.38
User Name : Admin
Vial# : 3
Sample Name : alcool racemo
Sample ID : fenilpropinolo
Sample Type : Unknown
Injection Volume : 0,50
ISTD Amount :

Data Name : C:\Documents and Settings\user\Desktop\Dina\Acetati Sonogashira\fenilpropinolo_rac_chi
Method Name : C:\Documents and Settings\user\Desktop\Dina\Acetati Sonogashira\Fenilpropanolo_chir_2.
[Description]
alcool racemo con metodo usato per i puri

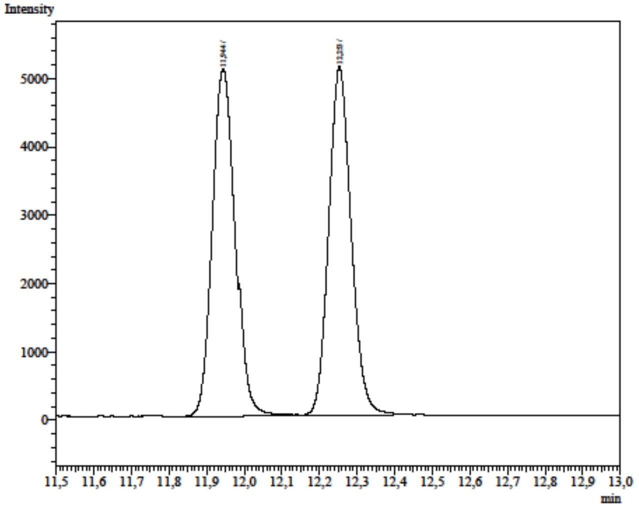

Peak#	Ret.Time	Area	Area%	Height	ID#	Cmpd Name
1	11,944	21503	50,4073	5085		
2	12,253	21156	49,5927	5105		
**Total**		42659	100,0000	10190		

Analysis Date & Time : 05/03/2024 16.12.23
User Name : Admin
Vial# : 5
Sample Name : alcool
Sample ID : MF22 alcool da EKR
Sample Type : Unknown
Injection Volume : 0,50
ISTD Amount :

Data Name : C:\Documents and Settings\user\Desktop\Dina\Acetati Sonogashira\MF22_EKR_alcool.gc
Method Name : C:\Documents and Settings\user\Desktop\Dina\Acetati Sonogashira\Fenilpropanolo_chir_2.
[Description]
alcool da EKR con Novozyme435 dopo colonna (5 mM in MeOH)

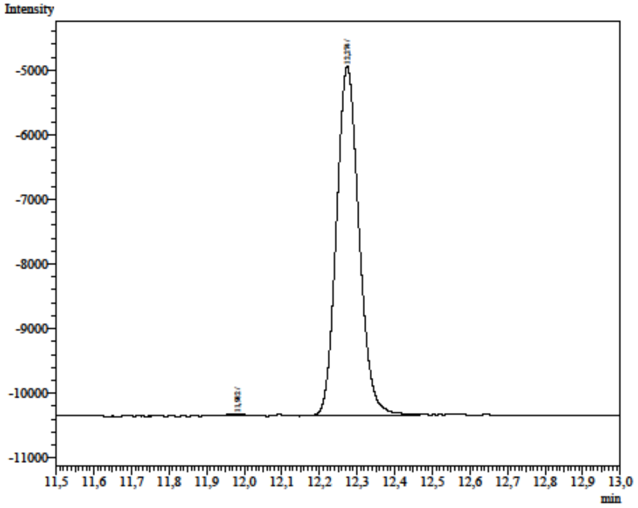

Peak#	Ret.Time	Area	Area%	Height	ID#	Cmpd Name
1	11,982	141	0,6240	31		
2	12,274	22433	99,3760	5380		
**Total**		22574	100,0000	5411		

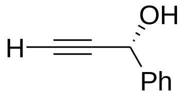

(*S*)-**12b**

GLC chromatograms of alcohols (±)-**12b** (left) and (*S*)-**12b** (right)

Analysis Date & Time : 05/03/2024 17.02.15
User Name : Admin
Vial# : 4
Sample Name : acetato racemo
Sample ID : MF21 acetato racemo
Sample Type : Unknown
Injection Volume : 0,50
ISTD Amount :

Data Name : C:\Documents and Settings\user\Desktop\Dina\Acetati Sonogashira\MF21_acetato_rac_chi
Method Name : C:\Documents and Settings\user\Desktop\Dina\Acetati Sonogashira\Fenilpropanolo_chir_2.
[Description]
acetato racemo MF21 con metodo usato per i puri

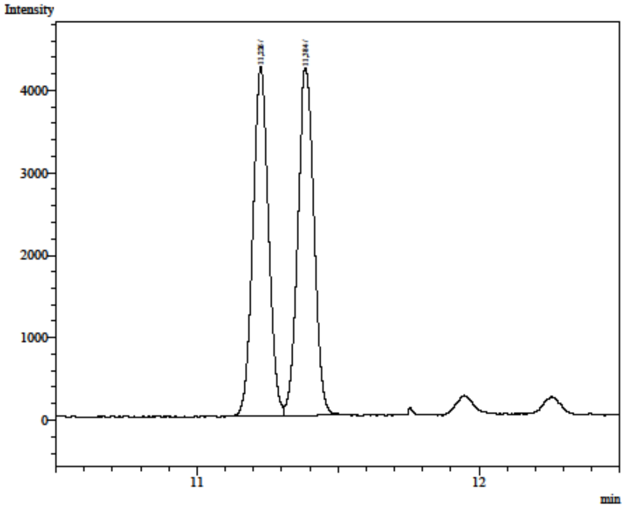

Peak#	Ret.Time	Area	Area%	Height	ID#	Cmpd Name
1	11,226	16208	49,2048	4228		
2	11,384	16732	50,7952	4212		
Total		32940	100,0000	8440		

Analysis Date & Time : 05/03/2024 16.29.01
User Name : Admin
Vial# : 6
Sample Name : acetato
Sample ID : MF22 acetato da EKR
Sample Type : Unknown
Injection Volume : 0,50
ISTD Amount :

Data Name : C:\Documents and Settings\user\Desktop\Dina\Acetati Sonogashira\MF22_EKR_acetato.gc
Method Name : C:\Documents and Settings\user\Desktop\Dina\Acetati Sonogashira\Fenilpropanolo_chir_2.
[Description]
acetato da EKR con Novozyme435 dopo colonna (5 mM MeOH)

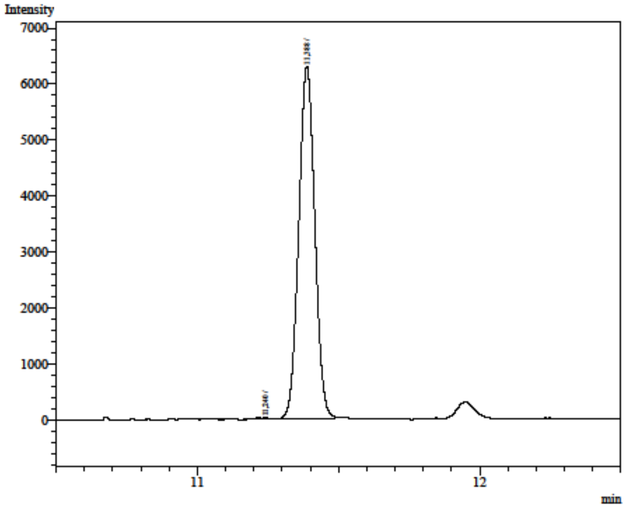

Peak#	Ret.Time	Area	Area%	Height	ID#	Cmpd Name
1	11,240	95	0,3807	26		
2	11,388	24805	99,6193	6278		
Total		24900	100,0000	6304		

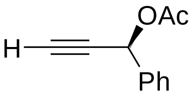

(*R*)-**S1b**

GLC chromatograms of acetates (±)-**S1b** (left) and (*R*)-**S1b** (right)

Analysis Date & Time : 11/04/2024 10.08.25
User Name : Admin
Vial# : 3
Sample Name : eptinolo racemo
Sample ID : eptinolo rac
Sample Type : Unknown
Injection Volume : 1,00
ISTD Amount :

Data Name : C:\Documents and Settings\user\Desktop\Dina\Rautenstrauch\eptinolo_rac_prova2.gcd
Method Name : C:\Documents and Settings\user\Desktop\Dina\Rautenstrauch\chirale_rampa_110_150.gcm
[Description]
prova di separazione enantiomeri eptinolo racemo; iniezione scouting in rampa da 110 a 150°C

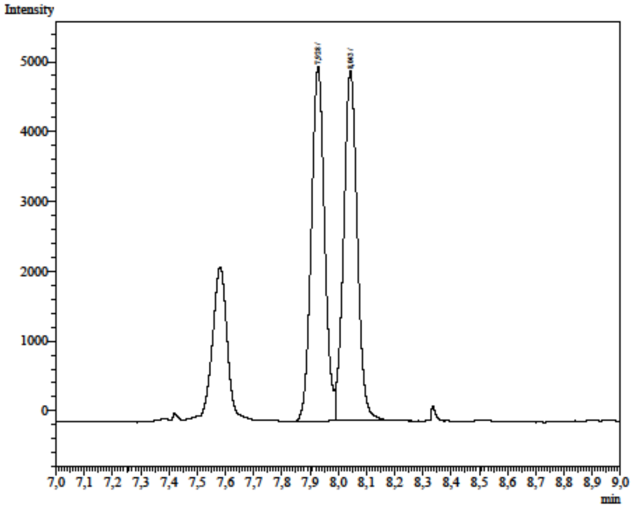

Peak#	Ret.Time	Area	Area%	Height	ID#	Cmpd Name
1	7,928	16690	49,7580	5063		
2	8,043	16852	50,2420	4995		
Total		33542	100,0000	10058		

Analysis Date & Time : 07/05/2024 7.30.41
User Name : Admin
Vial# : 3
Sample Name : EKR 3-eptin-1-olo ee 76.8%
Sample ID : fcc
Sample Type : Unknown
Injection Volume : 1,00
ISTD Amount :

Data Name : C:\Documents and Settings\user\Desktop\Dina\Rautenstrauch\EKR_DSpc1282_OH_fcc.gc
Method Name : C:\Documents and Settings\user\Desktop\Dina\Rautenstrauch\chirale_rampa_110_150.gcm
[Description]
II EKR di alcool DSpc1281 ee 76.8%; alcool isolato dopo cromatografia

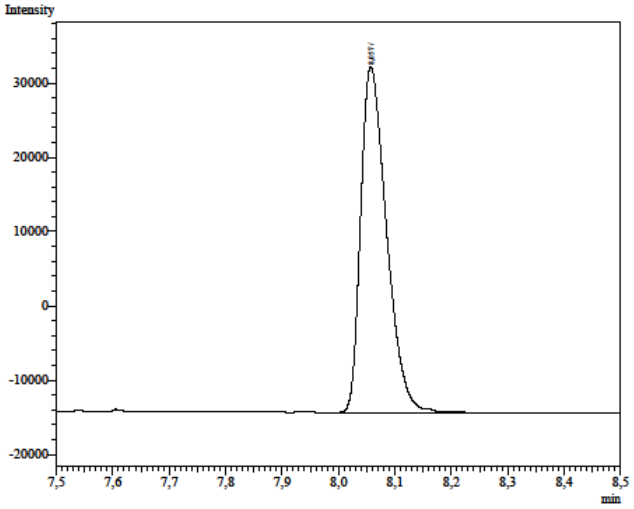

Peak#	Ret.Time	Area	Area%	Height	ID#	Cmpd Name
1	8,057	148309	100,0000	46452		
Total		148309	100,0000	46452		

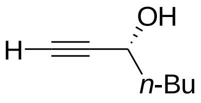

(*R*)-**12c**

GLC chromatograms of alcohols (±)-**12c** (left) and (*R*)-**12c** (right), after the second EKR.

Analysis Date & Time : 11/04/2024 10.08.25
User Name : Admin
Vial# : 3
Sample Name : eptinolo racemo
Sample ID : eptinolo rac
Sample Type : Unknown
Injection Volume : 1,00
ISTD Amount :

Data Name : C:\Documents and Settings\user\Desktop\Dina\Rautenstrauch\eptinolo_rac_prova2.gcd
Method Name : C:\Documents and Settings\user\Desktop\Dina\Rautenstrauch\chirale_rampa_110_150.gcm
[Description]
prova di separazione enantiomeri eptinolo racemo; iniezione scouting in rampa da 110 a 150°C

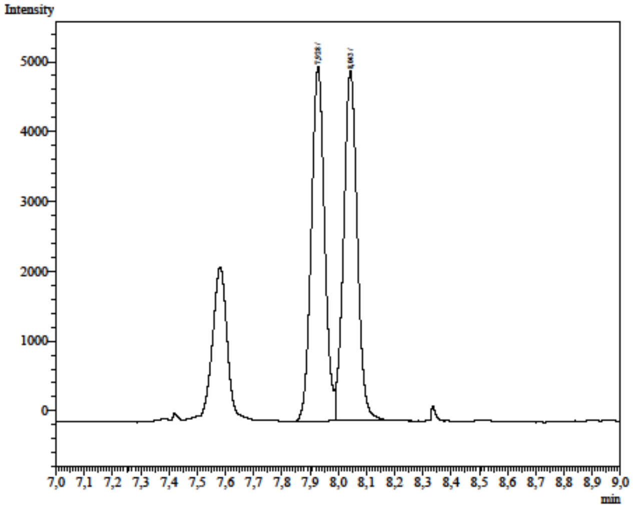

Peak#	Ret.Time	Area	Area%	Height	ID#	Cmpd Name
1	7,928	16690	49,7580	5063		
2	8,043	16852	50,2420	4995		
Total		33542	100,0000	10058		

Analysis Date & Time : 08/05/2024 13.57.06
User Name : Admin
Vial# : 3
Sample Name : EKR 3-eptin-1-olo acetato ee 98.6%
Sample ID : fcc
Sample Type : Unknown
Injection Volume : 1,00
ISTD Amount :

Data Name : C:\Documents and Settings\user\Desktop\Dina\Rautenstrauch\EKR_DSpc1283_OH_fcc.gc
Method Name : C:\Documents and Settings\user\Desktop\Dina\Rautenstrauch\chirale_rampa_110_150.gcm
[Description]
II EKR di acetato DSpc1281 ee 98.6% via idrolisi enzimatica: prodotto isolato via cromatografia

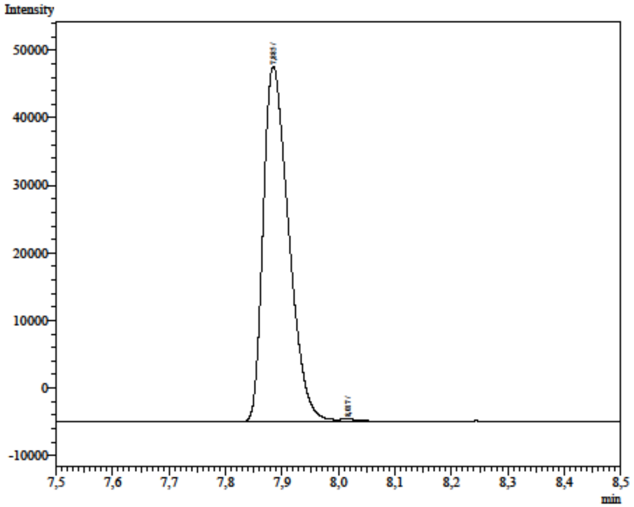

Peak#	Ret.Time	Area	Area%	Height	ID#	Cmpd Name
1	7,885	165203	99,2728	52572		
2	8,017	1210	0,7272	409		
Total		166413	100,0000	52981		

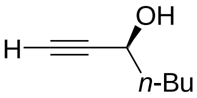

(*S*)-**12c**

GLC chromatograms of alcohols (±)-**12c** (left) and (*S*)-**12c** (right), after enzymatic hydrolysis of (*S*)-**S12c**.

Operator:HPLC Timebase:HPLC2 Sequence:Acetati_Sonogashira_08MAR24 Page 1-1
28/3/2024 3.22 PM

5 DSpc1267 C6nBuOAc rac 5mM MeOH

Sample Name:	DSpc1267 C6nBuOAc rac 5mM MeOH	*Injection Volume:*	2,0
Vial Number:	BC4	*Channel:*	UV_VIS_1
Sample Type:	unknown	*Wavelength:*	223
Control Program:	Iso_mezzoIPA_99Hex_bis	*Bandwidth:*	n.a.
Quantif. Method:	INSTRUMENTS_IQ_Iso17D	*Dilution Factor:*	1,0000
Recording Time:	13/3/2024 16.23	*Sample Weight:*	1,0000
Run Time (min):	30,00	*Sample Amount:*	1,0000

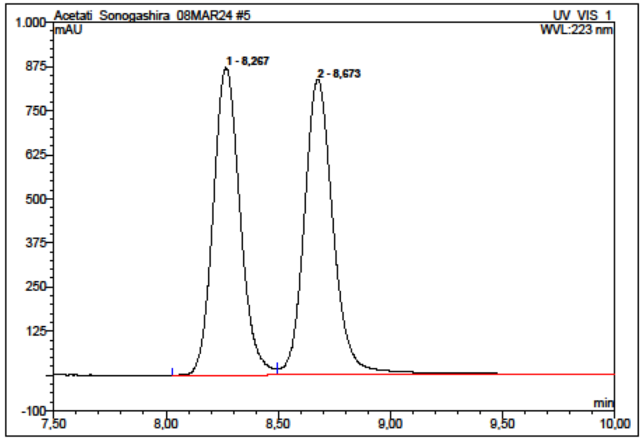

No.	Ret.Time min	Peak Name	Height mAU	Area mAU*min	Rel.Area %	Amount	Type
1	8,27	n.a.	871,859	119,969	48,69	n.a.	BM
2	8,67	n.a.	835,551	126,434	51,31	n.a.	MB
Total:			1707,410	246,403	100,00	0,000	

default/Integration

Chromeleon (c) Dionex 1996-2006
Version 6.80 SR9 Build 2673 (161349)

Operator:HPLC Timebase:HPLC2 Sequence:Acetati_Sonogashira_08MAR24 Page 1-1
14/3/2024 12.01 PM

4 GT37 C6nBuOAc chir 5mM MeOH

Sample Name:	GT37 C6nBuOAc chir 5mM MeOH	*Injection Volume:*	2,0
Vial Number:	BD3	*Channel:*	UV_VIS_1
Sample Type:	unknown	*Wavelength:*	223
Control Program:	Iso_mezzoIPA_99Hex_bis	*Bandwidth:*	n.a.
Quantif. Method:	INSTRUMENTS_IQ_Iso17D	*Dilution Factor:*	1,0000
Recording Time:	13/3/2024 15.53	*Sample Weight:*	1,0000
Run Time (min):	30,00	*Sample Amount:*	1,0000

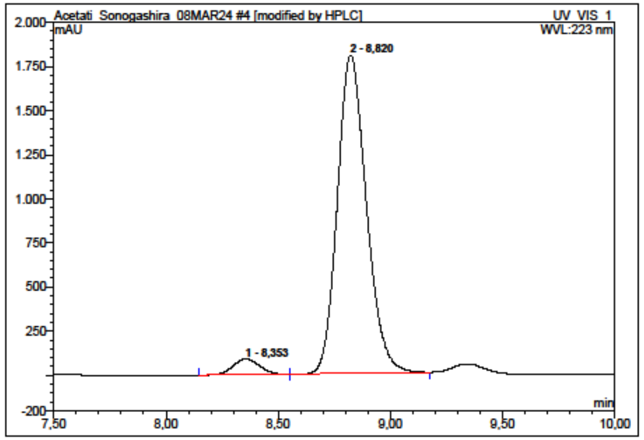

No.	Ret.Time min	Peak Name	Height mAU	Area mAU*min	Rel.Area %	Amount	Type
1	8,35	n.a.	88,124	11,807	4,21	n.a.	BM *
2	8,82	n.a.	1802,414	268,532	95,79	n.a.	MB*
Total:			1890,538	280,340	100,00	0,000	

default/Integration

Chromeleon (c) Dionex 1996-2006
Version 6.80 SR9 Build 2673 (161349)

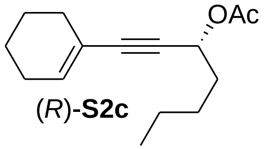

(*R*)-**S2c**

HPLC chromatograms of acetates (±)-**S2c** (left) and (*R*)-**S2c** (right), before hydrolysis.

Operator:HPLC Timebase:HPLC2 Sequence:Acetati_Sonogashira_04APR24 Page 1-1
4/4/2024 1.10 PM

3 NF25 rac C6iPrOAc

Sample Name:	**NF25 rac C6iPrOAc**	*Injection Volume:*	**2,0**
Vial Number:	**BD4**	*Channel:*	**UV_VIS_1**
Sample Type:	**unknown**	*Wavelength:*	**223**
Control Program:	**Iso_mezzoIPA_99Hex_short**	*Bandwidth:*	**n.a.**
Quantif. Method:	**INSTRUMENTS_IQ_Iso17D**	*Dilution Factor:*	**1,0000**
Recording Time:	**4/4/2024 12.49**	*Sample Weight:*	**1,0000**
Run Time (min):	**20,00**	*Sample Amount:*	**1,0000**

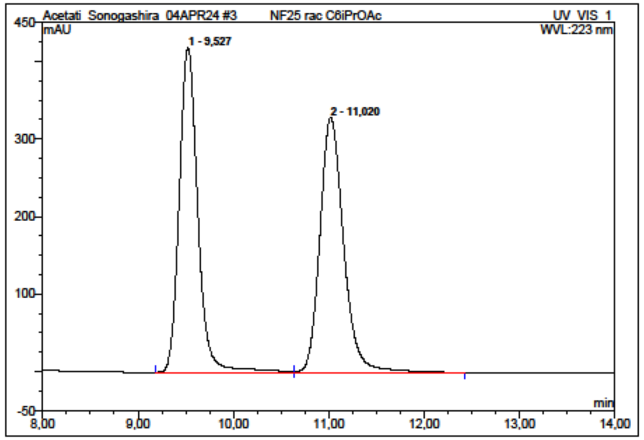

No.	Ret.Time min	Peak Name	Height mAU	Area mAU*min	Rel.Area %	Amount	Type
1	9,53	n.a.	418,075	94,106	49,94	n.a.	BM
2	11,02	n.a.	328,257	94,325	50,06	n.a.	MB
Total:			746,331	188,431	100,00	0,000	

default/Integration

Chromeleon (c) Dionex 1996-2006
Version 6.80 SR9 Build 2673 (161349)

Operator:HPLC Timebase:HPLC2 Sequence:Acetati_Sonogashira_04APR24 Page 1-1
4/4/2024 12.47 PM

1 MF33 C6iPrOAc chir da EKR

Sample Name:	**MF33 C6iPrOAc chir da EKR**	*Injection Volume:*	**2,0**
Vial Number:	**BD1**	*Channel:*	**UV_VIS_1**
Sample Type:	**unknown**	*Wavelength:*	**223**
Control Program:	**Iso_mezzoIPA_99Hex_bis**	*Bandwidth:*	**n.a.**
Quantif. Method:	**INSTRUMENTS_IQ_Iso17D**	*Dilution Factor:*	**1,0000**
Recording Time:	**4/4/2024 11.58**	*Sample Weight:*	**1,0000**
Run Time (min):	**30,00**	*Sample Amount:*	**1,0000**

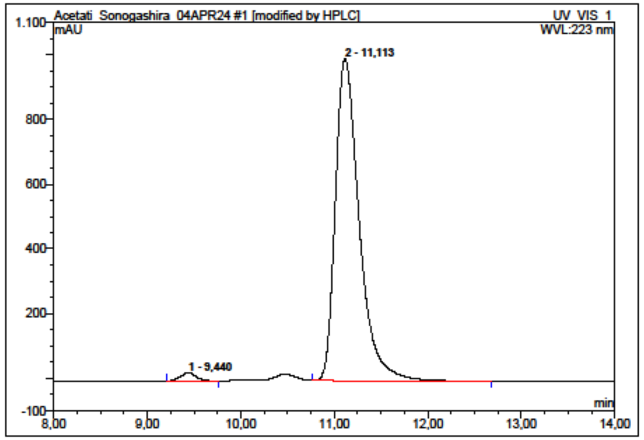

No.	Ret.Time min	Peak Name	Height mAU	Area mAU*min	Rel.Area %	Amount	Type
1	9,44	n.a.	24,774	4,770	1,60	n.a.	BMB*
2	11,11	n.a.	993,894	293,422	98,40	n.a.	BMB
Total:			1018,668	298,193	100,00	0,000	

default/Integration

Chromeleon (c) Dionex 1996-2006
Version 6.80 SR9 Build 2673 (161349)

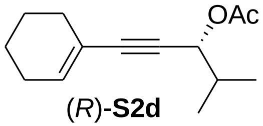

(*R*)-**S2d**

HPLC chromatograms of acetates (±)-**S2d** (left) and (*R*)-**S2d** (right), before hydrolysis.

2. Determination of the enantiomeric excess of dienes 15a-l.

2.1. Hydrogenation of compound (S)-15a and chiral GLC analysis.

For the determination of the enantiomeric excess of **15a**, its hydrogenation over Pd/C was carried out to obtain enantiomers (of the major diastereomer) of **S3** which could be separated by GLC on a β DEX™ 120 column. The same reaction was carried out on racemic **15a** which was prepared to find the conditions for the best separation of the enantiomers.

Scheme S4.

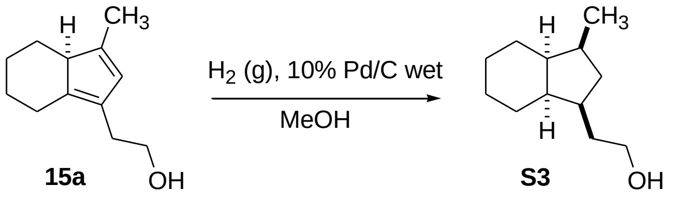

2-(3-Methyloctahydroinden-1-yl)ethanol (S3).

To a solution of (*S*)**-15a** (52 mg, 0.29 mmol) in MeOH (5.8 mL), 10% Pd/C wet (69 mg, 0.029 mmol) was added under a nitrogen atmosphere. The resulting suspension was first flushed with hydrogen under vigorous stirring and then maintained under a hydrogen atmosphere (balloon) at room temperature. After 2 h, the mixture was filtered over a Celite pad, and the residual solution was evaporated under reduced pressure. The oily residue was purified by flash chromatography (eluent: *n*-hexane/EtOAc, 1:4; R_f = 0.37) and compound **S3** (38 mg, 72%; colorless oil) was obtained as a mixture containing a major diastereoisomer (85% by ^{1}H NMR) in mixture with three minor diastereomers, which was analyzed by chiral GLC. ^{1}H NMR (400 MHz, $CDCl_3$) δ (ppm) (major diastereoisomer): 3.68 – 3.56 (m, 2H), 2.18 – 2.10 (m, 1H), 1.93 – 1.57 (m, 4H), 1.53 – 1.18 (m, 10H), 0.91 (t, *J* = 6.4 Hz, 3H), 0.80 – 0.73 (m, 2H).

GLC conditions. Oven temp.: 125 °C for 90 minutes. Racemate **S3**: 61.20 and 62.39 min.

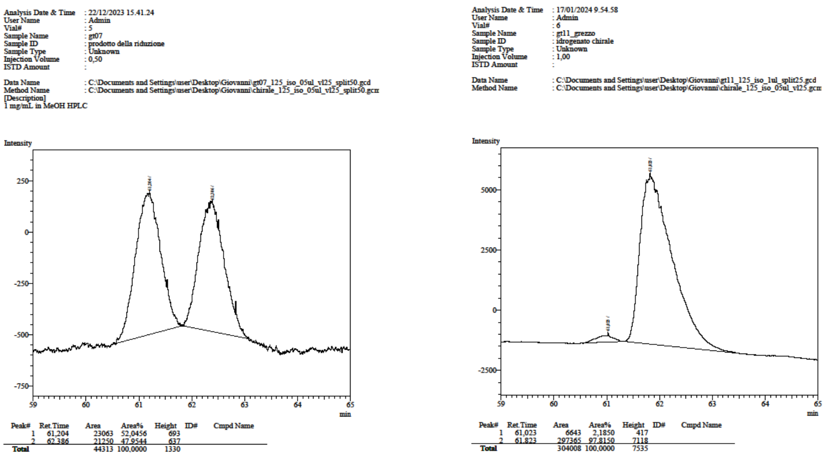

GLC chromatograms of racemic (left) and chiral **S3** (right).

2.2. Reaction of dienes 15a-l with N-phenylmaleimmide: synthesis of compounds 18a-l.

For the determination of the enantiomeric excess of dienes **15a-l**, their cycloadducts with *N*-phenylmaleimide (compounds **18a-l**) were prepared, according to either Method A or B, with generally high facial and *endo*-selectivity. The enantiomers could be separated by HPLC either on a Lux 5µm Amylose-1 or a Lux 5 µm Cellulose-4 column. The same reactions were carried out on racemic **15a-l** which were prepared to find the conditions for the best separation of the enantiomers.

Table S1.

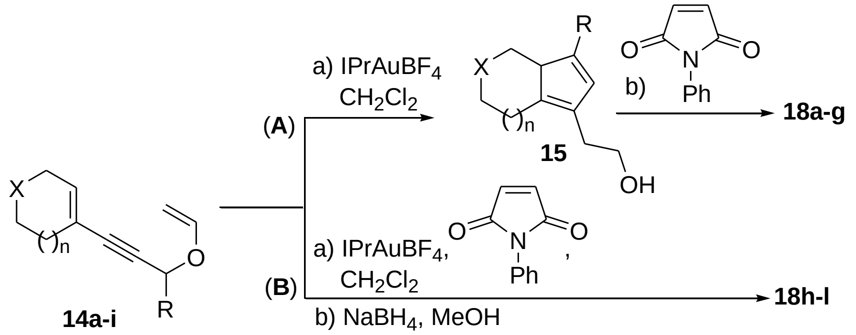

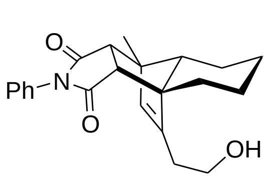

18a (91%)

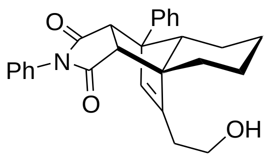

18b (81%)

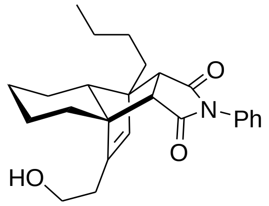

18c (99%)

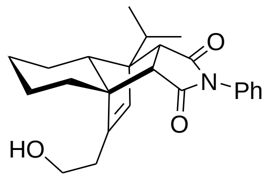

18d (99%)

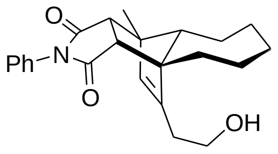

18e (94%)

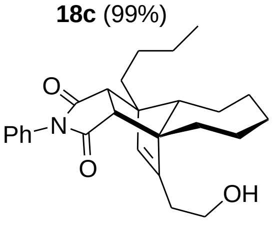

18f (75%)

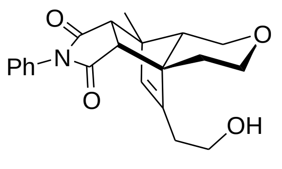

18g (82%)

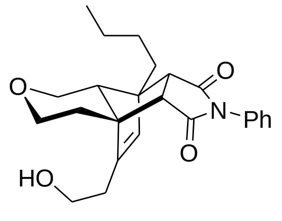

18h (74%)

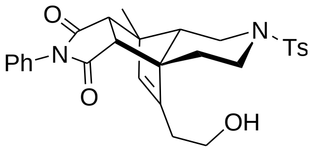

18i (64%)

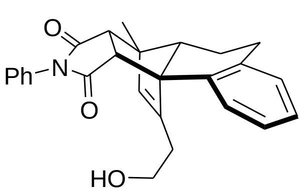

18j (88%)

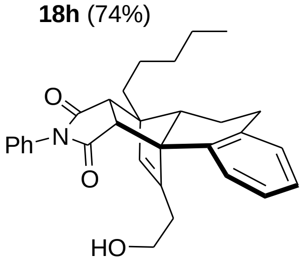

18k (84%)

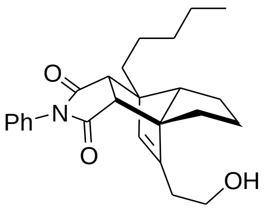

18l (45%)

Procedure A. Alcohols **15a-g** were prepared following the General procedure for the propargyl Claisen rearrangement/Nazarov cyclization reaction. A solution of the crude alcohol **15a-g** (0.2 mmol) in DCM (0.05 M) was prepared and *N*-phenylmaleimide (1 equiv.) was added in one portion. The mixture was stirred at 25 °C under nitrogen atmosphere for 30 minutes. Water was added (5 mL) and, after separation of the phases, the product was extracted with DCM (2 x 5 mL). The combined organic extracts were dried over anhydrous Na_2SO_4. After filtration and evaporation of the solvent, the crude oil was purified by flash chromatography to afford the corresponding cycloadduct **18a-g**.

Procedure B. To a solution of commercially available gold(I) complex $IPrAuBF_4$ (2 mol%) and *N*-phenylmaleimide (0.2 mmol) in DCM (2 mL) stirred at 25 °C under nitrogen atmosphere was added a solution of propargyl vinyl ether **14h-l** (0.2 mmol) in DCM (2 mL; final concentration 0.05 M) and the reaction mixture was stirred at 25 °C until complete consumption of starting material (TLC monitoring). The mixture was diluted with MeOH (8 mL) and $NaBH_4$ (8 mg, 0.2 mmol) immediately added. After 10 minutes the reduction was completed. Water (2 mL) was added and the solvent concentrated under *vacuum* to a small volume; water was added once more to the residue (up to 10 mL) and the product extracted with DCM (3 x 10 mL). The combined organic extracts were washed with brine (20 mL) and dried over anhydrous Na_2SO_4. After filtration and evaporation of the solvent, the crude oil was purified by flash chromatography to afford the corresponding cycloadduct **18h-l**.

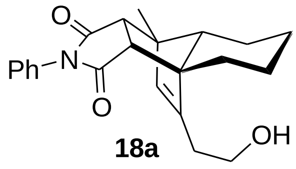

18a

Cycloadduct 18a. Prepared following Procedure A, starting from (*S*)-**15a** (36 mg, 0.2 mmol) and obtaining pure **18a** after purification by flash chromatography (EtOAc/*n*-hexane, 1:4; R_f = 0.15) as a white solid (64 mg, 91%).
e.e. 96%. M.p. 142.3 – 143.4 °C. $[\alpha]_D^{22}$ –13.3 (*c* 1.0, $CHCl_3$). ^1H NMR (400 MHz, $CDCl_3$) δ (ppm): 7.43 − 7.39 (m, 2H), 7.35 − 7.31 (m, 1H), 7.17 − 7.12 (m, 2H), 5.53 (s, 1H), 3.82 − 3.67 (m, 2H), 3.10 (AB system, J_{AB} = 7.6 Hz, 2H), 2.47 − 2.42 (m, 1H), 2.42 − 2.33 (m, 1H), 2.19 − 2.11 (m, 1H), 1.75 − 1.64 (m, 2H), 1.59 − 1.47 (m, 2H), 1.47 − 1.37 (m, 2H), 1.45 (s, 3H), 1.28 − 1.18 (m, 1H), 1.18 − 1.01 (m, 2H). Spectroscopical data identical to those reported for the corresponding racemic compound.([1])

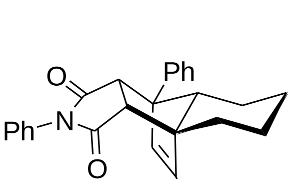

18b

Cycloadduct 18b. Prepared following Procedure A, starting from (*S*)-**15b** (48 mg, 0.2 mmol) and obtaining pure **18b** after purification by flash chromatography (EtOAc/*n*-hexane, 1:2; R_f = 0.28) as a white solid (67 mg, 81%).
e.e. 98%. $[\alpha]_D^{27}$ +17.6 (*c* 1.0, $CHCl_3$). M.p. 197.3 – 199.1 °C. ^1H NMR (400 MHz, $CDCl_3$) δ (ppm): 7.44 – 7.27 (m, 8H), 7.20 – 7.17 (m, 2H), 6.10 (s, 1H), 3.92 – 3.76 (m, 2H), 3.72 (d, *J* = 7.6 Hz, 1H), 3.27 (d, *J* = 7.6 Hz, 1H), 2.61 – 2.42 (m, 2H), 2.31 – 2.25 (m, 1H), 2.05 – 2.02 (m, 1H), 1.81 – 1.72 (m, 1H), 1.70 – 1.55 (m, 2H), 1.46 (br s, 1H), 1.32 – 1.16 (m, 3H), 1.16 – 0.98 (m, 1H). $^{13}C\{^1H\}$ NMR (100.4 MHz, $CDCl_3$) δ (ppm): 175.7, 175.4, 145.3, 138.0, 131.8, 128.9 (2C), 128.5 (2C), 128.3, 127.3 (2C),

([1]) Rinaldi, A.; Langé, V.; Scarpi, D.; Occhiato, E. G. One-Pot Access to 1,7a-Dihydro-1,3a-ethano-indene and 1,8a-Dihydro-1,3a-ethano-azulene Skeletons by a Sequential Gold(I)-Catalyzed Propargyl Claisen Rearrangement/Nazarov Cyclization/[4+2] Cycloaddition Reaction. *J. Org. Chem.* **2020**, *85*, 5078−5086.

127.0, 126.5, 126.2 (2C), 68.7, 63.5, 60.4, 59.9, 53.6, 53.2, 30.9, 28.7, 23.9, 23.6, 22.4. MS (ESI) *m/z* (%): 849 ($[2M + Na]^+$, 100), 436 ($[M + Na]^+$, 13). Anal. Calcd for $C_{27}H_{27}NO_3$: C, 78.42; H, 6.58; N, 3.39. Found: C, 78.52; H, 6.64; N, 3.15.

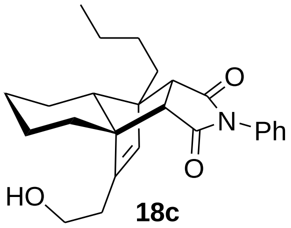

18c

Cycloadduct 18c. Prepared following Procedure A, starting from (*R*)-**15c** (44 mg, 0.2 mmol) and obtaining pure **18c** after purification by flash chromatography (EtOAc/*n*-hexane, 2:5; R_f = 0.45) as a white solid (78 mg, 99%). e.e. 85%. $[\alpha]_D^{22}$ +10.3 (*c* 1.0, $CHCl_3$). M.p. 119.4 – 120.0 °C. ^1H NMR (400 MHz, $CDCl_3$) δ (ppm): 7.43 – 7.39 (m, 2H), 7.34 – 7.30 (m, 1H), 7.16 – 7.14 (m, 2H), 5.56 (s, 1H), 3.81 – 3.68 (m, 2H), 3.28 (d, *J* = 7.6 Hz, 1H), 3.07 (d, *J* = 7.6 Hz, 1H), 2.47 – 2.34 (m, 2H), 2.20 – 2.12 (m, 1H), 2.06 – 1.98 (m, 1H), 1.84 – 1.47 (m, 7H), 1.46 – 1.32 (m, 4H), 1.28 – 1.01 (m, 3H), 0.94 (t, J = 7.2 Hz, 3H). $^{13}C\{^1H\}$ NMR (100.4 MHz, $CDCl_3$) δ (ppm): 176.6, 176.2, 144.2, 131.8, 128.9 (2C), 128.2 (2C), 126.2 (2C), 65.8, 60.4, 60.1, 59.0, 53.1, 50.0, 30.8, 28.7, 28.6, 26.9, 24.0, 23.5, 23.4, 22.2, 14.1. MS (ESI) *m/z* (%): 809 ($[2M + Na]^+$, 100), 416 ($[M + Na]^+$, 24). HRMS (ESI Orbitrap) *m/z*: $[M + H]^+$ calcd for $C_{25}H_{32}NO_3$: 394.2382. Found: 394.2377.

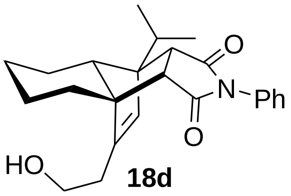

18d

Cycloadduct 18d. Prepared following Procedure A, starting from (*R*)-**15d** (41 mg, 0.2 mmol) and obtaining pure **18d** after purification by flash chromatography (EtOAc/*n*-hexane, 2:3; R_f = 0.29) as a white solid (75 mg, 99%). e.e. 93%. $[\alpha]_D^{23}$ +11.9 (*c* 1.0, $CHCl_3$). M.p. 150.2 – 151.9 °C. ^1H NMR (400 MHz, $CDCl_3$) δ (ppm): 7.43 – 7.38 (m, 2H), 7.34 – 7.30 (m, 1H), 7.16 – 7.13 (m, 2H), 5.74 (s, 1H), 3.84 – 3.72 (m, 2H), 3.37 (d, *J* = 7.6 Hz, 1H), 3.06 (d, *J* = 7.6 Hz, 1H), 2.49 – 2.36 (m, 3H), 2.22 – 2.14 (m, 1H), 1.72 – 1.59 (m, 3H), 1.58 – 1.50 (m, 1H), 1.48 – 1.44 (m, 1H), 1.40 (t, *J* = 5.6 Hz, 1H), 1.33 – 1.22 (m, 1H), 1.21 – 1.12 (m, 1H), 1.16 (d, *J* = 6.8 Hz, 3H), 1.10 – 1.01 (m, 1H), 1.03 (d, *J* = 7.2 Hz, 3H). $^{13}C\{^1H\}$ NMR (100.4 MHz, $CDCl_3$) δ (ppm): 176.7, 176.2, 144.0, 131.8, 129.0 (2C), 128.3, 126.9, 126.2 (2C), 64.8, 64.6, 60.4, 58.5, 53.2, 48.7, 30.8, 28.6, 26.9, 24.2, 23.6, 23.4, 19.0, 18.2. MS (ESI) *m/z* (%): 781 ($[2M + Na]^+$, 100), 402 ($[M + Na]^+$, 22). Anal. Calcd for $C_{24}H_{29}NO_3$: C, 75.96; H, 7.70; N, 3.69. Found: C, 75.80; H, 7.85; N, 3.52.

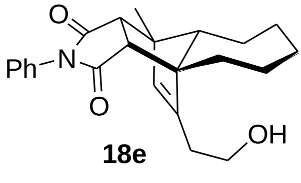

18e

Cycloadduct 18e. Prepared following Procedure A, starting from (*S*)-**15e** (38 mg, 0.2 mmol) and obtaining pure **18e** after purification by flash chromatography (EtOAc/*n*-hexane, 1:3; R_f = 0.26) as a white solid (69 mg, 94%). e.e. 94%. $[\alpha]_D^{24}$ –16.9 (*c* 1.0, $CHCl_3$). M.p. 144.3 – 145.5 °C. ^1H NMR (400 MHz, $CDCl_3$) δ (ppm): 7.42 – 7.38 (m, 2H), 7.34 – 7.30 (m, 1H), 7.15 – 7.12 (m, 2H), 5.52 (s, 1H), 3.79 – 3.70 (m, 1H), 3.70 – 3.61 m, 1H), 3.10 (q_{AB}, J_{AB} = 7.6 Hz, 2H), 2.44 – 2.34 (m, 1H), 2.29 – 2.20 (m, 1H), 2.19 – 2.02 (m, 2H), 1.86 – 1.72 (m, 2H), 1.71 – 1.58 (m, 2H), 1.56 – 1.42 (m, 2H), 1.46 (s, 3H), 1.40 – 1.30 (m, 2H), 1.29 – 1.19 (m, 2H). $^{13}C\{^1H\}$ NMR (100.4 MHz, $CDCl_3$) δ (ppm): 176.5, 176.4, 145.6, 131.7, 128.9

(2C), 128.4, 128.3, 126.2 (2C), 73.0, 63.4, 60.6, 55.7, 53.5, 51.3, 31.5, 30.3, 29.7, 27.6, 23.3, 22.2, 16.0. MS (ESI) *m/z* (%): 753 ([2M + Na]$^+$, 100), 388 ([M + Na]$^+$, 26). Anal. Calcd for $C_{23}H_{27}NO_3$: C, 75.59; H, 7.45; N, 3.83. Found: C, 75.35; H, 7.42; N, 3.83.

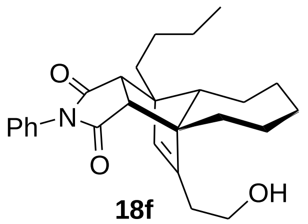

18f

Cycloadduct 18f. Prepared following Procedure A, starting from (*S*)-**15f** (47 mg, 0.2 mmol) and obtaining pure **18f** after purification by flash chromatography (EtOAc/*n*-hexane, 1:3; R_f = 0.40) as a white solid (61 mg, 75%).
e.e. 94%. $[\alpha]_D^{25}$ –15.1 (*c* 1.0, $CHCl_3$). M.p. 55.6 – 56.4 °C. ^1H NMR (400 MHz, $CDCl_3$) (4.6 : 1 mixture of isomers; attributable signals) δ (ppm): 7.42 – 7.35 (m, 2H), 7.33 – 7.28 (m, 1H), 7.14 – 7.12 (m, 2H), 5.81 (s, 1H, minor), 5.51 (s, 1H, major), 3.77 – 3.57 (m, 2H), 3.39 (d, *J* = 7.6 Hz, 1H, minor), 3.30 (d, *J* = 7.6 Hz, 1H, major), 3.07 (d, *J* = 7.6 Hz, 1H, major), 2.99 (d, *J* = 7.6 Hz, 1H, minor), 2.41 – 2.33 (m, 1H), 2.27 – 2.21 (m, 1H), 2.17 – 2.03 (m, 3H), 1.83 – 1.59 (m, 6H), 1.52 – 1.20 (m, 9H), 0.95 (t, *J* = 7.2 Hz, 3H, major), 0.92 (t, *J* = 7.2 Hz, 3H, minor). $^{13}C\{^1H\}$ NMR (100.4 MHz, $CDCl_3$) (mixture of isomers; major isomer reported) δ (ppm): 176.5 (2C), 145.1, 131.7, 128.9 (2C), 128.4, 128.2, 126.2 (2C), 70.0, 62.8, 60.5, 59.4, 50.9, 48.9, 31.4, 30.3, 29.6, 28.3, 27.6, 26.1, 23.2 (2C), 22.1, 14.0. MS (ESI) *m/z* (%): 837 ([2M + Na]$^+$, 100), 430 ([M + Na]$^+$, 19). Anal. Calcd for $C_{26}H_{33}NO_3$: C, 76.62; H, 8.16; N, 3.44. Found: C, 76.60; H, 8.18; N, 3.43.

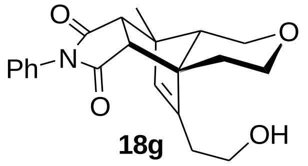

18g

Cycloadduct 18g. Prepared following Procedure A, starting from (*S*)-**15g** (36 mg, 0.2 mmol) and obtaining pure **18g** after purification by flash chromatography (EtOAc/*n*-hexane, 1:1 + 1% Et_3N; R_f = 0.18) as a white solid (58 mg, 82%).
e.e. 94%. $[\alpha]_D^{25}$ –7.5 (*c* 1.0, $CHCl_3$). M.p. 169.8 – 170.8 °C. ^1H NMR (400 MHz, $CDCl_3$) (10 : 1 mixture of isomers; major isomer reported) δ (ppm): 7.46 – 7.32 (m, 3H), 7.17 – 7.15 (m, 2H), 5.65 (s, 1H), 3.94 – 3.89 (m, 1H), 3.81 – 3.73 (m, 3H), 3.39 (t, *J* = 10.8 Hz, 1H), 3.22 – 3.13 (m, 2H), 2.38 – 2.29 (m, 2H), 2.21 – 2.15 (m, 1H), 2.00 – 1.92 (m, 1H), 1.84 – 1.80 (m, 1H), 1.65 – 1.58 (m, 2H), 1.46 (s, 3H). $^{13}C\{^1H\}$ NMR (100.4 MHz, $CDCl_3$) (mixture of isomers; major isomer reported) δ (ppm): 175.9, 175.5, 143.6, 131.7, 129.1, 129.0 (2C), 128.4, 126.2 (2C), 65.4, 65.3, 64.7, 60.1, 57.5, 54.8, 53.7, 53.0, 30.9, 29.1, 15.7. MS (ESI) *m/z* (%): 729 ([2M + Na]$^+$, 67), 376 ([M + Na]$^+$, 100). Anal. Calcd for $C_{21}H_{23}NO_4$: C, 71.37; H, 6.56; N, 3.96. Found: C, 71.27; H, 6.64; N, 3.71.

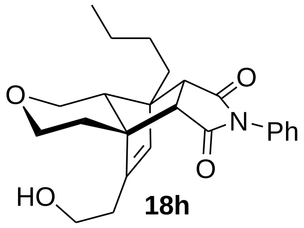

18h

Cycloadduct 18h. Prepared following Procedure B, starting from propargyl vinyl ether (*R*)-**14h** (44 mg, 0.2 mmol) and obtaining pure **18h** after purification by flash chromatography (EtOAc/*n*-hexane, 1:1; R_f = 0.32) as a white solid (58 mg, 74%).
e.e. 99%. $[\alpha]_D^{25}$ +11.0 (*c* 1.0, $CHCl_3$). M.p. 104.0 – 105.6 °C. ^1H NMR (400 MHz, $CDCl_3$) δ (ppm): 7.43 – 7.39 (m, 2H), 7.35 – 7.31 (m, 1H), 7.16 – 7.14 (m, 2H), 5.67 (s, 1H), 3.89 (dd, *J* = 12.0, 4.8 Hz, 1H), 3.81 – 3.68 (m, 3H), 3.38 (t, *J* = 10.8 Hz, 1H), 3.28 (d, *J* = 7.2 Hz, 1H), 3.21 – 3.14

(m, 2H), 2.36 – 2.28 (m, 2H), 2.20 – 2.12 (m, 1H), 1.98 – 1.80 (m, 4H), 1.76 (br s, 1H), 1.40 – 1.29 (m, 4H), 0.93 (t, *J* = 6.8 Hz, 3H). $^{13}C\{^1H\}$ NMR (100.4 MHz, $CDCl_3$) δ (ppm): 175.9, 175.5, 143.3, 131.6, 129.0 (2C), 128.6, 128.4, 126.2 (2C), 65.7, 64.6, 64.0, 60.0, 59.3, 56.7, 52.7, 50.7, 30.9, 29.5, 29.1, 27.5, 23.2, 14.0. MS (ESI) *m/z* (%): 813 ($[2M + Na]^+$, 100), 418 ($[M + Na]^+$, 67). Anal. Calcd for $C_{24}H_{29}NO_4$: C, 72.89; H, 7.39; N, 3.54. Found: C, 72.75; H, 7.42; N, 3.37.

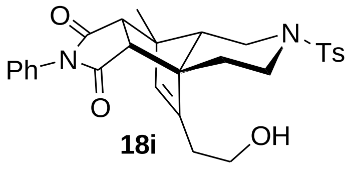

18i

Cycloadduct 18i. Prepared following Procedure B, starting from propargyl vinyl ether (*S*)-**14i** (66 mg, 0.2 mmol) and obtaining pure **18i** after purification by flash chromatography (EtOAc/*n*-hexane, 1:1; R_f = 0.23) as a white solid (65 mg, 64%).

e.e. 98%. $[\alpha]_D^{23}$ –16.3 (*c* 0.42, $CHCl_3$). M.p. 223.0 – 225.0 °C. ^1H NMR (400 MHz, $CDCl_3$) δ (ppm): 7.62 – 7.60 (m, 2H), 7.43 – 7.39 (m, 2H), 7.35 – 7.30 (m, 3H), 7.14 – 7.12 (m, 2H), 5.56 (s, 1H), 3.86 – 3.81 (m, 1H), 3.66 – 3.58 (m, 3H), 3.18 (s, 2H), 2.46 – 239 (m, 1H), 2.43 (s, 3H), 2.29 (t, *J* = 11.2 Hz, 1H), 2.19 (td, *J* = 12.4, 2.4 Hz, 1H), 2.12 – 1.97 (m, 3H), 1.91 (dd, *J* = 10.8, 4.8 Hz, 1H), 1.48 (s, 3H). $^{13}C\{^1H\}$ NMR (100.4 MHz, acetone-d6) δ (ppm): 176.6, 176.1, 144.4, 144.3, 135.5, 133.7, 130.7 (2C), 129.9, 129.6 (2C), 128.9, 128.5 (2C), 127.9 (2C), 65.5, 60.2, 58.4, 55.4, 54.9, 53.3, 45.0, 44.4, 31.6, 28.4, 21.5, 15.7. MS (ESI) *m/z* (%): 1035 ($[2M + Na]^+$, 100), 529 ($[M + Na]^+$, 23), 507 ($[M + 1]^+$, 8). HRMS (ESI Orbitrap) *m/z*: $[M + H]^+$ calcd for $C_{28}H_{31}N_2O_5S$: 507.1954. Found: 507.1946.

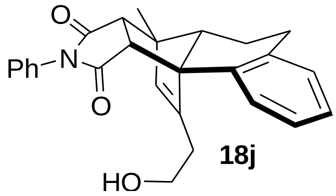

18j

Cycloadduct 18j. Prepared following Procedure B, starting from propargyl vinyl ether (*S*)-**14j** (45 mg, 0.2 mmol) and obtaining pure **18j** after purification by flash chromatography (EtOAc/*n*-hexane, 1:2; R_f = 0.26) as a white solid (70 mg, 88%).

e.e. 95%. $[\alpha]_D^{22}$ –112.5 (*c* 0.92, $CHCl_3$). M.p. 164.3 – 165.9 °C. ^1H NMR (400 MHz, $CDCl_3$) δ (ppm): 8.35 (d, *J* = 7.6 Hz, 1H), 7.47 – 7.43 (m, 2H), 7.39 – 7.35 (m, 1H), 7.28 – 7.12 (m, 5H), 5.57 (s, 1H), 3.58 (d, *J* = 7.6 Hz, 1H), 3.47 – 3.39 (m, 1H), 3.33 – 3.25 (m, 2H), 2.88 – 2.77 (m, 2H), 2.25 – 2.17 (m, 1H), 2.04 – 1.95 (m, 2H), 1.78 – 1.65 (m, 2H), 1.57 (s, 3H), 1.11 (br m, 1H). $^{13}C\{^1H\}$ NMR (100.4 MHz, $CDCl_3$) δ (ppm): 176.2, 176.0, 148.0, 137.4, 135.6, 313.9, 130.0, 129.1 (2C), 128.7, 128.5, 127.1, 126.7, 126.5 (2C), 126.3, 67.2, 62.4, 60.3, 55.5, 54.7, 52.1, 32.4, 29.1, 18.8, 15.3. MS (ESI) *m/z* (%): 821 ($[2M + Na]^+$, 100), 422 ($[M + Na]^+$, 38). Anal. Calcd for $C_{26}H_{25}NO_3$: C, 78.17; H, 6.31; N, 3.51. Found: C, 77.92; H, 6.35; N, 3.72.

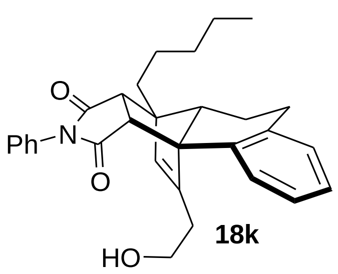

18k

Cycloadduct 18k. Prepared following Procedure B, starting from propargyl vinyl ether (*S*)-**14k** (56 mg, 0.2 mmol) and obtaining pure **18k** after purification by flash chromatography (EtOAc/*n*-hexane, 1:4; R_f = 0.15) as a white solid (77 mg, 84%).

e.e. 94%. $[\alpha]_D^{20}$ –68.7 (*c* 1.0, $CHCl_3$). M.p. 68.9 – 71.1 °C. ^1H NMR (400

MHz, $CDCl_3$) (8 : 1 mixture of diastereoisomers; major isomer reported) δ (ppm): 8.38 (d, J = 7.6 Hz, 1H), 7.47 – 7.43 (m, 2H), 7.39 – 7.35 (m, 1H), 7.28 – 7.12 (m, 5H), 5.58 (s, 1H), 3.52 (q_{AB}, J_{AB} = 7.6 Hz, 2H), 3.46 – 3.38 (m, 1H), 3.33 – 3.24 (m, 1H), 2.86 – 2.80 (m, 2H), 2.25 – 2.08 (m, 3H), 2.03 – 1.97 (m, 1H), 1.93 – 1.86 (m, 1H), 1.81 – 1.71 (m, 1H), 1.70 – 1.56 (m, 2H), 1.44 – 1.33 (m, 5H), 1.15 (br s, 1H), 0.93 (t, J = 6.0 HZ, 3H). $^{13}C\{^1H\}$ NMR (100.4 MHz, $CDCl_3$) (mixture of diastereoisomers; major isomer reported) δ (ppm): 176.4, 176.0, 147.5, 137.3, 135.6, 131.9, 130.0, 129.0 (2C), 128.7, 128.5, 127.1, 126.5 (3C), 126.3, 65.0, 61.9, 60.3, 59.4, 51.8, 50.8, 32.43, 32.40, 29.1, 28.6, 24.2, 22.6, 19.3, 14.1. MS (ESI) *m/z* (%): 933 ($[2M + Na]^+$, 100), 478 ($[M + Na]^+$, 44). Anal. Calcd for $C_{30}H_{33}NO_3$: C, 79.09; H, 7.30; N, 3.07. Found: C, 78.94; H, 7.22; N, 2.96.

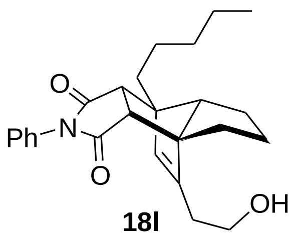

18l

Cycloadduct 18l. Prepared following Procedure B, starting from propargyl vinyl ether (*S*)-**14l** (65 mg, 0.3 mmol) and obtaining pure **18l** after purification by flash chromatography (EtOAc/*n*-hexane, 1:2; R_f = 0.19) as a white foam (53 mg, 45%). e.e. 92%. $[\alpha]_D^{18}$ –28.6 (*c* 0.51, $CHCl_3$). ^1H NMR (400 MHz, $CDCl_3$) δ (ppm): 7.43 – 7.38 (m, 2H), 7.35 – 7.31 (m, 1H), 7.17 – 7.12 (m, 2H), 5.56 (s, 1H), 3.78 – 3.72 (m, 1H), 3.69 – 3.63 (m, 1H), 3.53 (d, J = 7.2 Hz, 1H), 3.19 (d, J = 7.6 Hz, 1H), 2.40 – 2.32 (m, 1H), 2.25 – 2.18 (m, 1H), 2.17 – 2.08 (m, 2H), 2.08 – 1.93 (m, 3H), 1.92 – 1.82 (m, 1H), 1.68 –1.60 (m, 1H), 1.54 (br s, 1H), 1.45 – 1.27 (m, 8H), 0.90 (t, J = 6.8 Hz, 3H). $^{13}C\{^1H\}$ NMR (100.4 MHz, $CDCl_3$) δ (ppm): 176.3, 176.1, 145.7, 131.9, 128.9 (2C), 128.3, 127.3, 126.4 (2C), 73.4, 69.9, 60.7, 58.6, 57.7, 48.4, 32.4, 31.3, 31.0, 30.1, 25.6, 25.5, 22.5, 20.2, 14.0. MS (ESI) *m/z* (%): 809 ($[2M + Na]^+$, 100), 416 ($[M + Na]^+$, 21). HRMS (ESI Orbitrap) *m/z*: $[M + H]^+$ calcd for $C_{25}H_{32}NO_3$: 394.2382. Found: 394.2375.

2.3 HPLC chromatograms of racemic and chiral compounds 18a-l.

Column A: Lux 5µ Amylose-1 column, 250 x 4.60 mm.

Column B: Lux 5µ Cellulose-4 column, 250 x 4.60 mm.

Conditions: 0.5 mL/min flow rate in *n*-hexane/IPA mixture as the eluent (isocratic) at room temperature. Injection of 1-2 µL of a 5 mM solution in CH_3OH. Detection at λ = 223 nm.

**Compound**	**Column**	***n*-hexane/IPA (%)**	**R_t (min)**	**e.e. (%)**
**18a**	A	50-50	17.83 and 26.03	96
**18b**	B	50-50	26.82 and 36.53	98
**18c**	A	50-50	14.72 and 21.75	85
**18d**	A	50-50	20.62 and 26.01	93
**18e**	A	50-50	19.41 and 20.85	94
**18f**	A	50-50	12.28 and 15.01	94
**18g**	B	65-35	40.77 and 44.99	94
**18h**	B	65-35	27.35 and 31.64	99
**18i**	A	20-80	27.27 and 35.37	98
**18j**	A	50-50	17.53 and 23.44	95
**18k**	A	70-30	27.34 and 29.10	94
**18l**	A	50-50	14.42 and 20.31	92

Operator:HPLC Timebase:HPLC2 Sequence:Cicloaddotto_C6Me_10feb25 Page 1-1
10/2/2025 5.43 PM

1 MF20 C6Me rac 5mM MeOH

Sample Name:	**MF20 C6Me rac 5mM MeOH**	*Injection Volume:*	**2,0**
Vial Number:	**BE6**	*Channel:*	**UV_VIS_1**
Sample Type:	**unknown**	*Wavelength:*	**223**
Control Program:	**Iso_50IPA_50Hex**	*Bandwidth:*	**n.a.**
Quantif. Method:	**INSTRUMENTS_IQ_Iso17D**	*Dilution Factor:*	**1,0000**
Recording Time:	**10/2/2025 16.41**	*Sample Weight:*	**1,0000**
Run Time (min):	**35,00**	*Sample Amount:*	**1,0000**

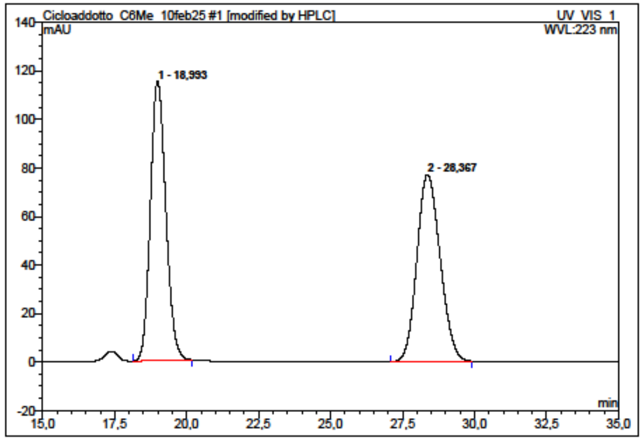

No.	Ret.Time min	Peak Name	Height mAU	Area mAU*min	Rel.Area %	Amount	Type
1	18,99	n.a.	115,288	71,249	49,66	n.a.	BMB
2	28,37	n.a.	77,155	72,231	50,34	n.a.	BMB
Total:			192,443	143,481	100,00	0,000	

default/Integration
Chromeleon (c) Dionex 1996-2006
Version 6.80 SR9 Build 2673 (161349)

Operator:HPLC Timebase:HPLC2 Sequence:MF20_01mar24 Page 1-1
1/3/2024 12.39 PM

3 MF18 chir 5mM MeOH 50IPA

Sample Name:	**MF18 chir 5mM MeOH 50IPA**	*Injection Volume:*	**2,0**
Vial Number:	**BE1**	*Channel:*	**UV_VIS_1**
Sample Type:	**unknown**	*Wavelength:*	**223**
Control Program:	**Iso_50IPA_50Hex**	*Bandwidth:*	**n.a.**
Quantif. Method:	**INSTRUMENTS_IQ_Iso17D**	*Dilution Factor:*	**1,0000**
Recording Time:	**1/3/2024 11.56**	*Sample Weight:*	**1,0000**
Run Time (min):	**40,00**	*Sample Amount:*	**1,0000**

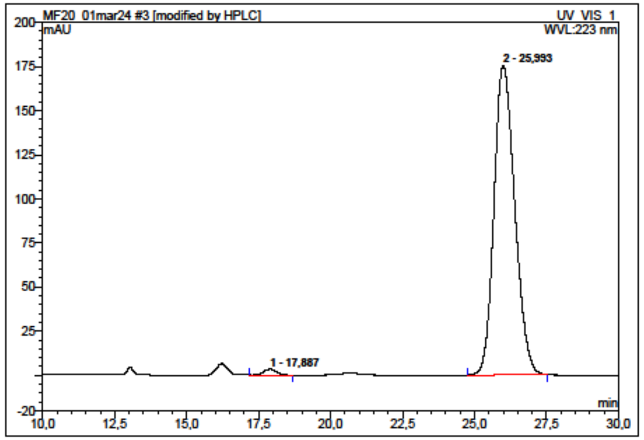

No.	Ret.Time min	Peak Name	Height mAU	Area mAU*min	Rel.Area %	Amount	Type
1	17,89	n.a.	3,371	1,780	1,20	n.a.	BMB*
2	25,99	n.a.	175,658	146,403	98,80	n.a.	BMB
Total:			179,029	148,183	100,00	0,000	

default/Integration
Chromeleon (c) Dionex 1996-2006
Version 6.80 SR9 Build 2673 (161349)

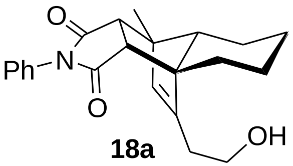

18a

HPLC analysis of compound racemic (left) and chiral (right) compound **18a**.

Operator:HPLC Timebase:HPLC2 Sequence:Cicloaddotti_C6Ph_alfaTPent_01apr25 Page 1-1
1/4/2025 1.40 PM

3 GT33 C6Ph rac 5 mM

Sample Name:	**GT33 C6Ph rac 5 mM**	*Injection Volume:*	**2,0**
Vial Number:	**BB1**	*Channel:*	**UV_VIS_1**
Sample Type:	**unknown**	*Wavelength:*	**223**
Control Program:	**Iso_50IPA_50Hex**	*Bandwidth:*	**n.a.**
Quantif. Method:	**INSTRUMENTS_IQ_Iso17D**	*Dilution Factor:*	**1,0000**
Recording Time:	**1/4/2025 12.37**	*Sample Weight:*	**1,0000**
Run Time (min):	**50,00**	*Sample Amount:*	**1,0000**

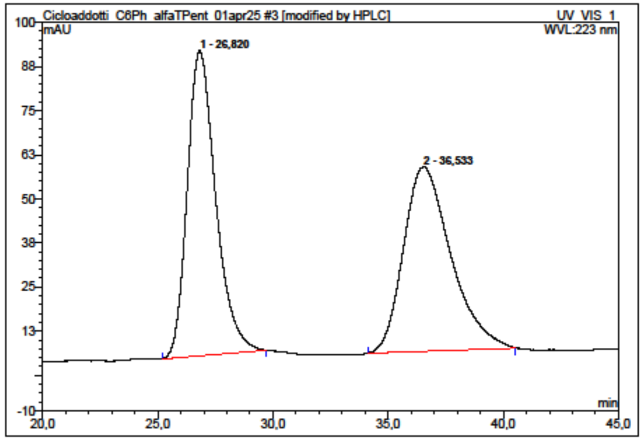

No.	Ret.Time min	Peak Name	Height mAU	Area mAU*min	Rel.Area %	Amount	Type
1	26,82	n.a.	86,399	123,883	49,81	n.a.	BMB
2	36,53	n.a.	52,294	124,837	50,19	n.a.	BMB
Total:			138,693	248,720	100,00	0,000	

default/Integration

Chromeleon (c) Dionex 1996-2006
Version 6.80 SR9 Build 2673 (161349)

Operator:HPLC Timebase:HPLC2 Sequence:Cicloaddotti_C6Ph_alfaTPent_01apr25 Page 1-1
1/4/2025 2.22 PM

4 MF31 C6Ph chir 5 mM

Sample Name:	**MF31 C6Ph chir 5 mM**	*Injection Volume:*	**2,0**
Vial Number:	**BB2**	*Channel:*	**UV_VIS_1**
Sample Type:	**unknown**	*Wavelength:*	**223**
Control Program:	**Iso_50IPA_50Hex**	*Bandwidth:*	**n.a.**
Quantif. Method:	**INSTRUMENTS_IQ_Iso17D**	*Dilution Factor:*	**1,0000**
Recording Time:	**1/4/2025 13.28**	*Sample Weight:*	**1,0000**
Run Time (min):	**50,00**	*Sample Amount:*	**1,0000**

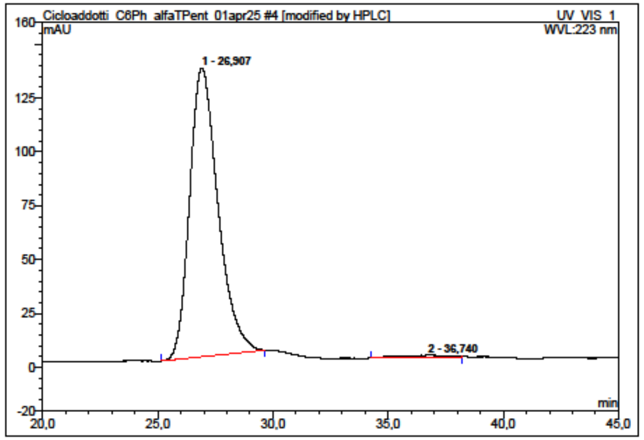

No.	Ret.Time min	Peak Name	Height mAU	Area mAU*min	Rel.Area %	Amount	Type
1	26,91	n.a.	133,958	191,257	98,75	n.a.	BMB*
2	36,74	n.a.	0,981	2,413	1,25	n.a.	BMB*
Total:			134,938	193,670	100,00	0,000	

default/Integration

Chromeleon (c) Dionex 1996-2006
Version 6.80 SR9 Build 2673 (161349)

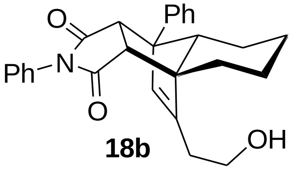

18b

HPLC analysis of compound racemic (left) and chiral (right) compound **18b**.

Operator:HPLC Timebase:HPLC2 Sequence:Cicloaddotti_Sonogashira_08MAR24 Page 1-1
16/4/2024 11.57 AM

11 MF34 C6nBu rac CA

Sample Name:	MF34 C6nBu rac CA	*Injection Volume:*	2,0
Vial Number:	BA5	*Channel:*	UV_VIS_1
Sample Type:	unknown	*Wavelength:*	223
Control Program:	Iso_50IPA_50Hex	*Bandwidth:*	n.a.
Quantif. Method:	INSTRUMENTS_IQ_Iso17D	*Dilution Factor:*	1,0000
Recording Time:	16/4/2024 11.25	*Sample Weight:*	1,0000
Run Time (min):	30,00	*Sample Amount:*	1,0000

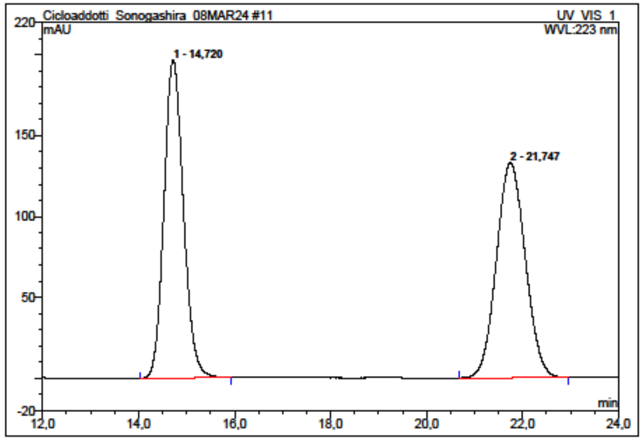

No.	Ret.Time min	Peak Name	Height mAU	Area mAU*min	Rel.Area %	Amount	Type
1	14,72	n.a.	196,788	92,015	49,84	n.a.	BMB
2	21,75	n.a.	132,847	92,616	50,16	n.a.	BMB
Total:			329,635	184,631	100,00	0,000	

default/Integration

Chromeleon (c) Dionex 1996-2006
Version 6.80 SR9 Build 2673 (161349)

Operator:HPLC Timebase:HPLC2 Sequence:Cicloaddotti_Sonogashira_08MAR24 Page 1-1
5/4/2024 11.39 AM

8 GT43 C6nBu chir (altro enantiomero) 50IPA

Sample Name:	GT43 C6nBu chir (altro enantiomero) 50IPA	*Injection Volume:*	2,0
Vial Number:	BE5	*Channel:*	UV_VIS_1
Sample Type:	unknown	*Wavelength:*	223
Control Program:	Iso_50IPA_50Hex	*Bandwidth:*	n.a.
Quantif. Method:	INSTRUMENTS_IQ_Iso17D	*Dilution Factor:*	1,0000
Recording Time:	4/4/2024 18.25	*Sample Weight:*	1,0000
Run Time (min):	30,00	*Sample Amount:*	1,0000

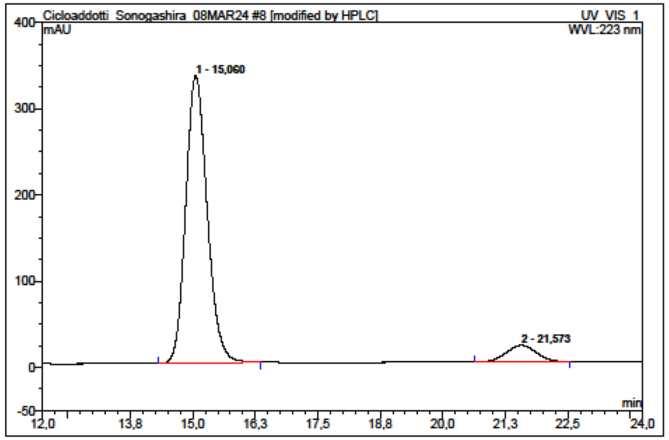

No.	Ret.Time min	Peak Name	Height mAU	Area mAU*min	Rel.Area %	Amount	Type
1	15,06	n.a.	332,657	164,991	92,54	n.a.	BMB
2	21,57	n.a.	19,043	13,302	7,46	n.a.	BMB
Total:			351,699	178,293	100,00	0,000	

default/Integration

Chromeleon (c) Dionex 1996-2006
Version 6.80 SR9 Build 2673 (161349)

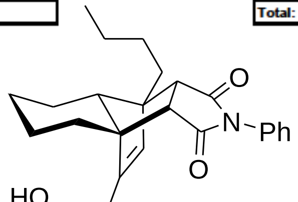

18c

HPLC analysis of compound racemic (left) and chiral (right) compound **18c**.

Operator:HPLC Timebase:HPLC2 Sequence:Cicloaddotti_Sonogashira_08MAR24 Page 1-1
10/4/2024 3.53 PM

10 MF35 C6iPr rac (II iniezione)

Sample Name:	MF35 C6iPr rac (II iniezione)	*Injection Volume:*	2,0
Vial Number:	BE5	*Channel:*	UV_VIS_1
Sample Type:	unknown	*Wavelength:*	223
Control Program:	Iso_50IPA_50Hex	*Bandwidth:*	n.a.
Quantif. Method:	INSTRUMENTS_IQ_Iso17D	*Dilution Factor:*	1,0000
Recording Time:	10/4/2024 14.08	*Sample Weight:*	1,0000
Run Time (min):	30,00	*Sample Amount:*	1,0000

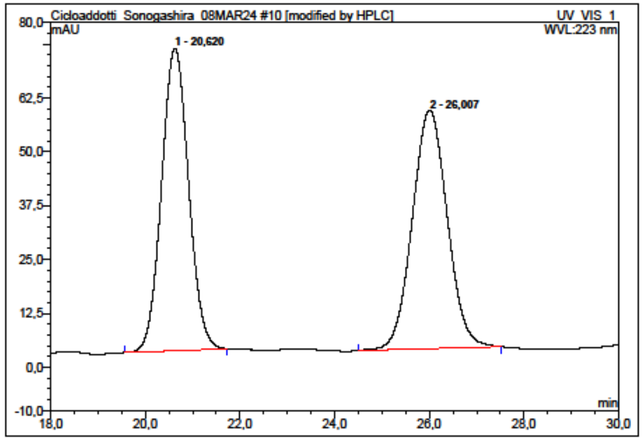

No.	Ret.Time min	Peak Name	Height mAU	Area mAU*min	Rel.Area %	Amount	Type
1	20,62	n.a.	70,137	46,357	49,48	n.a.	BMB
2	26,01	n.a.	55,285	47,327	50,52	n.a.	BMB
Total:			125,422	93,684	100,00	0,000	

default/Integration

Chromeleon (c) Dionex 1996-2006
Version 6.80 SR9 Build 2673 (161349)

Operator:HPLC Timebase:HPLC2 Sequence:Cicloaddotti_Sonogashira_08MAR24 Page 1-1
10/4/2024 3.56 PM

9 MF38 C6iPr chir 50IPA

Sample Name:	MF38 C6iPr chir 50IPA	*Injection Volume:*	2,0
Vial Number:	BE4	*Channel:*	UV_VIS_1
Sample Type:	unknown	*Wavelength:*	223
Control Program:	Iso_50IPA_50Hex	*Bandwidth:*	n.a.
Quantif. Method:	INSTRUMENTS_IQ_Iso17D	*Dilution Factor:*	1,0000
Recording Time:	10/4/2024 13.27	*Sample Weight:*	1,0000
Run Time (min):	30,00	*Sample Amount:*	1,0000

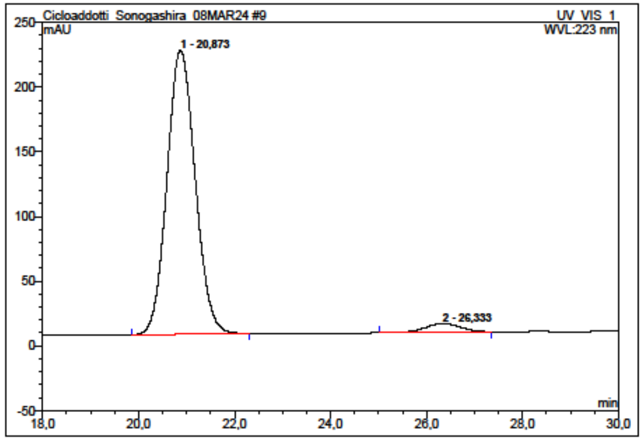

No.	Ret.Time min	Peak Name	Height mAU	Area mAU*min	Rel.Area %	Amount	Type
1	20,87	n.a.	219,500	149,209	96,26	n.a.	BMB
2	26,33	n.a.	6,698	5,801	3,74	n.a.	BMB
Total:			226,198	155,010	100,00	0,000	

default/Integration

Chromeleon (c) Dionex 1996-2006
Version 6.80 SR9 Build 2673 (161349)

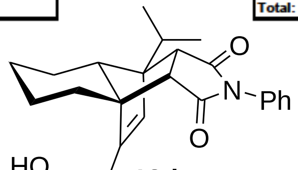

18d

HPLC analysis of compound racemic (left) and chiral (right) compound **18d**.

Operator:HPLC Timebase:HPLC2 Sequence:Cicloaddotti_C7_Sonogashira_18APR24 Page 1-1
19/4/2024 2.55 PM

3 GT50 C7Me rac 50IPA II iniezione

Sample Name:	**GT50 C7Me rac 50IPA II iniezione**	*Injection Volume:*	**2,0**
Vial Number:	**BA2**	*Channel:*	**UV_VIS_1**
Sample Type:	**unknown**	*Wavelength:*	**223**
Control Program:	**Iso_50IPA_50Hex**	*Bandwidth:*	**n.a.**
Quantif. Method:	**INSTRUMENTS_IQ_Iso17D**	*Dilution Factor:*	**1,0000**
Recording Time:	**19/4/2024 11.51**	*Sample Weight:*	**1,0000**
Run Time (min):	**30,00**	*Sample Amount:*	**1,0000**

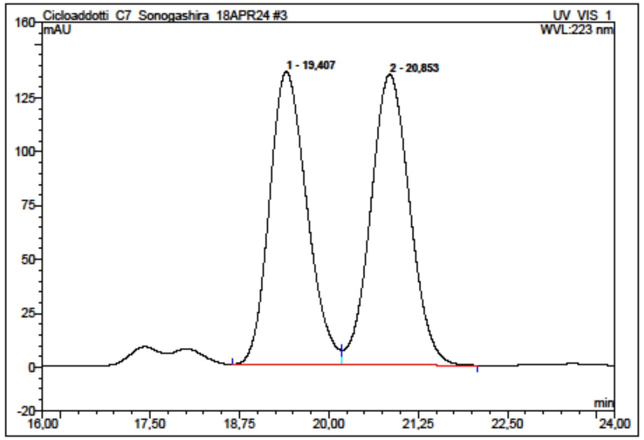

No.	Ret.Time min	Peak Name	Height mAU	Area mAU*min	Rel.Area %	Amount	Type
1	19,41	n.a.	135,879	82,453	49,25	n.a.	BM
2	20,85	n.a.	134,723	84,974	50,75	n.a.	MB
Total:			270,602	167,427	100,00	0,000	

default/Integration

Chromeleon (c) Dionex 1996-2006
Version 6.80 SR9 Build 2673 (161349)

Operator:HPLC Timebase:HPLC2 Sequence:Cicloaddotti_C7_Sonogashira_18APR24 Page 1-1
19/4/2024 2.57 PM

4 MF47 C7Me chir 50IPA II iniezione

Sample Name:	**MF47 C7Me chir 50IPA II iniezione**	*Injection Volume:*	**2,0**
Vial Number:	**BA3**	*Channel:*	**UV_VIS_1**
Sample Type:	**unknown**	*Wavelength:*	**223**
Control Program:	**Iso_50IPA_50Hex**	*Bandwidth:*	**n.a.**
Quantif. Method:	**INSTRUMENTS_IQ_Iso17D**	*Dilution Factor:*	**1,0000**
Recording Time:	**19/4/2024 12.21**	*Sample Weight:*	**1,0000**
Run Time (min):	**30,00**	*Sample Amount:*	**1,0000**

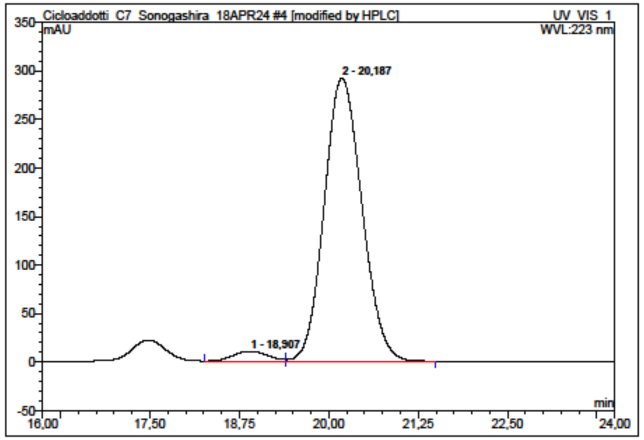

No.	Ret.Time min	Peak Name	Height mAU	Area mAU*min	Rel.Area %	Amount	Type
1	18,91	n.a.	10,205	5,681	3,09	n.a.	BM *
2	20,19	n.a.	292,016	178,022	96,91	n.a.	MB*
Total:			302,220	183,704	100,00	0,000	

default/Integration

Chromeleon (c) Dionex 1996-2006
Version 6.80 SR9 Build 2673 (161349)

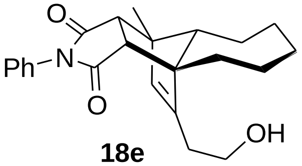

18e

HPLC analysis of compound racemic (left) and chiral (right) compound **18e**.

Operator:HPLC Timebase:HPLC2 Sequence:Cicloaddotti_C7Bu_C6OMe_10giu24 Page 1-1
10/6/2024 3.20 PM

2 GT55 C7Bu rac 50IPA

Sample Name:	GT55 C7Bu rac 50IPA	*Injection Volume:*	2,0
Vial Number:	BA2	*Channel:*	UV_VIS_1
Sample Type:	unknown	*Wavelength:*	223
Control Program:	Iso_50IPA_50Hex	*Bandwidth:*	n.a.
Quantif. Method:	INSTRUMENTS_IQ_Iso17D	*Dilution Factor:*	1,0000
Recording Time:	10/6/2024 11.58	*Sample Weight:*	1,0000
Run Time (min):	30,00	*Sample Amount:*	1,0000

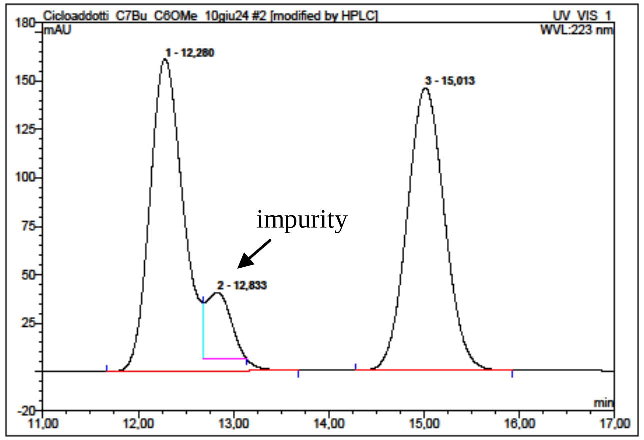

No.	Ret.Time min	Peak Name	Height mAU	Area mAU*min	Rel.Area %	Amount	Type
1	12,28	n.a.	160,877	68,189	47,16	n.a.	BMB*
2	12,83	n.a.	34,454	10,368	7,17	n.a.	Rd*
3	15,01	n.a.	145,899	66,020	45,66	n.a.	BMB
Total:			341,229	144,576	100,00	0,000	

default/Integration

Chromeleon (c) Dionex 1996-2006
Version 6.80 SR9 Build 2673 (161349)

Operator:HPLC Timebase:HPLC2 Sequence:Cicloaddotti_C7Bu_C6OMe_10giu24 Page 1-1
10/6/2024 3.21 PM

3 GT67 C7Bu chir 50IPA

Sample Name:	GT67 C7Bu chir 50IPA	*Injection Volume:*	2,0
Vial Number:	BA3	*Channel:*	UV_VIS_1
Sample Type:	unknown	*Wavelength:*	223
Control Program:	Iso_50IPA_50Hex	*Bandwidth:*	n.a.
Quantif. Method:	INSTRUMENTS_IQ_Iso17D	*Dilution Factor:*	1,0000
Recording Time:	10/6/2024 12.29	*Sample Weight:*	1,0000
Run Time (min):	30,00	*Sample Amount:*	1,0000

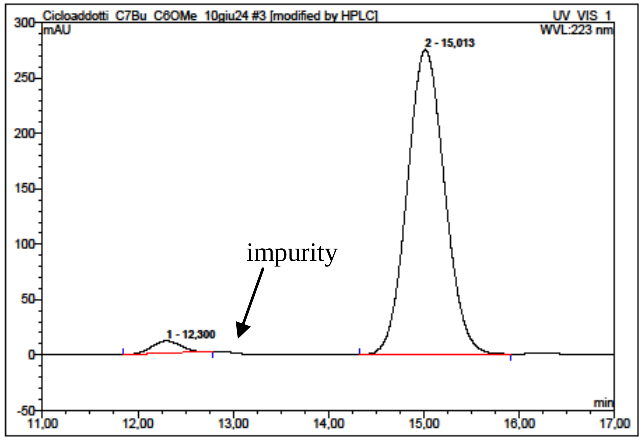

No.	Ret.Time min	Peak Name	Height mAU	Area mAU*min	Rel.Area %	Amount	Type
1	12,30	n.a.	10,864	3,789	2,95	n.a.	BMB*
2	15,01	n.a.	275,230	124,582	97,05	n.a.	BMB
Total:			286,094	128,371	100,00	0,000	

default/Integration

Chromeleon (c) Dionex 1996-2006
Version 6.80 SR9 Build 2673 (161349)

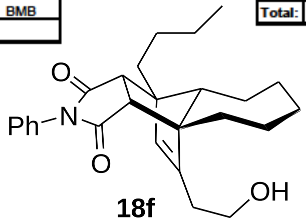

18f

HPLC analysis of compound racemic (left) and chiral (right) compound **18f**.

Operator:HPLC Timebase:HPLC2 Sequence:Cicloaddotti_C6OMe_11giu24 Page 1-1
11/6/2024 5.27 PM

7 GT75 C6[O]Me rac 35IPA

Sample Name:	GT75 C6[O]Me rac 35IPA	*Injection Volume:*	2,0
Vial Number:	BD1	*Channel:*	UV_VIS_1
Sample Type:	unknown	*Wavelength:*	223
Control Program:	Iso_35IPA	*Bandwidth:*	n.a.
Quantif. Method:	INSTRUMENTS_IQ_Iso17D	*Dilution Factor:*	1,0000
Recording Time:	11/6/2024 14.33	*Sample Weight:*	1,0000
Run Time (min):	60,02	*Sample Amount:*	1,0000

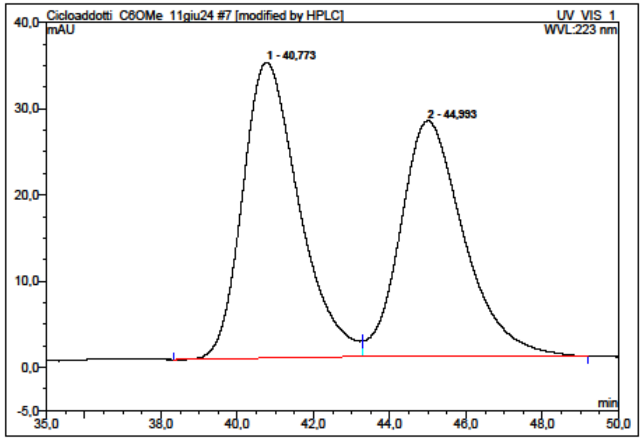

No.	Ret.Time min	Peak Name	Height mAU	Area mAU*min	Rel.Area %	Amount	Type
1	40,77	n.a.	34,315	58,740	52,68	n.a.	BM *
2	44,99	n.a.	27,326	52,773	47,32	n.a.	MB*
Total:			61,641	111,513	100,00	0,000	

default/Integration

Chromeleon (c) Dionex 1996-2006
Version 6.80 SR9 Build 2673 (161349)

Operator:HPLC Timebase:HPLC2 Sequence:Cicloaddotti_C6OMe_11giu24 Page 1-1
1/4/2025 9.31 AM

8 GT63 C6[O]Me chir 35IPA

Sample Name:	GT63 C6[O]Me chir 35IPA	*Injection Volume:*	2,0
Vial Number:	BB2	*Channel:*	UV_VIS_1
Sample Type:	unknown	*Wavelength:*	223
Control Program:	Iso_35IPA	*Bandwidth:*	n.a.
Quantif. Method:	INSTRUMENTS_IQ_Iso17D	*Dilution Factor:*	1,0000
Recording Time:	11/6/2024 15.34	*Sample Weight:*	1,0000
Run Time (min):	60,02	*Sample Amount:*	1,0000

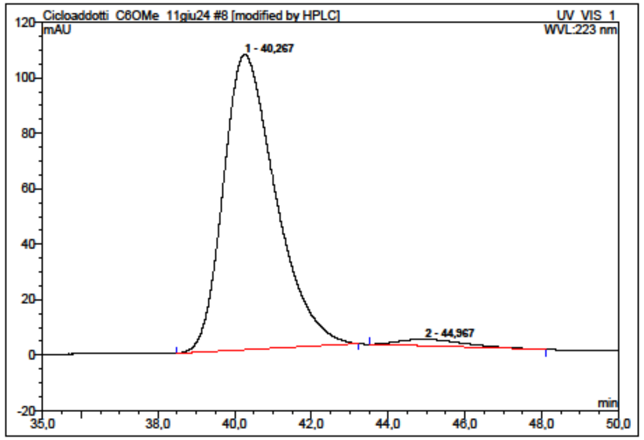

No.	Ret.Time min	Peak Name	Height mAU	Area mAU*min	Rel.Area %	Amount	Type
1	40,27	n.a.	106,417	168,200	97,32	n.a.	BMB
2	44,97	n.a.	2,421	4,640	2,68	n.a.	BMB*
Total:			108,837	172,839	100,00	0,000	

default/Integration

Chromeleon (c) Dionex 1996-2006
Version 6.80 SR9 Build 2673 (161349)

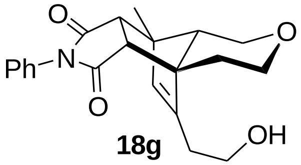

18g

HPLC analysis of compound racemic (left) and chiral (right) compound **18g**.

Operator:HPLC Timebase:HPLC2 Sequence:Cicloaddotti_C6O_19giu24 Page 1-1
19/6/2024 1.19 PM

3 GT78 C6[O]Bu rac 35IPA

Sample Name:	GT78 C6[O]Bu rac 35IPA	*Injection Volume:*	2,0
Vial Number:	BE1	*Channel:*	UV_VIS_1
Sample Type:	unknown	*Wavelength:*	223
Control Program:	Iso_35IPA_45min	*Bandwidth:*	n.a.
Quantif. Method:	INSTRUMENTS_IQ_Iso17D	*Dilution Factor:*	1,0000
Recording Time:	19/6/2024 12.26	*Sample Weight:*	1,0000
Run Time (min):	45,00	*Sample Amount:*	1,0000

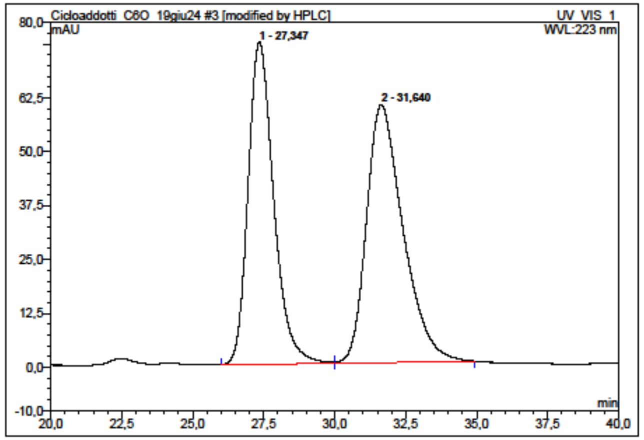

No.	Ret.Time min	Peak Name	Height mAU	Area mAU*min	Rel.Area %	Amount	Type
1	27,35	n.a.	74,674	78,148	46,71	n.a.	BM
2	31,64	n.a.	59,823	89,145	53,29	n.a.	MB
Total:			134,497	167,293	100,00	0,000	

default/Integration

Chromeleon (c) Dionex 1996-2006
Version 6.80 SR9 Build 2673 (161349)

Operator:HPLC Timebase:HPLC2 Sequence:Cicloaddotti_C6O_19giu24 Page 1-1
19/6/2024 1.18 PM

2 GT82 C6[O]Bu chir 35IPA

Sample Name:	GT82 C6[O]Bu chir 35IPA	*Injection Volume:*	2,0
Vial Number:	BE2	*Channel:*	UV_VIS_1
Sample Type:	unknown	*Wavelength:*	223
Control Program:	Iso_35IPA_45min	*Bandwidth:*	n.a.
Quantif. Method:	INSTRUMENTS_IQ_Iso17D	*Dilution Factor:*	1,0000
Recording Time:	19/6/2024 11.41	*Sample Weight:*	1,0000
Run Time (min):	45,00	*Sample Amount:*	1,0000

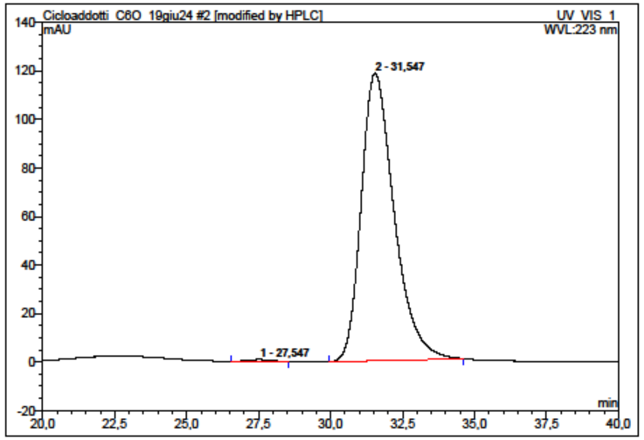

No.	Ret.Time min	Peak Name	Height mAU	Area mAU*min	Rel.Area %	Amount	Type
1	27,55	n.a.	0,899	0,795	0,50	n.a.	BMB*
2	31,55	n.a.	118,514	157,391	99,50	n.a.	BMB
Total:			119,414	158,186	100,00	0,000	

default/Integration

Chromeleon (c) Dionex 1996-2006
Version 6.80 SR9 Build 2673 (161349)

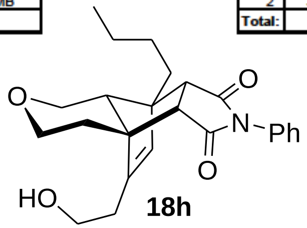

18h

HPLC analysis of compound racemic (left) and chiral (right) compound **18h**.

Operator:HPLC Timebase:HPLC2 Sequence:CICLOADDOTTI_NTs_04DIC24 Page 1-1
4/12/2024 1.21 PM

4 DSpc1291 rac NTs Me 5mM in acetone

Sample Name:	**DSpc1291 rac NTs Me 5mM in acetone**	*Injection Volume:*	**2,0**
Vial Number:	**BD1**	*Channel:*	**UV_VIS_1**
Sample Type:	**unknown**	*Wavelength:*	**223**
Control Program:	**Iso_80IPA_45min**	*Bandwidth:*	**n.a.**
Quantif. Method:	**INSTRUMENTS_IQ_Iso17D**	*Dilution Factor:*	**1,0000**
Recording Time:	**4/12/2024 10.26**	*Sample Weight:*	**1,0000**
Run Time (min):	**45,00**	*Sample Amount:*	**1,0000**

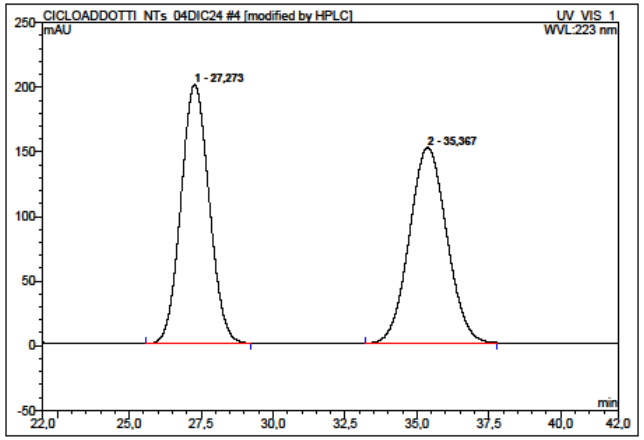

No.	Ret.Time min	Peak Name	Height mAU	Area mAU*min	Rel.Area %	Amount	Type
1	27,27	n.a.	200,144	227,248	50,00	n.a.	BMB
2	35,37	n.a.	151,263	227,290	50,00	n.a.	BMB
Total:			351,408	454,539	100,00	0,000	

default/Integration

Chromeleon (c) Dionex 1996-2006
Version 6.80 SR9 Build 2673 (161349)

Operator:HPLC Timebase:HPLC2 Sequence:CICLOADDOTTI_NTs_04DIC24 Page 1-1
4/12/2024 1.22 PM

5 DSpc1296 chir NTs Me 5mM in acetone

Sample Name:	**DSpc1296 chir NTs Me 5mM in acetone**	*Injection Volume:*	**2,0**
Vial Number:	**BD2**	*Channel:*	**UV_VIS_1**
Sample Type:	**unknown**	*Wavelength:*	**223**
Control Program:	**Iso_80IPA_45min**	*Bandwidth:*	**n.a.**
Quantif. Method:	**INSTRUMENTS_IQ_Iso17D**	*Dilution Factor:*	**1,0000**
Recording Time:	**4/12/2024 11.12**	*Sample Weight:*	**1,0000**
Run Time (min):	**45,00**	*Sample Amount:*	**1,0000**

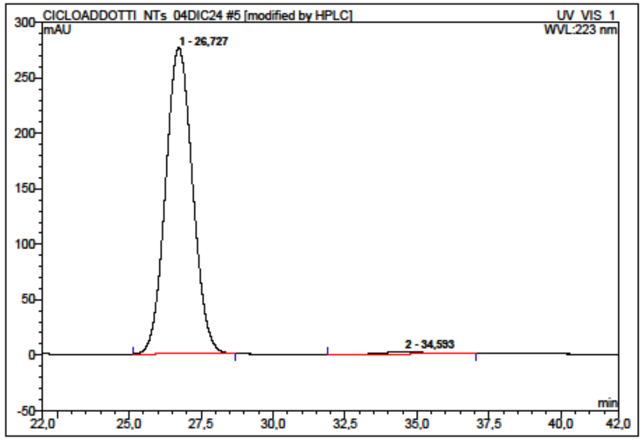

No.	Ret.Time min	Peak Name	Height mAU	Area mAU*min	Rel.Area %	Amount	Type
1	26,73	n.a.	276,158	300,309	98,99	n.a.	BMB
2	34,59	n.a.	2,283	3,076	1,01	n.a.	BMB*
Total:			278,441	303,385	100,00	0,000	

default/Integration

Chromeleon (c) Dionex 1996-2006
Version 6.80 SR9 Build 2673 (161349)

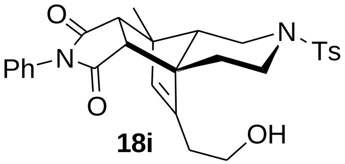

18i

HPLC analysis of compound racemic (left) and chiral (right) compound **18i**.

Operator:HPLC Timebase:HPLC2 Sequence:Cicloaddotti_Sonogashira_29nov24 Page 1-1
29/11/2024 3.40 PM

1 DSpc1303 rac alfaT Me 5mM

Sample Name:	**DSpc1303 rac alfaT Me 5mM**	*Injection Volume:*	**2,0**
Vial Number:	**BB1**	*Channel:*	**UV_VIS_1**
Sample Type:	**unknown**	*Wavelength:*	**223**
Control Program:	**Iso_50IPA_50Hex**	*Bandwidth:*	**n.a.**
Quantif. Method:	**INSTRUMENTS_IQ_Iso17D**	*Dilution Factor:*	**1,0000**
Recording Time:	**29/11/2024 14.19**	*Sample Weight:*	**1,0000**
Run Time (min):	**30,00**	*Sample Amount:*	**1,0000**

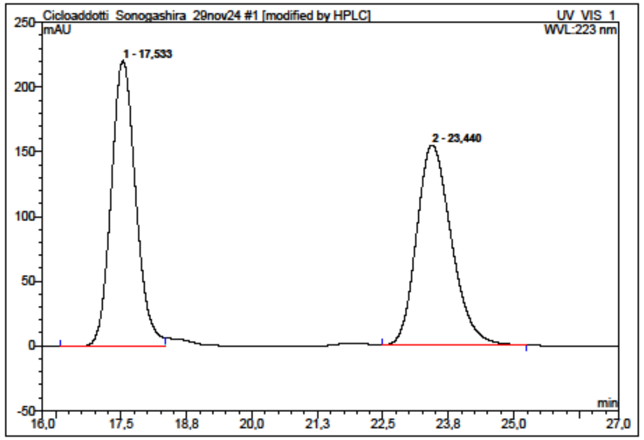

No.	Ret.Time min	Peak Name	Height mAU	Area mAU*min	Rel.Area %	Amount	Type
1	17,53	n.a.	220,548	119,206	50,00	n.a.	BM *
2	23,44	n.a.	154,791	119,205	50,00	n.a.	BMB
Total:			375,339	238,411	100,00	0,000	

default/Integration

Chromeleon (c) Dionex 1996-2006
Version 6.80 SR9 Build 2673 (161349)

Operator:HPLC Timebase:HPLC2 Sequence:Cicloaddotti_Sonogashira_29nov24 Page 1-1
29/11/2024 3.39 PM

2 DSpc1304 chir alfaT Me 5mM

Sample Name:	**DSpc1304 chir alfaT Me 5mM**	*Injection Volume:*	**2,0**
Vial Number:	**BB2**	*Channel:*	**UV_VIS_1**
Sample Type:	**unknown**	*Wavelength:*	**223**
Control Program:	**Iso_50IPA_50Hex**	*Bandwidth:*	**n.a.**
Quantif. Method:	**INSTRUMENTS_IQ_Iso17D**	*Dilution Factor:*	**1,0000**
Recording Time:	**29/11/2024 14.49**	*Sample Weight:*	**1,0000**
Run Time (min):	**30,00**	*Sample Amount:*	**1,0000**

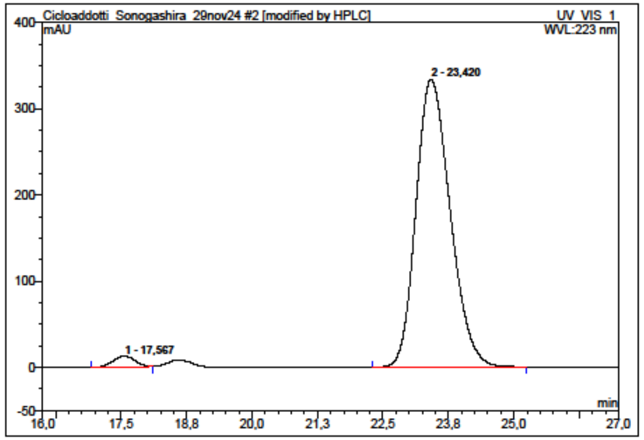

No.	Ret.Time min	Peak Name	Height mAU	Area mAU*min	Rel.Area %	Amount	Type
1	17,57	n.a.	12,394	6,016	2,33	n.a.	BMB
2	23,42	n.a.	333,889	252,609	97,67	n.a.	BMB
Total:			346,283	258,625	100,00	0,000	

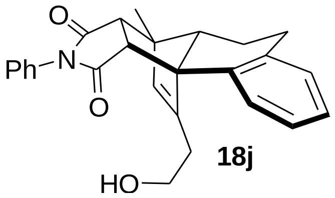

18j

default/Integration

Chromeleon (c) Dionex 1996-2006
Version 6.80 SR9 Build 2673 (161349)

HPLC analysis of compound racemic (left) and chiral (right) compound **18j**.

Operator:HPLC Timebase:HPLC2 Sequence:Cicloaddotti_alfaTPent_02apr25 Page 1-1
2/4/2025 3.39 PM

7 DSpc1316 alfaT Pent rac 5 mM

Sample Name:	DSpc1316 alfaT Pent rac 5 mM	*Injection Volume:*	2,0
Vial Number:	BC1	*Channel:*	UV_VIS_1
Sample Type:	unknown	*Wavelength:*	223
Control Program:	Iso_30IPA_40min	*Bandwidth:*	n.a.
Quantif. Method:	INSTRUMENTS_IQ_Iso17D	*Dilution Factor:*	1,0000
Recording Time:	2/4/2025 14.09	*Sample Weight:*	1,0000
Run Time (min):	50,00	*Sample Amount:*	1,0000

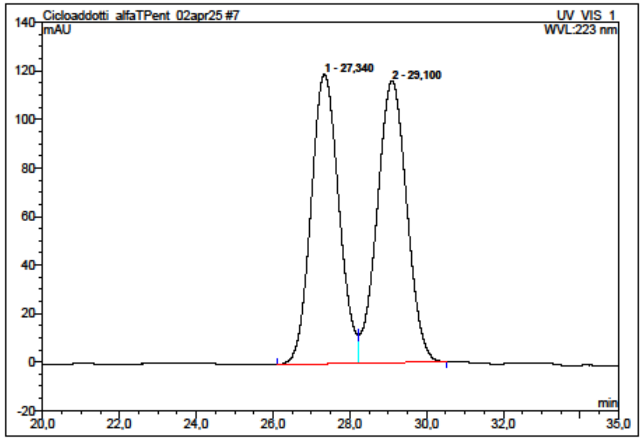

No.	Ret.Time min	Peak Name	Height mAU	Area mAU*min	Rel.Area %	Amount	Type
1	27,34	n.a.	119,421	99,995	49,09	n.a.	BM
2	29,10	n.a.	116,216	103,685	50,91	n.a.	MB
Total:			235,637	203,680	100,00	0,000	

default/Integration

Chromeleon (c) Dionex 1996-2006
Version 6.80 SR9 Build 2673 (161349)

Operator:HPLC Timebase:HPLC2 Sequence:Cicloaddotti_alfaTPent_02apr25 Page 1-1
2/4/2025 3.42 PM

8 DSpc1312 alfaT Pent chir 5 mM

Sample Name:	DSpc1312 alfaT Pent chir 5 mM	*Injection Volume:*	2,0
Vial Number:	BC2	*Channel:*	UV_VIS_1
Sample Type:	unknown	*Wavelength:*	223
Control Program:	Iso_30IPA	*Bandwidth:*	n.a.
Quantif. Method:	INSTRUMENTS_IQ_Iso17D	*Dilution Factor:*	1,0000
Recording Time:	2/4/2025 14.59	*Sample Weight:*	1,0000
Run Time (min):	40,00	*Sample Amount:*	1,0000

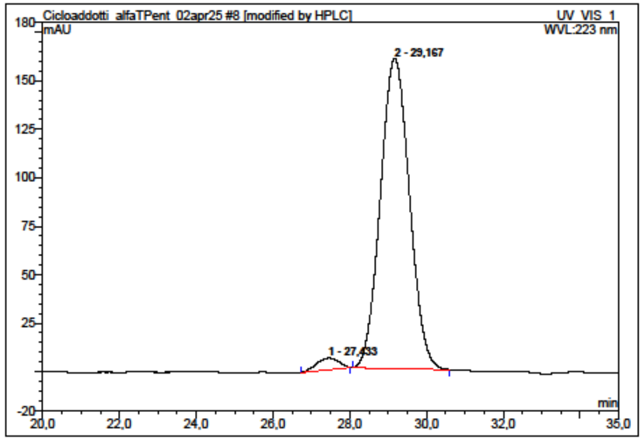

No.	Ret.Time min	Peak Name	Height mAU	Area mAU*min	Rel.Area %	Amount	Type
1	27,43	n.a.	6,062	4,086	2,83	n.a.	BMB*
2	29,17	n.a.	159,886	140,194	97,17	n.a.	BMB
Total:			165,949	144,280	100,00	0,000	

default/Integration

Chromeleon (c) Dionex 1996-2006
Version 6.80 SR9 Build 2673 (161349)

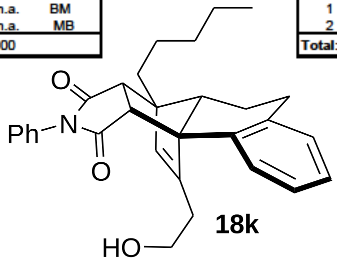

18k

HPLC analysis of compound racemic (left) and chiral (right) compound **18k**.

Operator:HPLC Timebase:HPLC2 Sequence:Cicloaddotti_Sonogashira_12dic24 Page 1-1
12/12/2024 4.51 PM

1 DSpc1327 rac C5 pent 5mM

Sample Name:	DSpc1327 rac C5 pent 5mM	*Injection Volume:*	2,0
Vial Number:	BA2	*Channel:*	UV_VIS_1
Sample Type:	unknown	*Wavelength:*	223
Control Program:	Iso_50IPA_50Hex	*Bandwidth:*	n.a.
Quantif. Method:	INSTRUMENTS_IQ_Iso17D	*Dilution Factor:*	1,0000
Recording Time:	12/12/2024 16.10	*Sample Weight:*	1,0000
Run Time (min):	30,00	*Sample Amount:*	1,0000

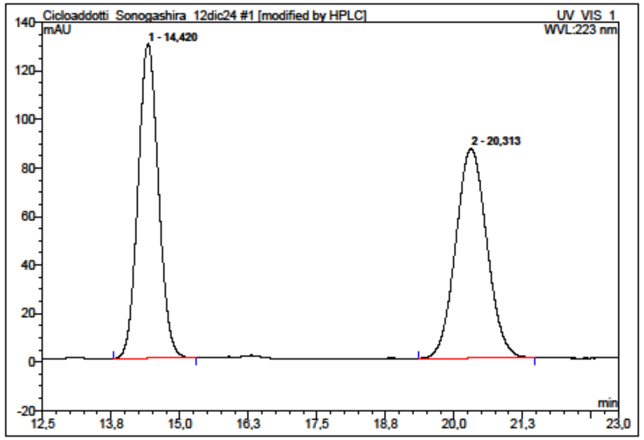

No.	Ret.Time min	Peak Name	Height mAU	Area mAU*min	Rel.Area %	Amount	Type
1	14,42	n.a.	129,606	55,744	49,49	n.a.	BMB
2	20,31	n.a.	86,589	56,897	50,51	n.a.	BMB
Total:			216,196	112,641	100,00	0,000	

default/Integration

Chromeleon (c) Dionex 1996-2006
Version 6.80 SR9 Build 2673 (161349)

Operator:HPLC Timebase:HPLC2 Sequence:Cicloaddotti_Sonogashira_12dic24 Page 1-1
12/12/2024 5.12 PM

2 DSpc1321 chir C5 pent 5mM

Sample Name:	DSpc1321 chir C5 pent 5mM	*Injection Volume:*	2,0
Vial Number:	BA3	*Channel:*	UV_VIS_1
Sample Type:	unknown	*Wavelength:*	223
Control Program:	Iso_50IPA_50Hex	*Bandwidth:*	n.a.
Quantif. Method:	INSTRUMENTS_IQ_Iso17D	*Dilution Factor:*	1,0000
Recording Time:	12/12/2024 16.41	*Sample Weight:*	1,0000
Run Time (min):	30,00	*Sample Amount:*	1,0000

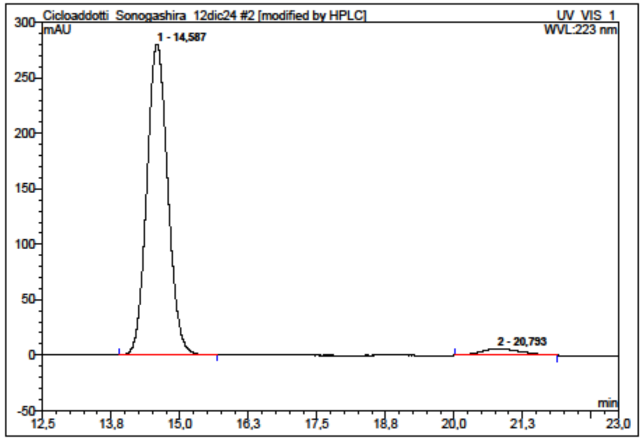

No.	Ret.Time min	Peak Name	Height mAU	Area mAU*min	Rel.Area %	Amount	Type
1	14,59	n.a.	280,404	123,787	96,10	n.a.	BMB
2	20,79	n.a.	6,091	5,017	3,90	n.a.	BMB
Total:			286,495	128,804	100,00	0,000	

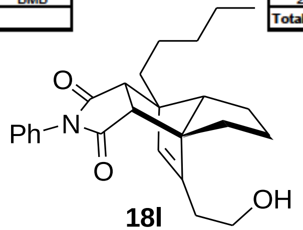

18l

default/Integration

Chromeleon (c) Dionex 1996-2006
Version 6.80 SR9 Build 2673 (161349)

HPLC analysis of compound racemic (left) and chiral (right) compound **18l**.

3. Determination of the absolute configuration of diene 15a.

3.1. Synthesis of compound 16.

To a solution of *t*$Bu_3PAuNTF_2$ (2 mol %) in DCM (3.8 mL) stirred at 25 °C under a nitrogen atmosphere was added a solution of propargyl vinyl ether (*S*)-**14a** (68 mg, 0.39 mmol) in DCM (3.8 mL; final concentration, 0.05 M), and the reaction mixture was stirred at 25 °C until complete consumption of the starting material (TLC monitoring, 10 minutes). (*R*)-(+)-*N*-(1-phenylethyl)maleimide (82 μL, 0.46 mmol, 1.2 equiv.) was then added, and after 15 min, the mixture was diluted with MeOH (15 mL) and immediately added with $NaBH_4$ (15 mg, 0.39 mmol). After 10 min, the reduction was complete. The solvent was then evaporated, water added to the residue (15 mL), and the product extracted with DCM (3 × 10 mL). The combined organic extracts were washed with brine (20 mL) and dried over anhydrous Na_2SO_4. After filtration and evaporation of the solvent, the crude oil was purified by flash chromatography (eluent: *n*-hexane/EtOAc, 4:1; R_f = 0.21) to afford compound **16** (133 mg, 91%), that contained a 3:1 mixture of *endo*/*exo* isomers, as a white solid. Crystals of pure *endo*-**16** suitable for X-ray structure determination were obtained by slow evaporation of a diethyl ether solution; the same crystals were also used for full spectroscopic characterization.

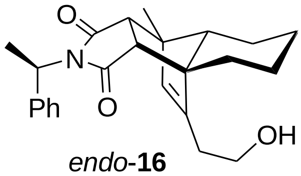

endo-**16**

$[\alpha]_D^{24}$ +4.7 (*c* 1.0, $CHCl_3$). M.p. 117.2 – 118.5 °C. ^{1}H NMR (400 MHz, $CDCl_3$) (8:1 mixture of diastereoisomers; major isomer reported) δ (ppm): 7.46 (d, *J* = 7.2 Hz, 2H), 7.32 – 7.24 (m, 3H), 5.24 (q, *J* = 7.6 Hz, 1H), 5.20 (s, 1H), 3.37 – 3.31 (m, 1H), 3.18 – 3.13 (m, 1H), 2.86 (s, 2H), 2.36 – 2.89 (m, 1H), 2.12 – 2.03 (m, 1H), 1.17 (d, *J* = 7.2 Hz, 3H), 1.67 – 1.55 (m, 2H), 1.47 – 1.29 (m, 6H), 1.16 – 0.94 (m, 4H). $^{13}C\{^1H\}$ NMR (100.4 MHz, $CDCl_3$) δ (ppm): 177.3, 177.0, 144.1, 139.8, 128.3 (2C), 128.22 (2C), 128.19, 127.6, 67.8, 60.1, 59.0, 55.5, 53.6, 52.8, 49.6, 30.3, 28.4, 23.8, 23.3, 21.6, 16.1, 15.3. MS (ESI) *m/z* (%): 781 ($[2M + Na]^+$, 19), 402 ($[M + Na]^+$, 100). Anal. Calcd for $C_{24}H_{29}NO_3$: C, 75.96; H, 7.70; N, 3.69. Found: C, 75.91; H, 7.32; N, 3.68.

3.2 Crystal structure determination of compound 16.

A single crystal was mounted in a loop and coated with a trace of silicone oil. Data collection were performed at 100 K with a Bruker Apex-II CCD diffractometer, using a Cu–Kα (λ = 1.54184 Å) radiation. Data were collected, reflections were indexed and processed, and the files scaled and corrected for absorption, using Bruker APEX2, SAINT and SADABS-2016/2 [1] routine. The integrated intensities, measured using the φ and ω scan mode, were corrected for Lorentz and polarization effects. Structures were solved by direct methods of SIR2019 [2], the refinement was performed using the full-matrix least squares on F^2 provided, within WinGX v.2013.3 routine [3], by SHELXL2018 [4].

Two crystallographically independent molecules are present in the asymmetric unit, in which all bond lengths are in normal ranges. Checked for P 21/m group with no result.

Data can be obtained free of charge from the Cambridge Crystallographic Data Centre via www.ccdc.cam.ac.uk/data_request/cif, with the deposition number **CCDC2424553**, that contains the supplementary crystallographic data for this structure.

Crystallographic data.

2x ($C_{24}H_{29}NO_3$), M=2x (379.48), Monoclinic, space group P 21

a=6.6050(6), *b*=14.850(1), *c*=20.563(2)Å, β=93.518(5), V=2013.1(4)$Å^3$, Z=2, D_c=1.252, μ=0.648mm^{-1}, F (000) = 816.

25274 reflections were collected with a 2.153<θ< 69.054 range with a completeness to theta 97.1%; 7005 were unique, the parameters were 505 and the final R index was 0.0498 for reflections having I>2σI.

A colourless prismatic crystal (0.150x0.250x0.450) was used for data collection.

Hydrogen atoms were all assigned in calculated positions as riding atoms.

Non-hydrogen atoms were refined anisotropically whereas hydrogen atoms were refined as isotropic.

References Section 3

[1] *APEX2, SAINT and SADABS*. Bruker AXS Inc., Madison, Wisconsin, USA, 2009.

[2] Burla, M.C.; Caliandro, R.; Carrozzini, B.; Cascarano, G. L.; Cuocci, C.; Giacovazzo, C.; Mallamo, M.; Mazzone, A.; Polidori, G. Crystal structure determination and refinement via SIR2014. *J. Appl. Cryst.* **2015**, *48*, 306–309.

[3] Farrugia, L. J. WinGX and ORTEP for Windows: an update. *J. Appl. Cryst*. **2012**, *45*, 849–854.

[4] Sheldrick, G.M. *SHELXL-2018, Program for Crystal Structure Refinement*. University of Göttingen, Göttingen 2018.

Table S2. Crystal data and structure refinement for **16**.

Identification code	GT29
Empirical formula	$C_{48}H_{58}N_2O_6$
Formula weight	758.96
Temperature	100(2) K
Wavelength	1.54178 A
Crystal system, space group	Monoclinic, P 21
Unit cell dimensions	a = 6.6050(7)Å α = 90 °
	b = 14.850(1)Å β = 93.518(5) °
	c = 20.563(2)Å γ = 90 °
Volume	2013.1(3) Å^3
Z, Calculated density	2, 1.252 Mg/m^3
Absorption coefficient	0.648 mm^{-1}
F(000)	816
Crystal size	0.400 x 0.250 x 0.150 mm
Theta range for data collection	2.153 to 69.054 °
Limiting indices	$-8 \leq h \leq 7$, $-17 \leq k \leq 17$, $-24 \leq l \leq 24$
Reflections collected / unique	25274 / 7005 [R(int) = 0.0610]
Completeness to theta = 67.679	97.1 %
Refinement method	Full-matrix least-squares on F^2
Data / restraints / parameters	7005 / 1 / 505
Goodness-of-fit on F^2	1.153
Final R indices [I > 2σ(I)]	R1 = 0.0498, wR2 = 0.1337
R indices (all data)	R1 = 0.0596, wR2 = 0.1547
Absolute structure parameter	0.17(9)
Extinction coefficient	n/a
Largest diff. peak and hole	0.374 and -0.415 e.Å^{-3}

3.3. ORTEP drawing of compound 16.

Crystals were obtained from diethyl ether (slow evaporation).

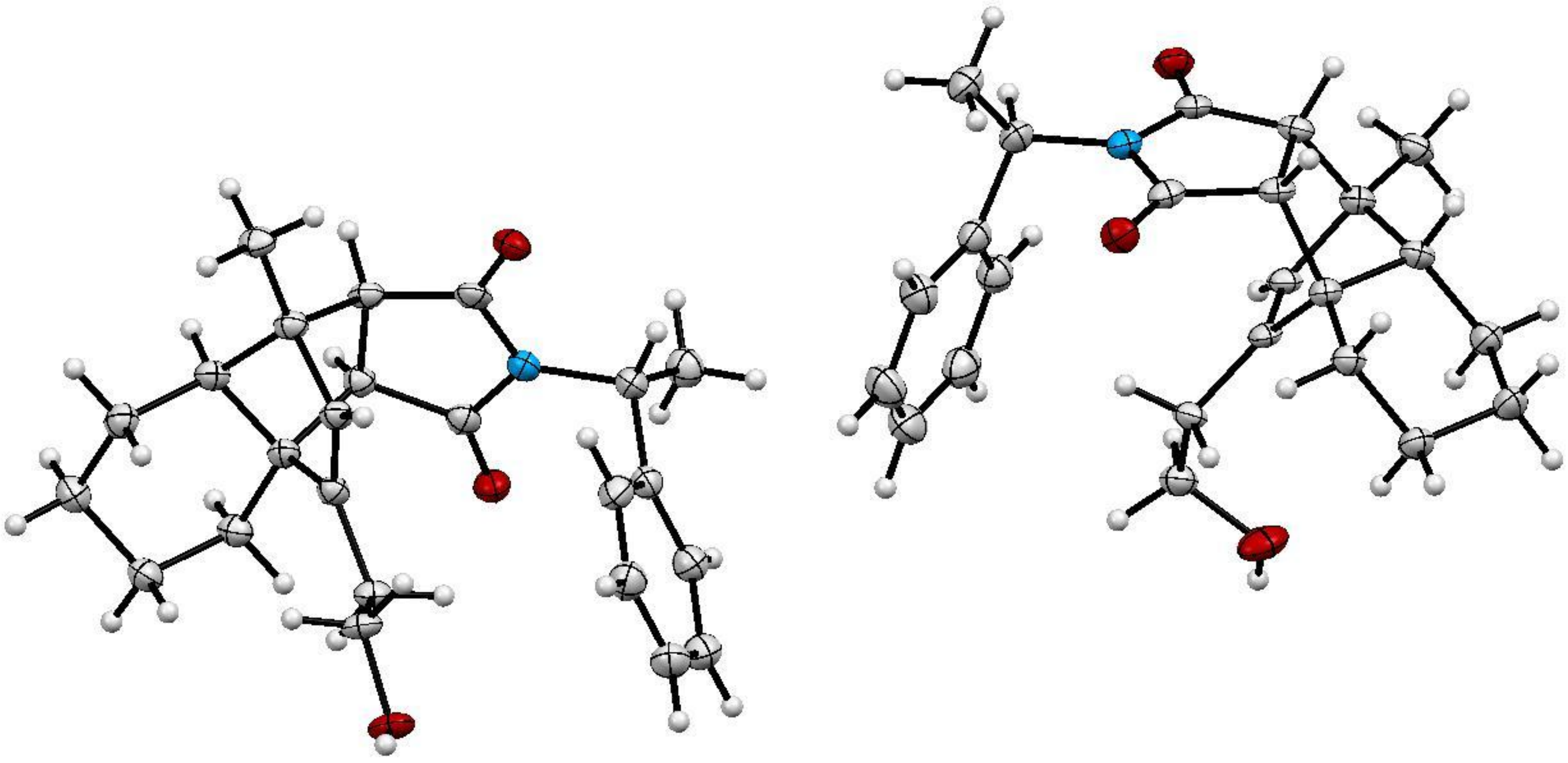

Figure S1. Thermal ellipsoid drawing of compound **16** (50% probability).

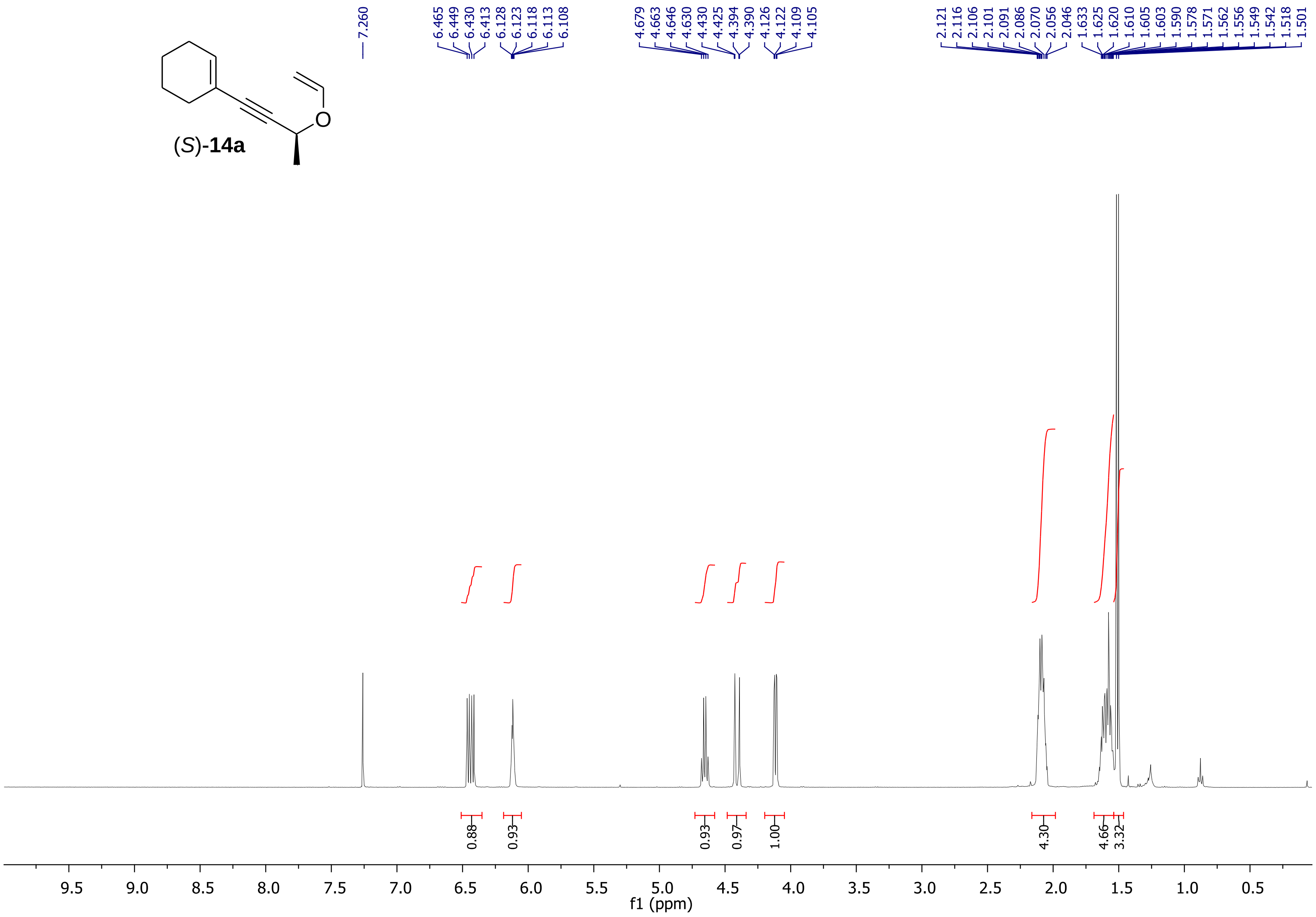

^{1}H NMR ($CDCl_3$, 400 MHz) of compound (*S*)-**14a**

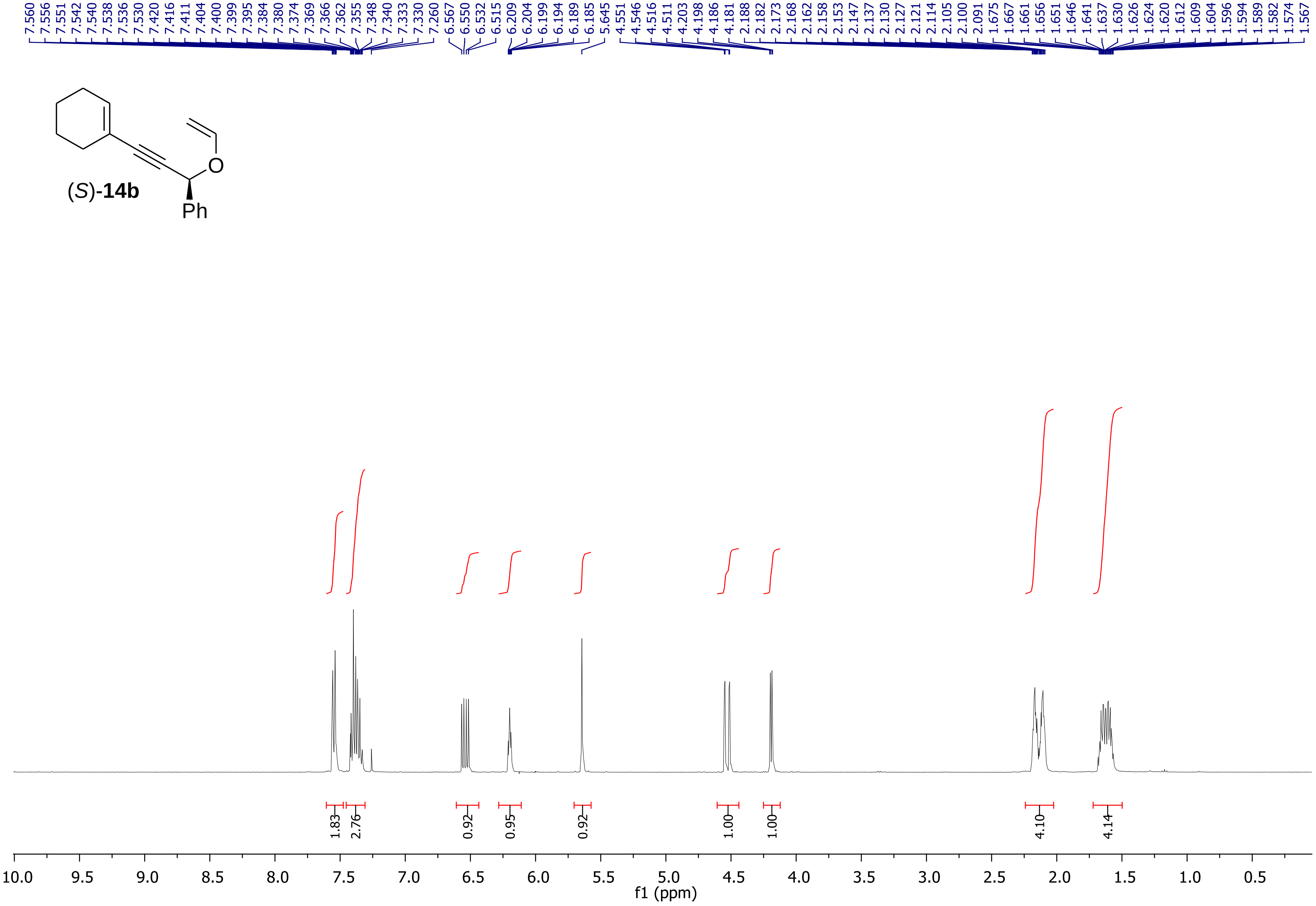

^{1}H NMR ($CDCl_3$, 400 MHz) of compound (*S*)-**14b**

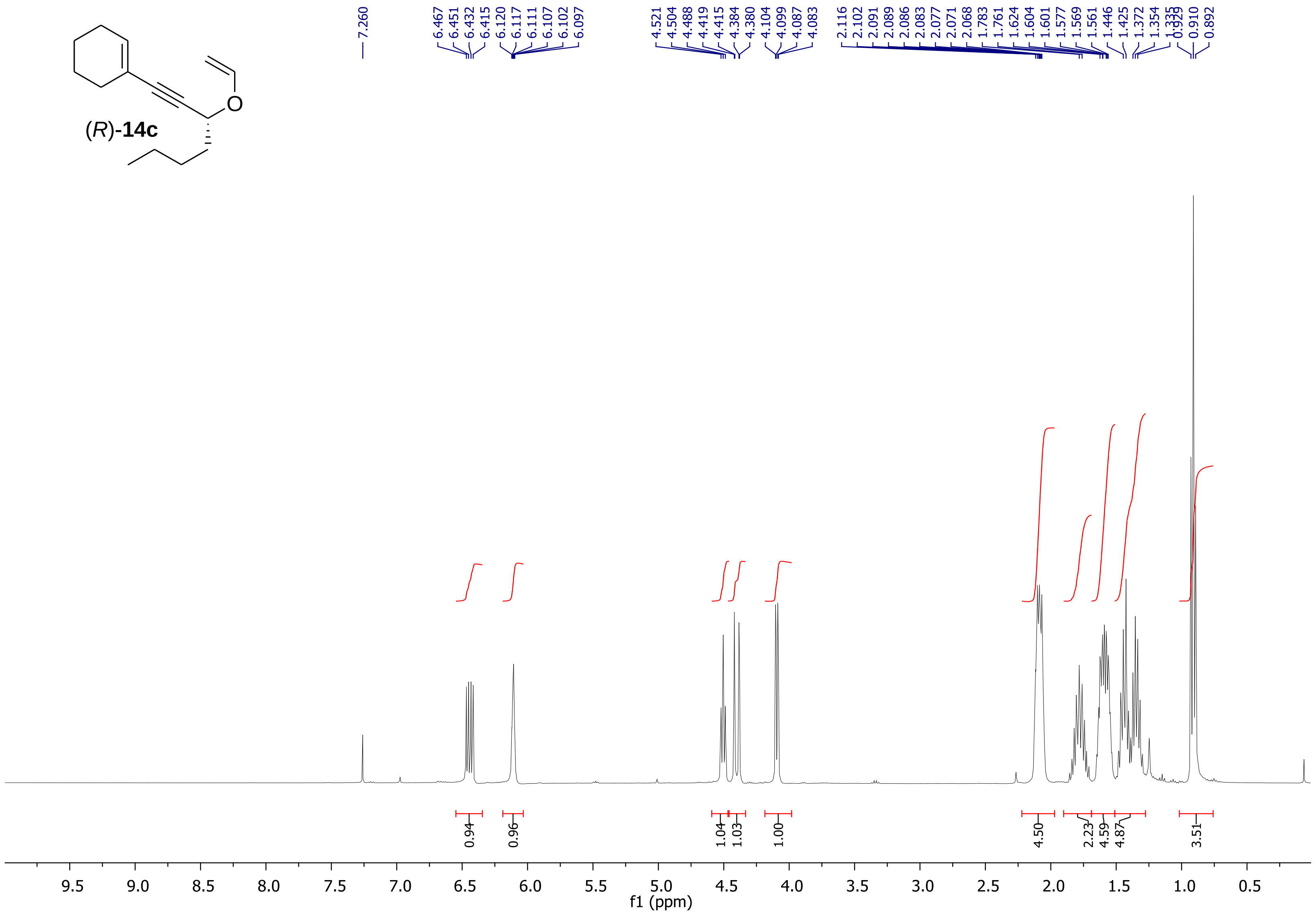

^{1}H NMR ($CDCl_3$, 400 MHz) of compound (*R*)-**14c**

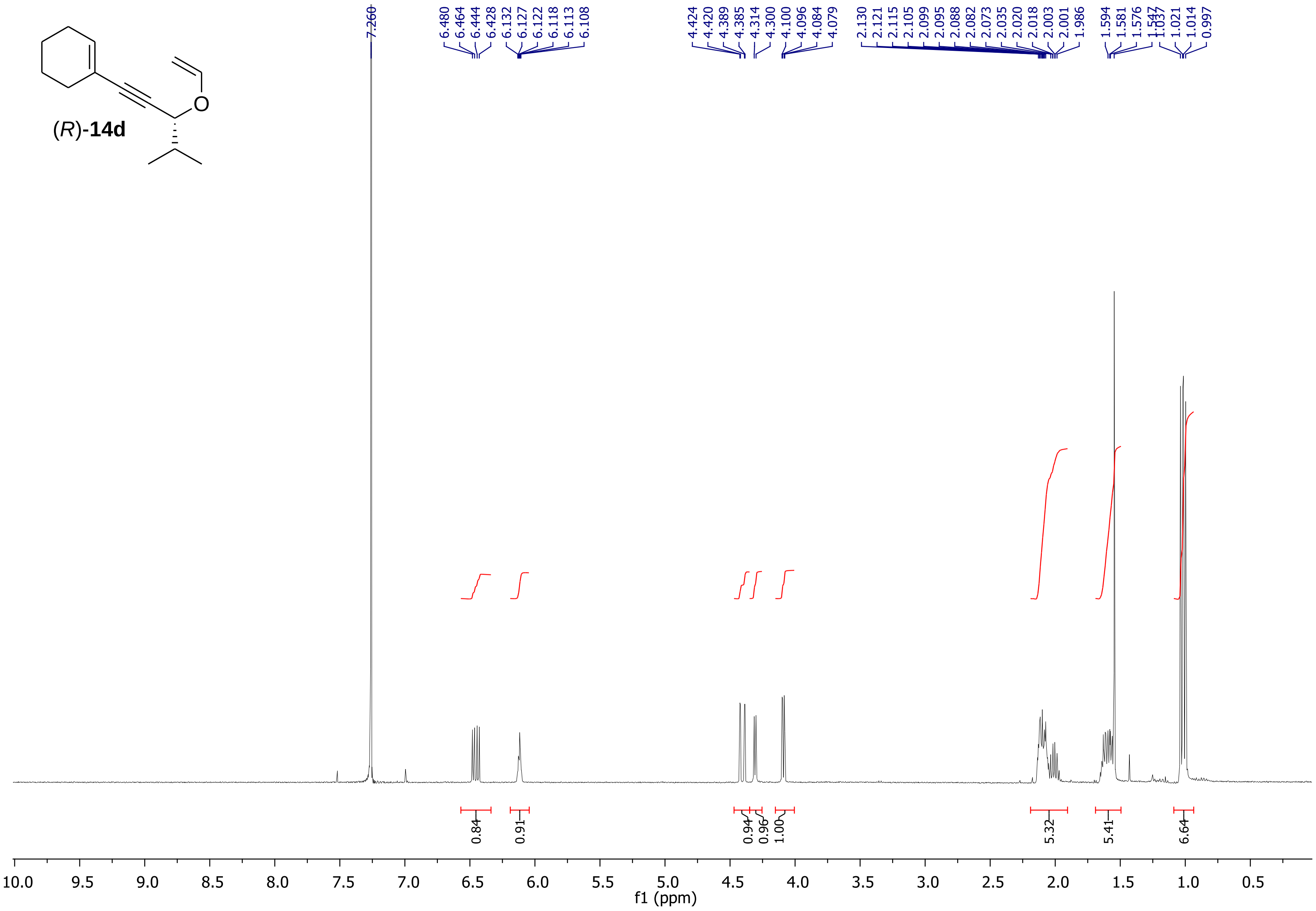

^{1}H NMR ($CDCl_3$, 400 MHz) of compound (*R*)-**14d**

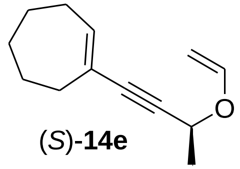

(*S*)-**14e**

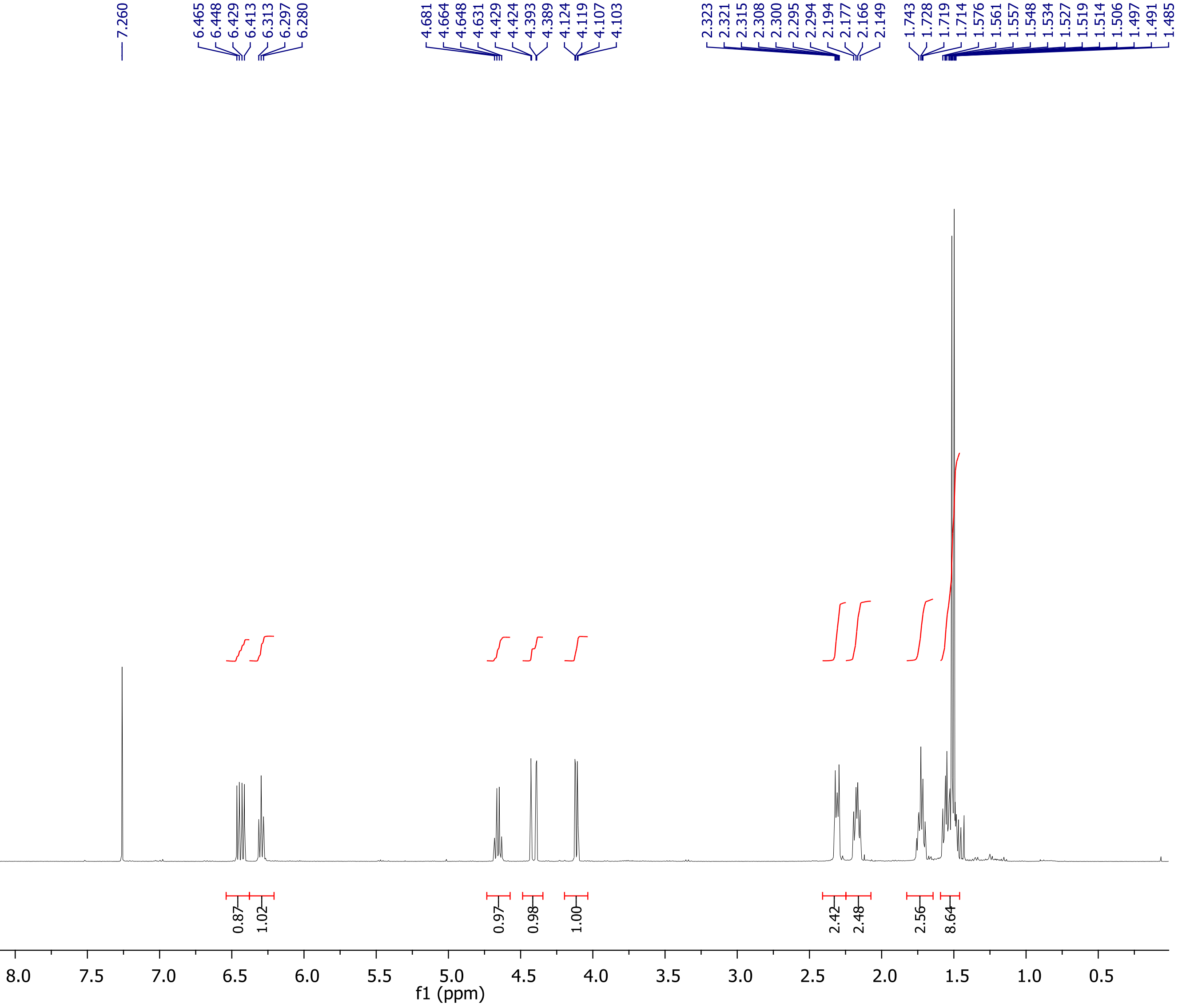

^{1}H NMR ($CDCl_3$, 400 MHz) of compound (*S*)-**14e**

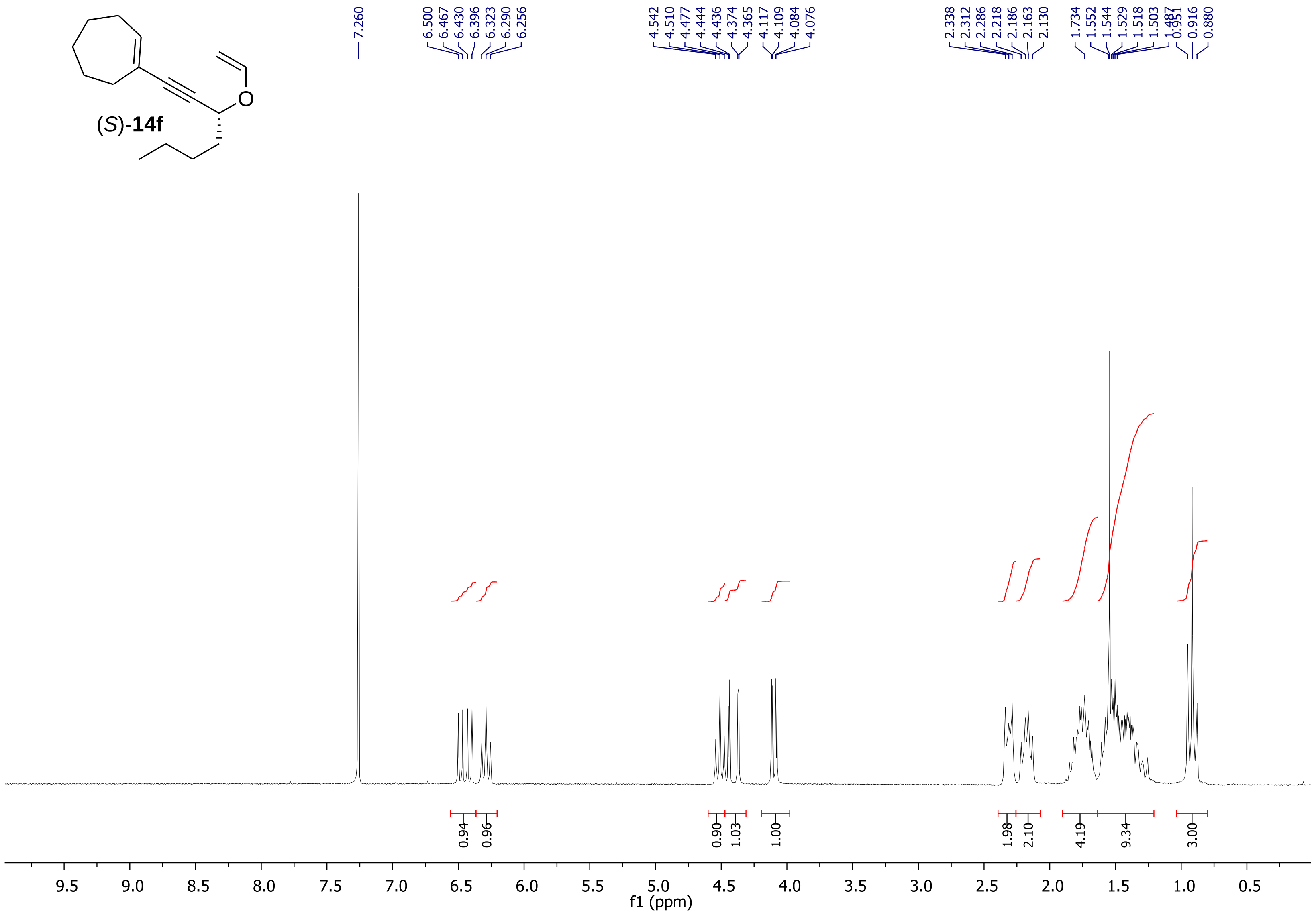

^{1}H NMR ($CDCl_3$, 200 MHz) of compound (*S*)-**14f**

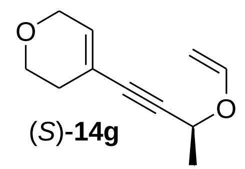

(*S*)-**14g**

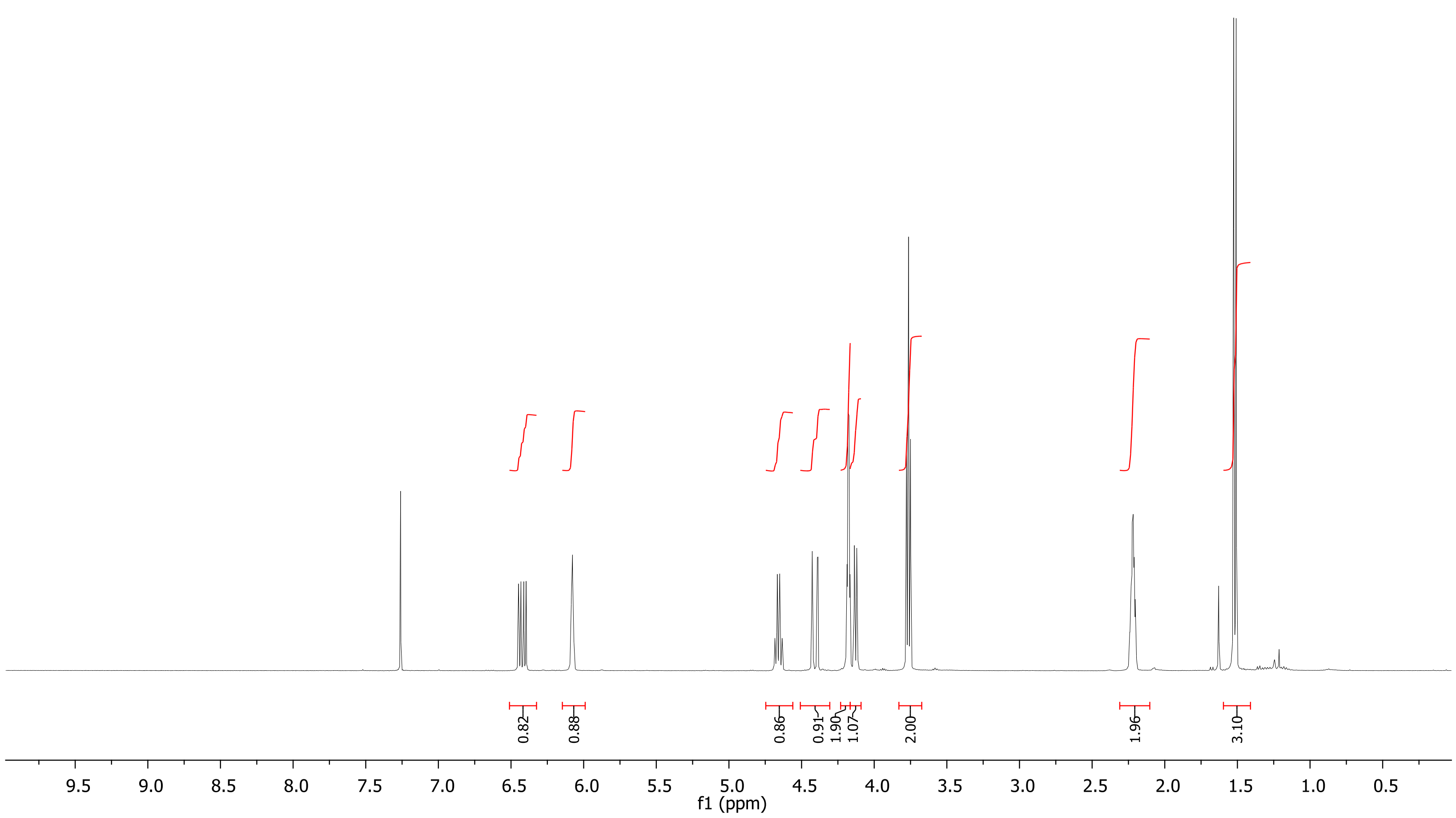

^{1}H NMR ($CDCl_3$, 400 MHz) of compound (*S*)-**14g**

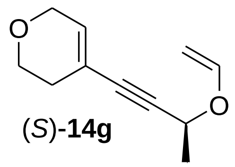

(*S*)-**14g**

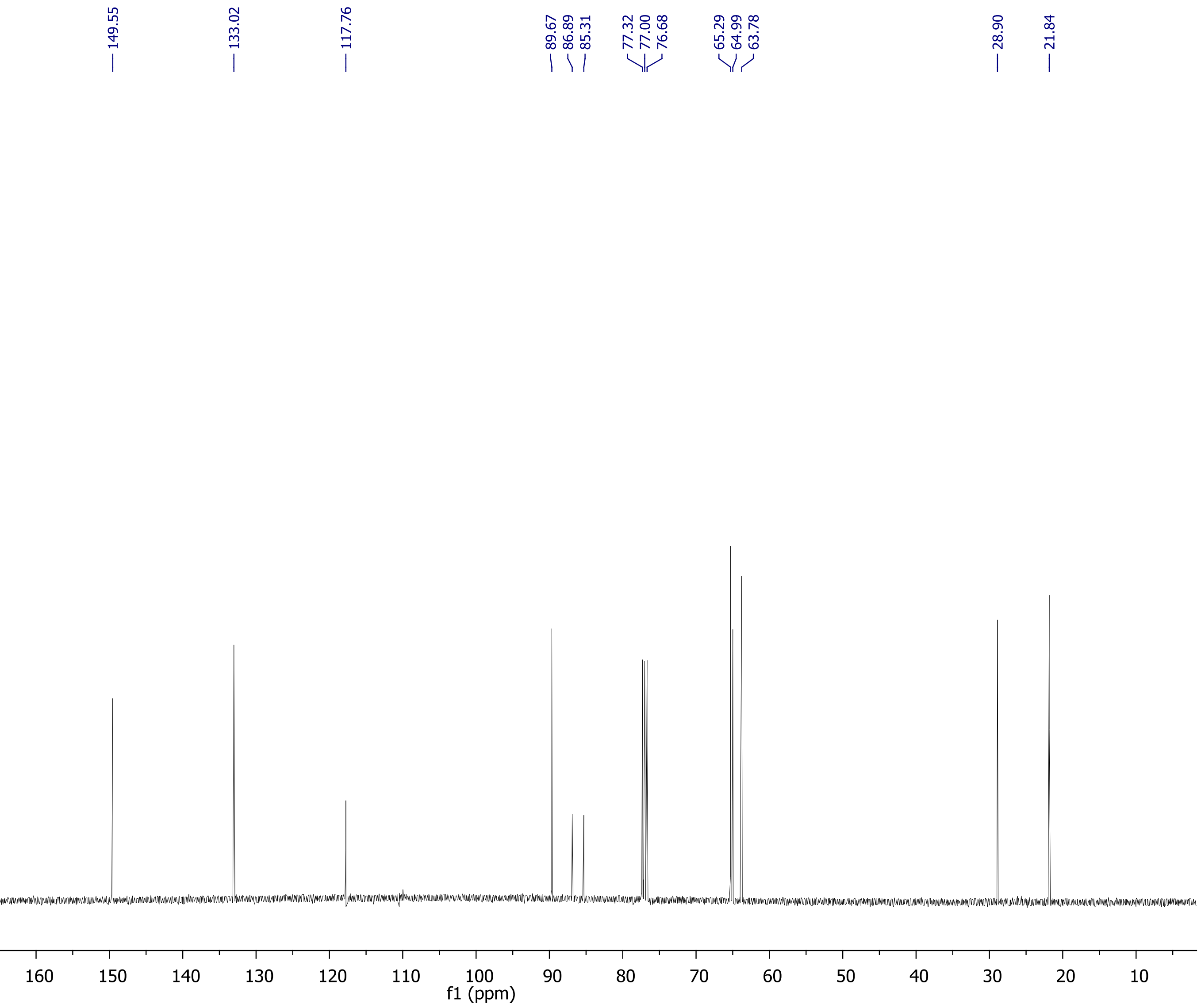

$^{13}C\{^{1}H\}$ NMR ($CDCl_3$, 100.4 MHz) of compound (*S*)-**14g**

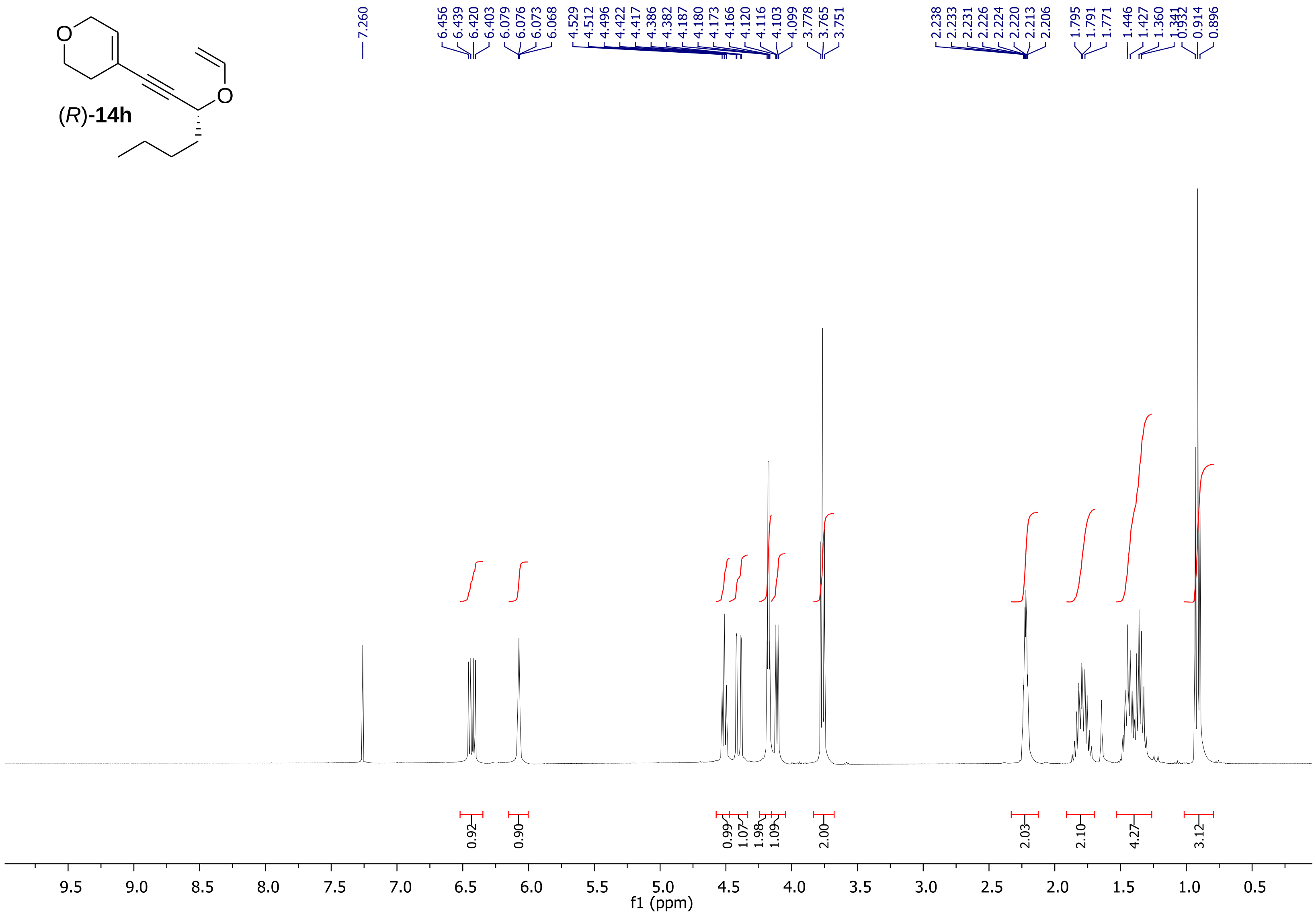

^{1}H NMR ($CDCl_3$, 400 MHz) of compound (*R*)-**14h**

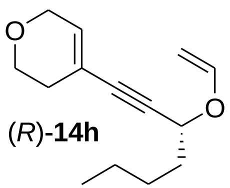

(*R*)-**14h**

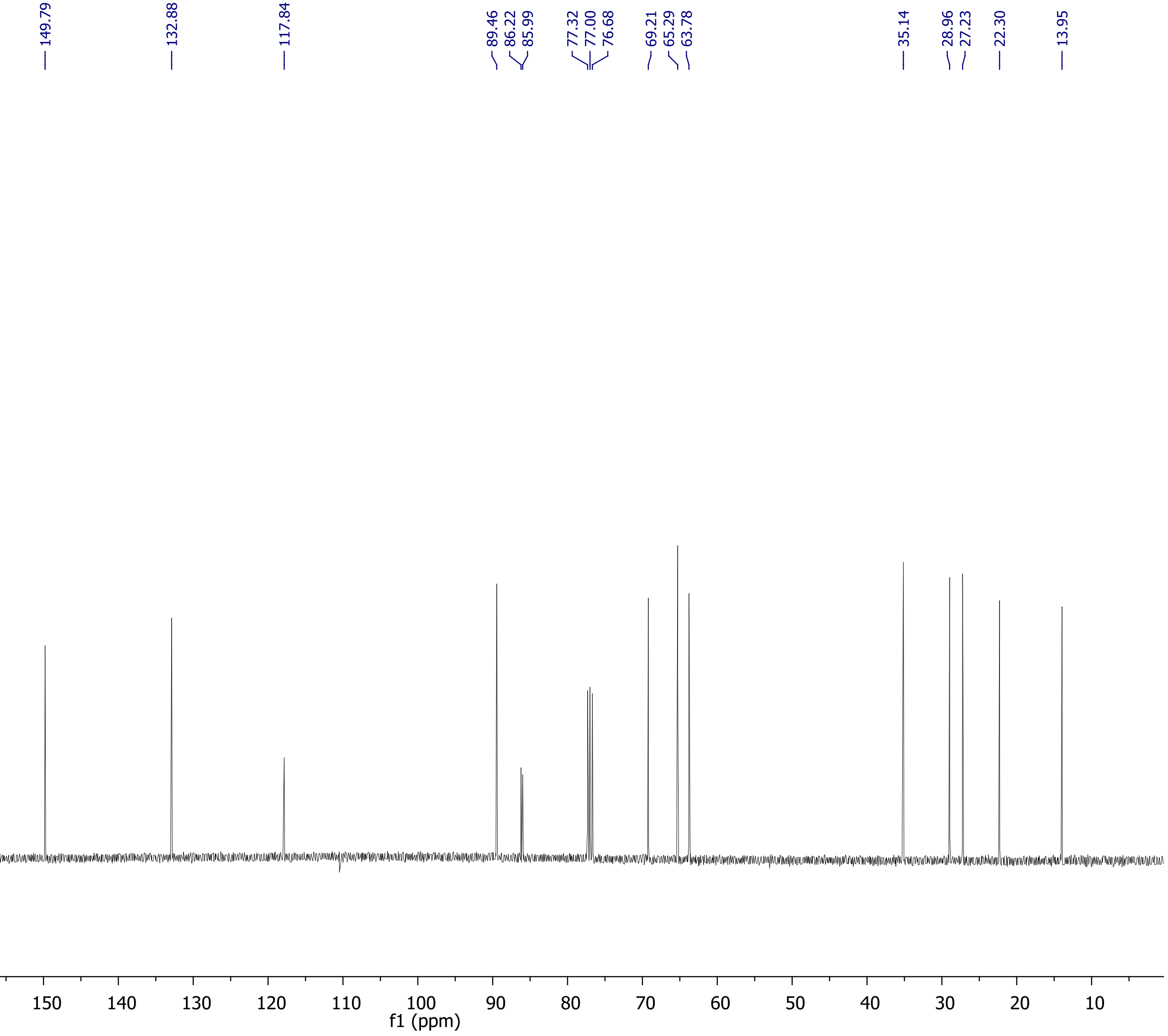

$^{13}C\{^{1}H\}$ NMR ($CDCl_3$, 100.4 MHz) of compound (*R*)-**14h**

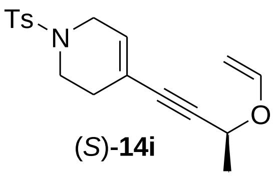

(*S*)-**14i**

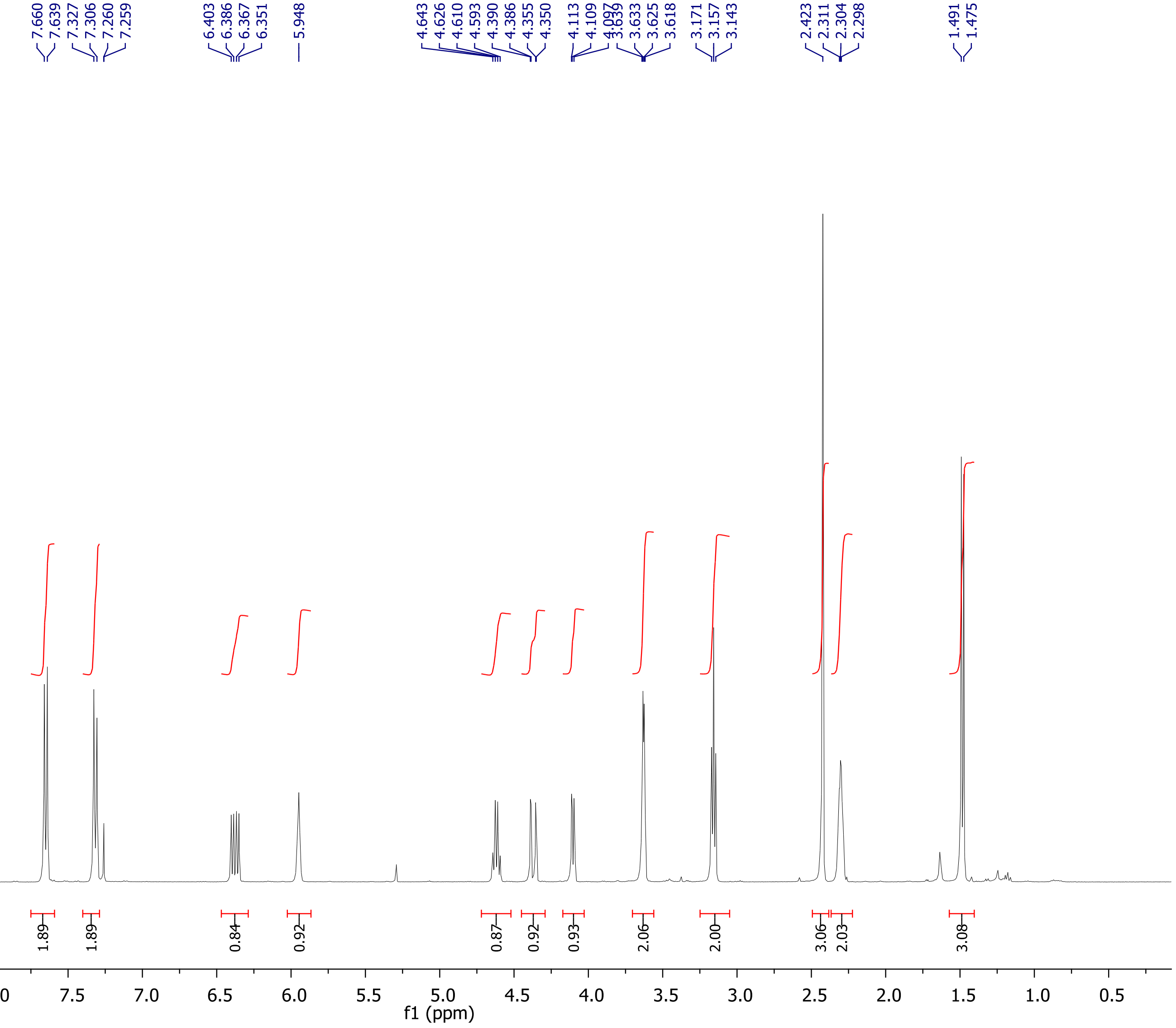

^{1}H NMR ($CDCl_3$, 400 MHz) of compound (*S*)-**14i**

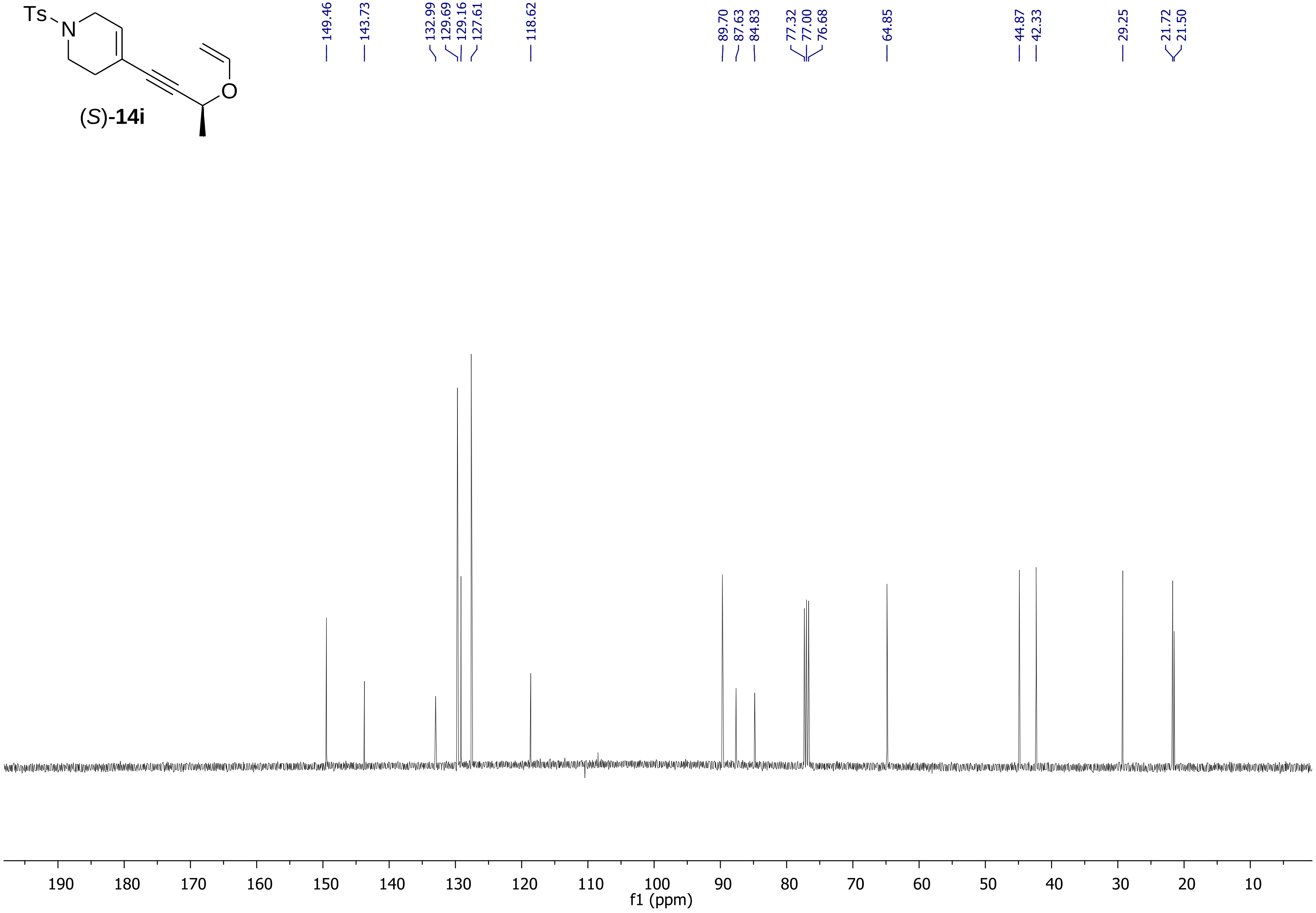

$^{13}C\{^1H\}$ NMR ($CDCl_3$, 100.4 MHz) of compound (*S*)-**14i**

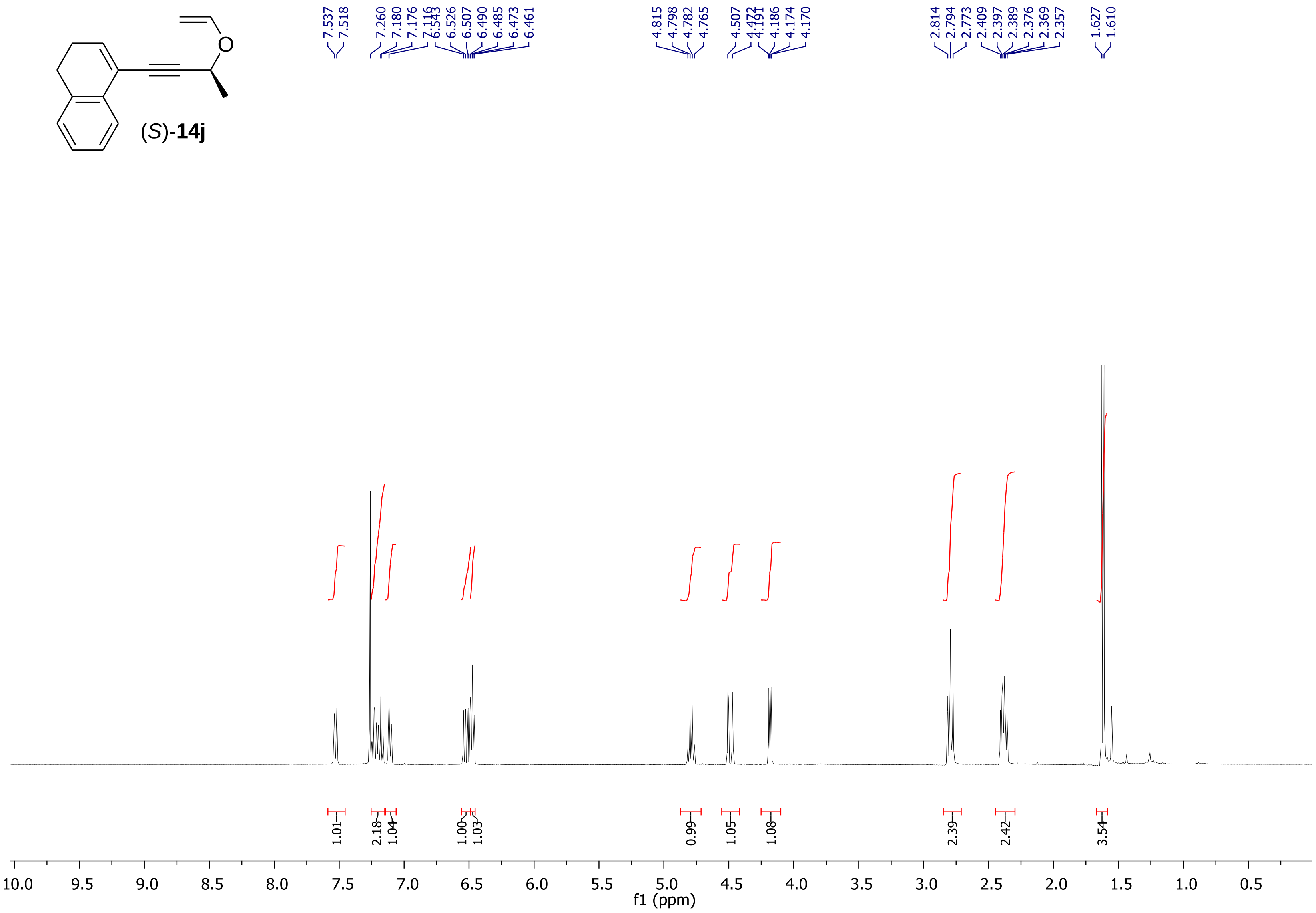

^{1}H NMR ($CDCl_3$, 400 MHz) of compound (*S*)-**14j**

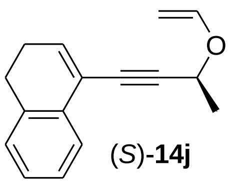

(*S*)-**14j**

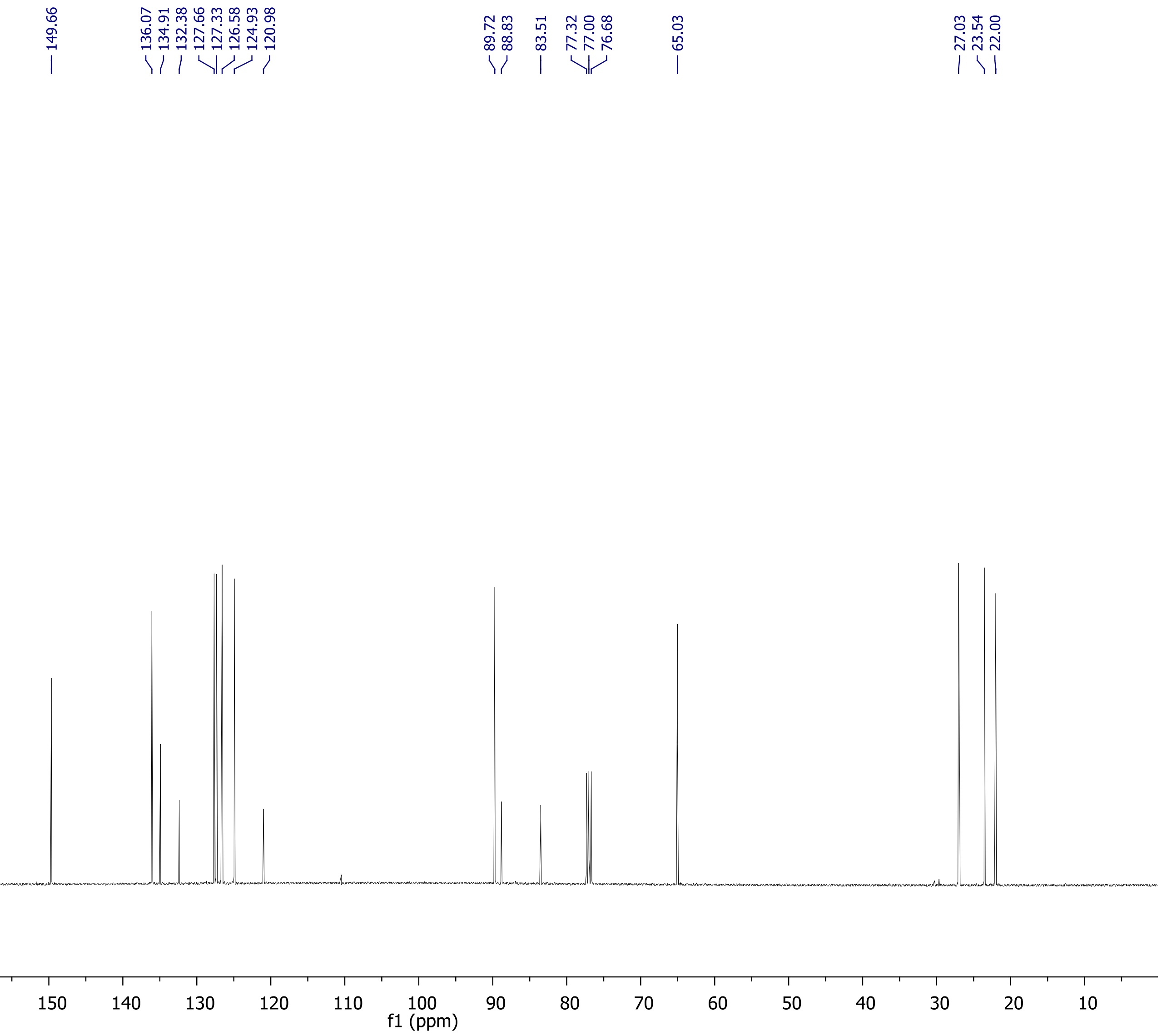

$^{13}C\{^{1}H\}$ NMR ($CDCl_3$, 100.4 MHz) of compound (*S*)-**14j**

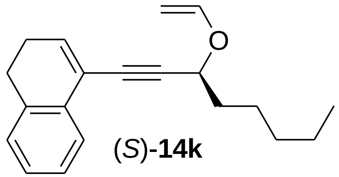

(*S*)-**14k**

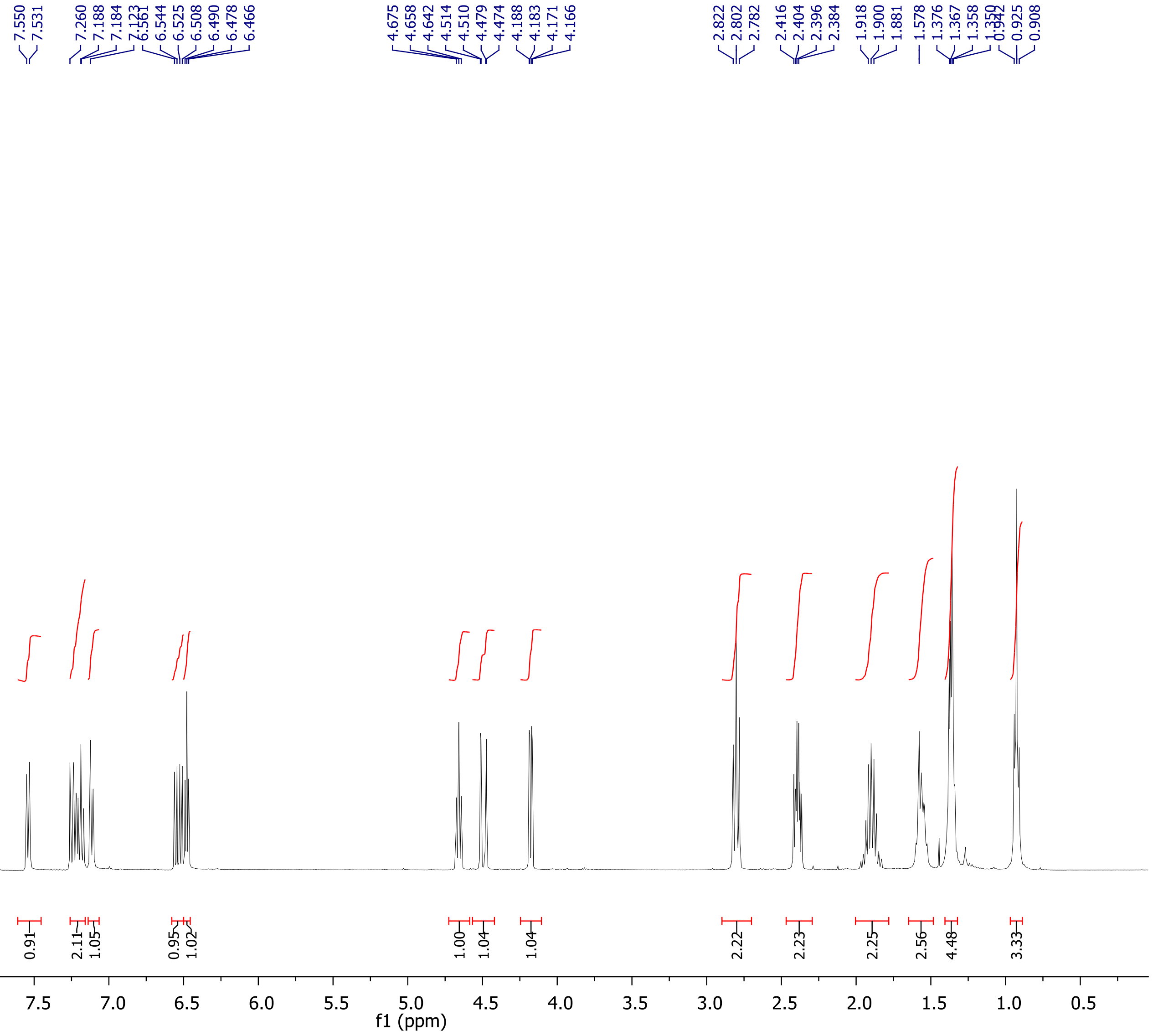

^{1}H NMR ($CDCl_3$, 400 MHz) of compound (*S*)-**14k**

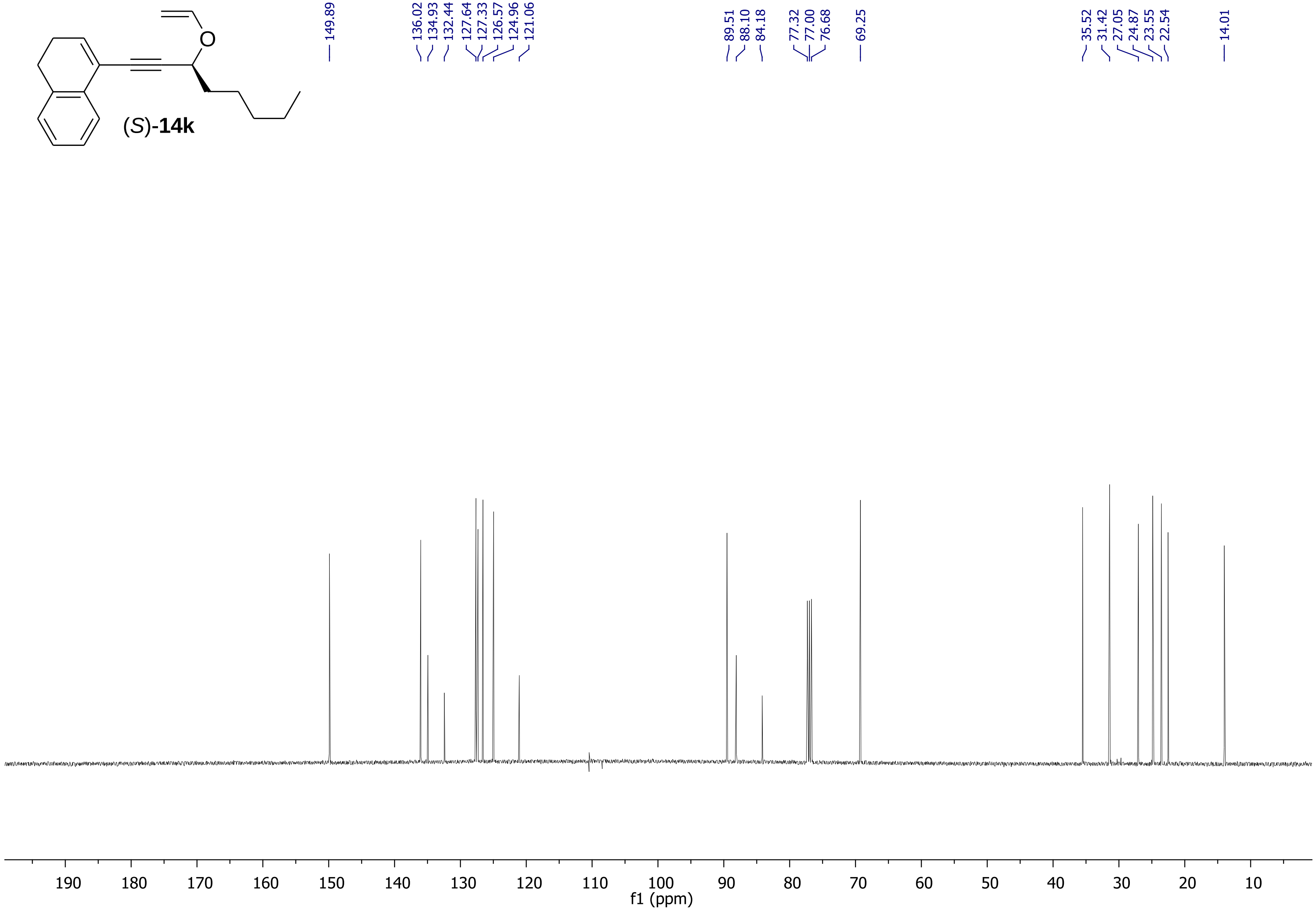

$^{13}C\{^{1}H\}$ NMR ($CDCl_3$, 100.4 MHz) of compound (*S*)-**14k**

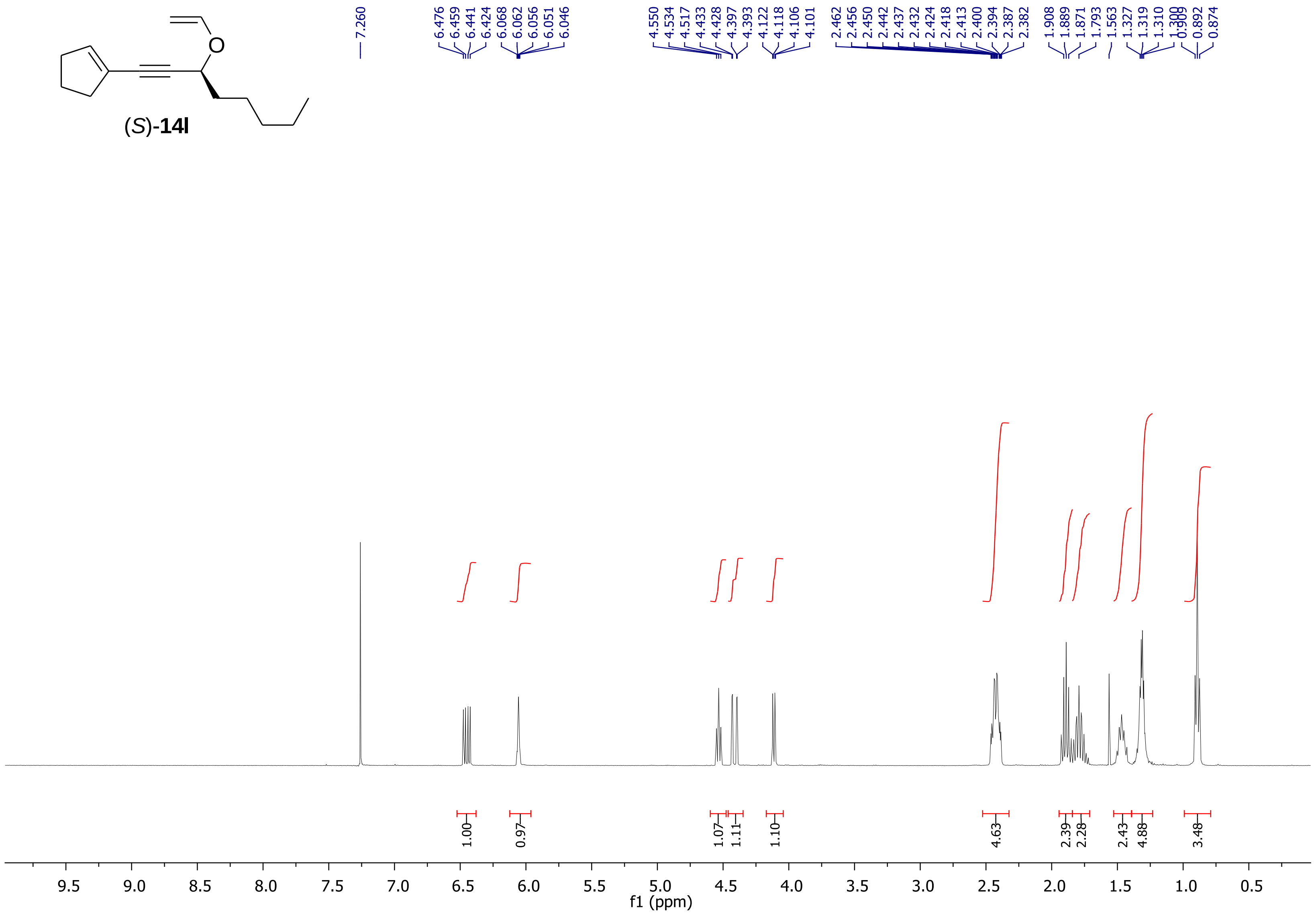

^{1}H NMR ($CDCl_3$, 400 MHz) of compound (*S*)-**14l**

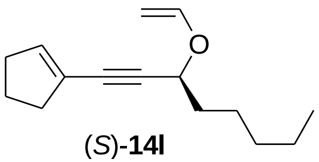

(*S*)-**14l**

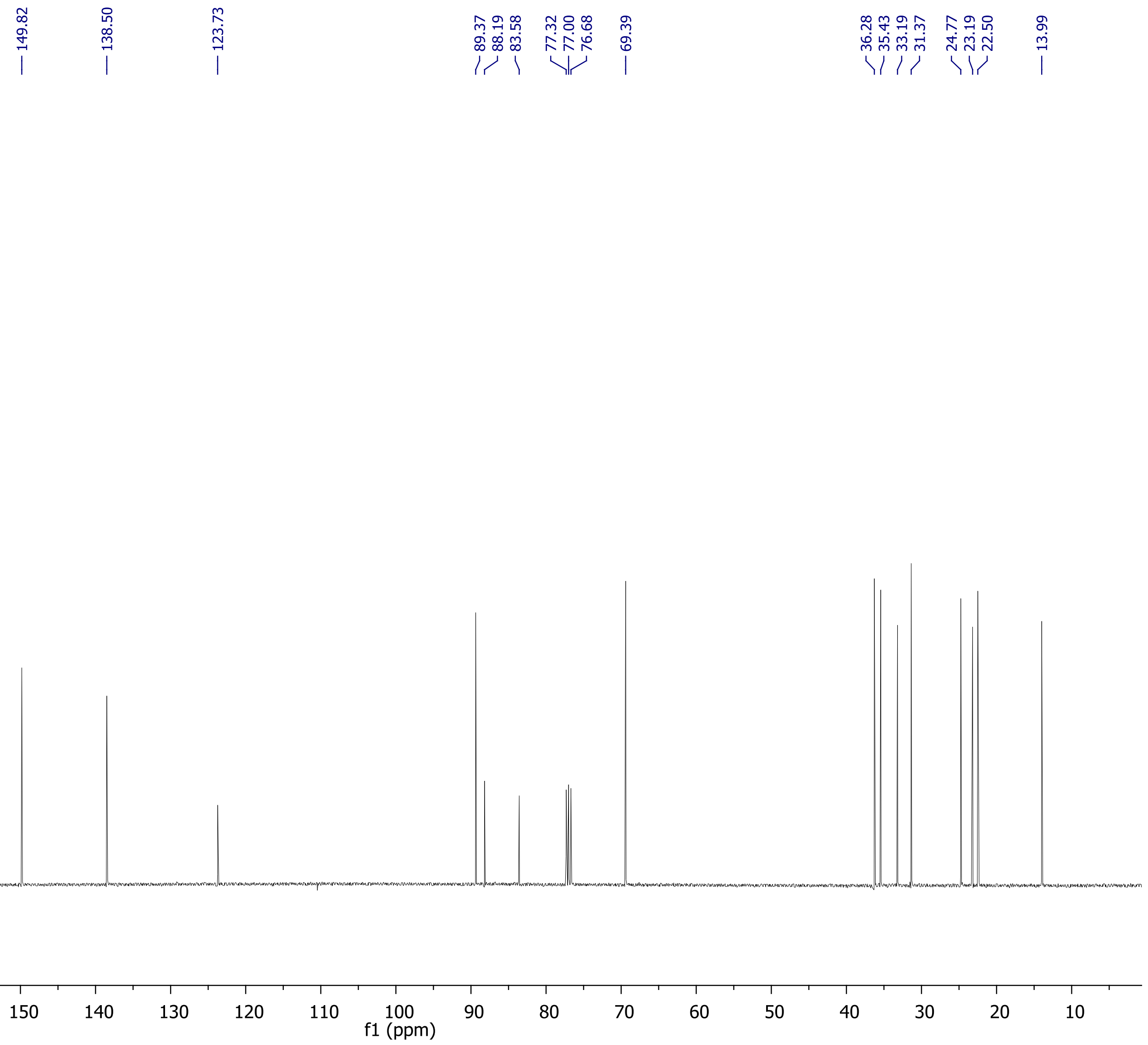

$^{13}C\{^{1}H\}$ NMR ($CDCl_3$, 100.4 MHz) of compound (*S*)-**14l**

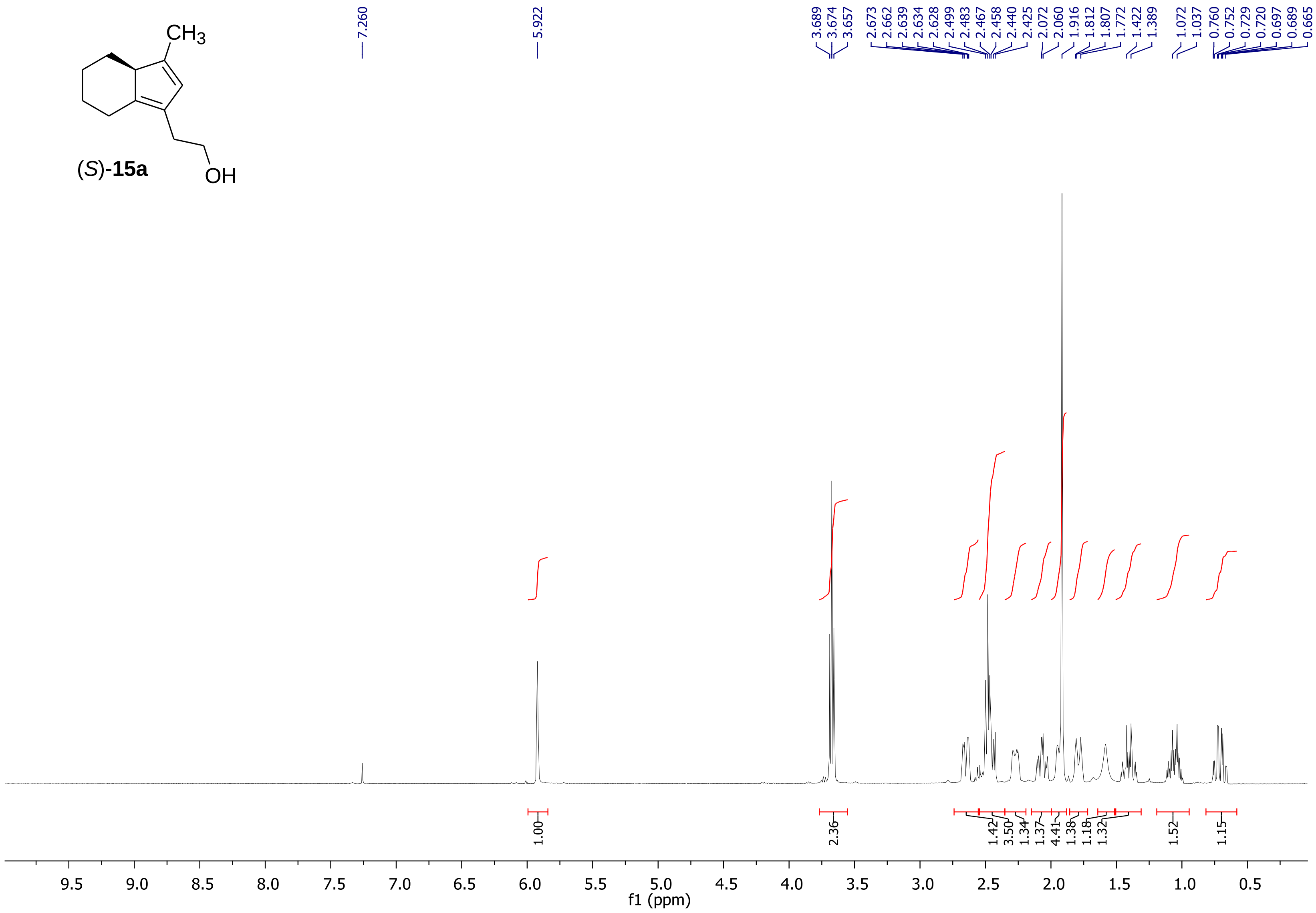

^{1}H NMR ($CDCl_3$, 400 MHz) of compound (*S*)-**15a**

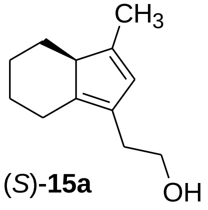

(*S*)-**15a**

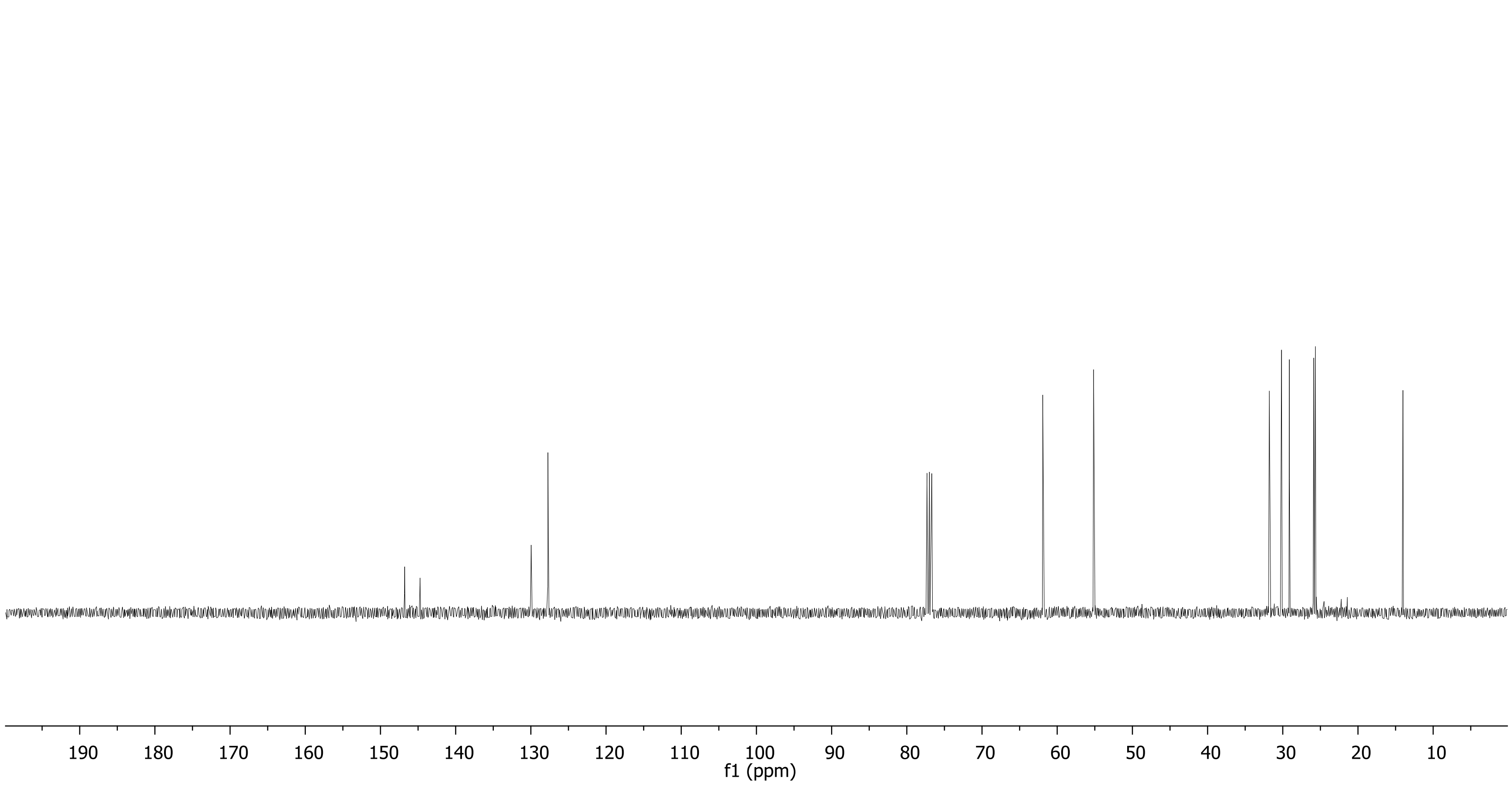

$^{13}C\{^{1}H\}$ NMR ($CDCl_3$, 100.4 MHz) of compound (*S*)-**15a**

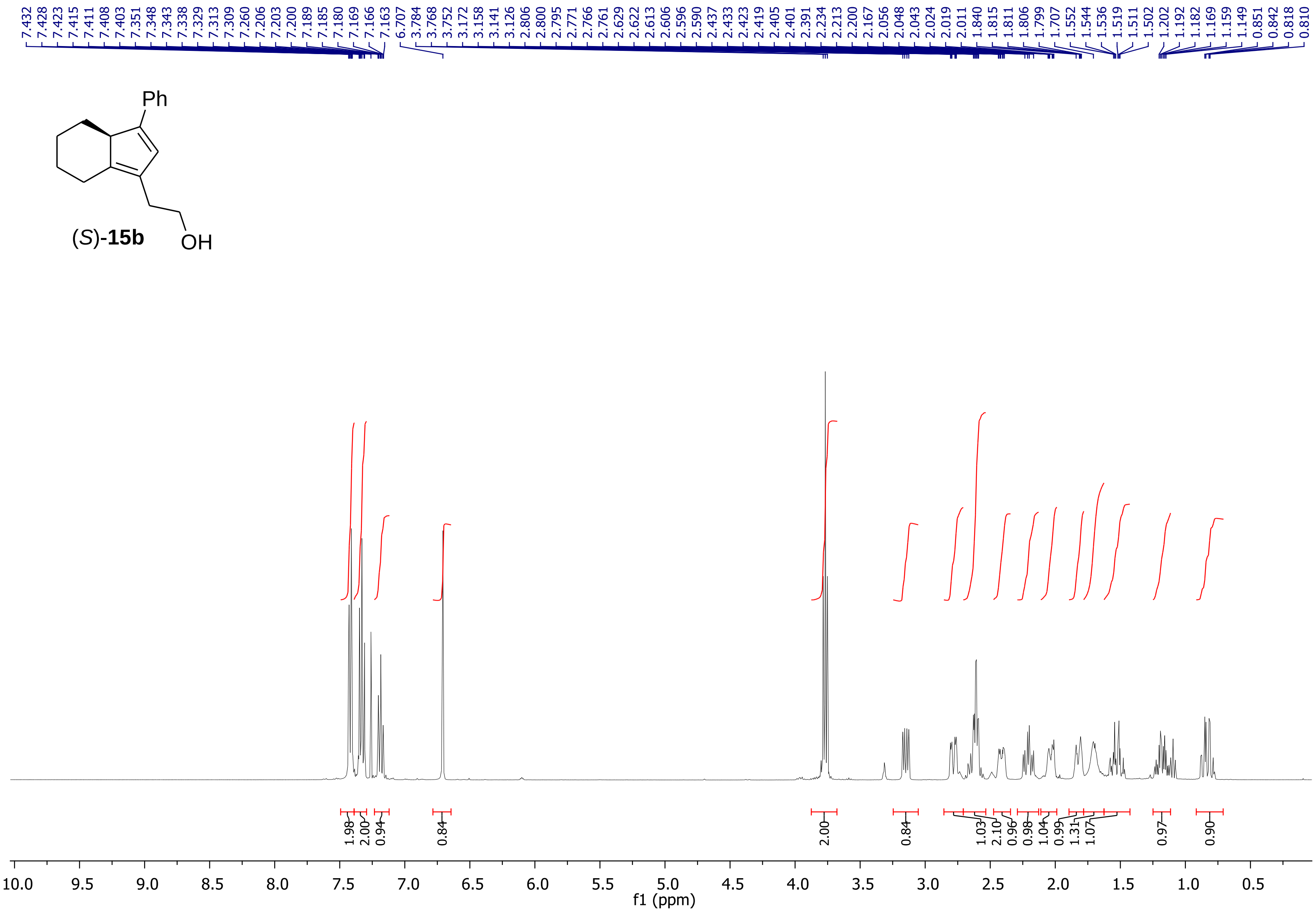

^{1}H NMR ($CDCl_3$, 400 MHz) of compound (*S*)-**15b**

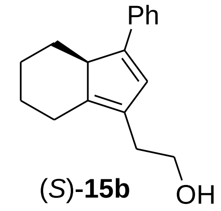

(*S*)-**15b**

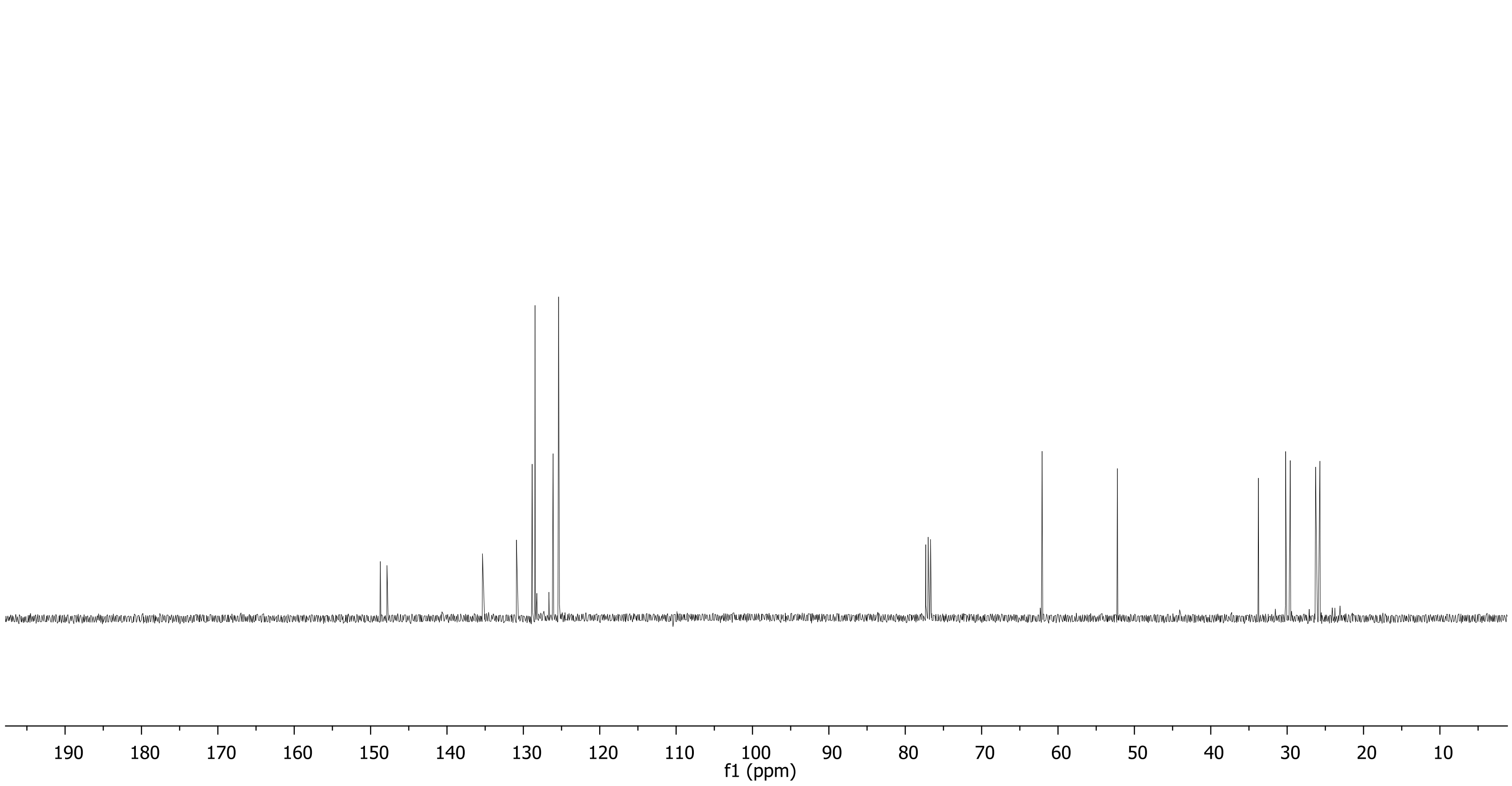

$^{13}C\{^{1}H\}$ NMR ($CDCl_3$, 100.4 MHz) of compound (*S*)-**15b**

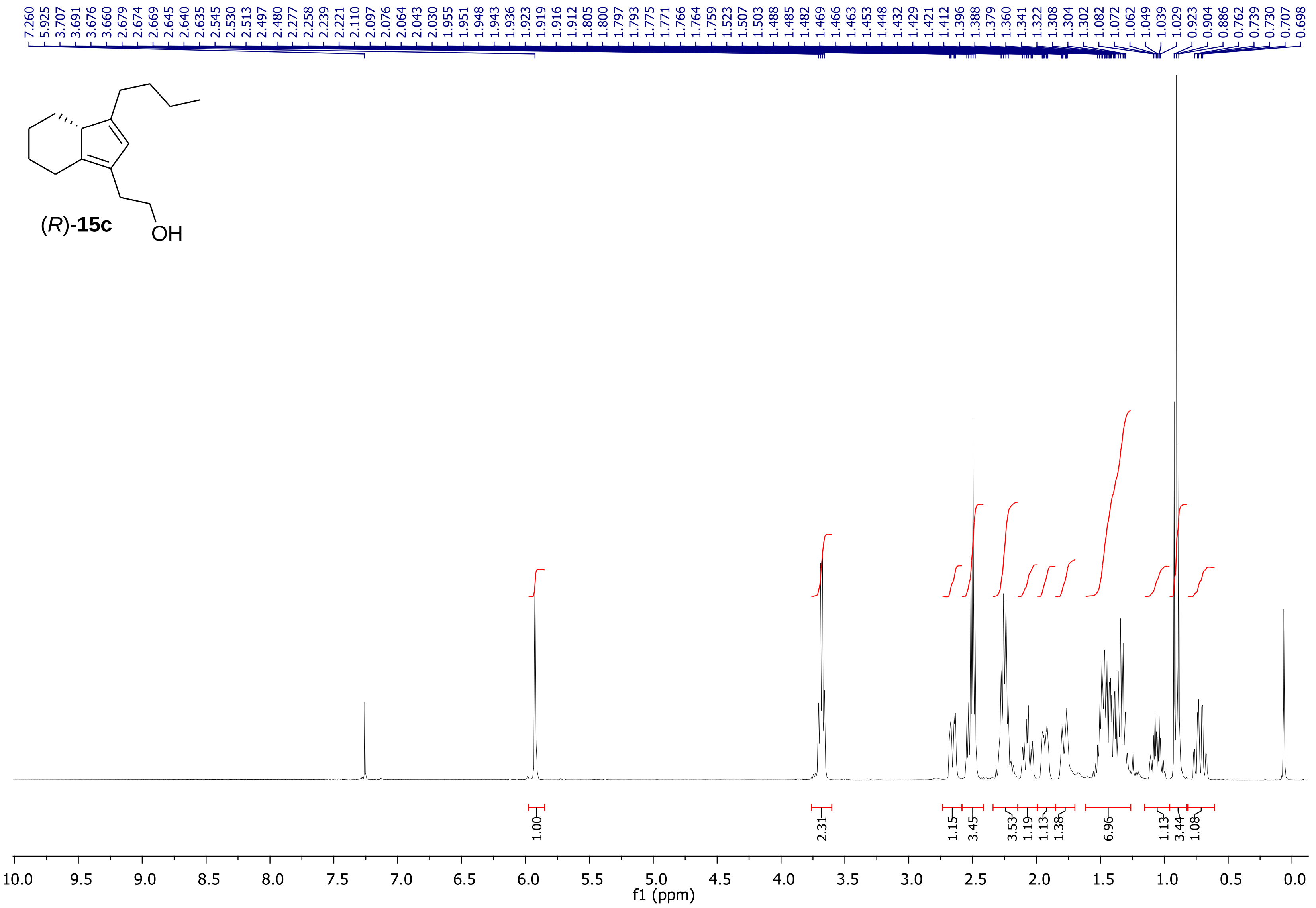

^{1}H NMR ($CDCl_3$, 400 MHz) of compound (*R*)-**15c**

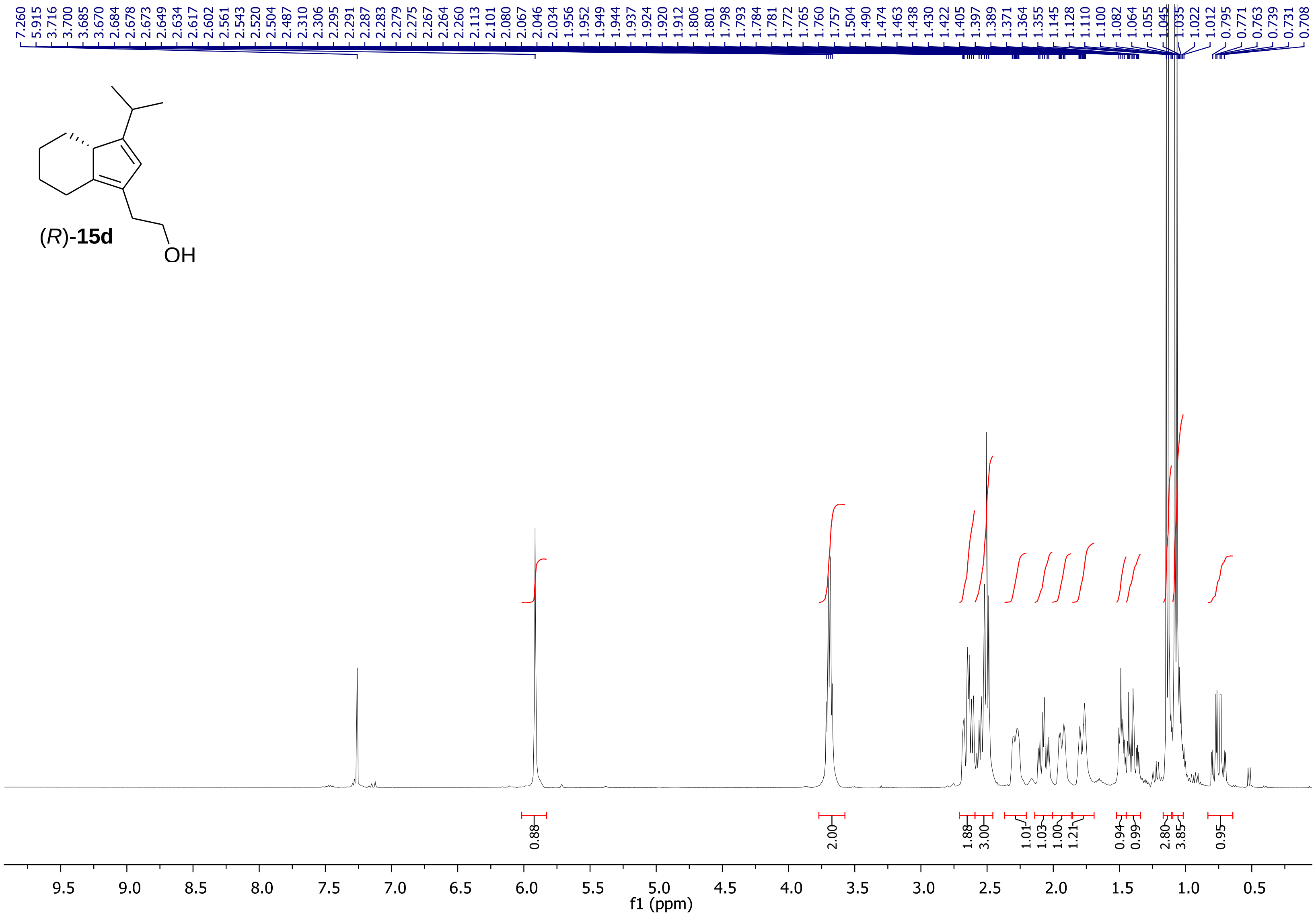

^{1}H NMR ($CDCl_3$, 200 MHz) of compound (*R*)-**15d**

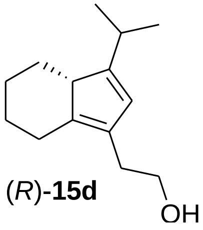

(*R*)-**15d**

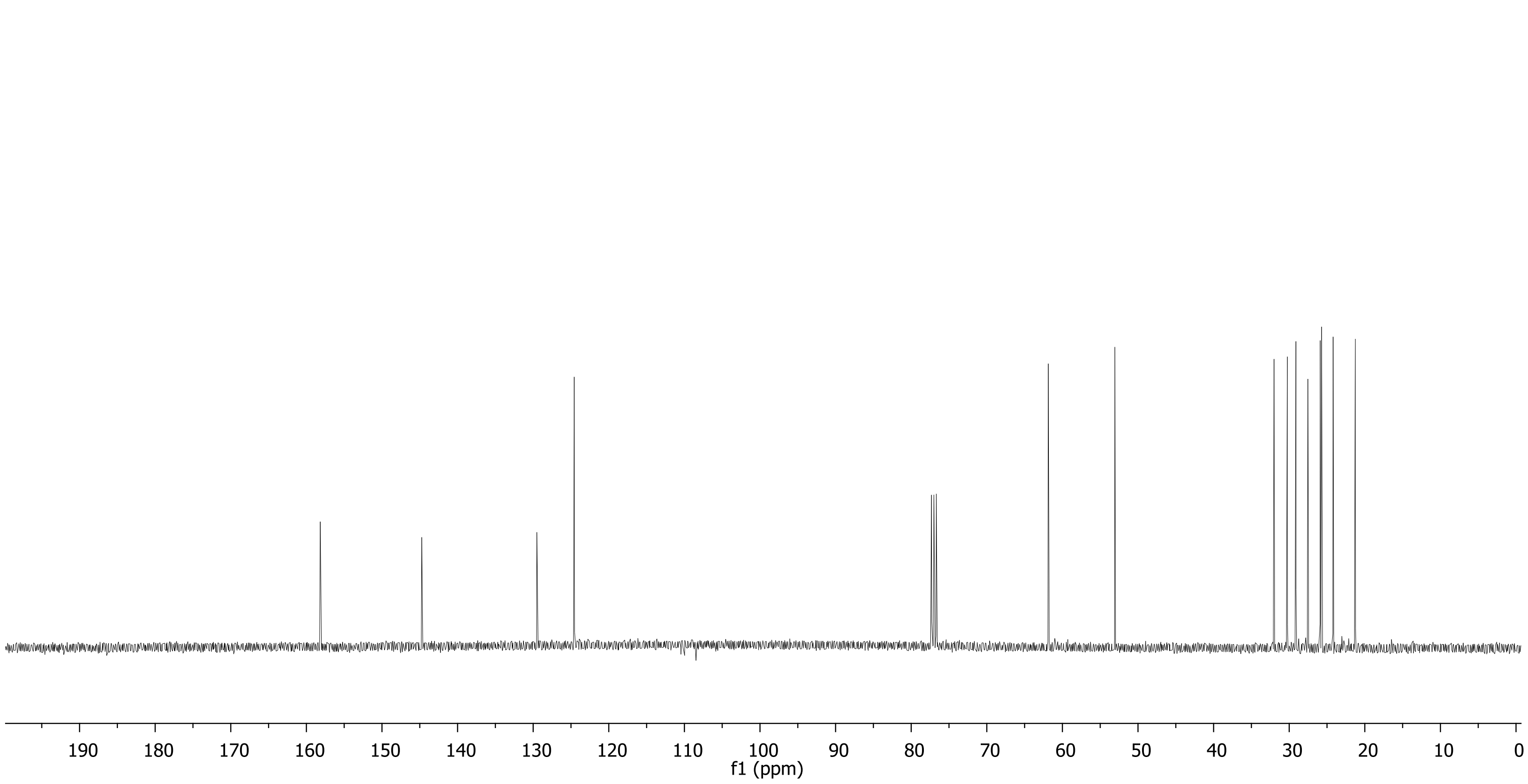

$^{13}C\{^{1}H\}$ NMR ($CDCl_3$, 100.4 MHz) of compound (*R*)-**15d**

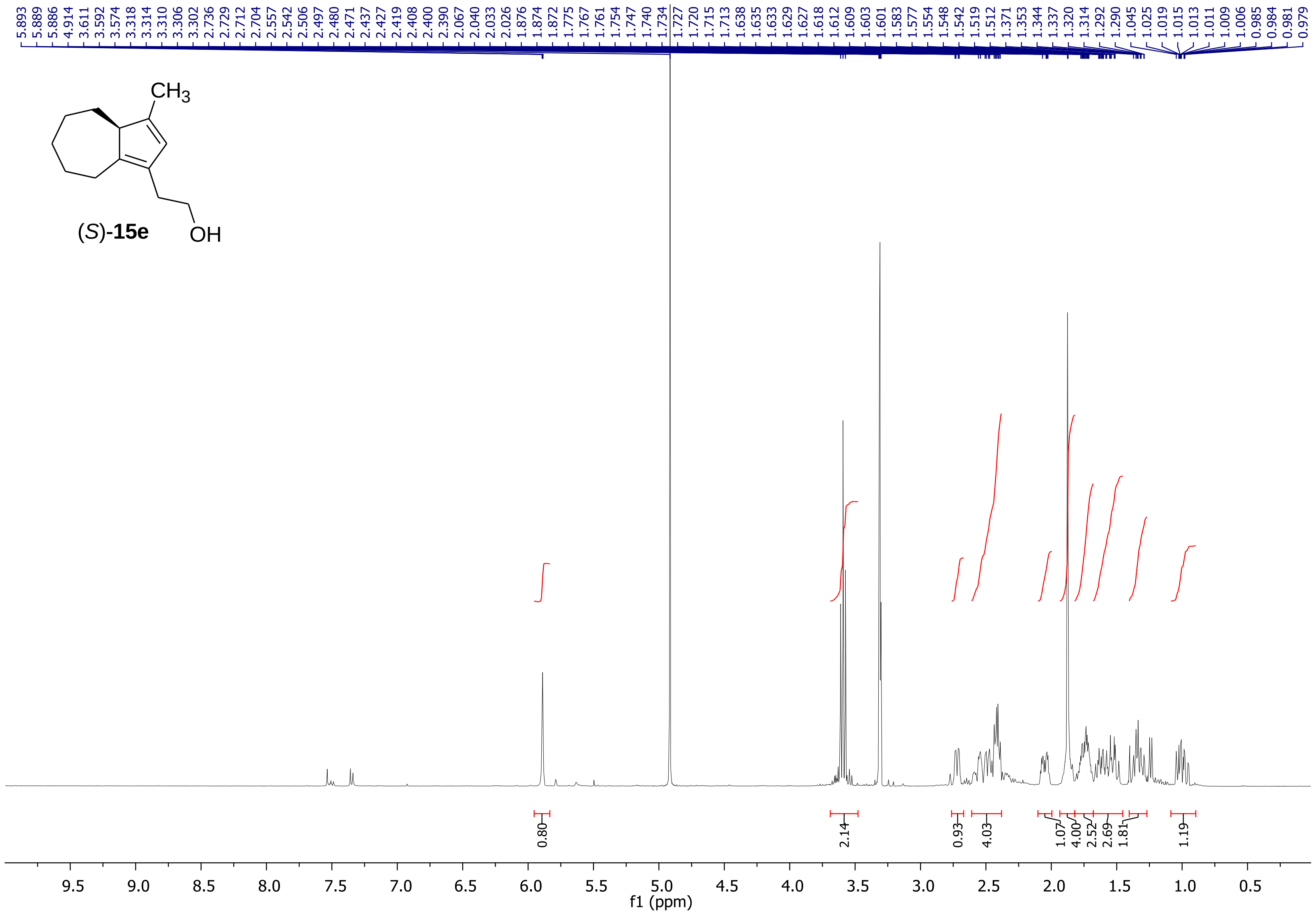

^{1}H NMR (CD_3OD, 400 MHz) of crude reaction mixture containing compound (*S*)-**15e**

^{1}H NMR (CD_3OD, 400 MHz) of crude reaction mixture containing compound (*S*)-**15f**

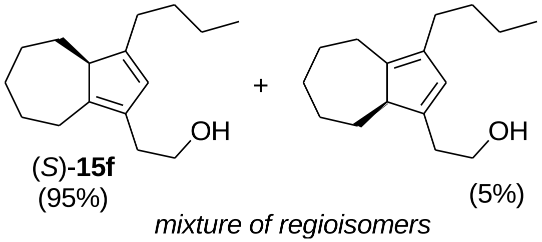

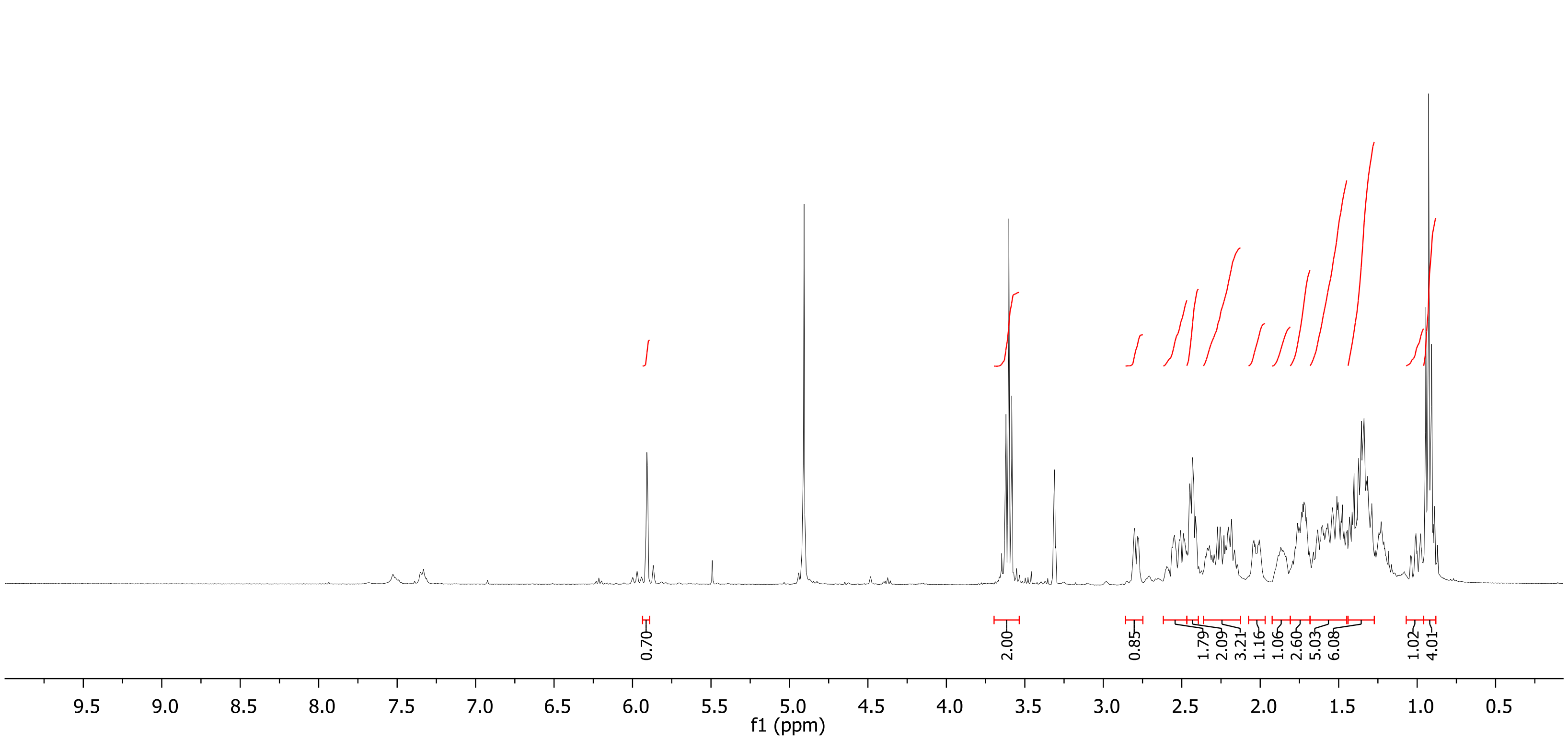

^{1}H NMR (CD_3OD, 400 MHz) of crude reaction mixture containing compound (*S*)-**15f**

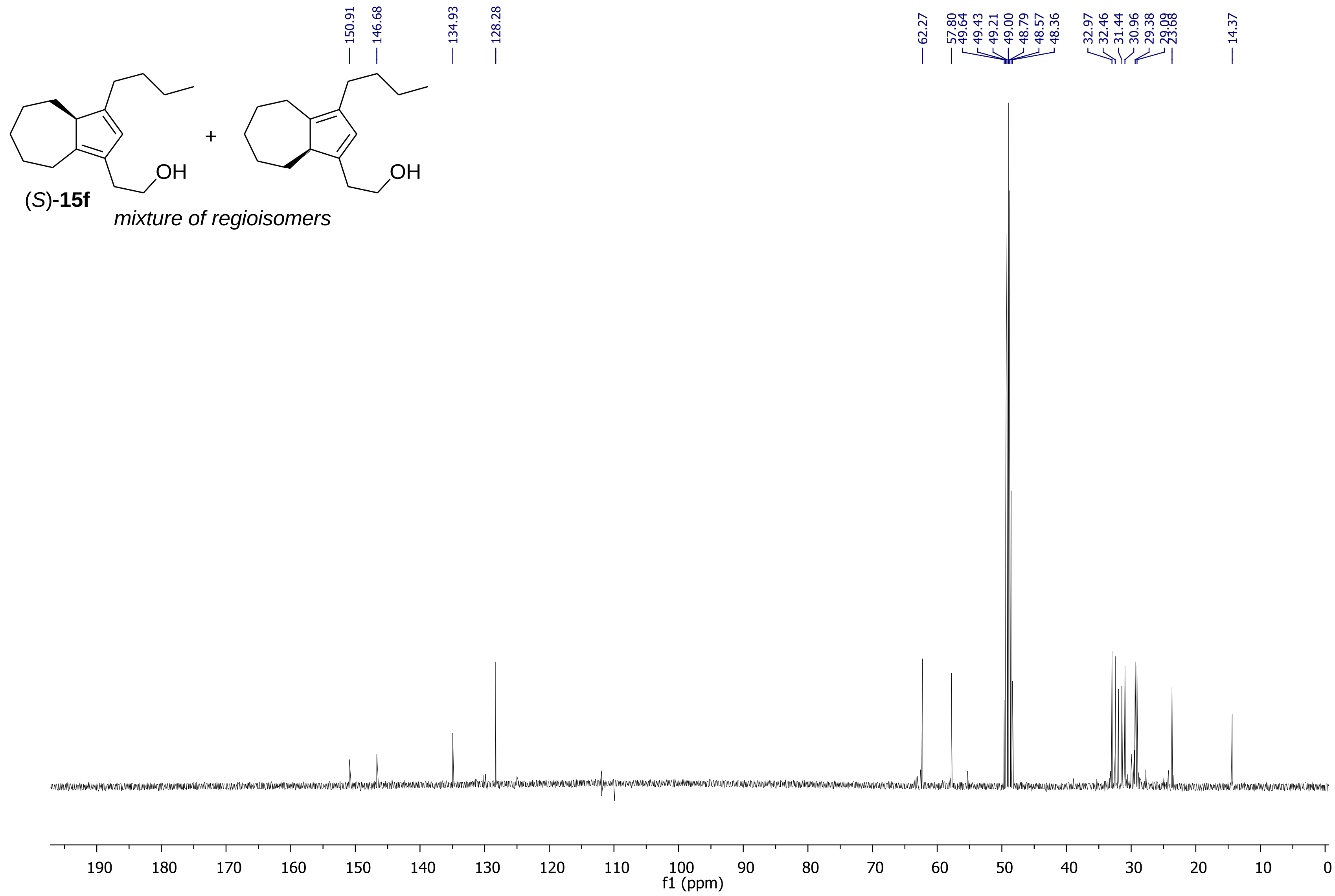

$^{13}C\{^{1}H\}$ NMR (CD_3OD, 100.4 MHz) of crude reaction mixture containing compound (*S*)-**15f**

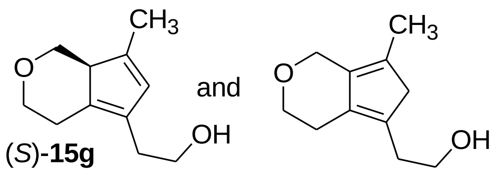

10 : 1 mixture of regioisomers

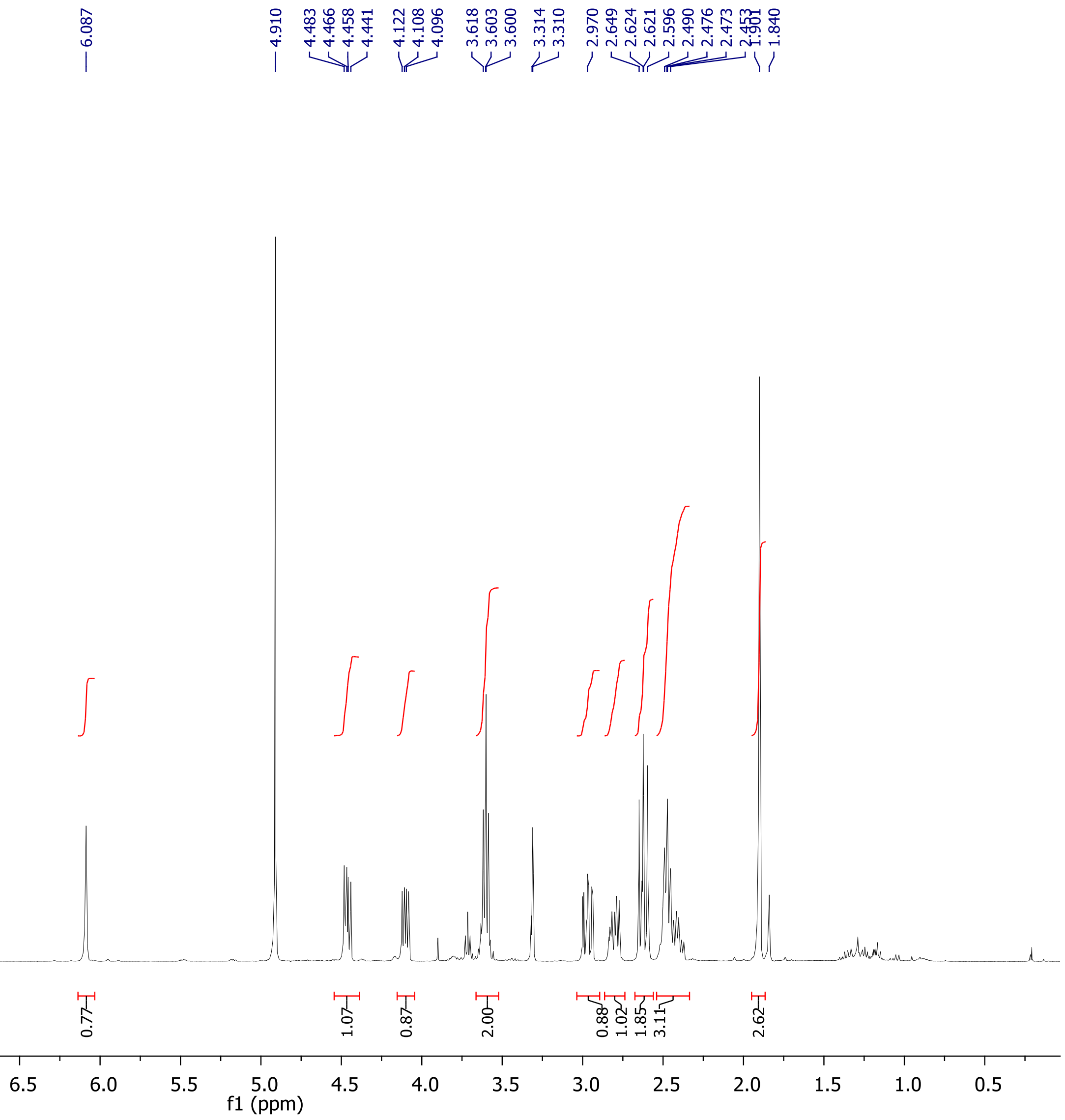

^{1}H NMR (CD_3OD, 400 MHz) of compound (*S*)-**15g**

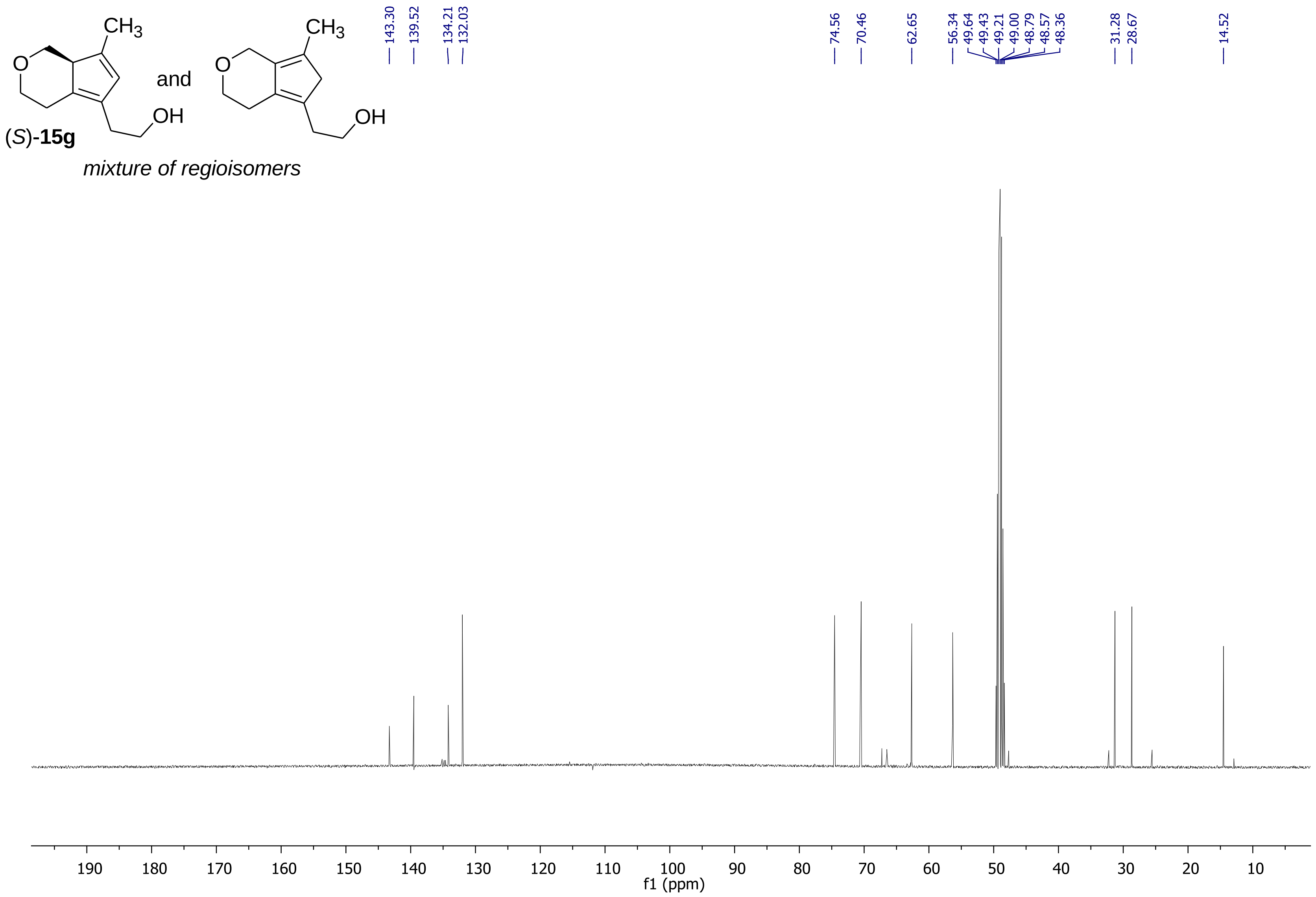

$^{13}C\{^{1}H\}$ NMR (CD_3OD, 100.4 MHz) of compound (*S*)-**15g**

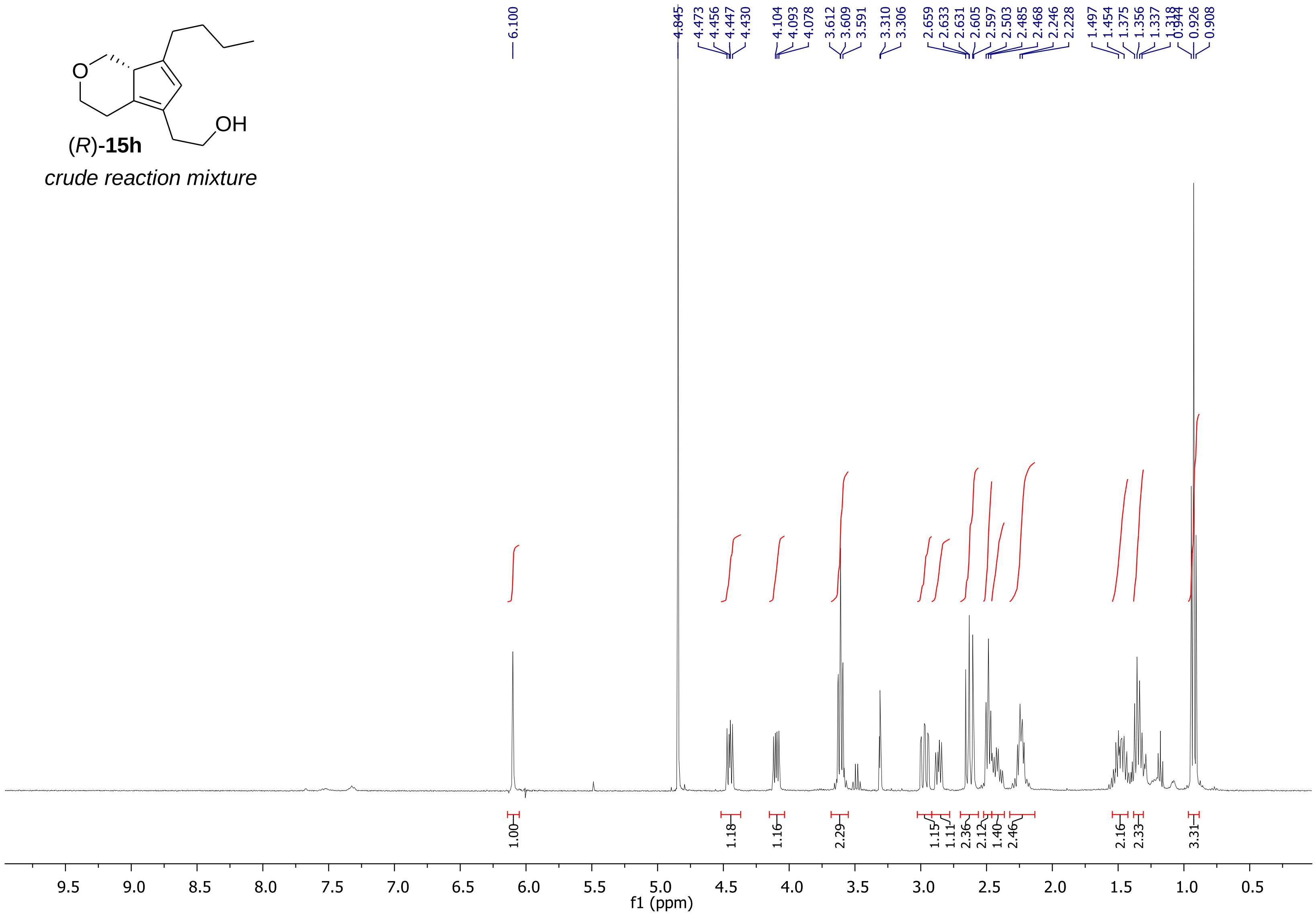

^{1}H NMR (CD_3OD, 400 MHz) of crude compound (*R*)-**15h**

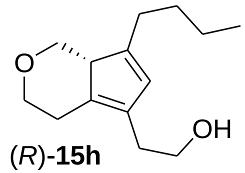

(R)-**15h**

crude reaction mixture

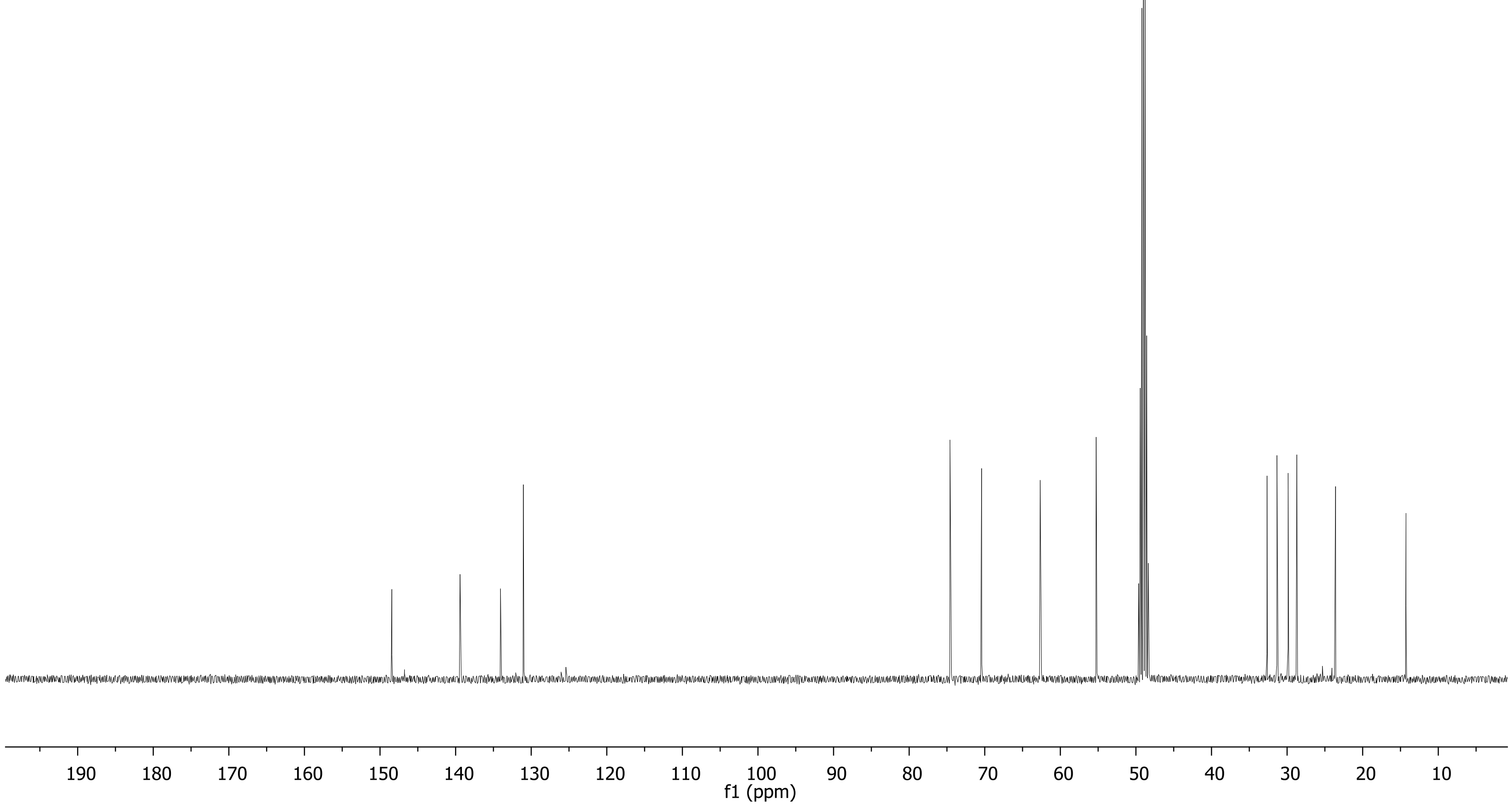

^{13}C{^{1}H} NMR (CD_3OD, 100.4 MHz) of crude compound (R)-**15h**

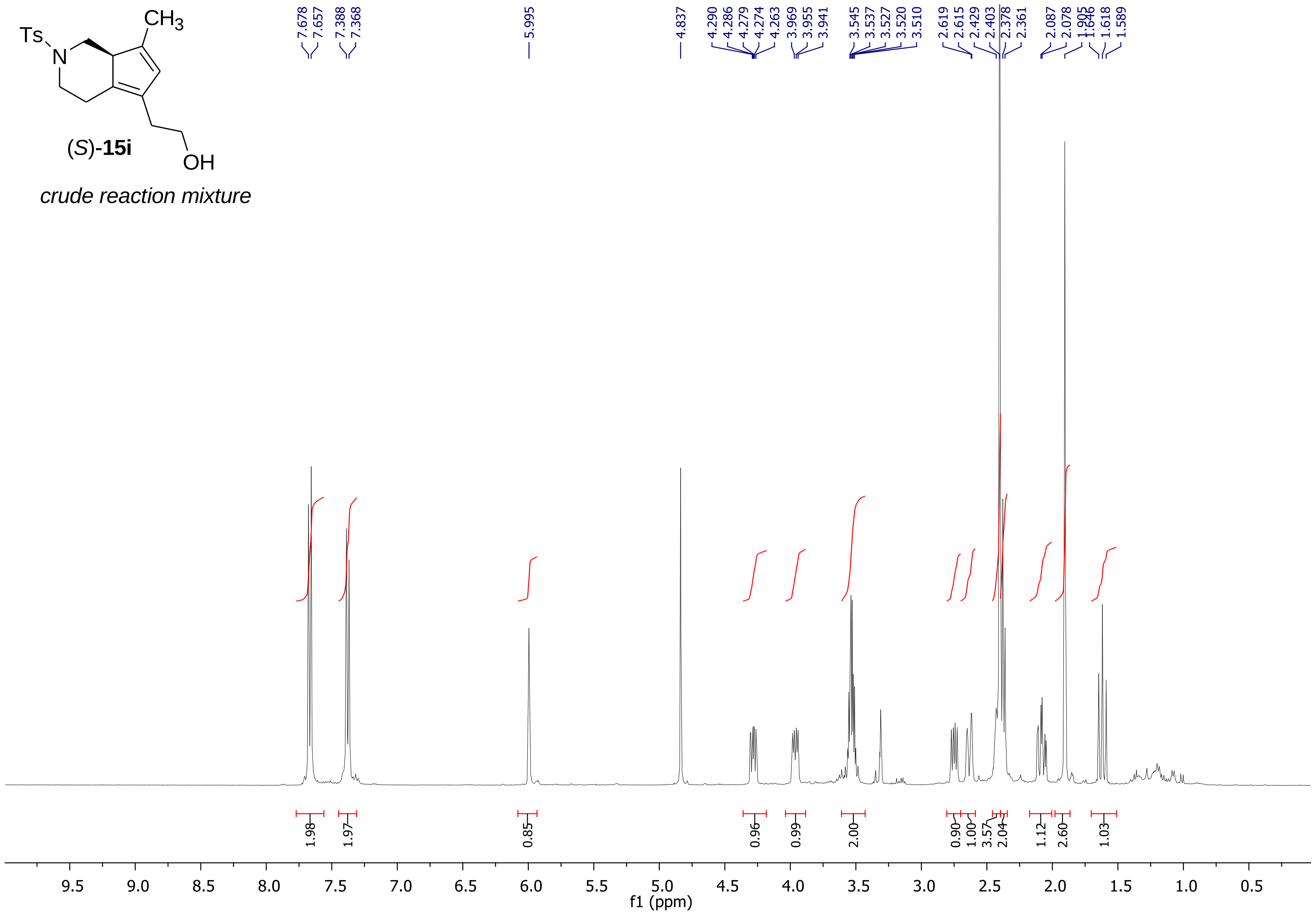

^{1}H NMR (CD_3OD, 400 MHz) of crude compound (*S*)-**15i**

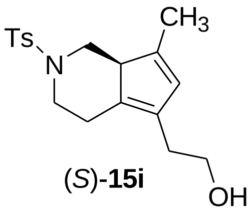

crude reaction mixture

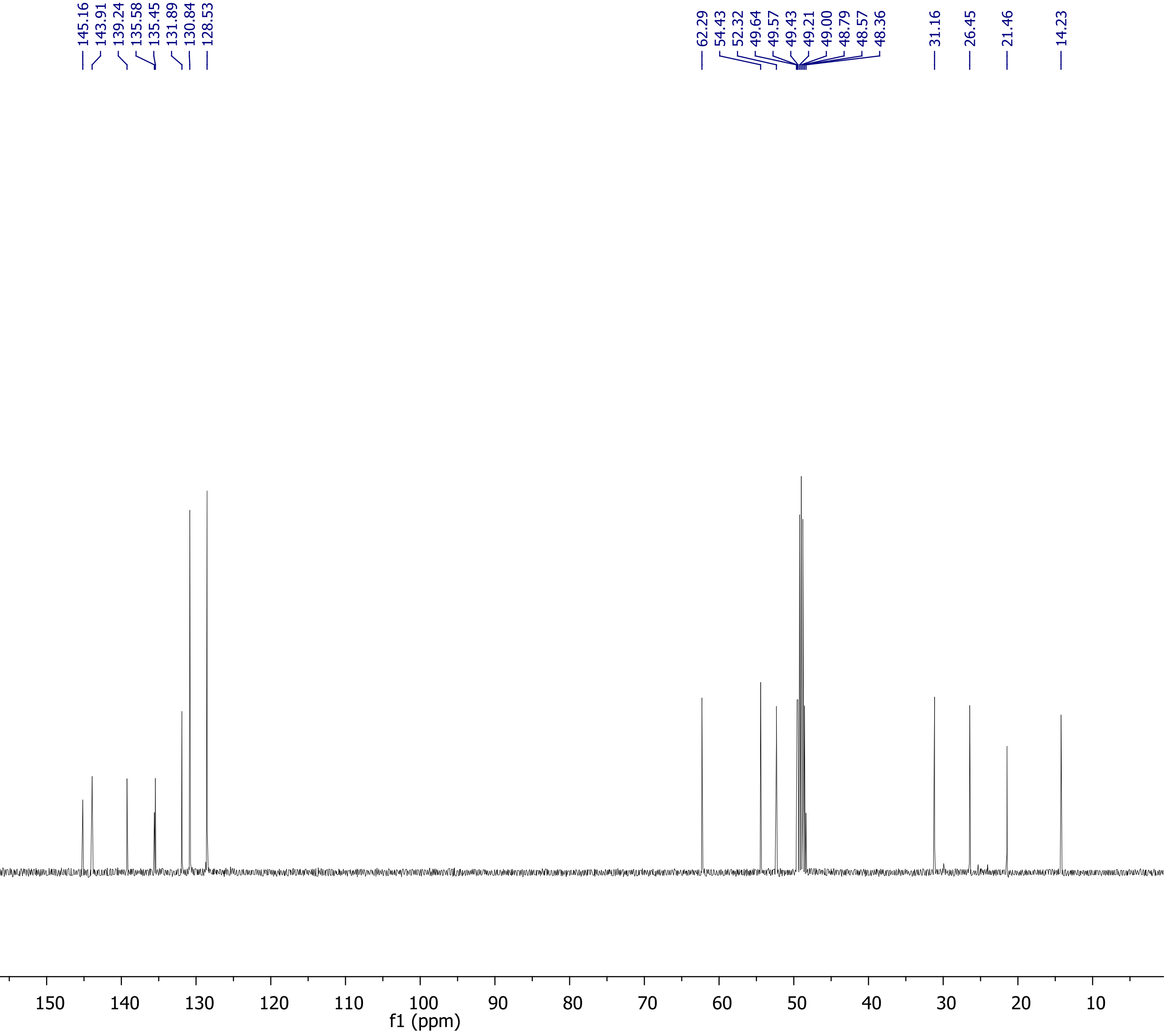

$^{13}C\{^{1}H\}$ NMR (CD_3OD, 100.4 MHz) of crude compound (*S*)-**15i**

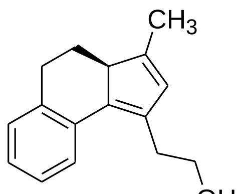

(*S*)-**15j**

crude reaction mixture

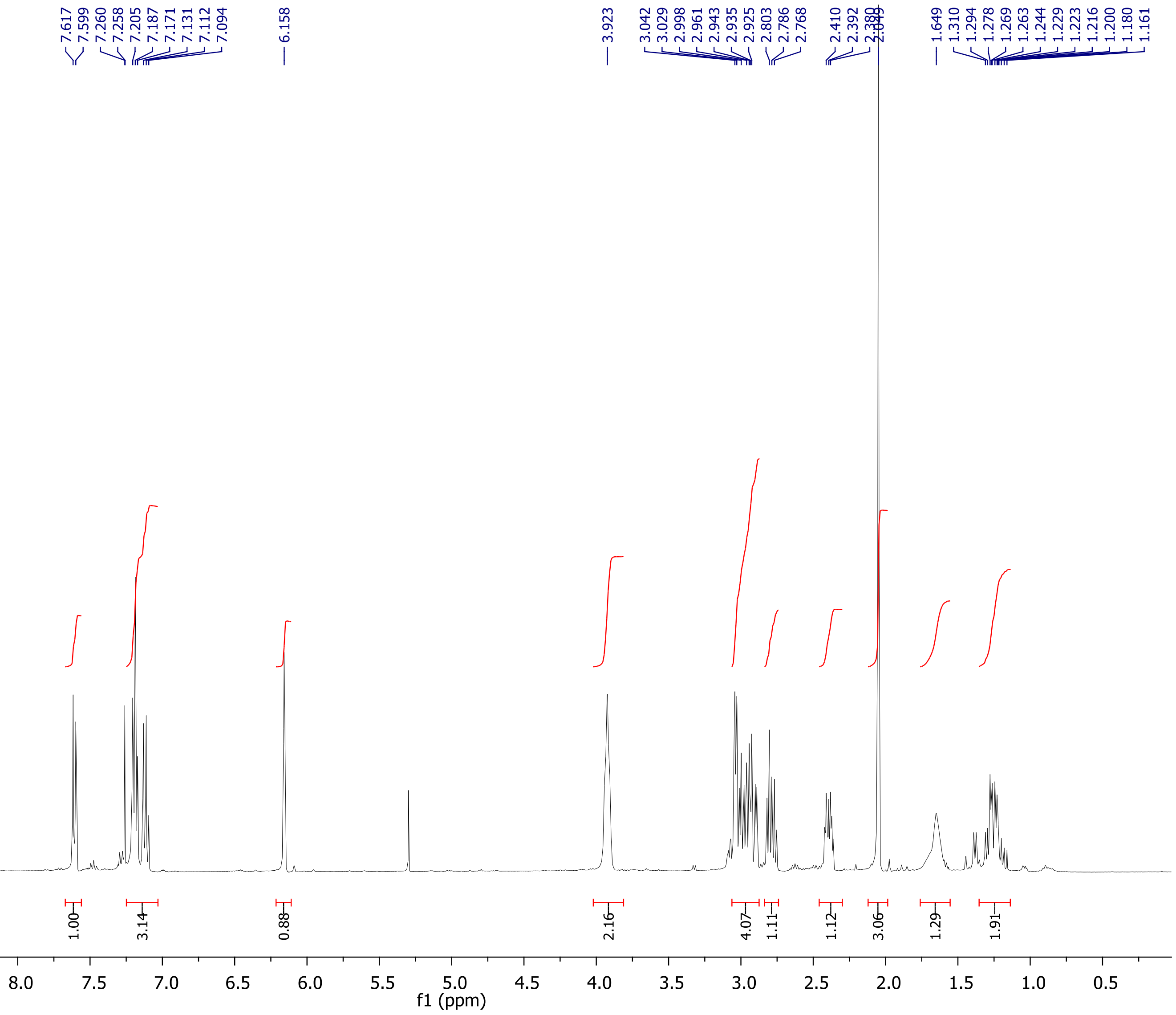

^{1}H NMR ($CDCl_3$, 400 MHz) of crude compound (*S*)-**15j**

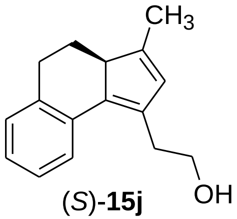

(*S*)-**15j**

crude reaction mixture

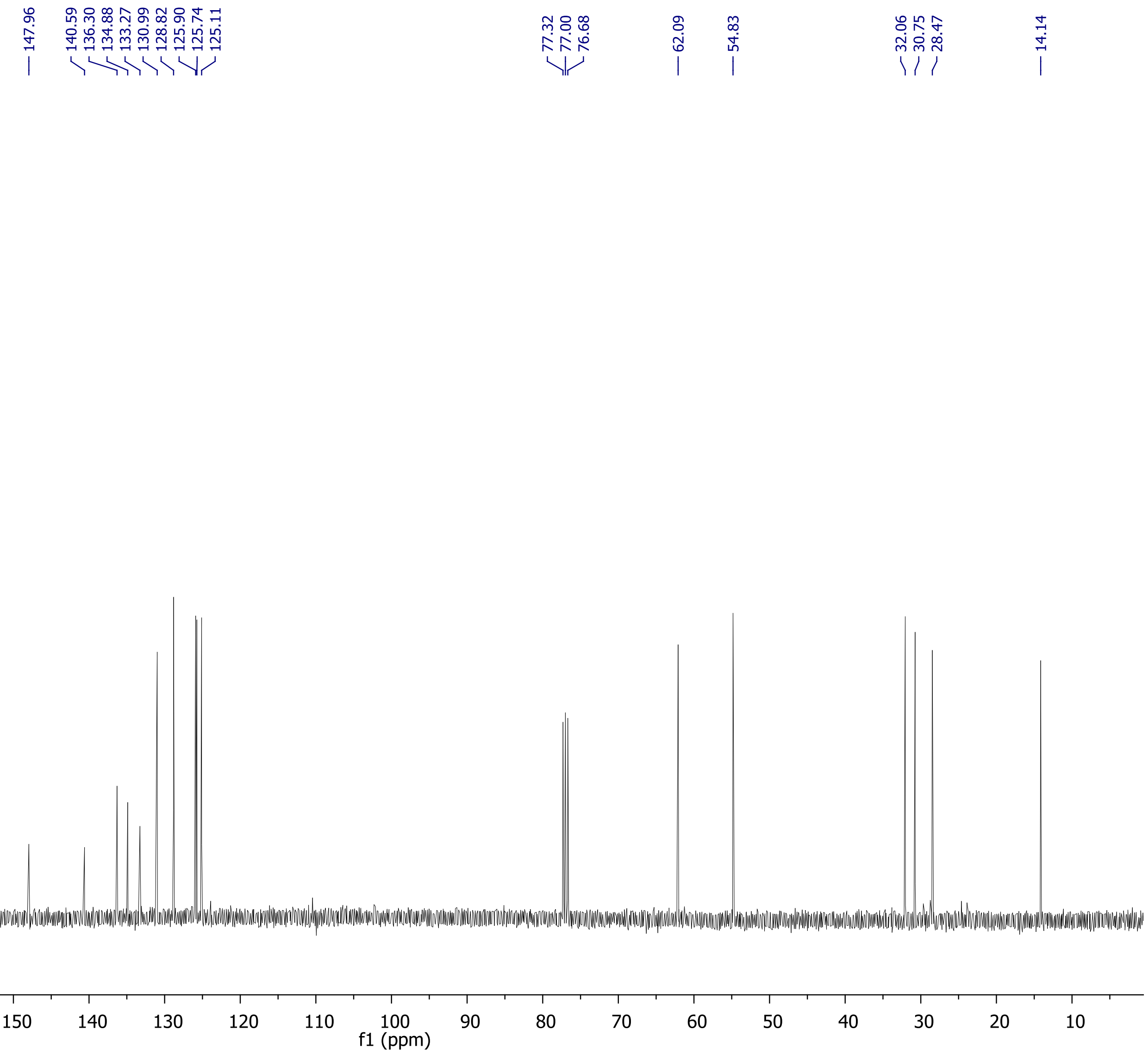

$^{13}C\{^{1}H\}$ NMR ($CDCl_3$, 100.4 MHz) of crude compound (*S*)-**15j**

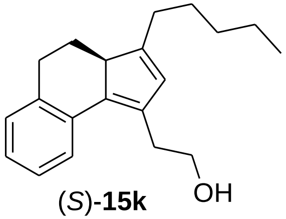

(*S*)-**15k**

crude reaction mixture

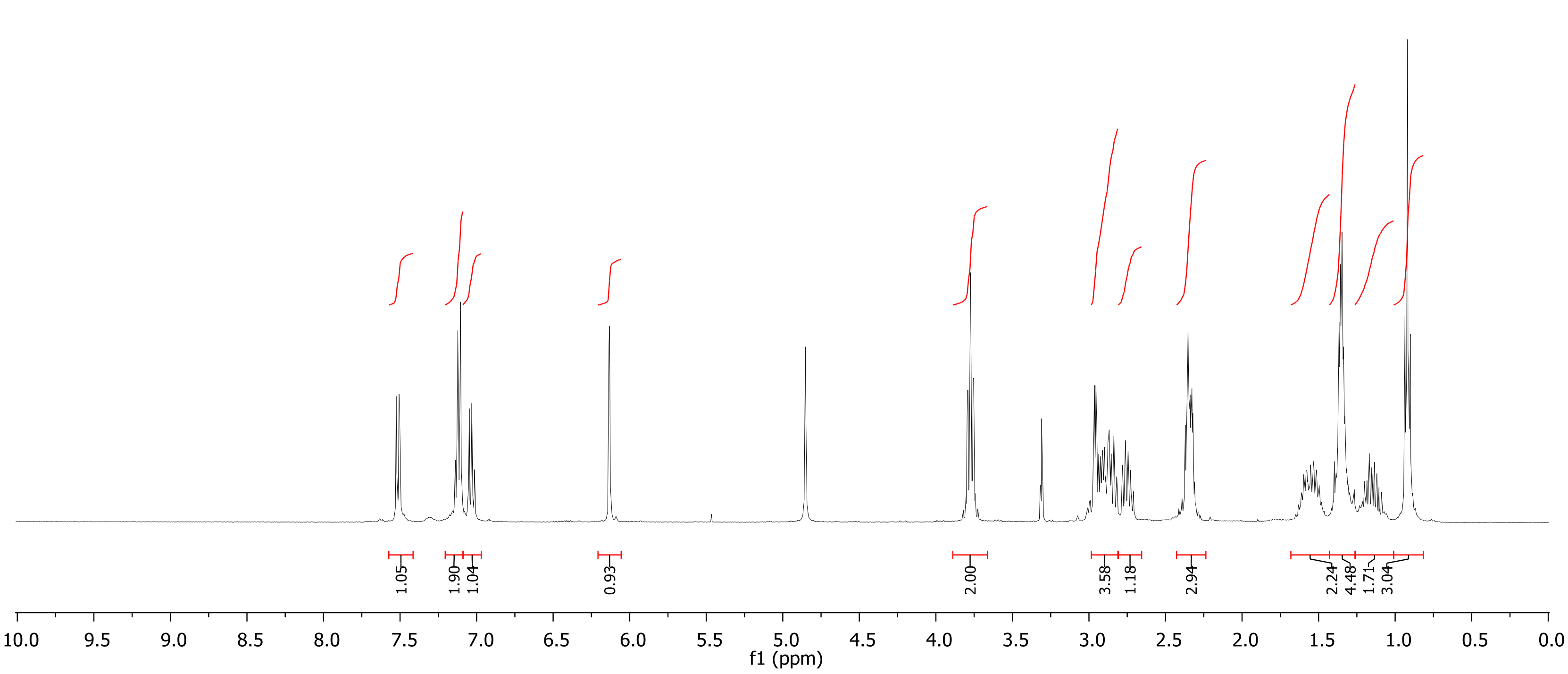

^{1}H NMR (CD_3OD, 400 MHz) of crude compound (*S*)-**15k**

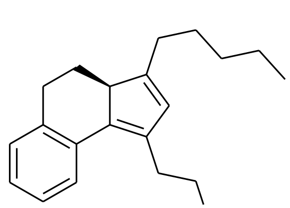

(*S*)-**15k**

crude reaction mixture

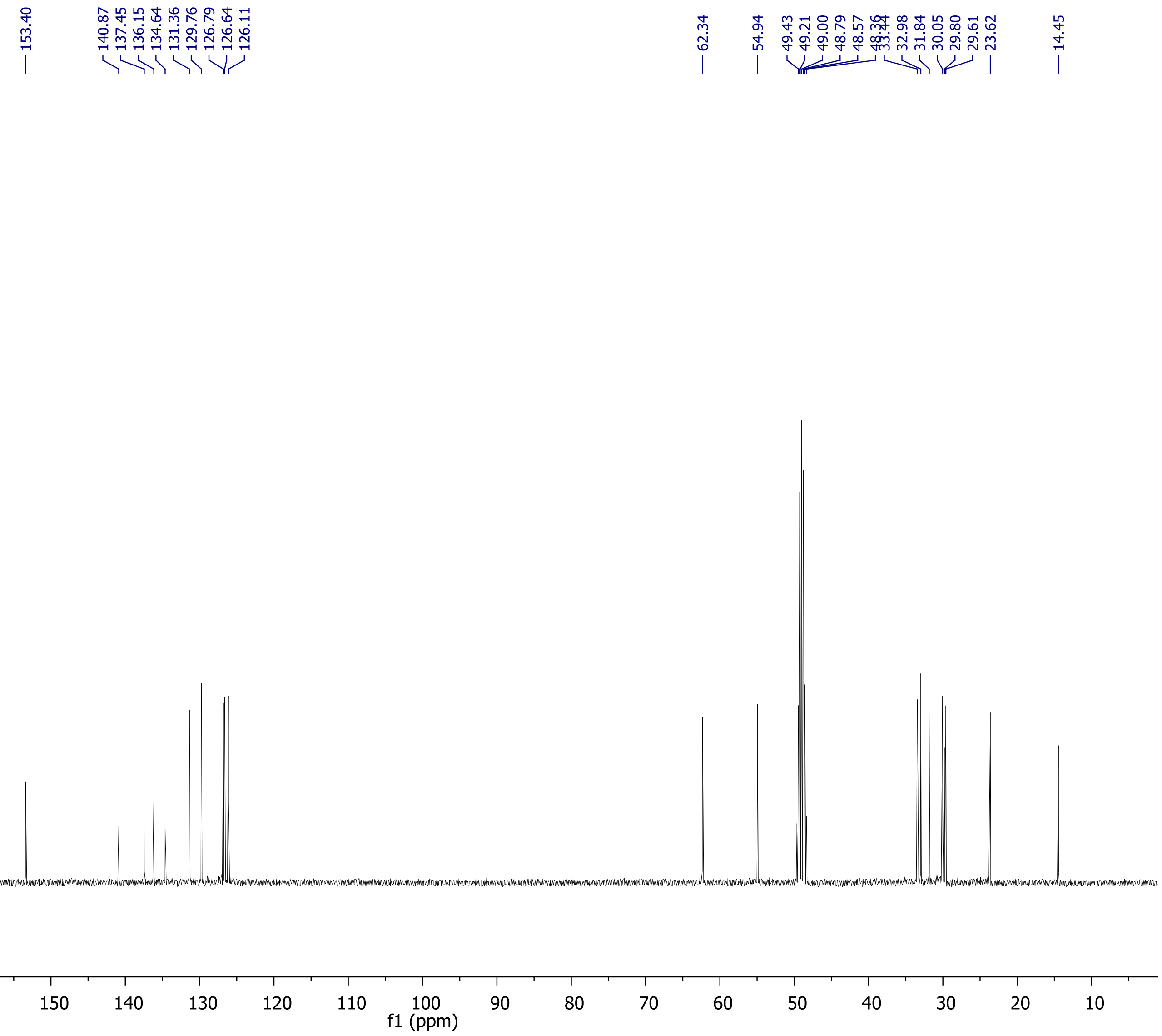

$^{13}C\{^{1}H\}$ NMR (CD_3OD, 100.4 MHz) of crude compound (*S*)-**15k**

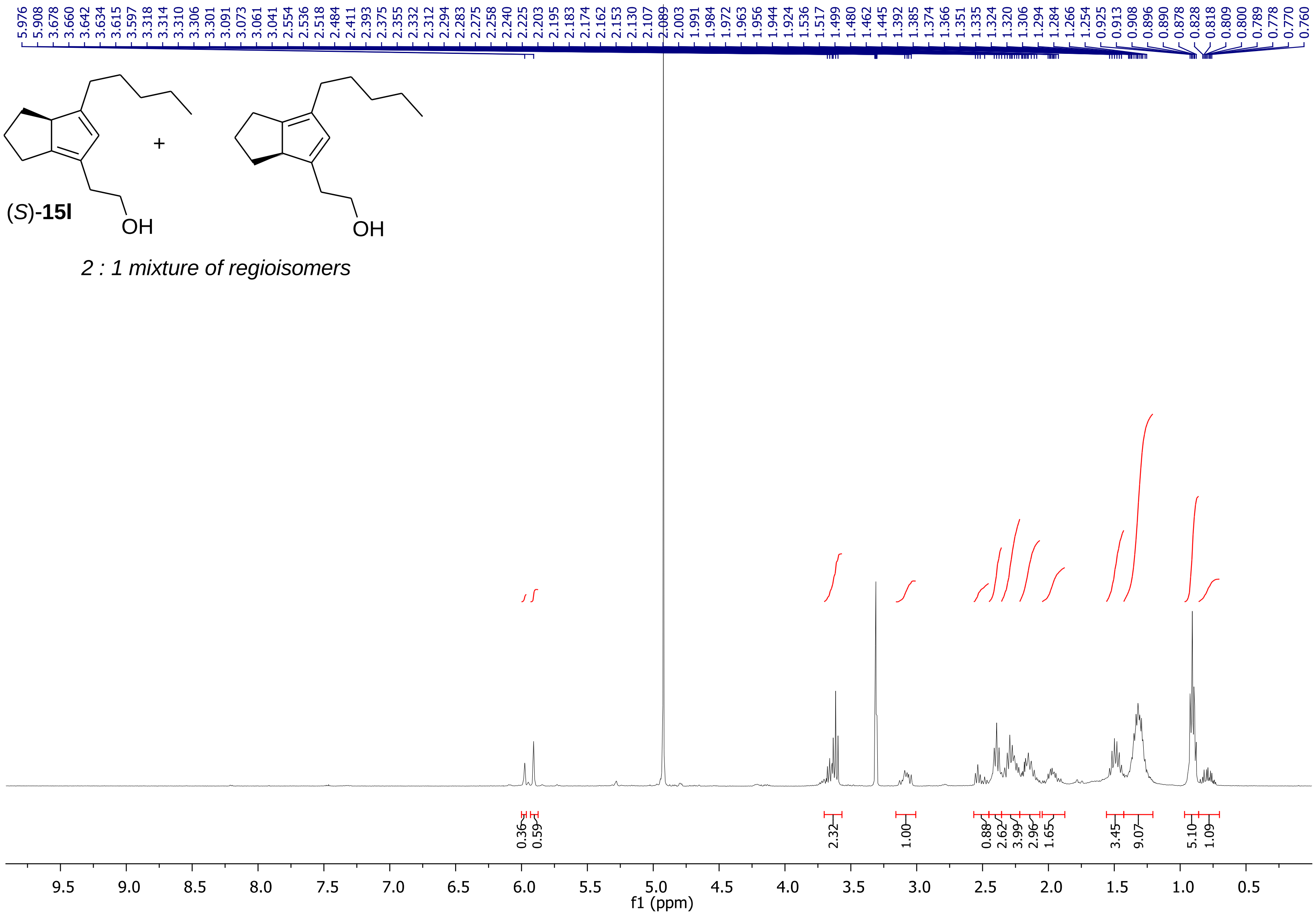

^{1}H NMR (CD_3OD, 400 MHz) of compound (*S*)-**15l**

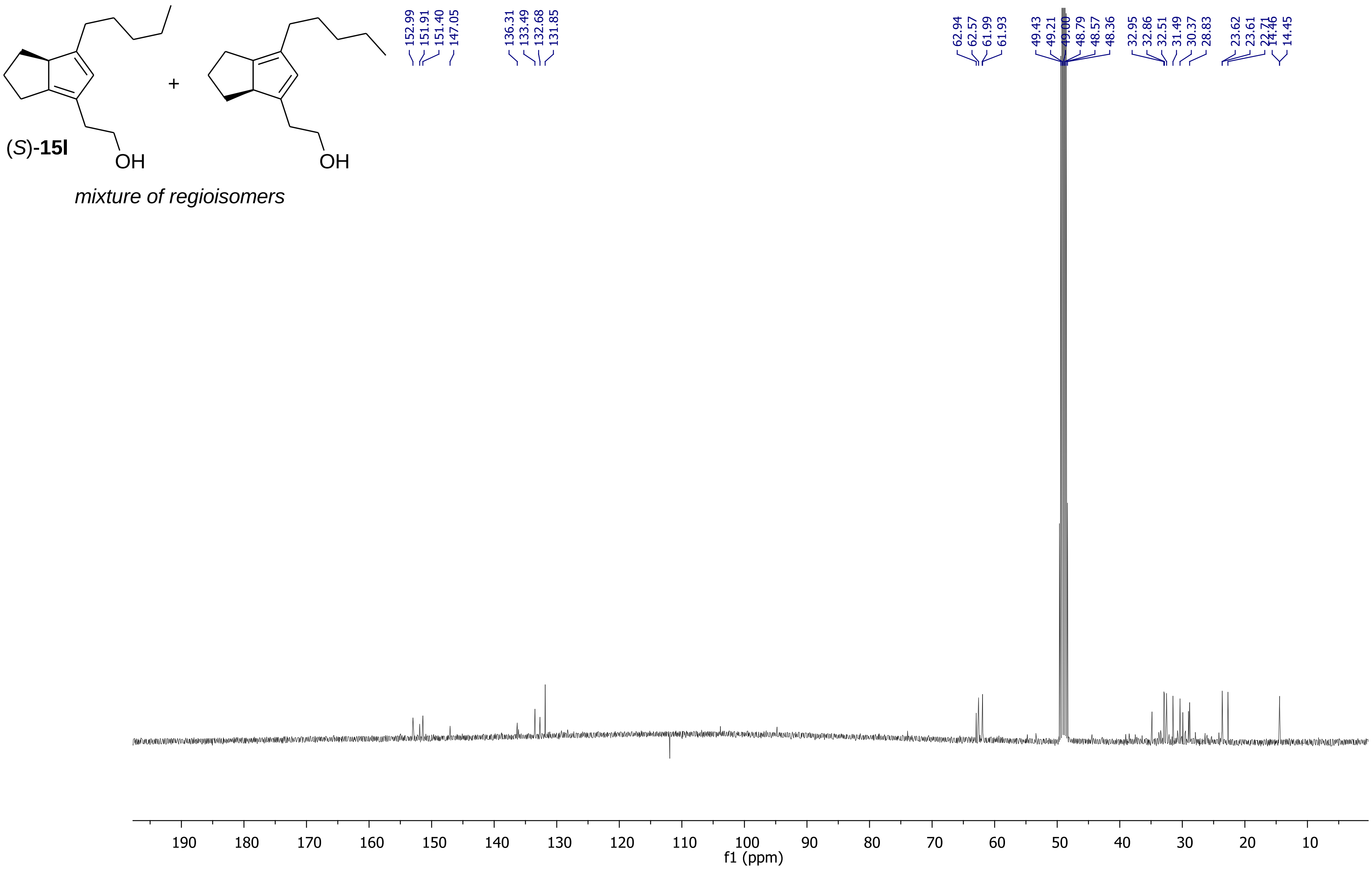

(*S*)-**15l**

mixture of regioisomers

$^{13}C\{^{1}H\}$ NMR (CD_3OD, 100.4 MHz) of compound (*S*)-**15l**

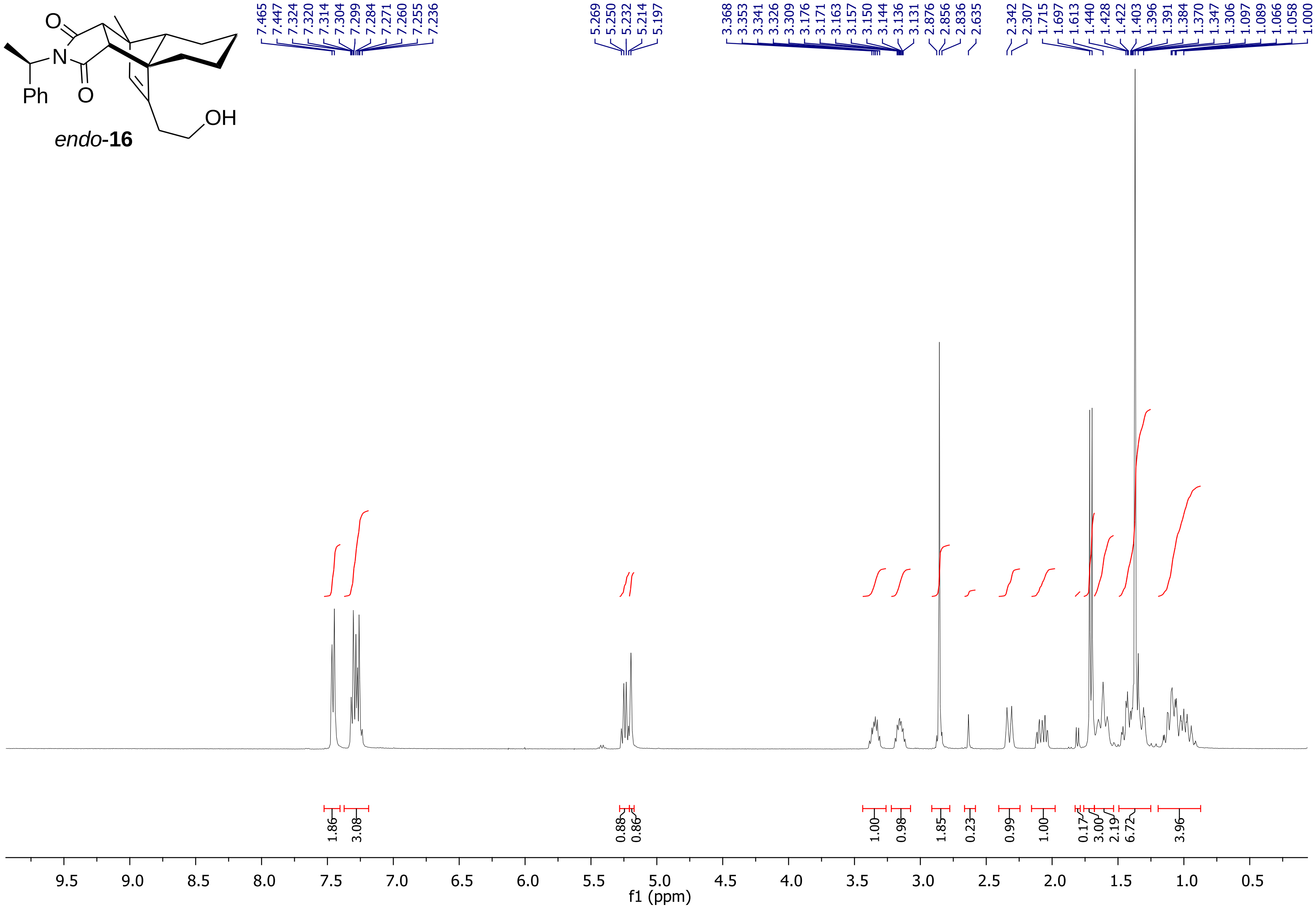

^{1}H NMR ($CDCl_3$, 400 MHz) of compound **16**

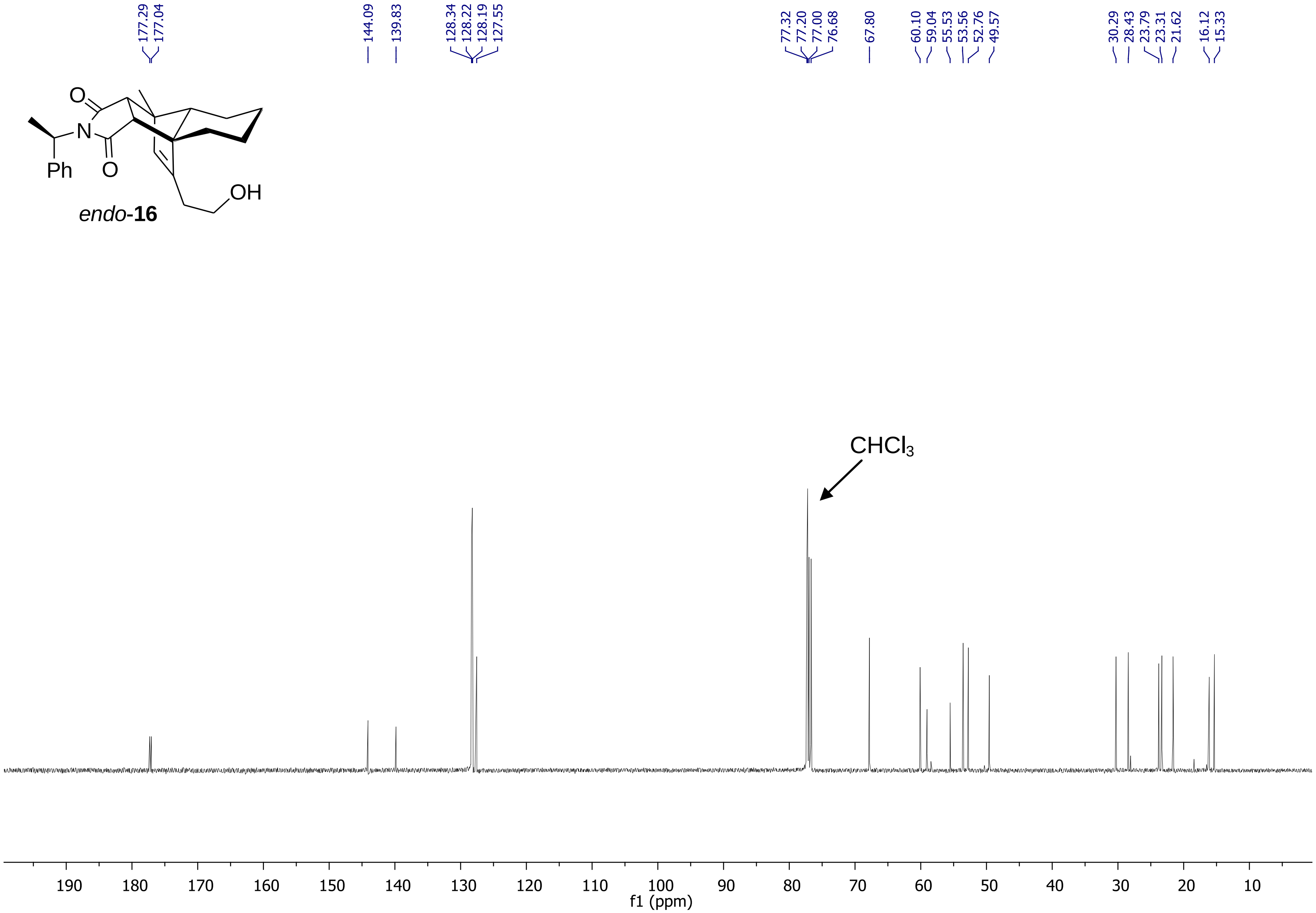

$^{13}C\{^{1}H\}$ NMR ($CDCl_3$, 100.4 MHz) of compound **16**

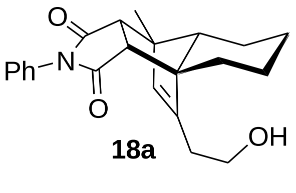

18a

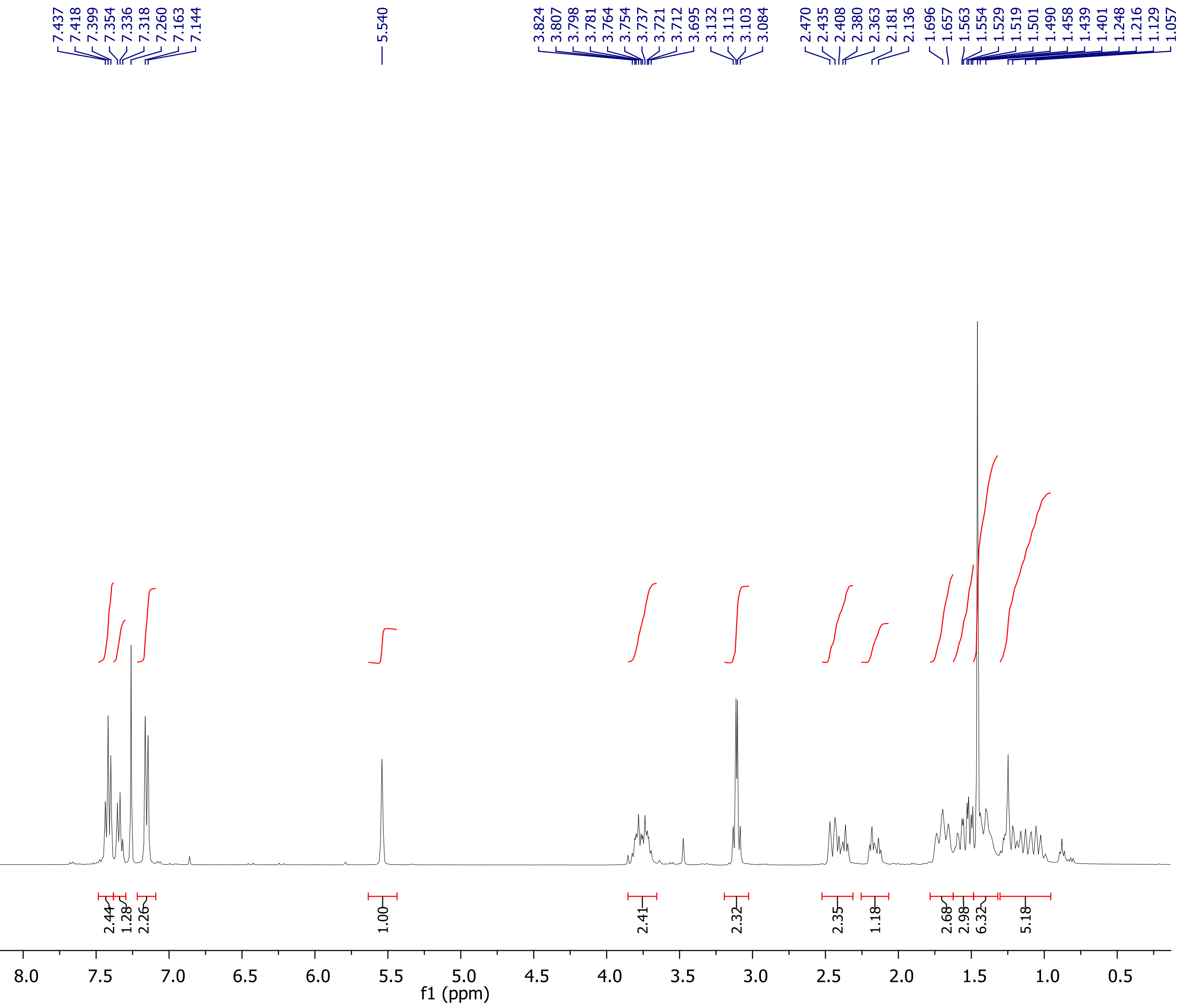

^{1}H NMR ($CDCl_3$, 400 MHz) of compound **18a**

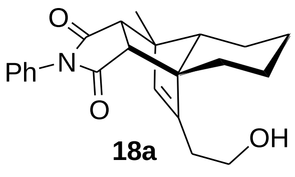

18a

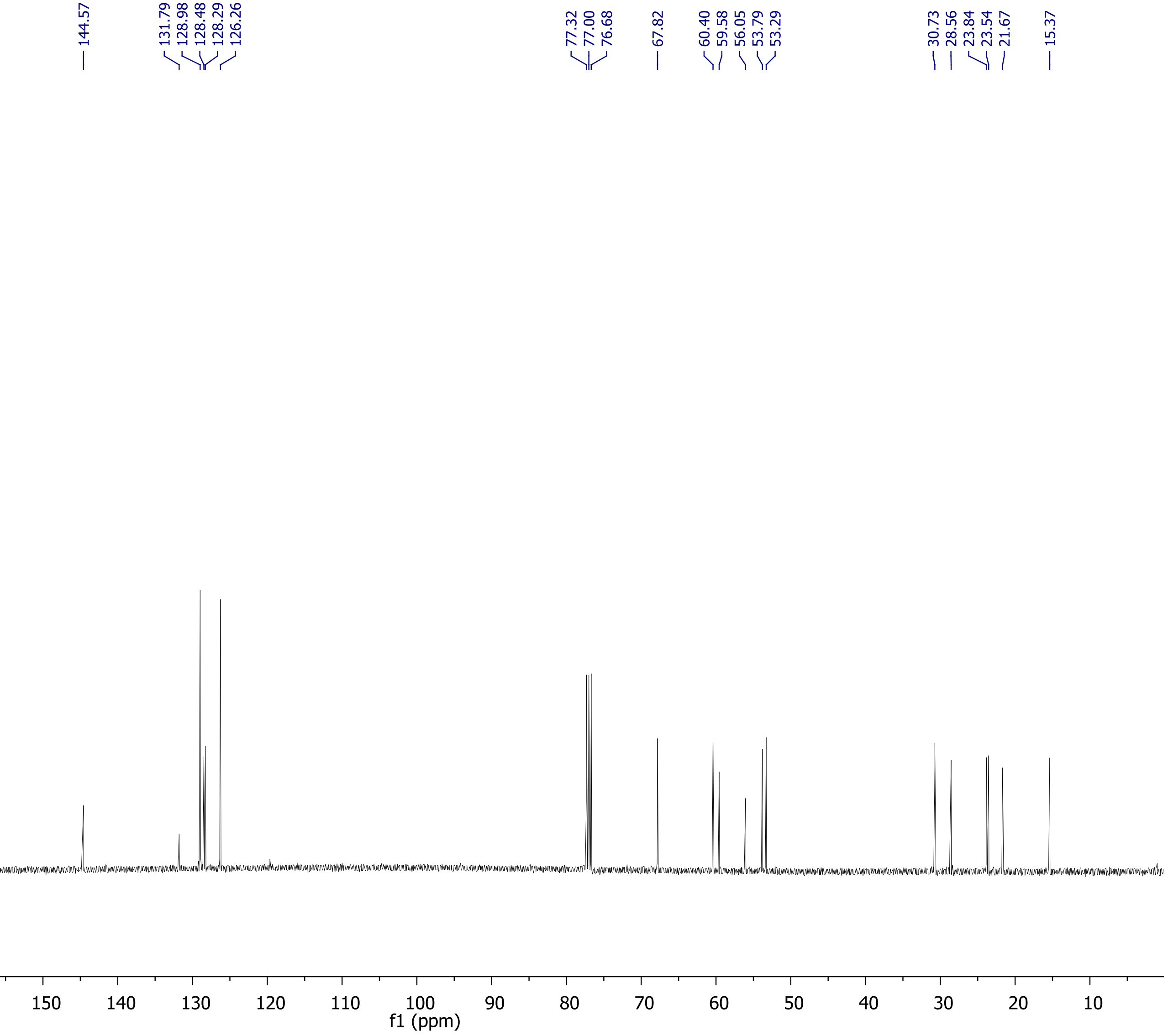

$^{13}C\{^{1}H\}$ NMR ($CDCl_3$, 100.4 MHz) of compound **18a**

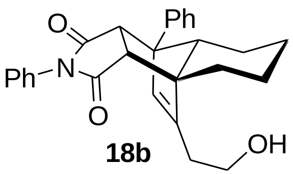

18b

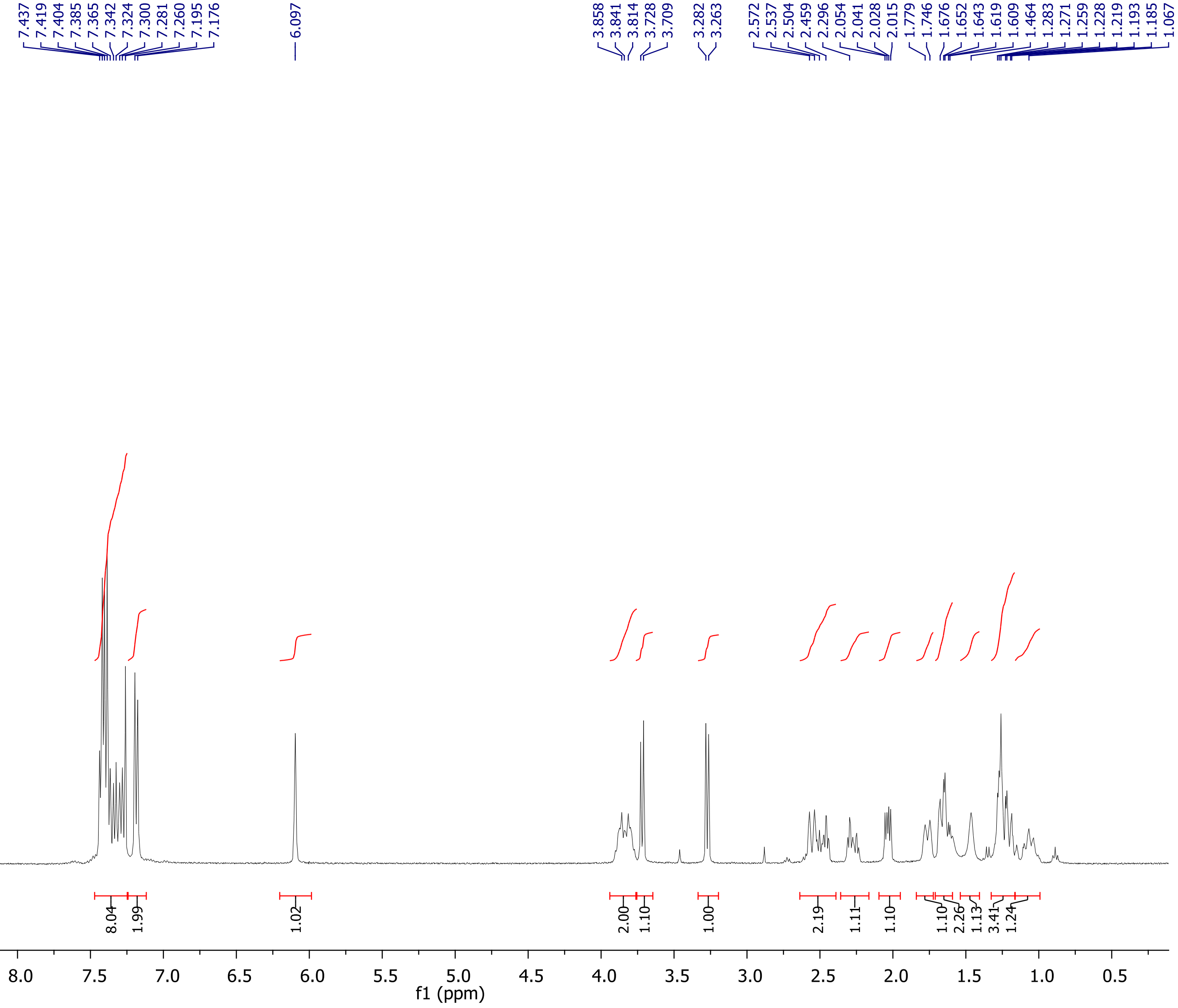

^{1}H NMR ($CDCl_3$, 400 MHz) of compound **18b**

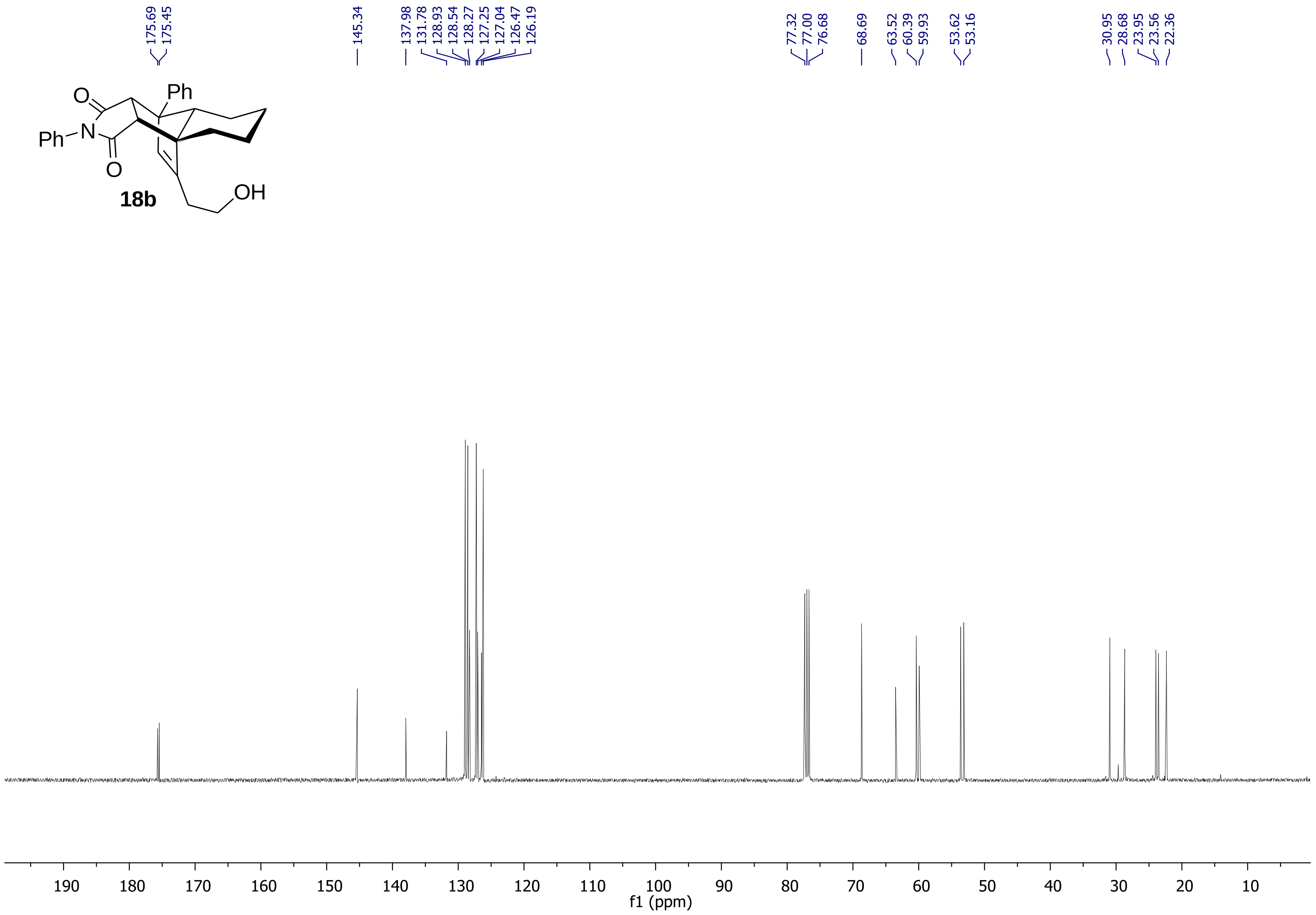

$^{13}C\{^{1}H\}$ NMR ($CDCl_3$, 100.4 MHz) of compound **18b**

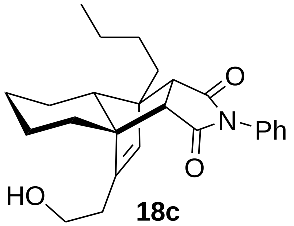

18c

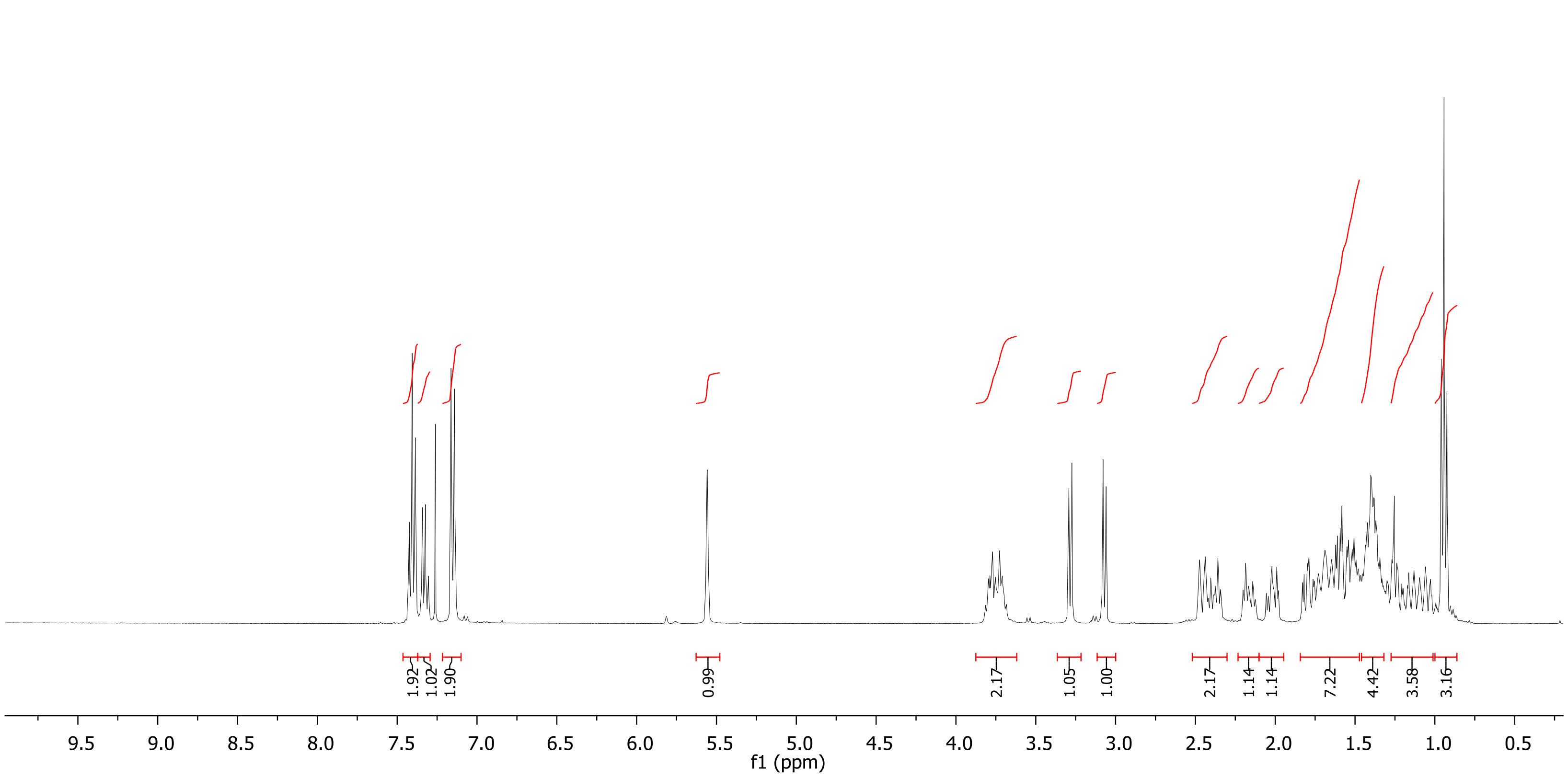

^{1}H NMR ($CDCl_3$, 400 MHz) of compound **18c**

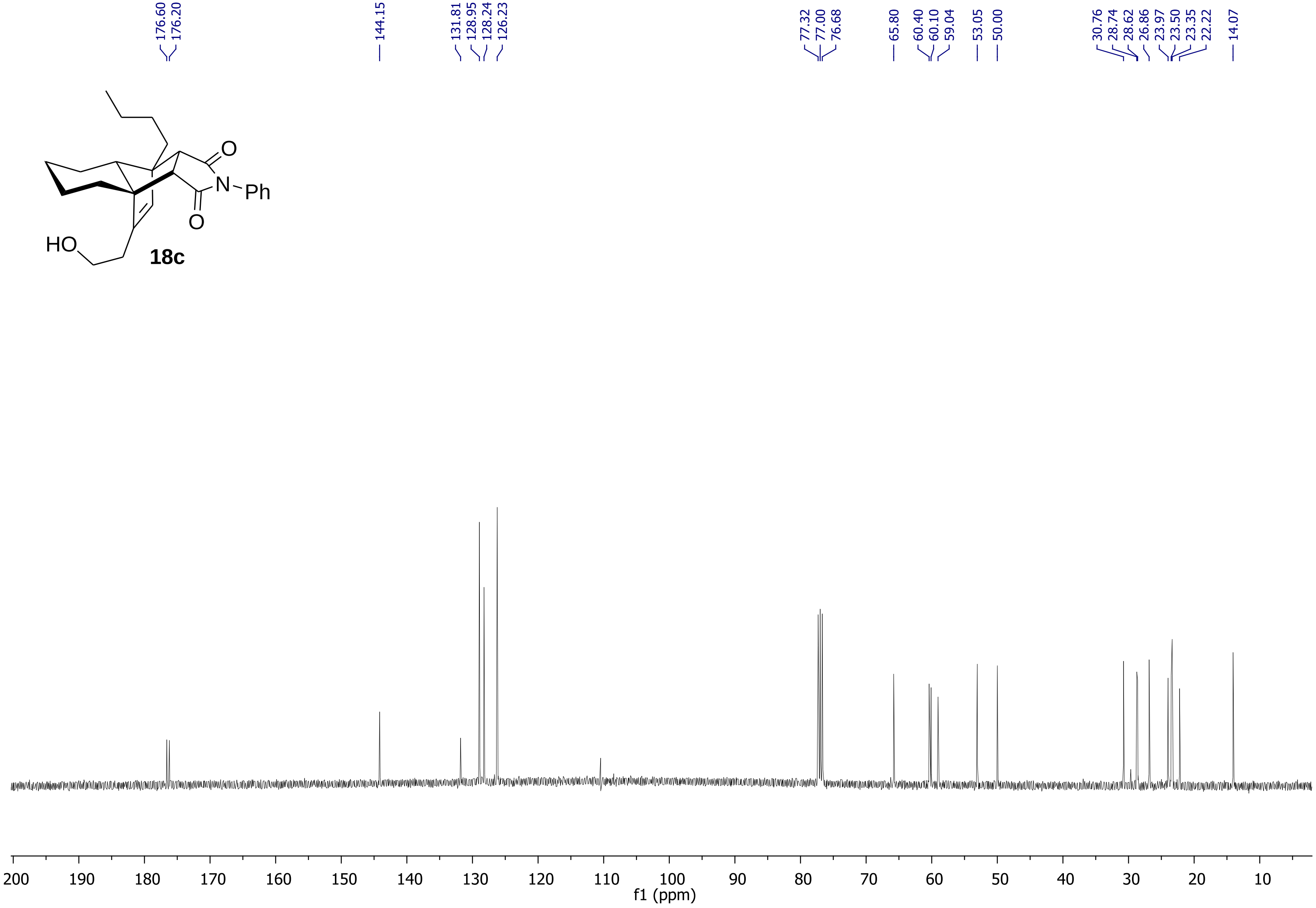

$^{13}C\{^{1}H\}$ NMR ($CDCl_3$, 100.4 MHz) of compound **18c**

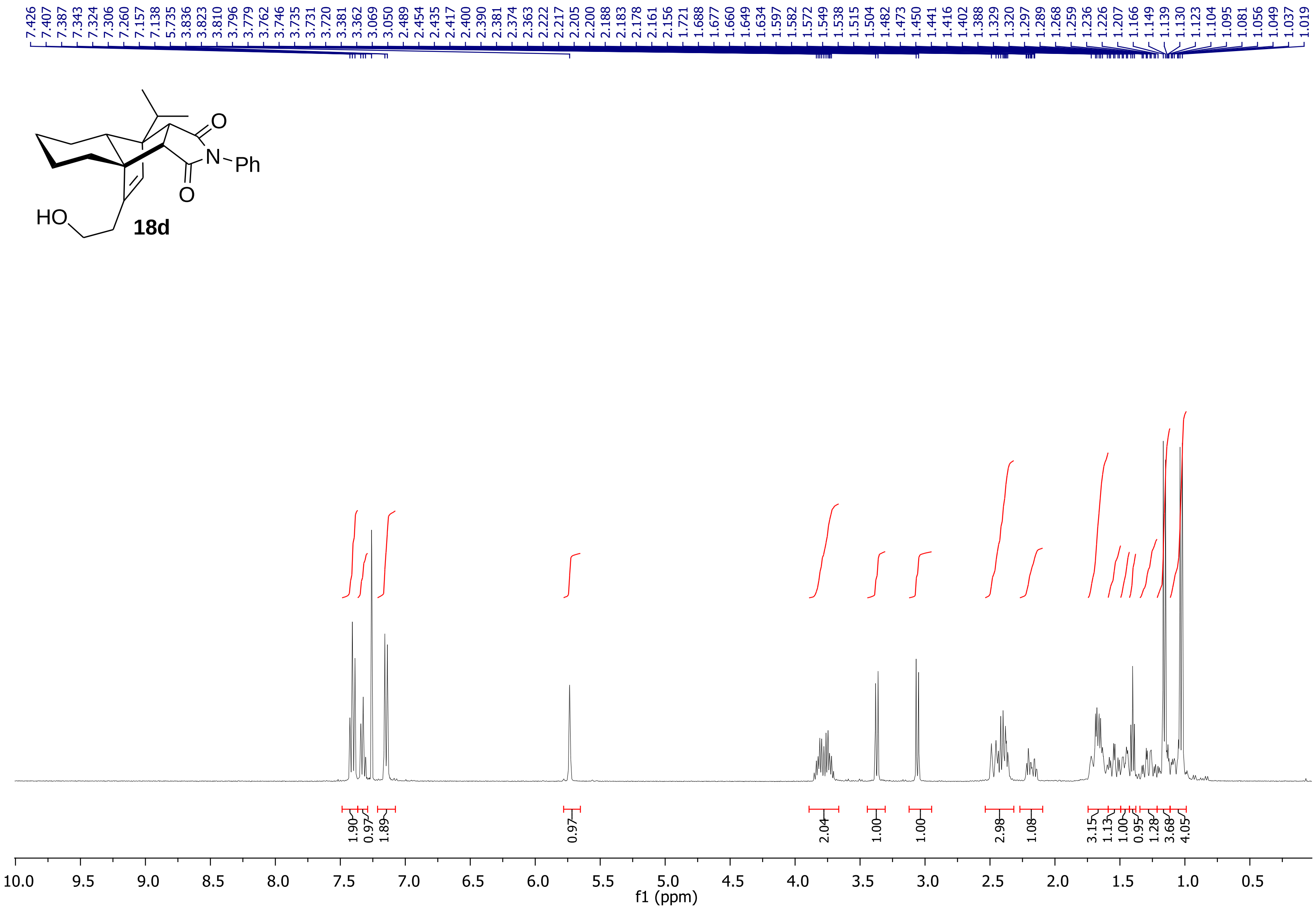

^{1}H NMR ($CDCl_3$, 400 MHz) of compound **18d**

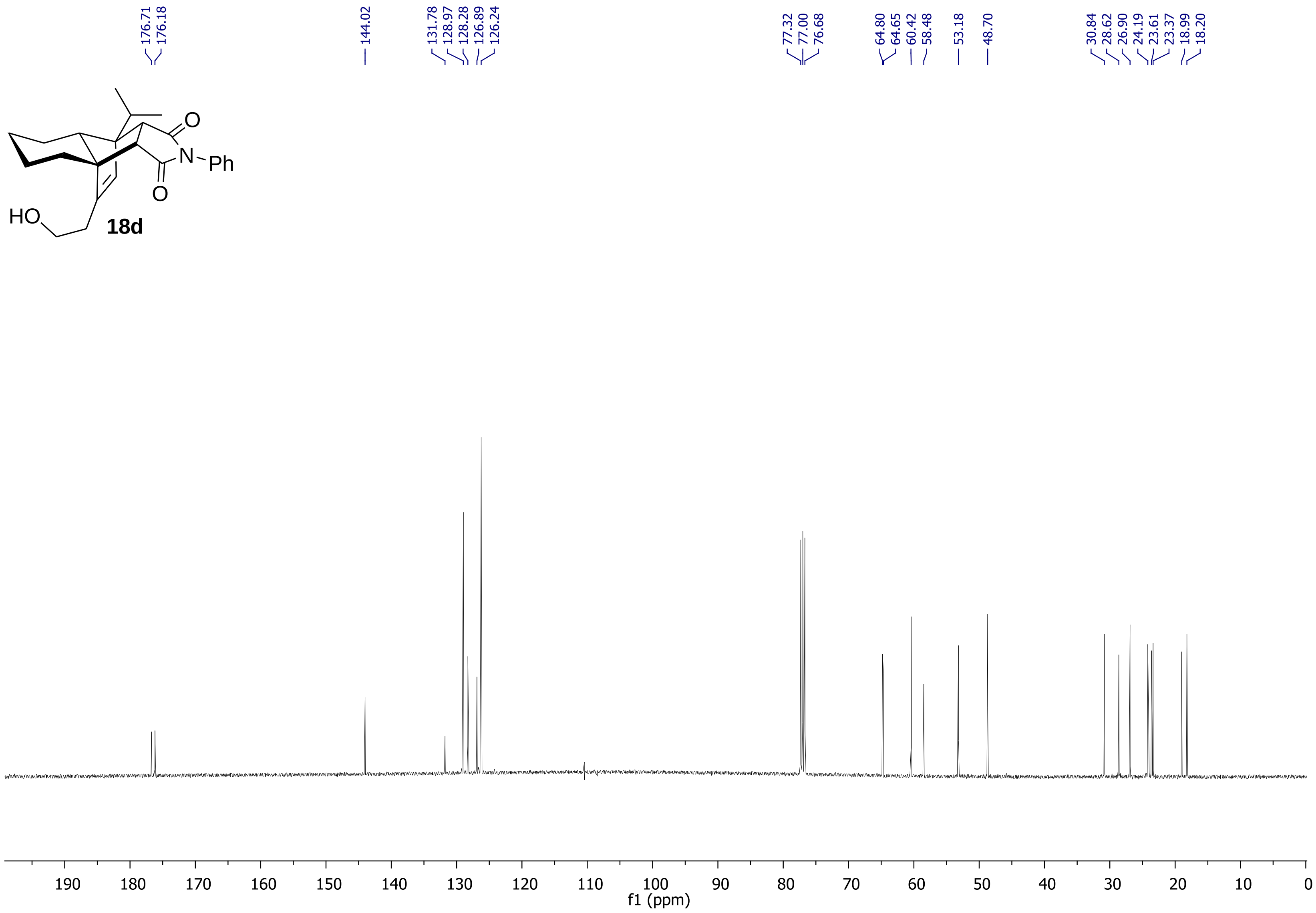

$^{13}C\{^{1}H\}$ NMR ($CDCl_3$, 100.4 MHz) of compound **18d**

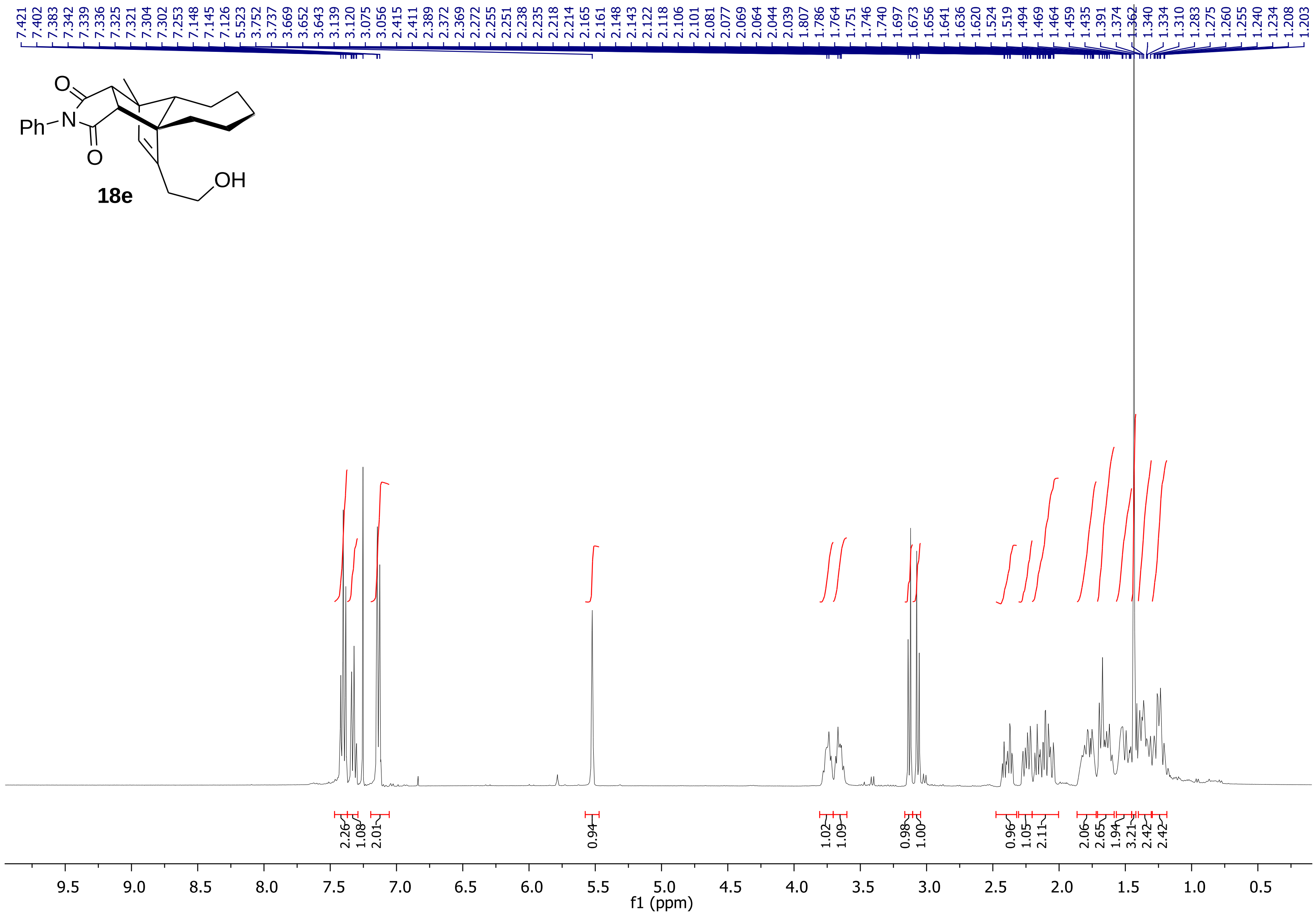

^{1}H NMR ($CDCl_3$, 400 MHz) of compound **18e**

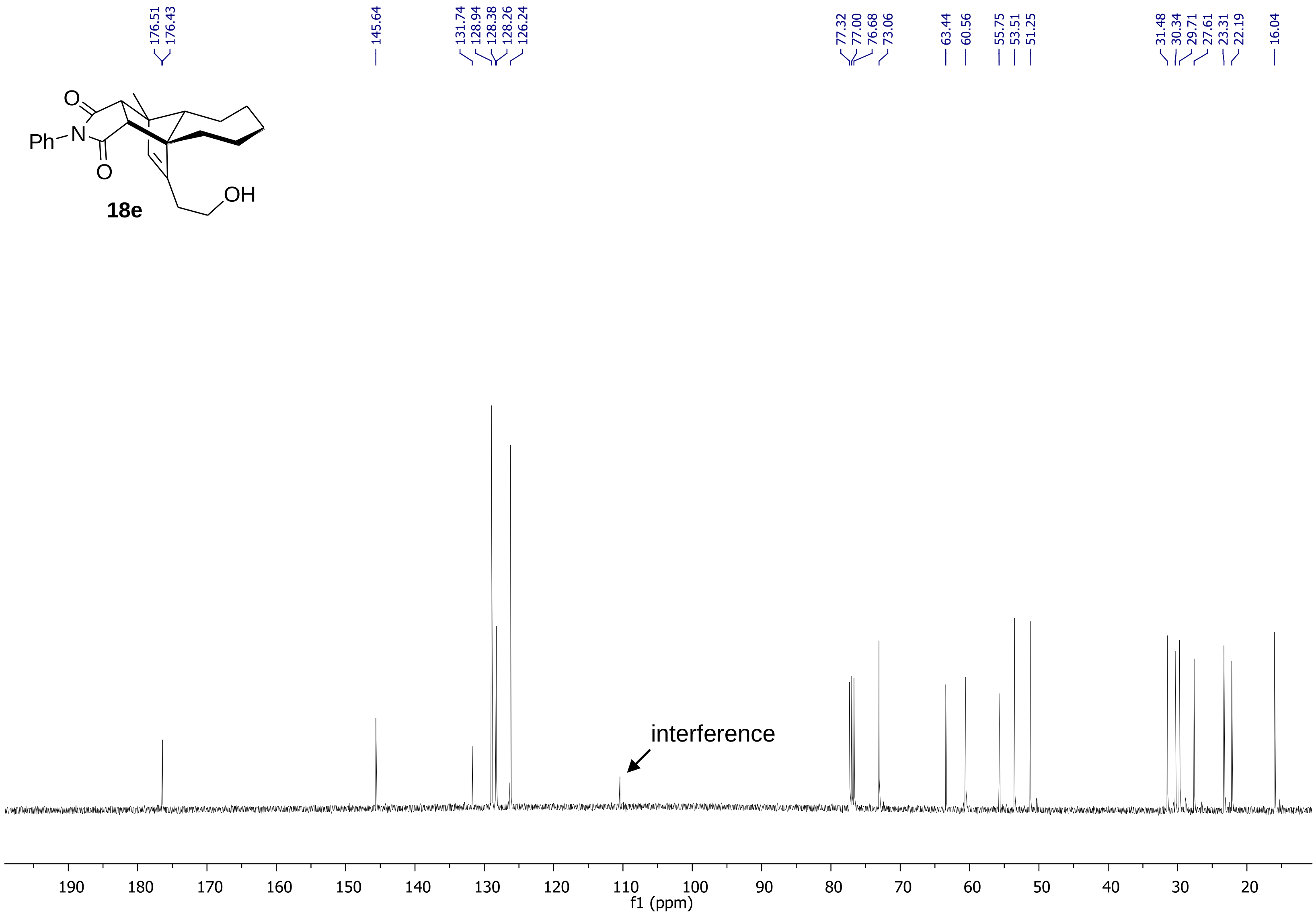

$^{13}C\{^{1}H\}$ NMR ($CDCl_3$, 100.4 MHz) of compound **18e**

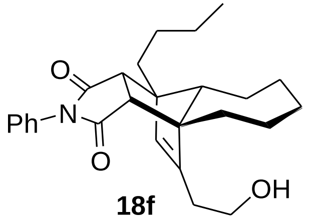

18f

4.6 : 1 mixture of isomers

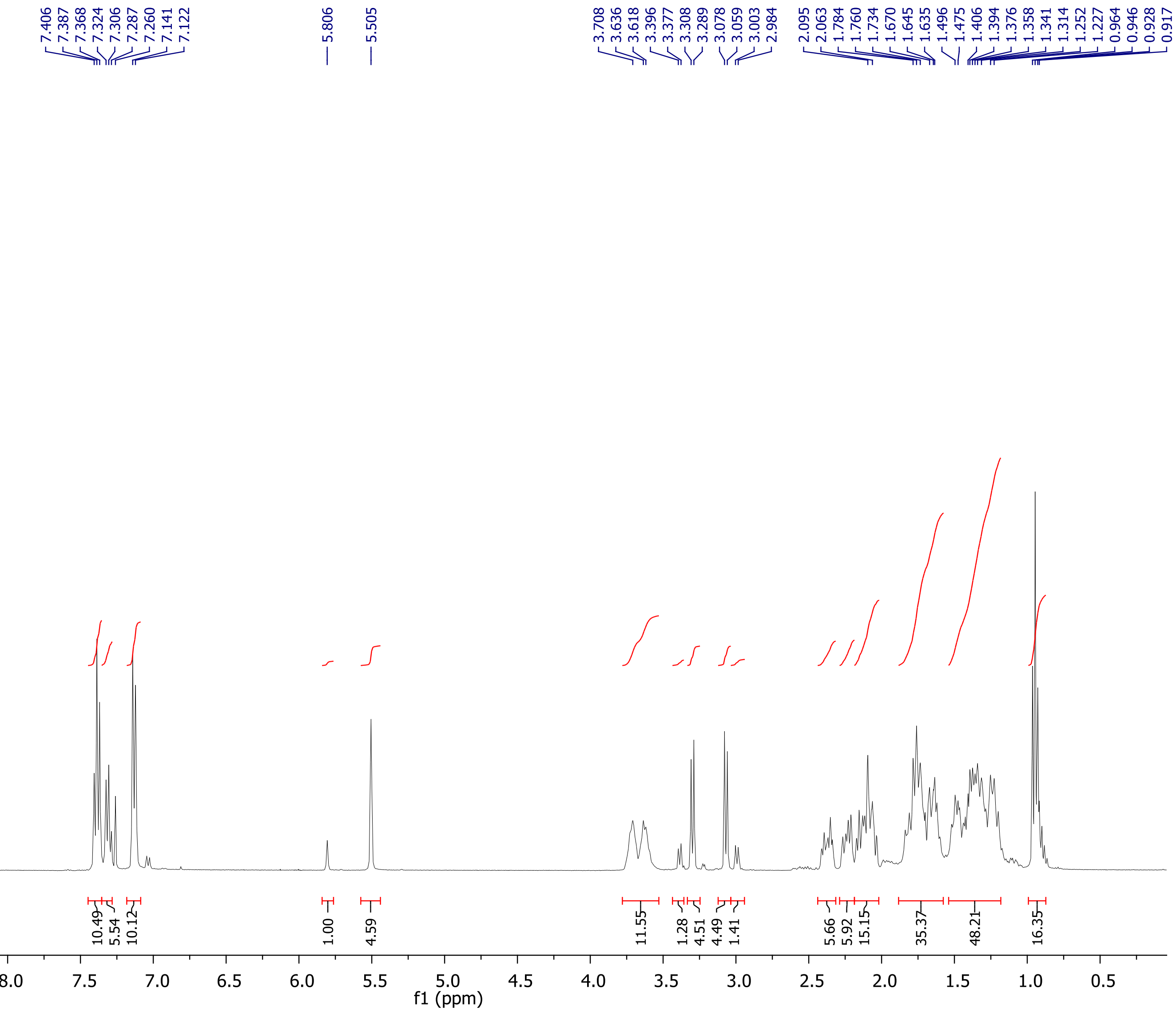

^{1}H NMR ($CDCl_3$, 400 MHz) of compound **18f**

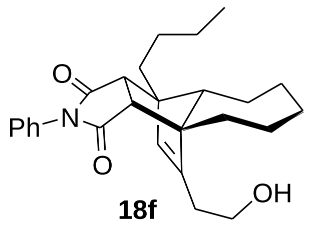

18f

mixture of isomers

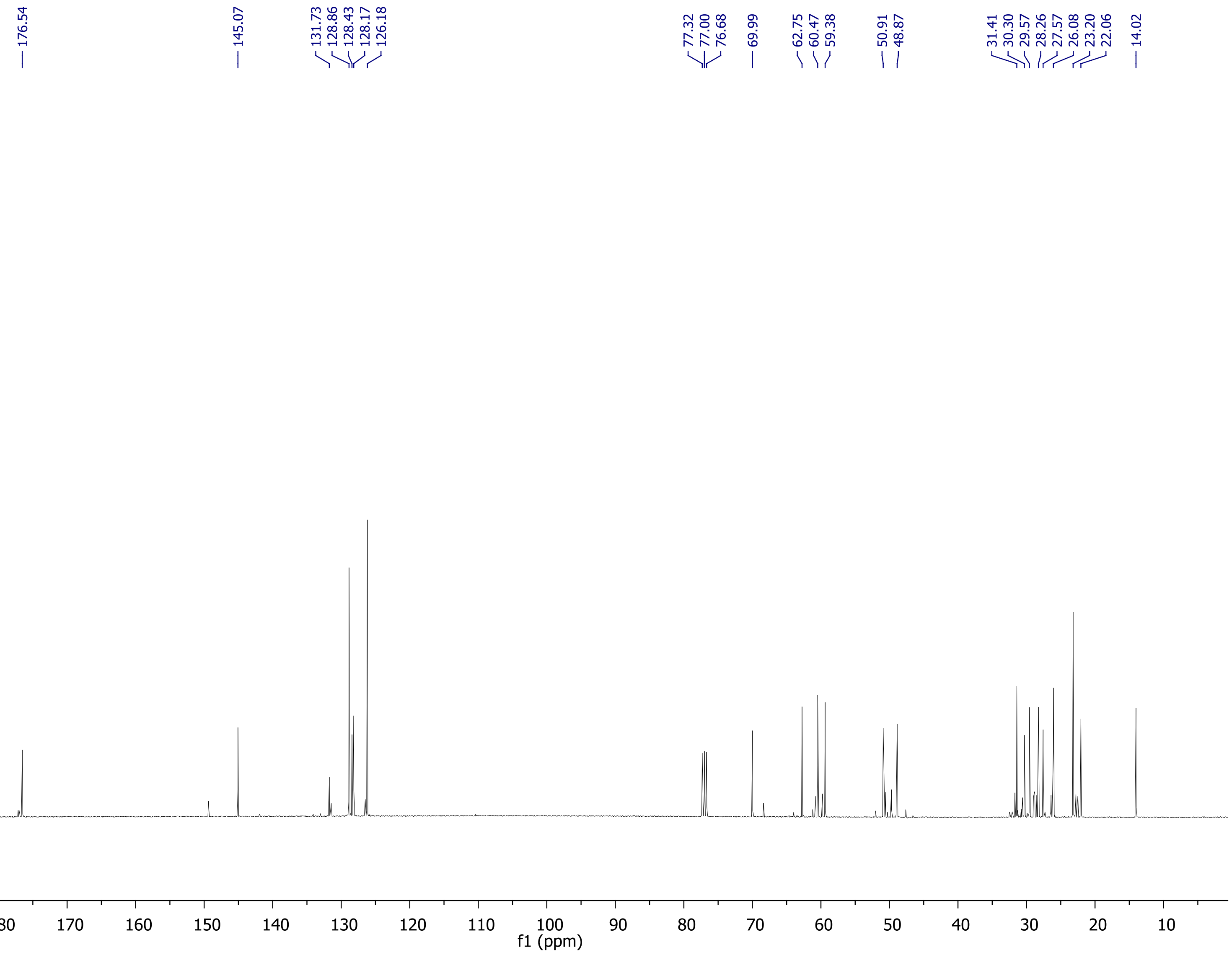

$^{13}C\{^{1}H\}$ NMR ($CDCl_3$, 100.4 MHz) of compound **18f**

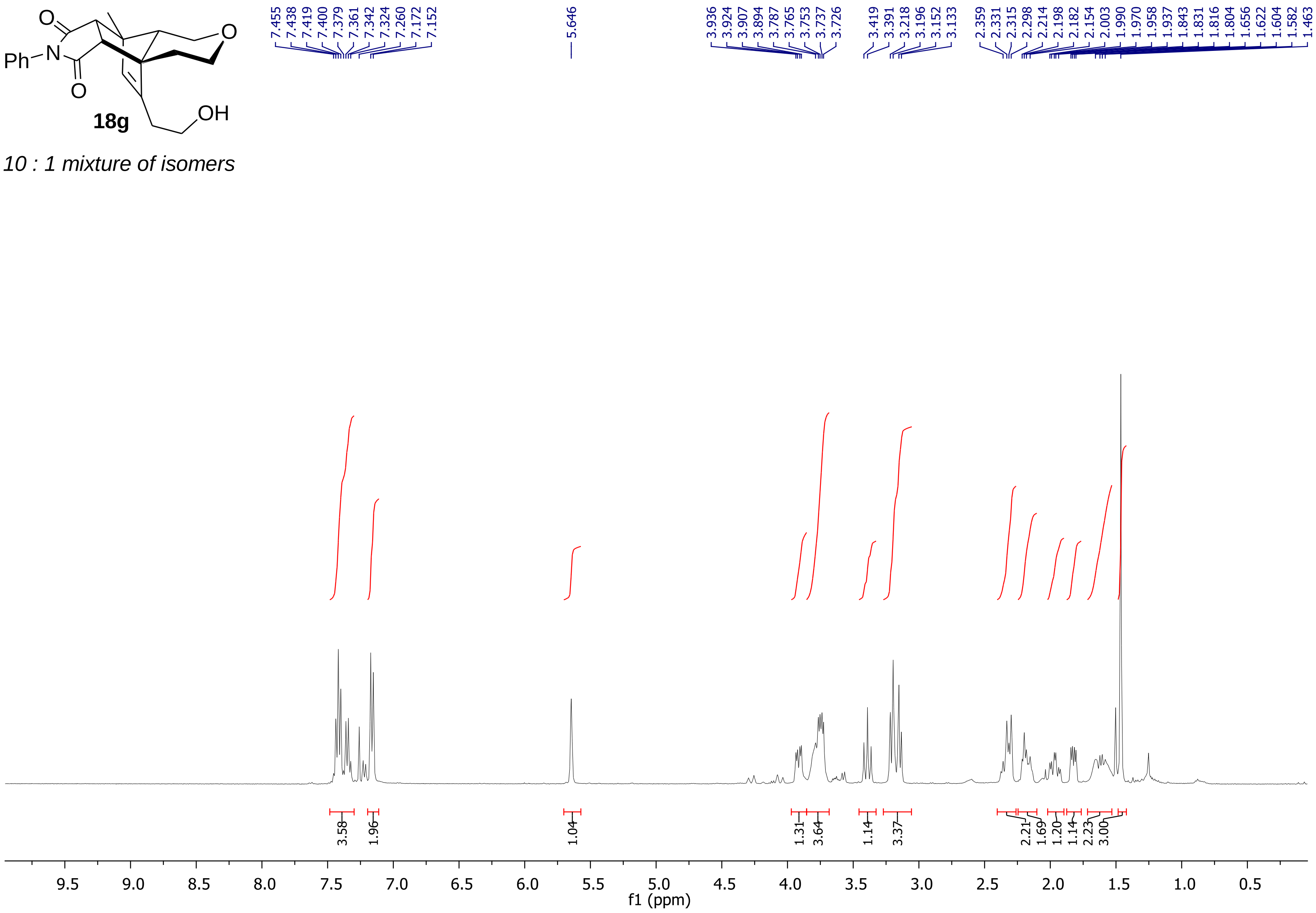

^{1}H NMR ($CDCl_3$, 400 MHz) of compound **18g**

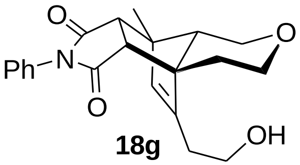

18g

mixture of isomers

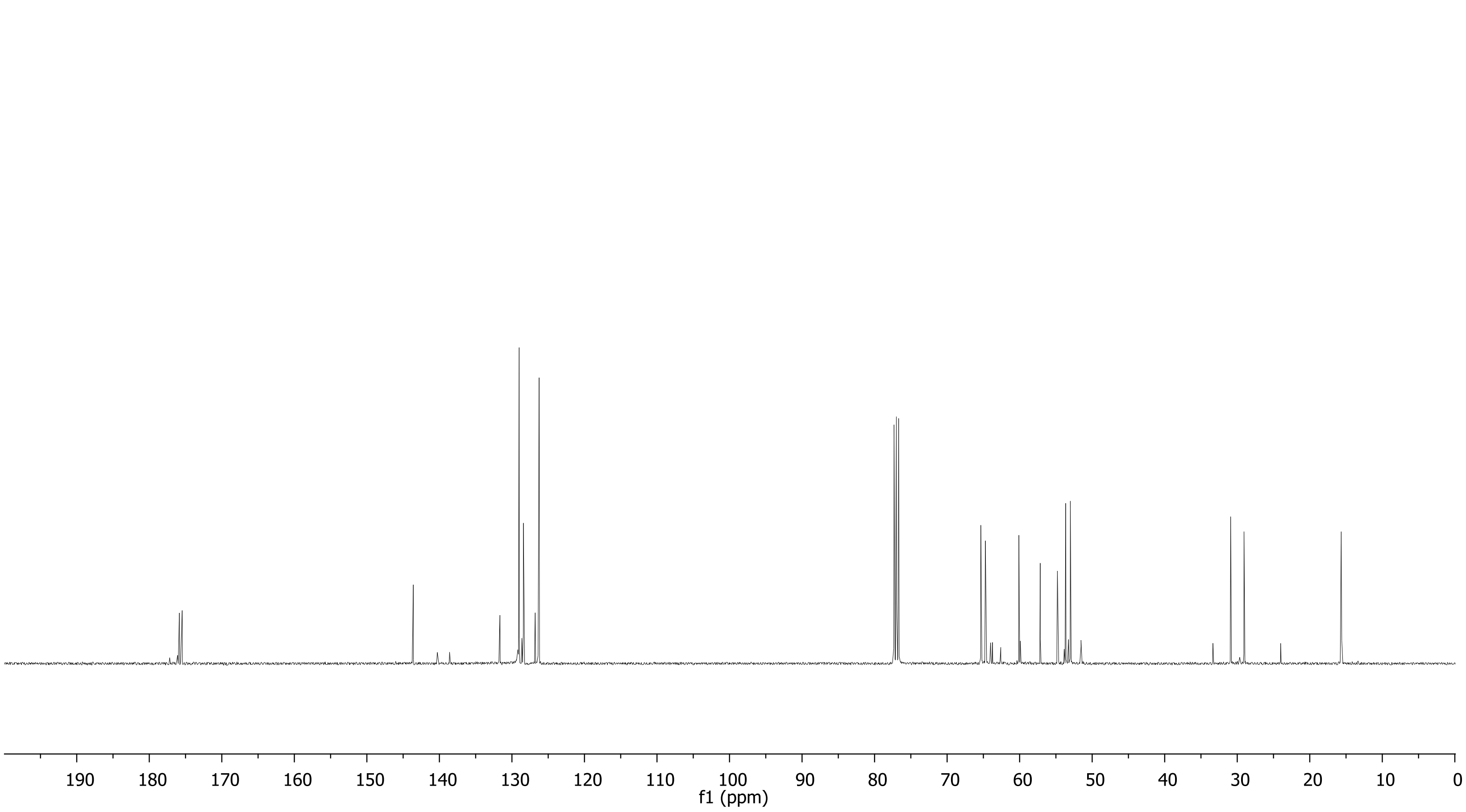

$^{13}C\{^{1}H\}$ NMR ($CDCl_3$, 100.4 MHz) of compound **18g**

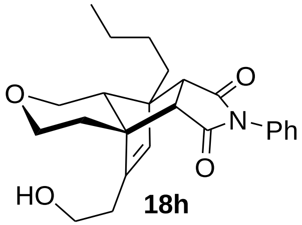

18h

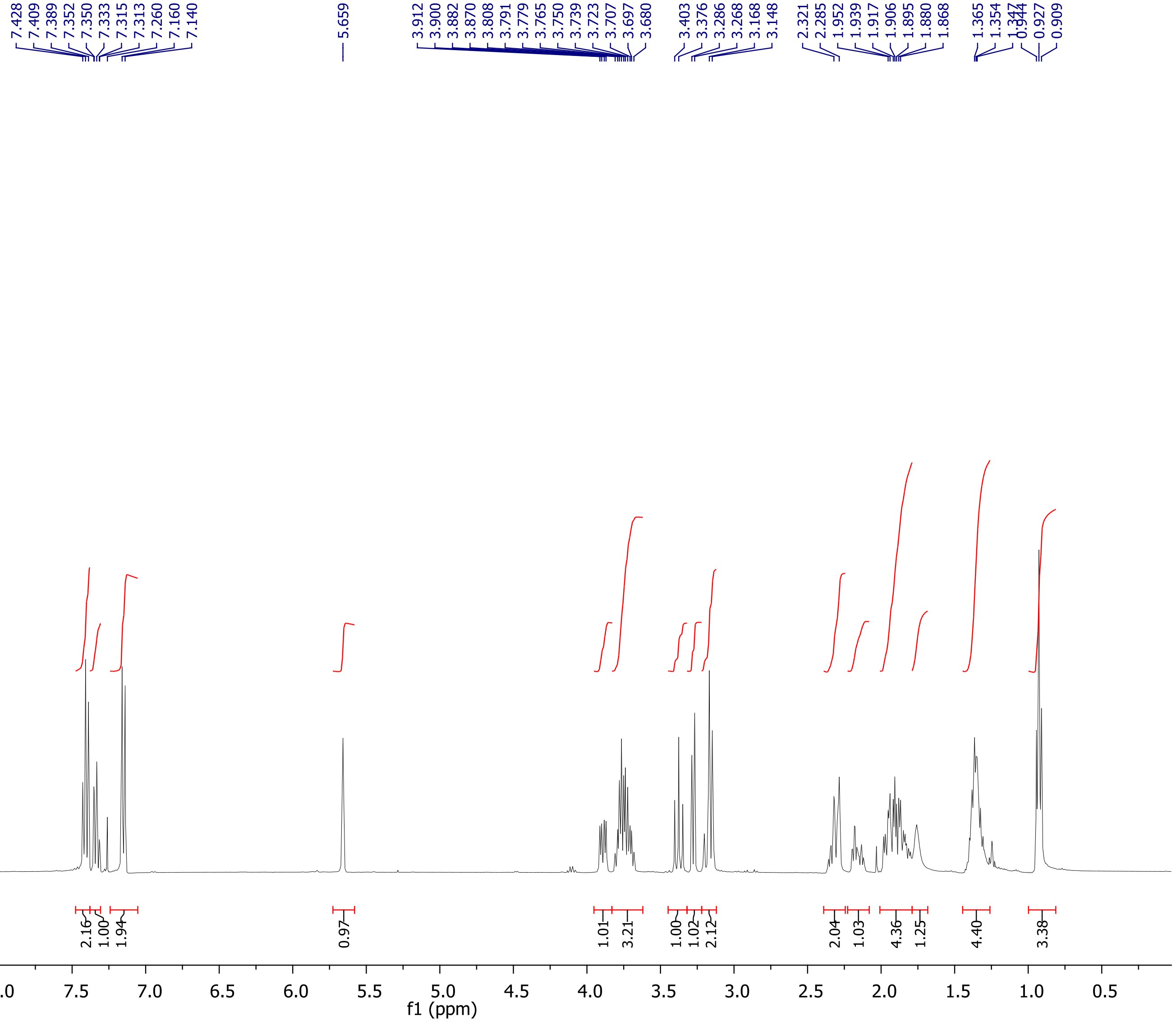

^{1}H NMR ($CDCl_3$, 400 MHz) of compound **18h**

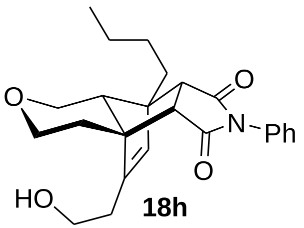

18h

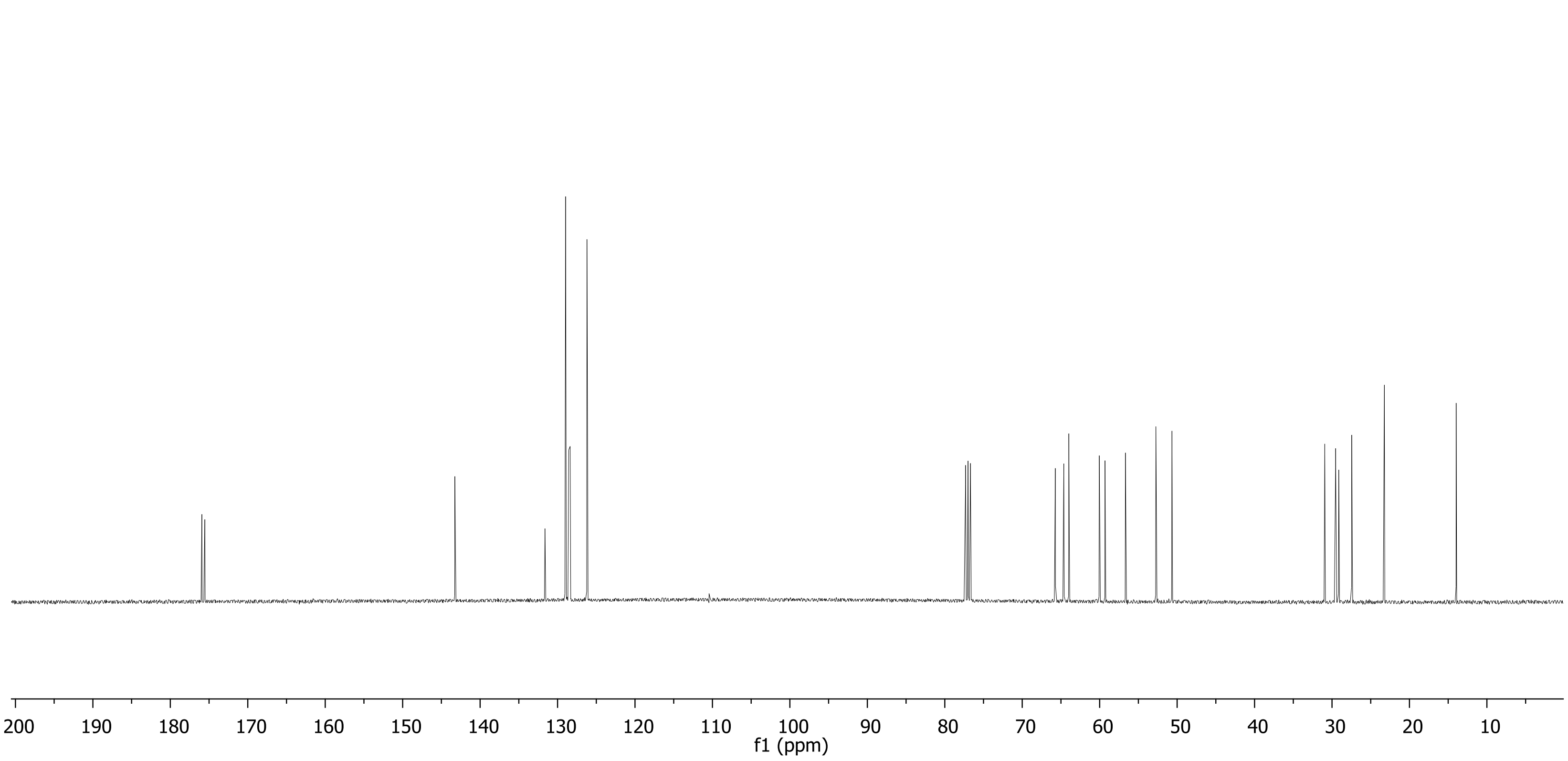

$^{13}C\{^{1}H\}$ NMR ($CDCl_3$, 100.4 MHz) of compound **18h**

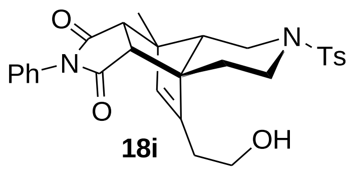

18i

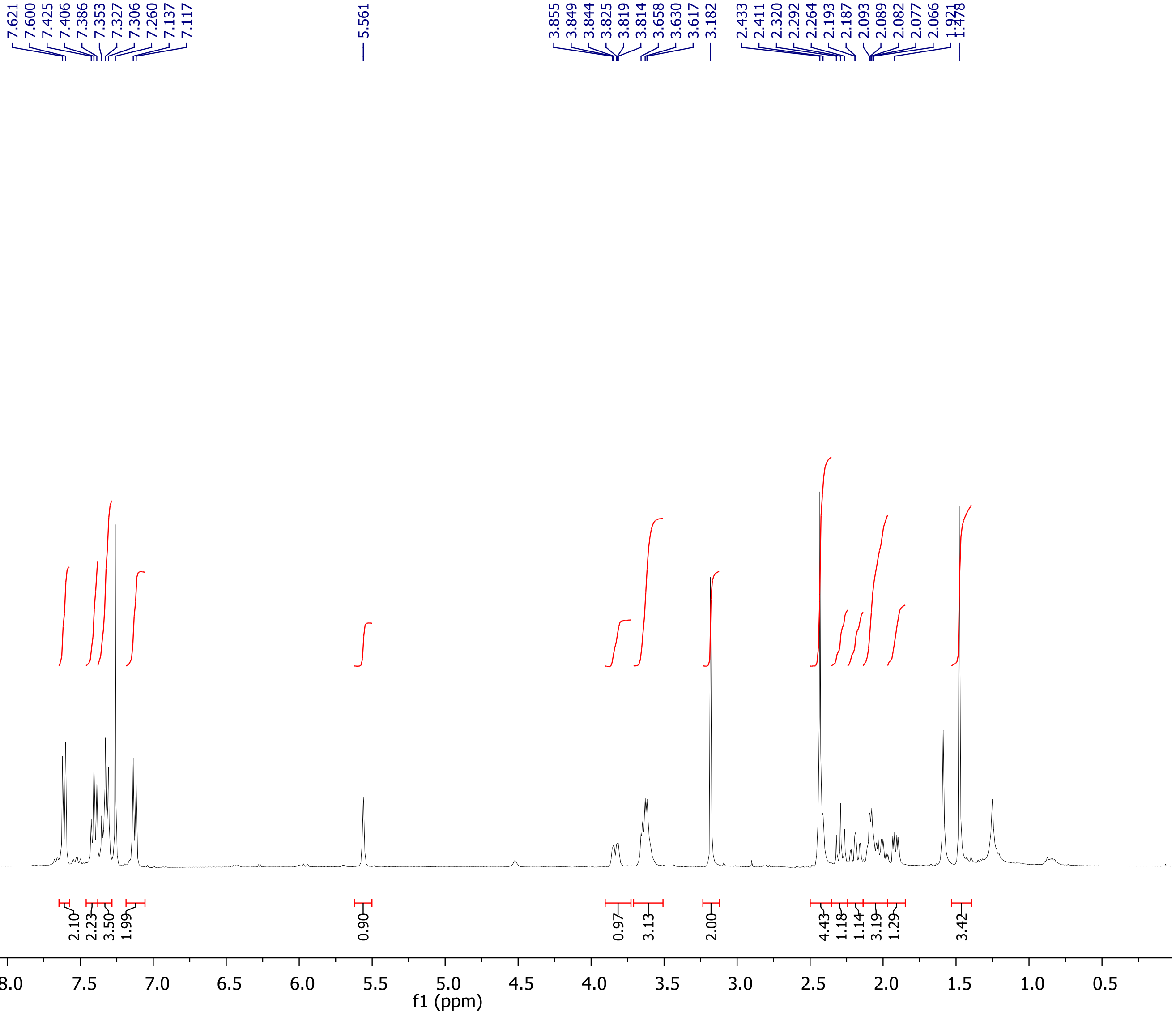

^{1}H NMR ($CDCl_3$, 400 MHz) of compound **18i**

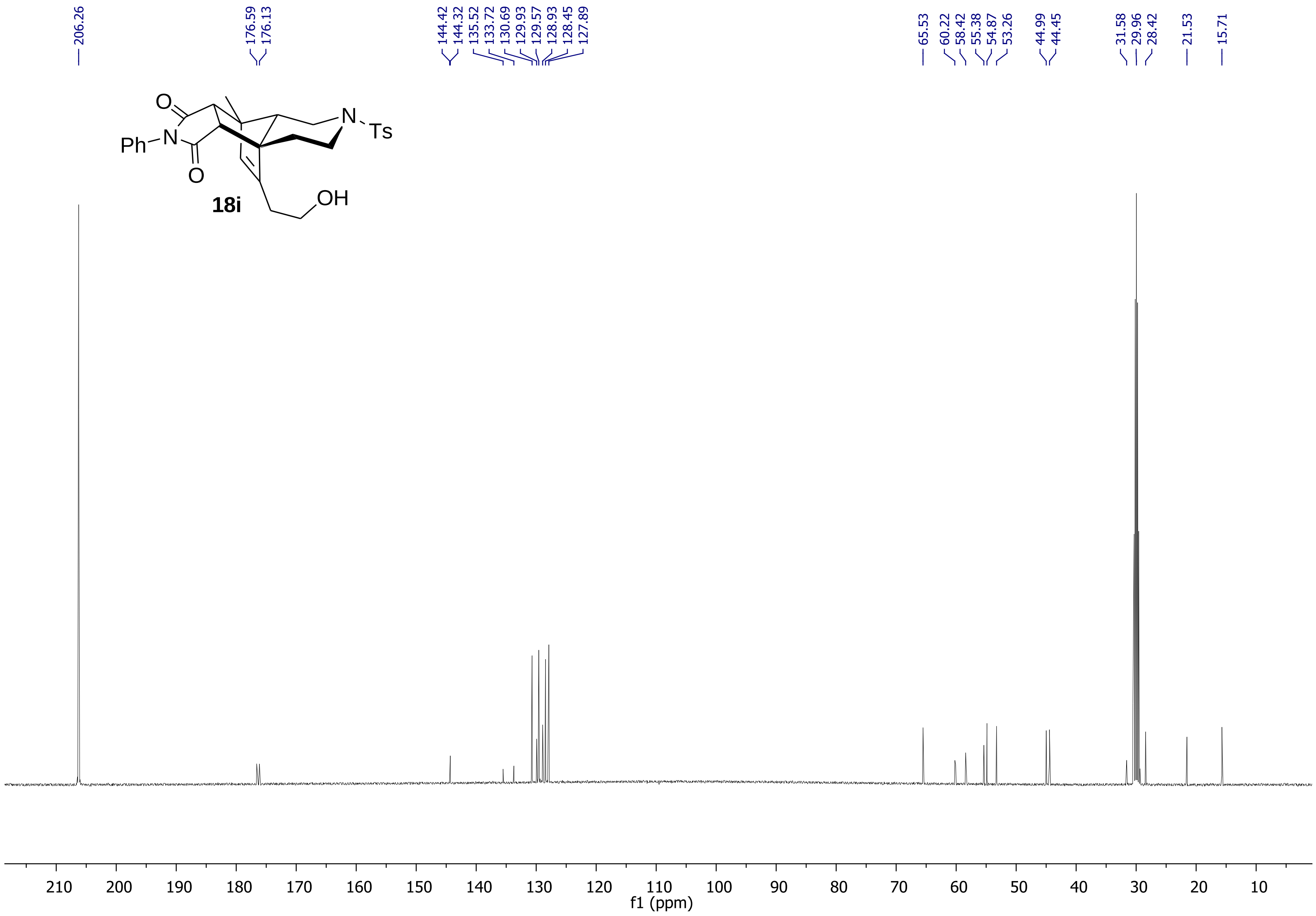

$^{13}C\{^{1}H\}$ NMR (acetone-d6, 100.4 MHz) of compound **18i**

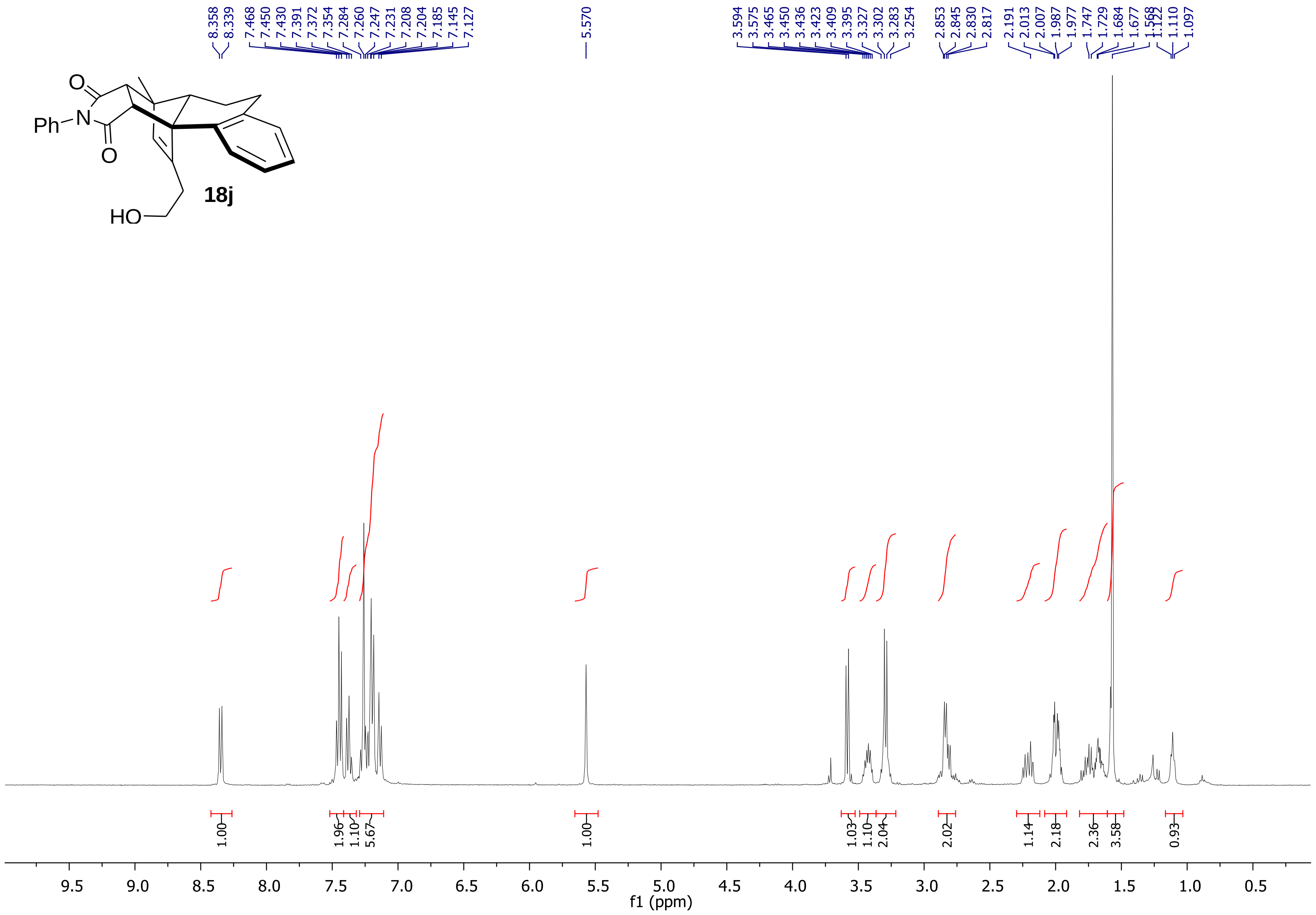

^{1}H NMR ($CDCl_3$, 400 MHz) of compound **18j**

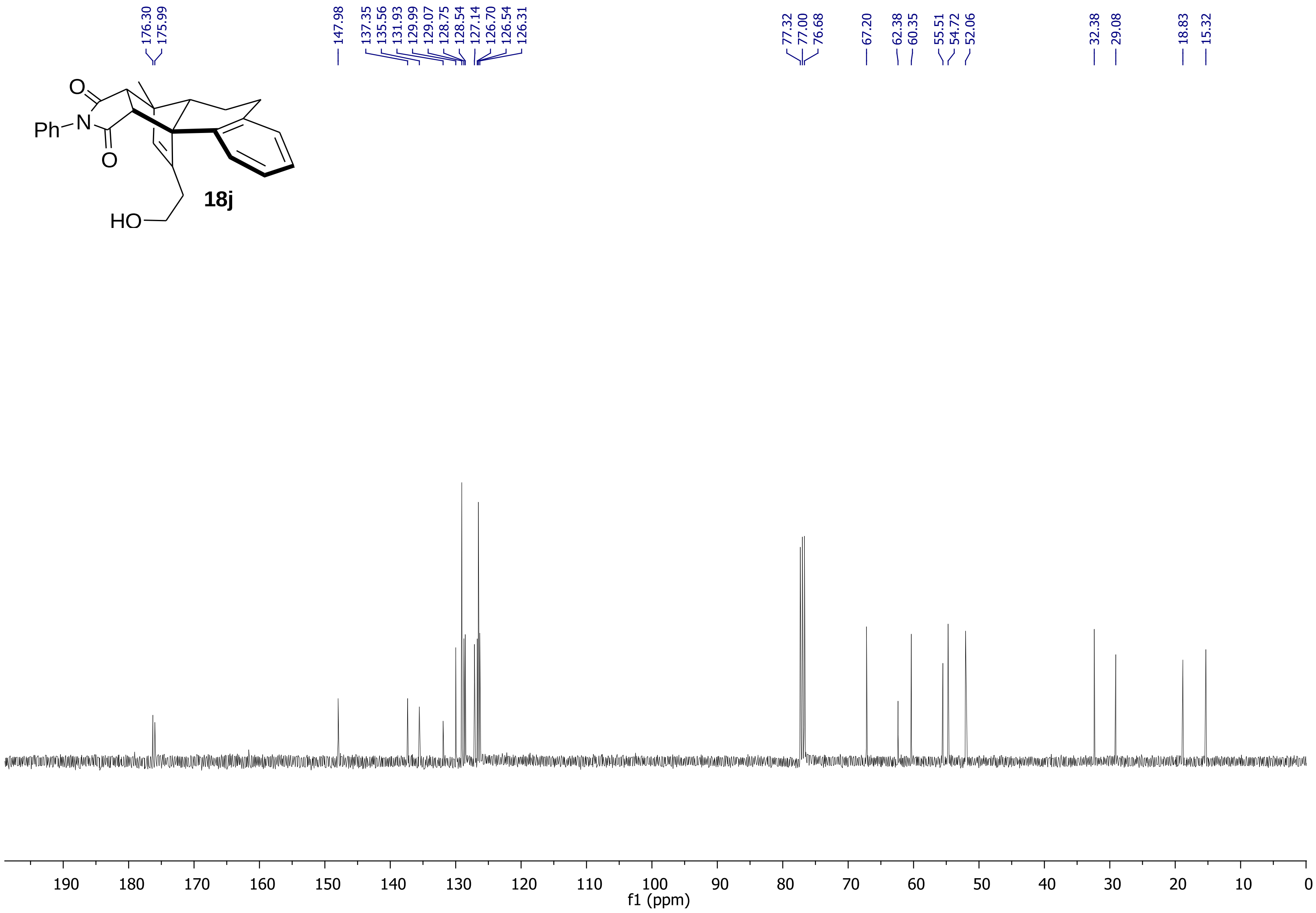

$^{13}C\{^{1}H\}$ NMR ($CDCl_3$, 100.4 MHz) of compound **18j**

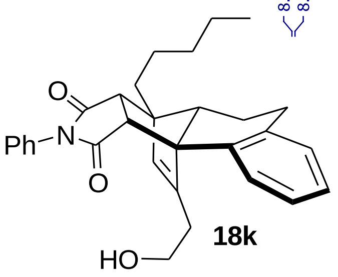

18k

8 : 1 mixture of diastereoisomers

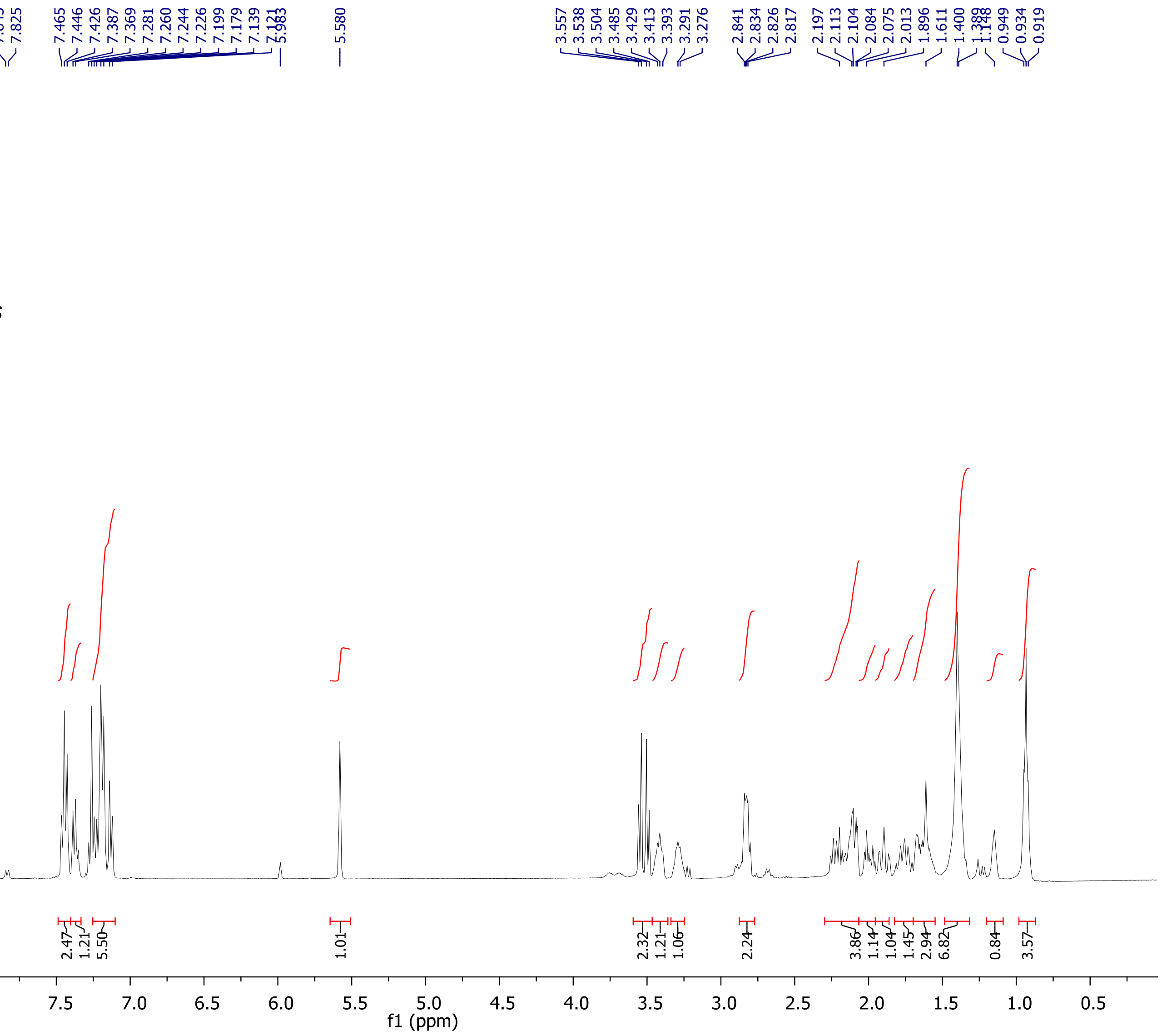

^{1}H NMR ($CDCl_3$, 400 MHz) of compound **18k**

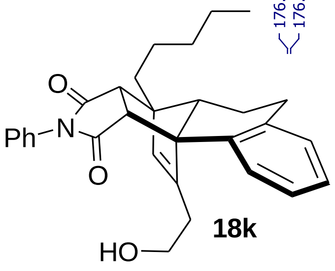

18k

mixture of diastereoisomers

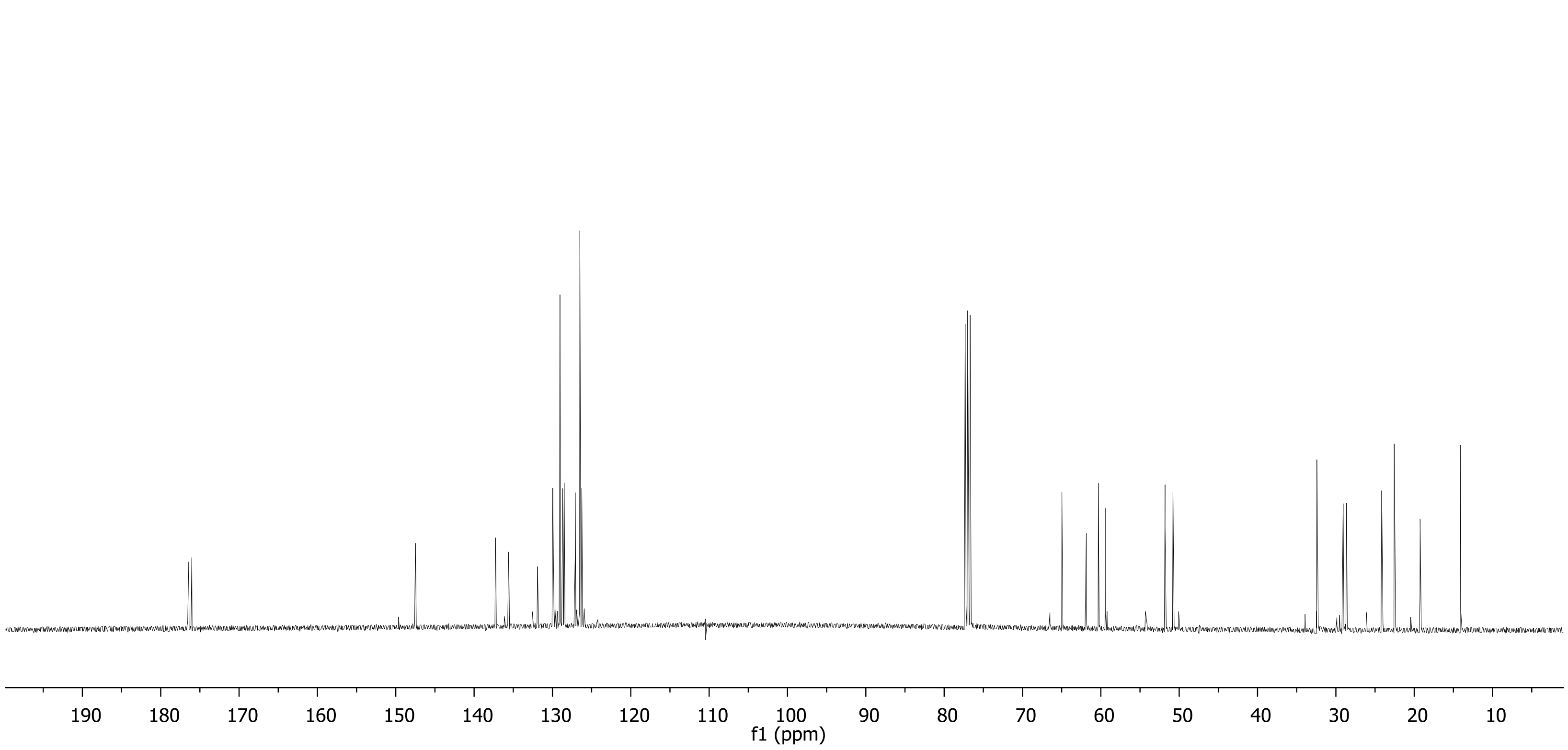

$^{13}C\{^{1}H\}$ NMR ($CDCl_3$, 100.4 MHz) of compound **18k**

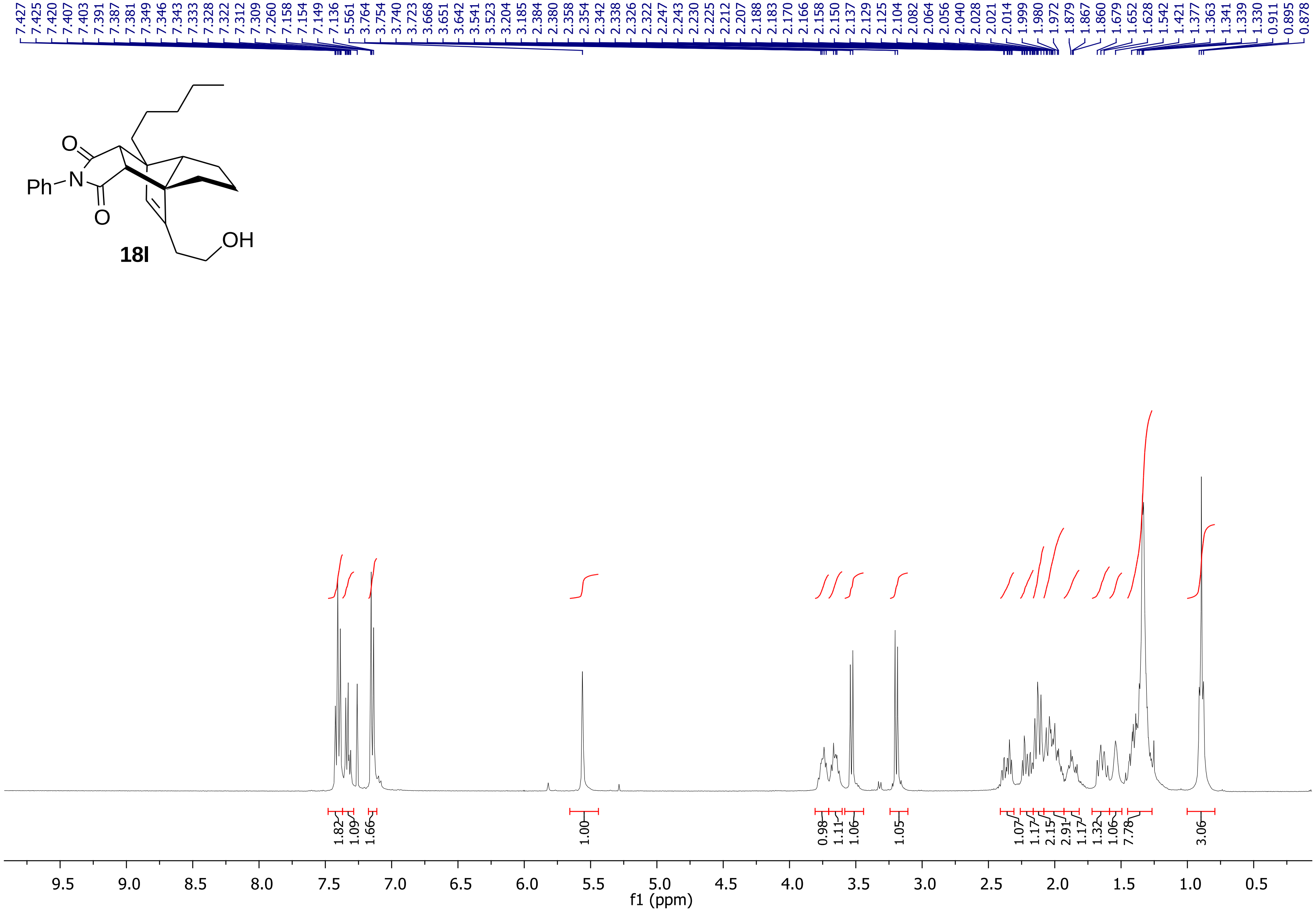

^{1}H NMR ($CDCl_3$, 400 MHz) of compound **18l**

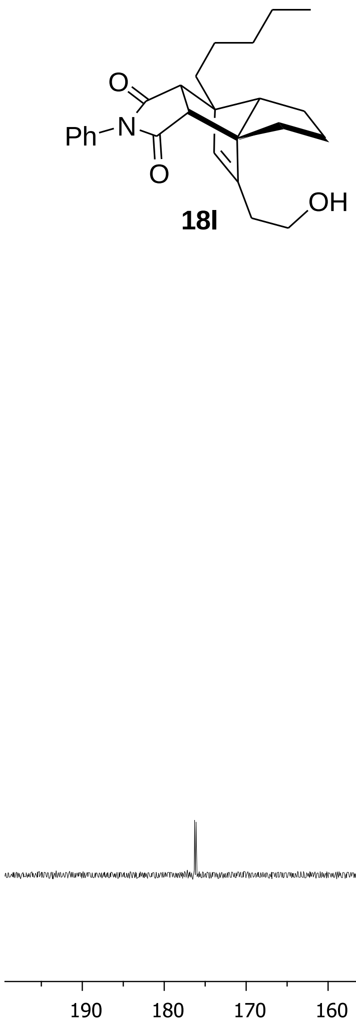

18l

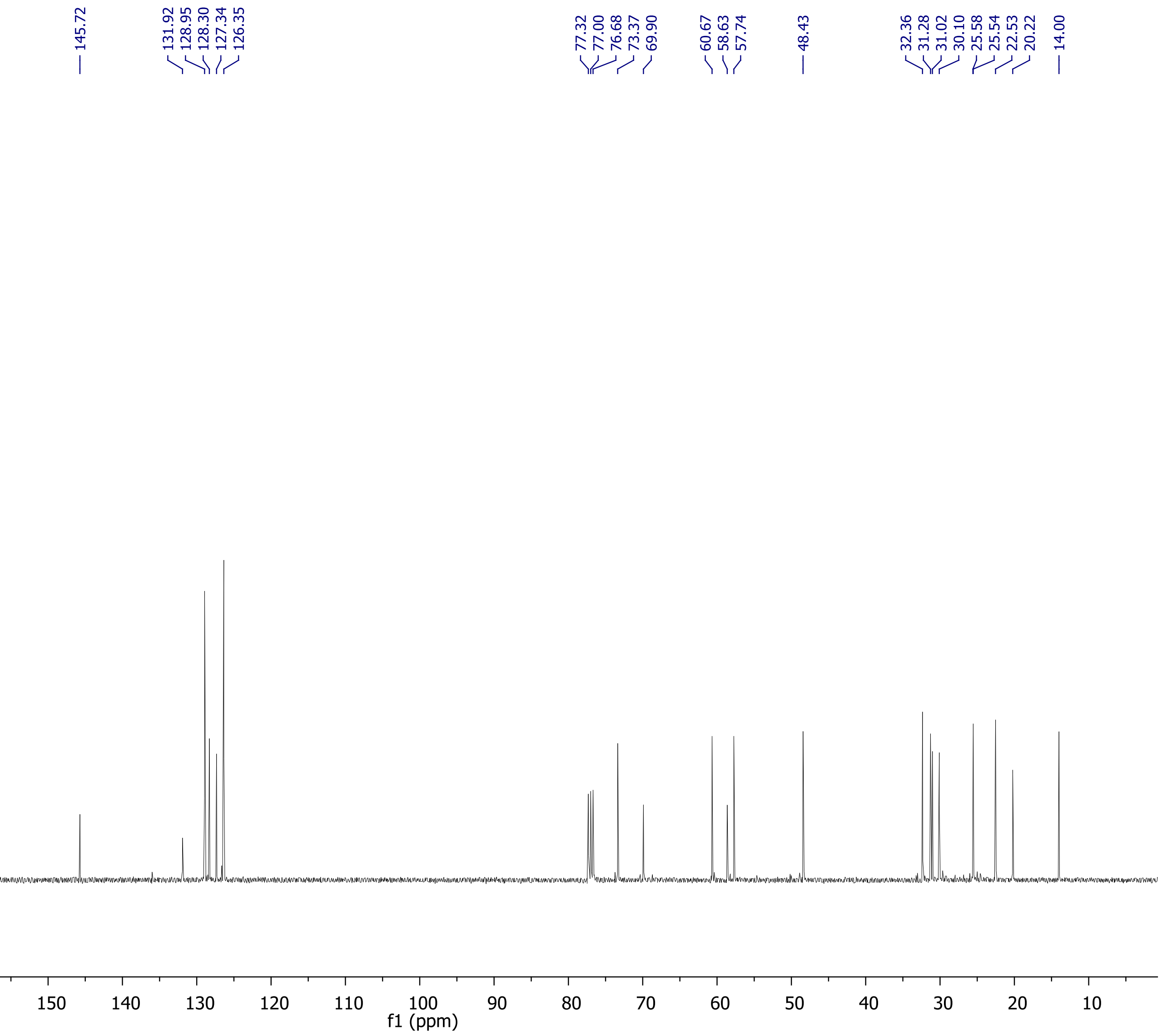

$^{13}C\{^{1}H\}$ NMR ($CDCl_3$, 100.4 MHz) of compound **18l**

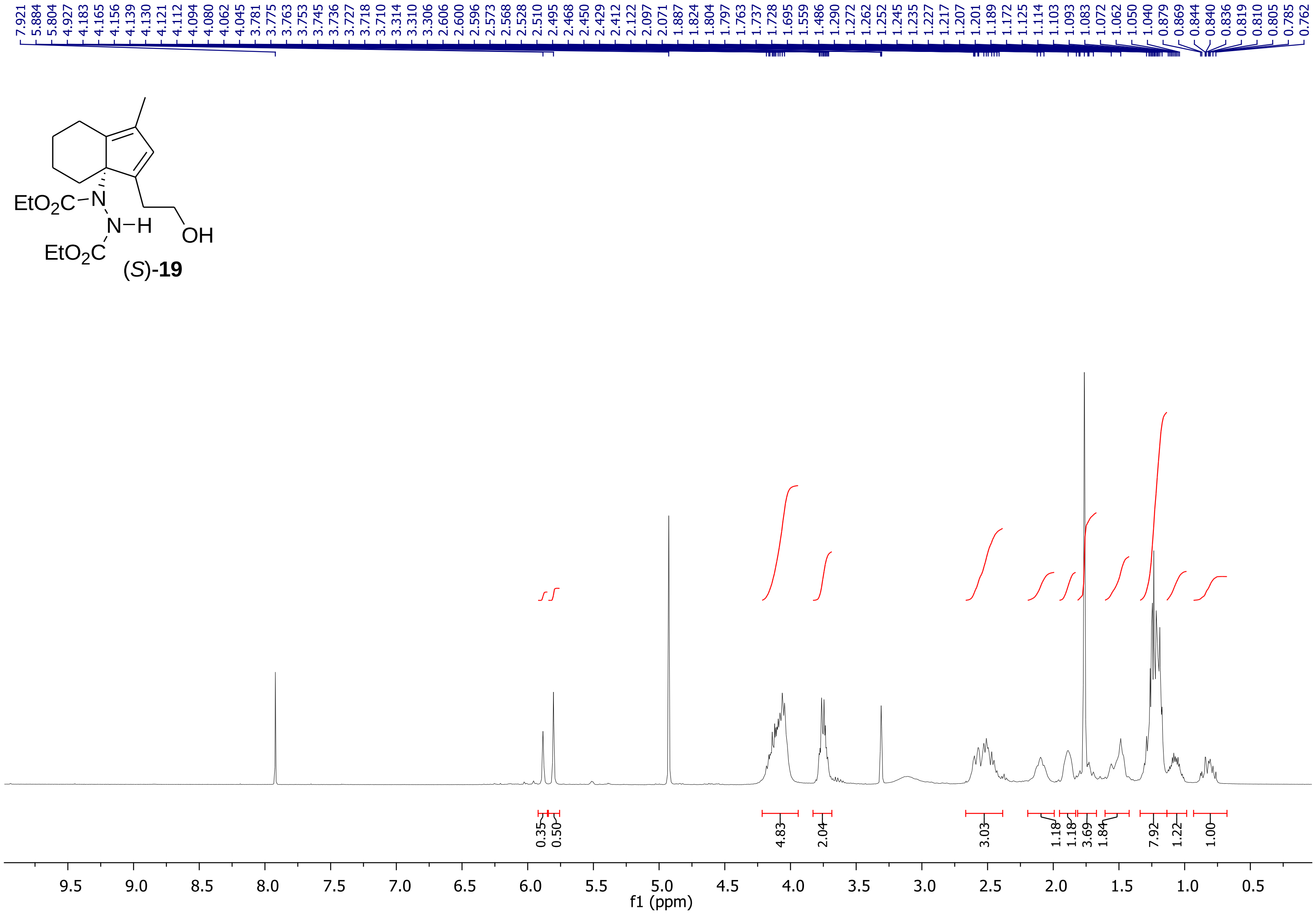

^{1}H NMR (CD_3OD, 400 MHz) of compound (*S*)-**19**

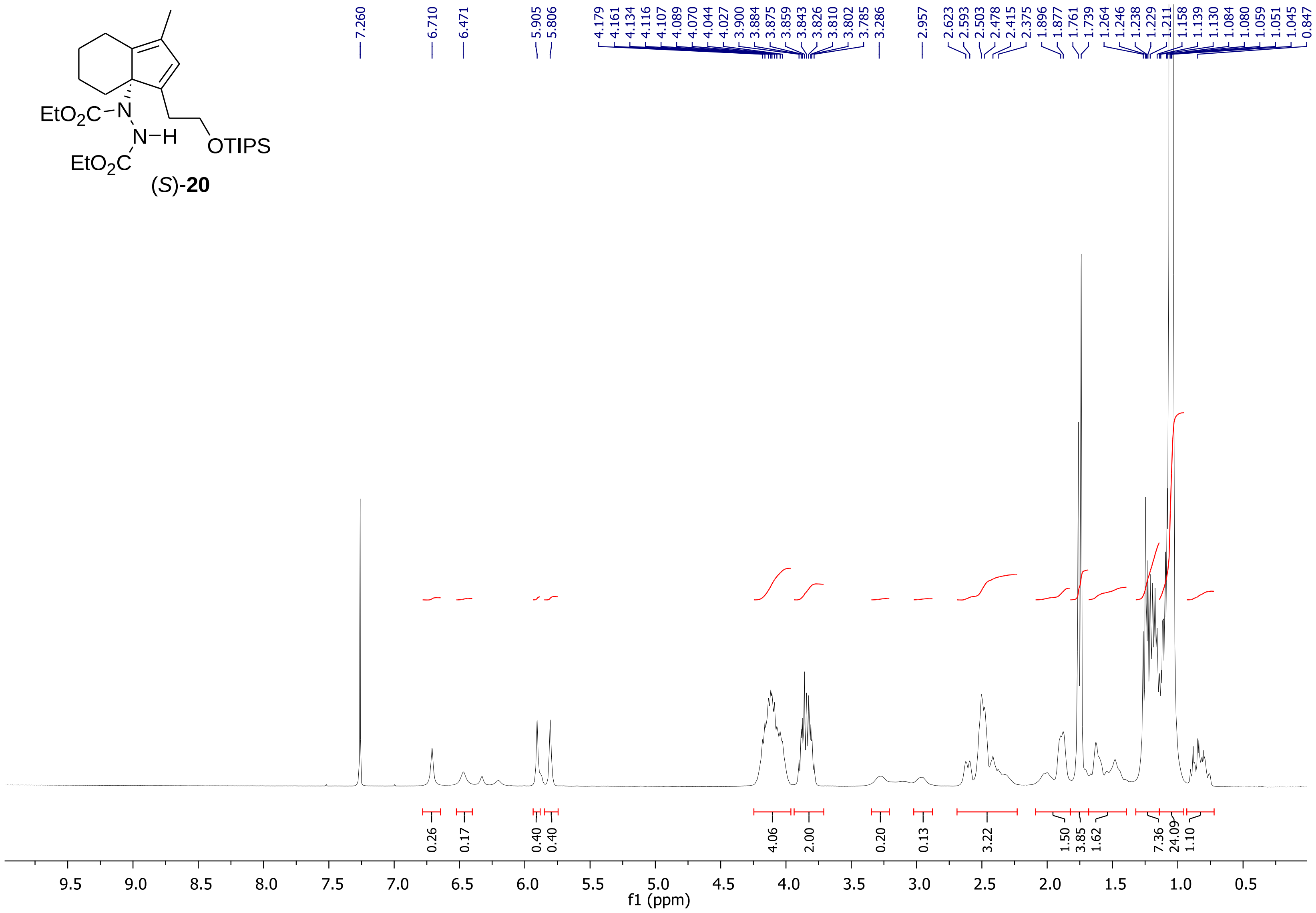

^{1}H NMR ($CDCl_3$, 400 MHz) of compound (*S*)-**20**

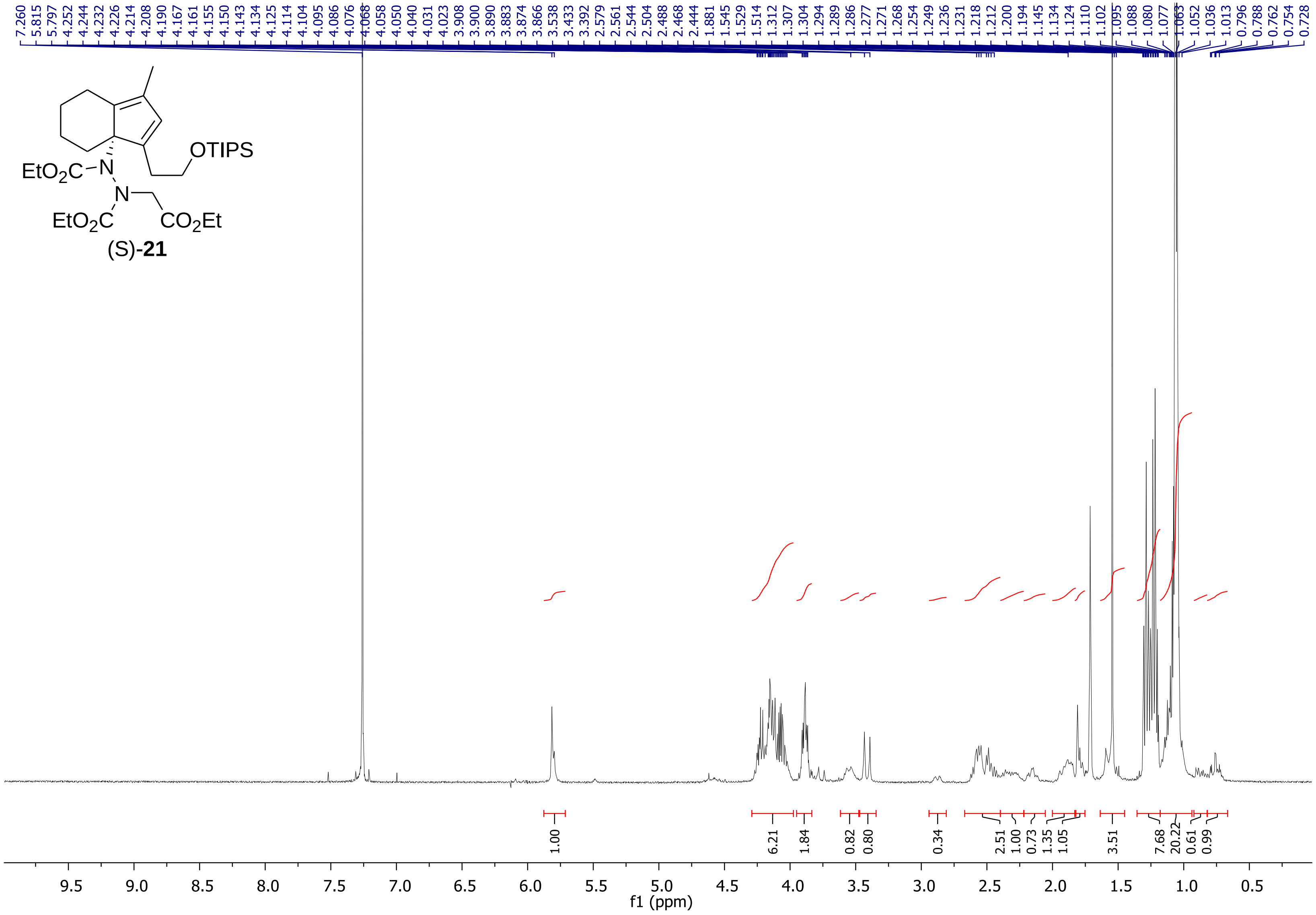

^{1}H NMR ($CDCl_3$, 400 MHz) of compound (*S*)-**21**

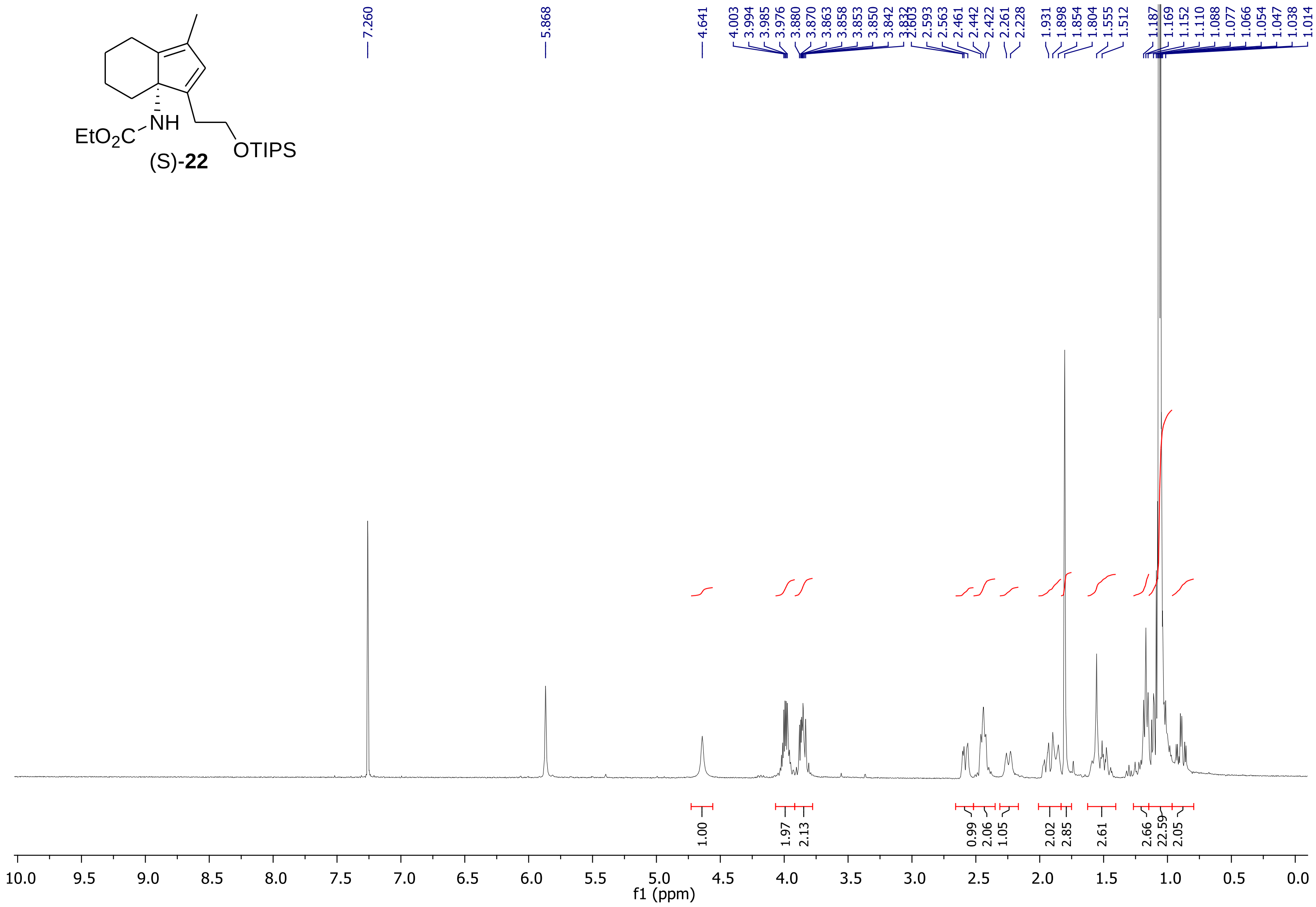

^{1}H NMR ($CDCl_3$, 400 MHz) of compound (*S*)-**22**

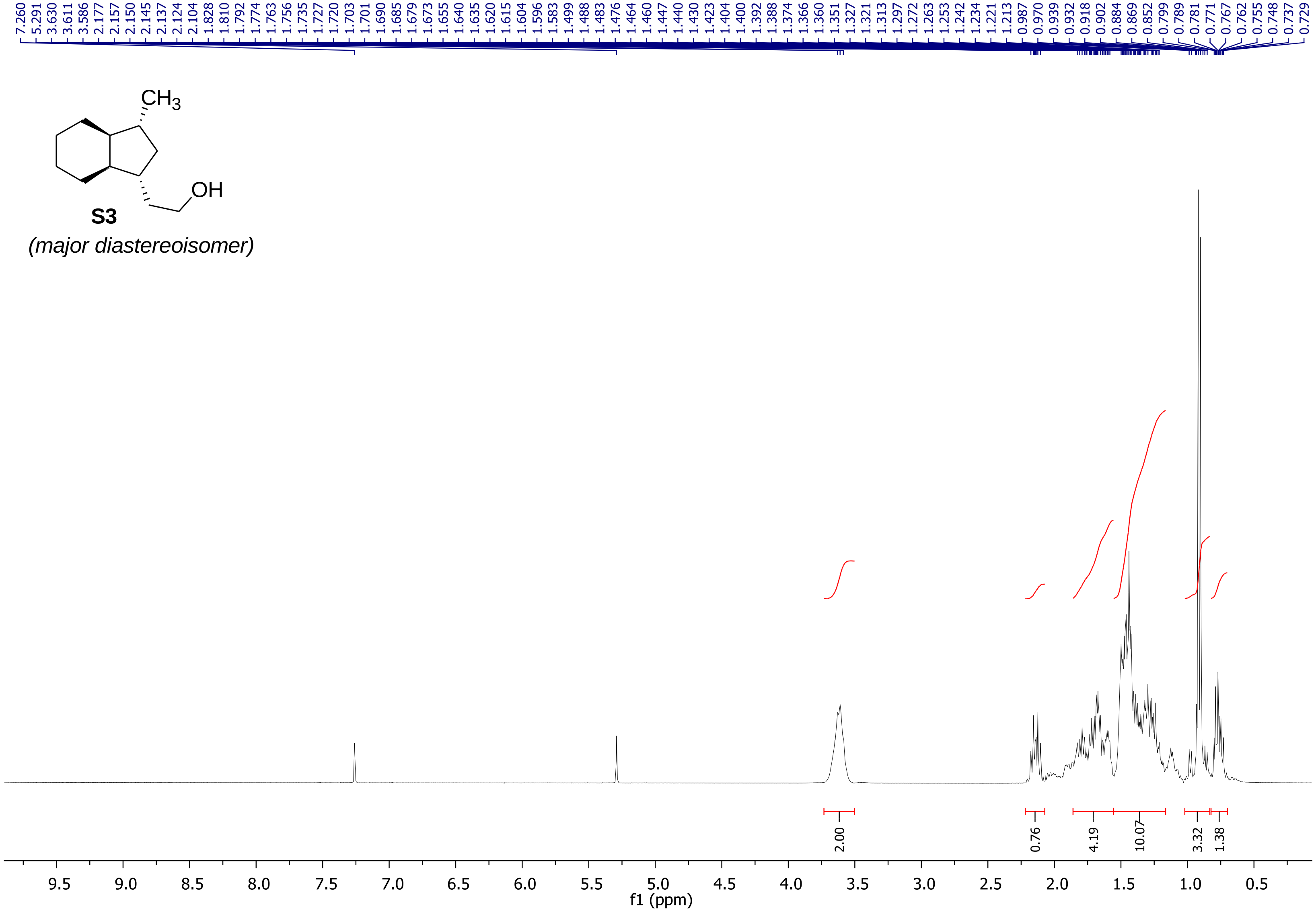

^{1}H NMR ($CDCl_3$, 400 MHz) of compound **S3**

5. Supplementary material for DFT calculation

The most stable conformer (with the lowest Gibbs Energy) for each structure shown in Scheme 7 (unless otherwise indicated) was identified by investigating the conformational space using the Conformer-Rotamer Ensemble Sampling Tool CREST [1], which combines semiempirical tight-binding methods xTB [2] with a meta-dynamic driven search algorithm. The calculation was performed with the Hamiltonian GFN2-xTB [3] with the conformer Energy-Window set to 12 kcal/mol. After removing doubles, only the structures inside an energy window of 6.00 Kcal/mol were considered for further optimization at a higher theory level.

Ultimate full optimization and analytical frequency calculations (T = 298.15 K and 1 atm pressure) were carried out at the DFT level by means of the WB97X [4] functional with the atom-pairwise dispersion correction D4 [5] and the def2-TZVPP basis set [6], using the ORCA 5.0.4 program package [7]. The effective core potential def2-ECP [8], including the relativistic effect, as implemented in Orca 5.0.4, was employed for the Au Atom. Solvent CH_2Cl_2 was simulated with the PCM model [9]. All stationary points were characterized by frequency calculation, to confirm the nature of the stationary points and to obtain ZPE-Energy and thermal corrections. Rotational and vibrational entropy were computed according to Herzberg Infrared and Raman Spectra and QRRHO of S. Grimme [10], respectively. IRC calculation starting on each TS was performed at the same theory level to verify that we indeed found the desired TS structure. To save computer time, PMe_3 was used instead of PPh_3.

References Section 5

[1] Pracht, P.; Bohle, F.; Grimme, S. Automated exploration of the low-energy chemical space with fast quantum chemical methods. *Phys. Chem. Chem. Phys.* **2020**, *22*, 7169–7192.

[2] Bannwarth, C.; Caldeweyher, E.; Ehlert, S.; Hansen, A.; Pracht, P.; Seibert, J.; Spicher, S.; Grimme, S. *WIREs Comput. Mol. Sci.*, **2020**, *11*, e1493.

[3] Bannwaarth, C.; Ehlert, S.; Grimme, S. GFN2-xTB An Accurate and Broadly Parametrized Self-Consistent Tight-Binding Quantum Chemical Method with Multipole Electrostatics and Density-Dependent Dispersion Contributions. *J. Chem. Theory Comput.* **2019**, *15*, 1652–1671.

[4] Chan, B.; Gill, P. M. W.; Kimura, M. Assessment of DFT Methods for Transition Metals with the TMC151 Compilation of Data Sets and Comparison with Accuracies for Main- Group Chemistry. *J. Chem. Theory Comput*. **2019**, *15*, 3610−3622.

[5] Caldeweyher, E.; Ehlert, S.; Hansen, A.; Neugebauer, H.; Spicher, S.; Bannwahrt, C.; Grimme, S. A generally applicable atomic-charge dependent London dispersion correction. *J.Chem.Phys*. **2019**, *150*, 154122.

[6] Weigend, F; Ahlrichs, R. Balanced basis sets of split valence, triple zeta valence and quadrupole zeta valence quality for H to Rn: Design and assessment of accuracy. *Phys. Chem. Chem. Phys*. **2005,** *7,* 3297–3305.

[7] Neese, F. Software update: The ORCA program system—Version 5.0. *WIREs Comput Mol Sci*. **2022**, *12*, e1606.

[8] Andrae, D.; Haeussermann, U.; Dolg, M.; Stoll, H.; Preuss, H. Energy-adjusted ab initio pseudopotentials for the second and third row transition elements. *Theor. Chim. Acta* **1990**, *77*, 123–141.

[9] Tomasi, J.; Mennucci, B.; Cammi, R. Quantum mechanical continuum solvation models. *Chem. Rev.* **2005**, *105,* 2999–3093.

[10] Grimme, S. Supramolecular Binding Thermodynamics by Dispersion-Corrected Density Functional Theory. *Chem. Eur. J.* **2012**, *18*, 9955–9964.

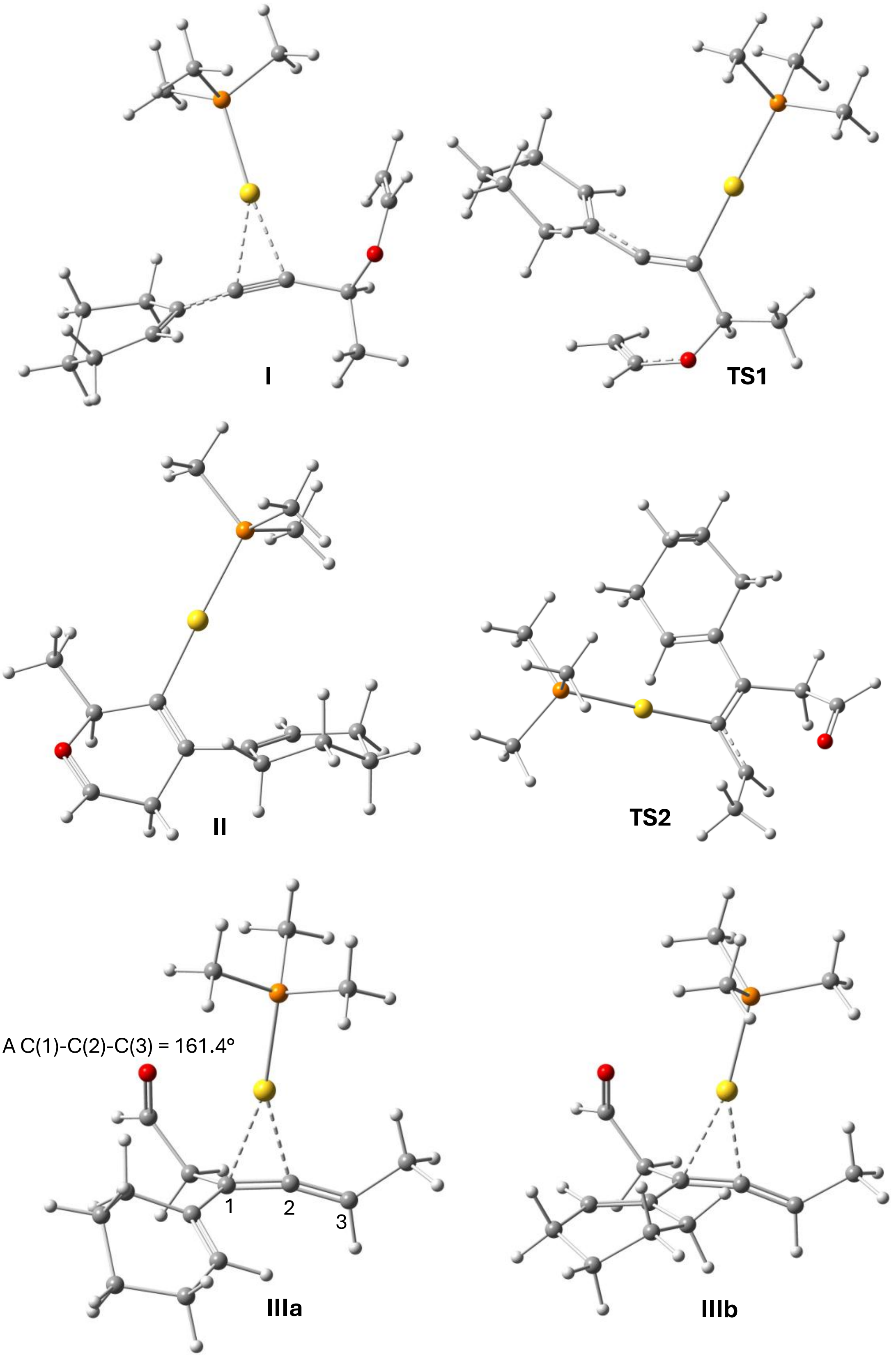

Figure S2. Calculated structures for Scheme 7

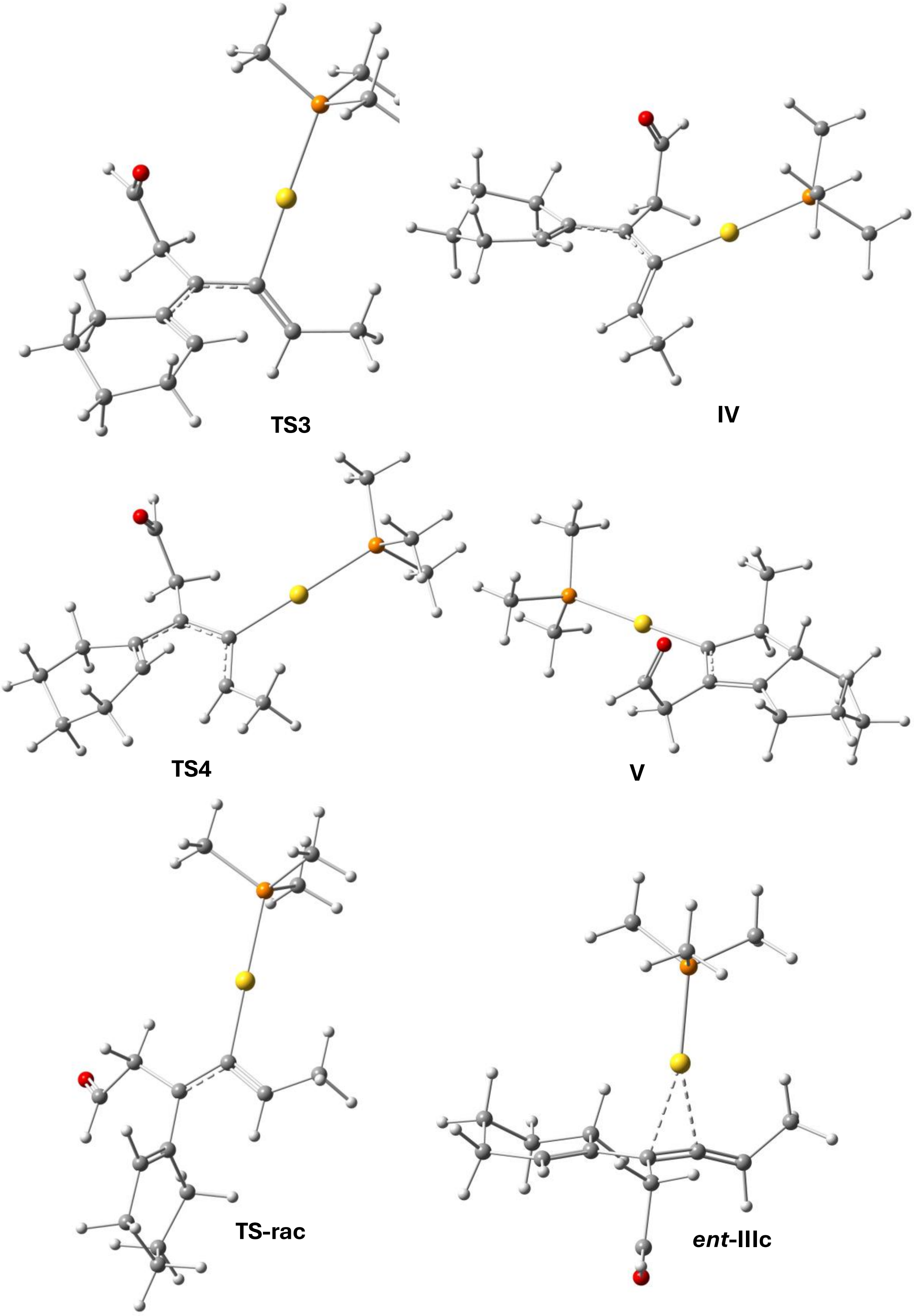

Figure S3. Calculated structures for Scheme 7.

5.1 Cartesian coordinates of the optimized structures.

I

CARTESIAN COORDINATES

```
  C   4.06580708471401      1.98457330413347      1.01430114099395
  C   5.28809365351055      1.32506916515580      0.38640459154705
  C   4.92330662635694      0.66460825558064     -0.93938070219383
  C   3.63772568322791     -0.09533737818260     -0.86781877927014
  C   2.77869672552063      0.01394912903832      0.15098103612428
  C   2.98797296901921      0.95000258884647      1.31871752787380
  H   4.82761987856450      1.41759516678012     -1.72979175853614
  H   6.08267134575985      2.05541194939590      0.23014562239137
  H   5.67960812688743      0.56840690480985      1.07269314815517
  H   4.33810936215654      2.50958691097938      1.93057580982714
  H   3.66443709767594      2.73192077271750      0.32300113662562
  H   3.40192173628988     -0.76528148099941     -1.68787872564615
  H   2.04071839197199      1.44030270342082      1.55691559329718
  H   3.26029728023584      0.36175290809664      2.20028015721397
  H   5.71439755885138     -0.01128781699618     -1.27171647134285
  C   1.60600590080194     -0.81480423571455      0.15151791092657
  C   0.68799384990323     -1.63138073317684      0.16244940814339
  C   0.04113597167681     -2.97174079626863      0.25779515676134
  H  -0.37767041837461     -3.08441942781376      1.26042788456482
  O  -0.98420303709846     -3.12201416936853     -0.70992690624593
  C   1.06965697976808     -4.05810217063835      0.00322316823041
  H   1.88259896647774     -3.97507132055634      0.72297002088843
  H   0.59713627757028     -5.03298762744228      0.11221373847250
  H   1.47492658139226     -3.96517730780937     -1.00372026496603
  C  -2.19556602185757     -2.56845589135871     -0.46585054381204
  H  -2.85110646273872     -2.73298263404282     -1.31238606707196
  C  -2.60137562975885     -1.91027057165607      0.61572850169879
  H  -3.61850814811119     -1.54583901233720      0.63599761882766
  H  -1.99060822755941     -1.74221977790882      1.49217771004309
  Au -0.41741487096980      0.28110728475035     -0.03372979632050
  P  -1.77740460072554      2.13004949592989     -0.25573497601752
  C  -3.48351822361921      1.80756177798597      0.24342375757326
  H  -3.50433944437436      1.49124906688804      1.28565511434521
  H  -4.07855261694620      2.71359807084767      0.12433830804049
  H  -3.89960121587266      1.01293320379766     -0.37490718236613
  C  -1.88270725747845      2.75444173571453     -1.94711093483610
  H  -2.54559916910216      3.61981632367300     -1.97912185184666
  H  -0.89034382540571      3.04206809562893     -2.29149672650037
  H  -2.27114801612928      1.97347410995004     -2.59941462409530
  C  -1.24270499698978      3.53623640900175      0.74461774314825
  H  -0.23582861830845      3.83047458954558      0.45138854963662
  H  -1.92572960432015      4.37336115907724      0.59541588278752
  H  -1.23725203069241      3.25283149182490      1.79628161842975
```

```
   Total DFT Energy (No VdW correction)   -1138.9245650265
        Van der Waals Correction:       -0.0085015430
        Temperature (Kelvin)           :       298.1500000000
        Pressure (atm)                 :         1.0000000000
        Total Mass (AMU)               :       449.3045000000
        Spin Degeneracy                :         1.0000000000
        Electronic Energy (Hartree)    :     -1138.9330665694
        Translational Energy (Hartree) :         0.0014162714
        Rotational Energy (Hartree)    :         0.0014162714
        Vibrational Energy (Hartree)   :         0.0207357684
        Zero Point Energy (Hartree)    :         0.3693584489
```

```
        Inner Energy (Hartree)          :      -1138.5401398092
        Enthalpy (Hartree)              :      -1138.5391956002
        Electronic entropy              :          0.0000000000
        Rotational entropy              :          0.0164156631
        Vibrational entropy             :          0.0389234114
        Translational entropy           :          0.0164156631
        Entropy                         :          0.0763383078
        Gibbs Energy (Hartree)          :      -1138.6155339080
        Magnitude of dipole moment (Debye) :        4.5002205371
-------------------------------------------------------------------------
```

TS1

CARTESIAN COORDINATES

C	-2.40551297175645	-1.29031453254687	0.12074190700414
C	-2.77798101808277	-2.75930844930471	0.20201649752071
O	-3.84192129562485	-2.90095429377681	1.15443802953656
C	-4.88189143655904	-2.09527165155387	1.02844027356221
C	-5.18663080588473	-1.38298512045073	-0.07742621018239
Au	-0.41916074478178	-0.59230203963838	0.05257443297790
H	-5.44653111319051	-2.00593974858106	1.94986431845486
P	1.76329437460985	0.13561400181795	-0.00924617161986
H	-6.03671161241745	-0.71735857764751	-0.03558180248516
H	-4.79045811915403	-1.62445019261869	-1.05395732922918
C	2.33172886040495	0.62260362674200	-1.65621258585584
H	2.24941847787436	-0.22488641597816	-2.33552053342469
H	3.37078233981322	0.95048788772817	-1.60776175197195
H	1.71104323767187	1.43575213499473	-2.03040288669758
C	2.95637649059127	-1.10666396209785	0.54329687135552
H	3.96625742171955	-0.69763484354530	0.49522760248605
H	2.88969998514562	-1.98598398014593	-0.09617172733064
H	2.73072490301869	-1.39843161219308	1.56819840866341
C	2.07985584778368	1.57842439915581	1.03520496962854
H	3.12971848557240	1.86490566840014	0.96261337229781
H	1.83887995944429	1.33881189843526	2.07007627628910
H	1.45332652960991	2.40813775050954	0.71046762239104
C	-3.24444381128077	-0.33324443522645	0.08925490661435
C	-3.66058460261445	1.03852358806695	0.06739366490023
C	-3.51244793473746	1.73890083995354	-1.06426266215526
C	-4.21661024025687	1.63253093151312	1.33994431717979
C	-3.88185983977579	3.18109917508852	-1.19117225577885
H	-3.09136052981341	1.24708167081615	-1.93507401453096
C	-4.21106451427923	3.15732908092525	1.29578992828203
H	-3.63123260121954	1.26893065458364	2.18714817085370
H	-5.23589159998462	1.26062294893753	1.48780261010799
C	-4.75226060335086	3.66453805565552	-0.03564801460156
H	-2.95665233982087	3.76592478214942	-1.24624553231250
H	-4.38165408591982	3.33224727395668	-2.15080999285117
H	-4.79989999287289	3.54962051442758	2.12573062271968
H	-3.18730199643415	3.51943362600804	1.42985215568965
H	-4.80353312963597	4.75383147642320	-0.04108580208286
H	-5.77303797798452	3.29552677590376	-0.17267377018320
C	-1.67259108702109	-3.68340343409991	0.65165712732968
H	-1.29011646521691	-3.37555281671809	1.62491733550273
H	-0.85752855734952	-3.65629202894319	-0.07069520262862
H	-2.04695615009551	-4.70409014624115	0.71921378910422
H	-3.14426086614352	-3.05900227088482	-0.78488188452968

```
   Total DFT Energy (No VdW correction)   -1138.9047419048
        Van der Waals Correction:       -0.0084890926
        Temperature (Kelvin)            :        298.1500000000
```

Pressure (atm)	:	1.0000000000
Total Mass (AMU)	:	449.3045000000
Spin Degeneracy	:	1.0000000000
Electronic Energy (Hartree)	:	-1138.9132309974
Translational Energy (Hartree)	:	0.0014162714
Rotational Energy (Hartree)	:	0.0014162714
Vibrational Energy (Hartree)	:	0.0194832135
Zero Point Energy (Hartree)	:	0.3693806433
Inner Energy (Hartree)	:	-1138.5215345977
Enthalpy (Hartree)	:	-1138.5205903887
Electronic entropy	:	0.0000000000
Rotational entropy	:	0.0164967580
Vibrational entropy	:	0.0353477735
Translational entropy	:	0.0164967580
Entropy	:	0.0728437648
Gibbs Energy (Hartree)	:	-1138.5934341535
Magnitude of dipole moment (Debye)	:	2.3803935664

II

CARTESIAN COORDINATES

C	-2.60517760440492	-1.41456058586365	-0.11856752508604
C	-2.82224003088255	-2.89996311096690	-0.16812327126689
O	-3.97337536825868	-3.23544302723519	-1.11944842745410
C	-4.96949850779765	-2.49354192163225	-1.13086217927121
C	-5.06969218044514	-1.32506406039015	-0.25992328477572
Au	-0.68421494095995	-0.62512357659548	-0.01592760242261
H	-5.74577927466397	-2.77568163954644	-1.84023362936506
P	1.42207466952427	0.32958398880397	0.07234981511761
H	-5.86766123936725	-0.67241044072395	-0.60493138447710
H	-5.37921764689778	-1.71654645224380	0.72273325963982
C	1.89780498884822	0.92426835827477	1.71652998753752
H	1.90395289108654	0.09052159582214	2.41776912589439
H	2.88953854435813	1.37680732593940	1.68148039078452
H	1.17384758839987	1.66336659265154	2.05855736435913
C	2.78471412887733	-0.75766452100300	-0.42007298408632
H	3.73442017615542	-0.22602555474992	-0.34967933667164
H	2.80855405426788	-1.62981055210459	0.23235595306921
H	2.63140132636702	-1.09180141881891	-1.44563410168727
C	1.60208950640450	1.78395563480441	-0.99349854255067
H	2.60761663142413	2.19647919163573	-0.90226449283133
H	1.41806625316367	1.50150086488879	-2.02946794504528
H	0.87243980293429	2.53928705494849	-0.70248705597284
C	-3.70699943195464	-0.65594367861661	-0.12933524449900
C	-3.72845183684007	0.82646610754385	-0.05661132871446
C	-3.13261976790332	1.47088467840372	0.94633124194153
C	-4.43488810700905	1.56582406051923	-1.17246261232015
C	-3.08428833953178	2.96549360519912	1.08284851981103
H	-2.63864761437138	0.88947035519715	1.71910187677619
C	-4.01206684804606	3.02938627953477	-1.24311463201285
H	-4.22667754397666	1.06435607937159	-2.12214285551859
H	-5.51943581382343	1.50671675279515	-1.02981172288846
C	-4.05607964759765	3.66713703498442	0.13968977827491
H	-2.05958522307054	3.30108506919139	0.88304992146279
H	-3.28848666572793	3.24194034806535	2.12000638826885
H	-4.65691256589036	3.56824754401053	-1.93929233008519
H	-2.99234169774877	3.09250667269379	-1.63646968636958
H	-3.82197499941714	4.73141673713229	0.08299795366799
H	-5.07230850967524	3.58471314519156	0.53769386164580
C	-1.68647168301489	-3.75426655012754	-0.66190018359542

H	-1.33604916771089	-3.40369274064267	-1.63282997726025
H	-0.86416216116254	-3.67712193556275	0.04838216166969
H	-1.98761338483237	-4.79853201132714	-0.73379441432266
H	-3.21084838882855	-3.27095884945223	0.78469879062972

```
Total DFT Energy (No VdW correction)   -1138.9420848265
     Van der Waals Correction:       -0.0087244799
     Temperature (Kelvin)          :       298.1500000000
     Pressure (atm)                :         1.0000000000
     Total Mass (AMU)              :       449.3045000000
     Spin Degeneracy               :         1.0000000000
     Electronic Energy (Hartree)   :     -1138.9508093064
     Translational Energy (Hartree) :        0.0014162714
     Rotational Energy (Hartree)   :         0.0014162714
     Vibrational Energy (Hartree)  :         0.0192678427
     Zero Point Energy (Hartree)   :         0.3718380670
     Inner Energy (Hartree)        :     -1138.5568708538
     Enthalpy (Hartree)            :     -1138.5559266448
     Electronic entropy            :         0.0000000000
     Rotational entropy            :         0.0164015813
     Vibrational entropy           :         0.0350181700
     Translational entropy         :         0.0164015813
     Entropy                       :         0.0724189846
     Gibbs Energy (Hartree)        :     -1138.6283456294
     Magnitude of dipole moment (Debye) :        6.1497601543
```

TS2

CARTESIAN COORDINATES

C	4.36158292937668	0.60977809897997	0.22986133092513
C	4.60474057048036	0.79859561014527	-1.26218474133567
C	3.28635253439305	0.77253350697370	-2.02924445558470
C	2.35301613351071	-0.29137282328995	-1.53471804289819
C	2.53432345504541	-0.98621475029524	-0.41045571612145
C	3.71928426405049	-0.74677743710307	0.49842193274743
H	2.77617139690816	1.73930765142675	-1.93858521492726
H	5.12988160985157	1.73563293603103	-1.45268329710678
H	5.25127320976238	-0.00787260596811	-1.62100502937668
H	5.29568892683911	0.69178884656289	0.78739344531891
H	3.69965705120155	1.40265064000583	0.59265925565501
H	1.47588102501235	-0.49729143235540	-2.14121138941007
H	3.39290451758796	-0.81294445455466	1.54040218187386
H	4.46485543064028	-1.53633232439055	0.36026888315552
H	3.46283241085754	0.63256496487235	-3.09811756597972
C	1.55713352744411	-2.02988587216362	-0.03222065713380
C	0.21382872663722	-1.80248540683406	0.05081137330738
C	-0.63536795315693	-2.90218912017301	0.26356167963837
H	-0.42651878437037	-3.82531298249339	-0.27564096786600
O	0.37588702877933	-3.87954659915109	1.92182846287949
C	-1.98969656538793	-2.81198753133912	0.84123424580267
H	-2.29634866737513	-3.76115019717749	1.27587366631059
H	-2.07022427594905	-2.00119436560317	1.56346219336502
H	-2.67467205707276	-2.59683684247156	0.01381115539901
C	1.55934852461871	-3.88529473145283	1.67604524816988
H	2.26628726920679	-4.30518237505572	2.40223672433388
C	2.12485554190253	-3.35788530234628	0.37872785832067
H	3.21009917188290	-3.36315791829101	0.41818818343082
H	1.82215956890052	-4.10768691715970	-0.36618396603933
Au	-0.57682090317521	0.14059744896602	0.02160656852444
P	-1.40856772954973	2.29482924924390	0.01233877041496

C	-2.90263892124202	2.51320333463054	-0.98682042883159
H	-3.68618744341640	1.85196542612905	-0.61882921288711
H	-3.24380502870434	3.54767351571587	-0.93262488036710
H	-2.68611403302109	2.25649076841203	-2.02320496134753
C	-0.24516108437615	3.52502916283308	-0.63111389777673
H	-0.69702260404542	4.51736803789634	-0.60606923842638
H	0.65923983502861	3.52226775968688	-0.02313018247998
H	0.02137601695605	3.27359302812818	-1.65727306392891
C	-1.86095400358479	2.91671924110985	1.65142989689879
H	-2.62350178100833	2.27266060001631	2.08771932462108
H	-0.98313080004211	2.90774671786741	2.29670145294055
H	-2.24543322139696	3.93435489403573	1.57388869579155

Total DFT Energy (No VdW correction)   -1138.9281490486
Van der Waals Correction: -0.0088273955

Temperature (Kelvin)	:	298.1500000000
Pressure (atm)	:	1.0000000000
Total Mass (AMU)	:	449.3045000000
Spin Degeneracy	:	1.0000000000
Electronic Energy (Hartree)	:	-1138.9369764441
Translational Energy (Hartree)	:	0.0014162714
Rotational Energy (Hartree)	:	0.0014162714
Vibrational Energy (Hartree)	:	0.0193934844
Zero Point Energy (Hartree)	:	0.3690256289
Inner Energy (Hartree)	:	-1138.5457247880
Enthalpy (Hartree)	:	-1138.5447805789
Electronic entropy	:	0.0000000000
Rotational entropy	:	0.0163993831
Vibrational entropy	:	0.0350218558
Translational entropy	:	0.0163993831
Entropy	:	0.0724204723
Gibbs Energy (Hartree)	:	-1138.6172010512
Magnitude of dipole moment (Debye)	:	2.1888015314

IIIa

CARTESIAN COORDINATES

C	3.24394337548933	-1.09871544123349	2.17800332020639
C	4.55092403589485	-1.36109001894898	1.44310608975790
C	4.27552821713058	-1.69204963397128	-0.01864046594827
C	3.24790862982502	-0.78923778012218	-0.63159126293437
C	2.45553771225923	0.02932672288240	0.06448894334704
C	2.51441138668966	0.09492198985210	1.57293931446087
H	5.19238938661140	-1.63769716795765	-0.61008326878281
H	5.18083471983994	-0.46827416089606	1.50183565903907
H	5.10514167863714	-2.17469682298353	1.91302617882313
H	2.60688721626478	-1.98612671728455	2.10823072041897
H	3.42406970489762	-0.91789866915398	3.23863321808687
H	3.17784230394307	-0.82683861421571	-1.71263481527236
H	3.00837904365928	1.02704797744168	1.86623570456433
H	1.50140669645812	0.15612509625787	1.97952959121181
H	3.92410112218600	-2.72574673994545	-0.11368884084665
C	1.52599178178607	0.96167593950976	-0.63092110127569
C	0.83134396241625	1.89188352874751	0.06716445224040
C	0.51317658871515	3.00972267730768	0.66316096480739
H	1.29948759300216	3.76207366644394	0.68752535354405
O	0.44263877249391	-0.97905103977959	-2.56891663620112
C	-0.78353258358346	3.37021782569894	1.31518818290374
H	-1.19907193383456	4.26461599134453	0.84880668067152
H	-1.51261362761541	2.56431639845474	1.24285120806158

H	-0.61839844269974	3.60200518903296	2.36843471974577
C	1.06125356609038	-0.02922415681636	-2.98102428567628
H	1.26533161806391	0.08020943642685	-4.05955978256670
C	1.58018874722226	1.10339856344951	-2.14118133687524
H	2.61286514713026	1.29617985690666	-2.44570297514319
H	1.01275955491217	1.98448734056875	-2.45540938476140
Au	-0.60595929718694	0.16563856813199	-0.05640385441909
P	-2.52721108371380	-1.06916402960375	0.30736156596822
C	-3.80875611334131	-0.14374472217421	1.18181629263274
H	-4.08177696602655	0.73735793039787	0.60249494127873
H	-4.68663807584585	-0.77573345351724	1.32103759075988
H	-3.42999956774258	0.17358616209015	2.15254005918763
C	-2.24790865166824	-2.55598843367726	1.29451479723445
H	-1.84900544809499	-2.27607186264046	2.26858620848008
H	-3.18915103920694	-3.09126097088175	1.42512586400740
H	-1.52960709011373	-3.19958921081611	0.78849515574744
C	-3.29927823068028	-1.64069378291201	-1.22234660565960
H	-4.19178334576020	-2.22223654682035	-0.98854411611898
H	-3.57332930783101	-0.78236832648715	-1.83425645103496
H	-2.59444722937298	-2.25966927720679	-1.77602980177072

```
 Total DFT Energy (No VdW correction)   -1138.9534948702
      Van der Waals Correction:       -0.0089707230
      Temperature (Kelvin)           :        298.1500000000
      Pressure (atm)                 :          1.0000000000
      Total Mass (AMU)               :        449.3045000000
      Spin Degeneracy                :          1.0000000000
      Electronic Energy (Hartree)    :      -1138.9624655932
      Translational Energy (Hartree) :          0.0014162714
      Rotational Energy (Hartree)    :          0.0014162714
      Vibrational Energy (Hartree)   :          0.0208799873
      Zero Point Energy (Hartree)    :          0.3685117672
      Inner Energy (Hartree)         :      -1138.5702412958
      Enthalpy (Hartree)             :      -1138.5692970868
      Electronic entropy             :          0.0000000000
      Rotational entropy             :          0.0162507478
      Vibrational entropy            :          0.0384559060
      Translational entropy          :          0.0162507478
      Entropy                        :          0.0757058871
      Gibbs Energy (Hartree)         :      -1138.6450029739
      Magnitude of dipole moment (Debye) :        3.4371213533
-------------------------------------------------------------------------
```

IIIb

CARTESIAN COORDINATES

C	4.57480139135065	0.61848433470338	-0.09781506659883
C	5.23081434292687	-0.09223213855528	-1.27359183178896
C	4.21122876747070	-0.33080901025843	-2.38140298727585
C	2.90588627820909	-0.84386362103202	-1.85298329563099
C	2.55766012574282	-0.81641352445194	-0.56631536309501
C	3.45377764506952	-0.22741949425548	0.49823133051125
H	4.02323744117881	0.59923525439863	-2.92985987031906
H	6.07173055938363	0.48768207638333	-1.65640613860731
H	5.63126565269760	-1.05271864709991	-0.93512554630559
H	5.30863215043410	0.84993964118940	0.67545008292814
H	4.16153785543711	1.57203791093711	-0.44131334472641
H	2.21645390192577	-1.26846138887808	-2.57650073851300
H	2.85600393229991	0.37849128006075	1.18185950560114
H	3.87972592471355	-1.03621908630471	1.10339865158187
H	4.59808231315513	-1.03643522781748	-3.12006136890919

C	1.26912349464755	-1.44554525603434	-0.13134520788575
C	0.20642263146446	-1.53634915320660	-0.96185756505397
C	-0.61068590971571	-1.97308556390441	-1.88302544279438
H	-0.27404149043235	-2.86293129404084	-2.41281916532279
O	1.01602764820321	-0.51125687576299	2.69175045836629
C	-1.93009142086751	-1.40115801769756	-2.29239176232847
H	-2.71317878473890	-2.15187503707167	-2.17712278678106
H	-2.19264322857492	-0.52460614449241	-1.70198210610920
H	-1.90397744495964	-1.12060299274420	-3.34639721699263
C	1.27046281729332	-1.66900833926858	2.46916508977519
H	1.47406020514617	-2.36524670650175	3.30013114417781
C	1.30001156495416	-2.31675406077329	1.11410057498701
H	2.19666600393296	-2.94346876341549	1.07343078030226
H	0.45399700364889	-3.01121614437626	1.10886438257302
Au	-0.17674042366284	0.35452990709648	0.17052328962836
P	-1.21643078160575	2.34056033874815	0.75551474413376
C	-2.49159286449813	2.12581576439219	2.01656265209297
H	-2.04790749189005	1.68961157807192	2.91065608082715
H	-2.93266390586925	3.09262744982748	2.26178784247955
H	-3.26539584595587	1.45668852500488	1.64261667664186
C	-2.03644395885784	3.17210281679529	-0.62233045104233
H	-2.50305569858491	4.09241336915832	-0.26902578134126
H	-1.30493218069213	3.40647449649631	-1.39433338062976
H	-2.79755409029267	2.51590189422652	-1.04284515804216
C	-0.06329252898195	3.55258052056859	1.43789717339511
H	0.42278742655423	3.13465870165626	2.31864685605626
H	0.69519524968814	3.79285963497607	0.69414411377310
H	-0.60408498734794	4.45845544325267	1.71441632626186

Total DFT Energy (No VdW correction) -1138.9498540974
Van der Waals Correction: -0.0089376934
Temperature (Kelvin) : 298.1500000000
Pressure (atm) : 1.0000000000
Total Mass (AMU) : 449.3045000000
Spin Degeneracy : 1.0000000000
Electronic Energy (Hartree) : -1138.9587917907
Translational Energy (Hartree) : 0.0014162714
Rotational Energy (Hartree) : 0.0014162714
Vibrational Energy (Hartree) : 0.0200223268
Zero Point Energy (Hartree) : 0.3683230518
Inner Energy (Hartree) : -1138.5676138692
Enthalpy (Hartree) : -1138.5666696602
Electronic entropy : 0.0000000000
Rotational entropy : 0.0162576702
Vibrational entropy : 0.0364387283
Translational entropy : 0.0162576702
Entropy : 0.0736956318
Gibbs Energy (Hartree) : -1138.6403652920
Magnitude of dipole moment (Debye) : 3.0545542383

TS3

CARTESIAN COORDINATES

C	5.23410210755088	-0.77189319968932	-0.52728351477851
C	5.19577529815999	-1.08085487313754	-2.01731865334651
C	3.95470894696391	-0.45877594554509	-2.64177461628289
C	2.73075800528778	-0.63950604497567	-1.81980402856653
C	2.72626247952393	-1.09942483949855	-0.54680862832519
C	4.02549888953334	-1.37036100010571	0.18489694805570
H	4.08925144532819	0.62011218151917	-2.78840278288932

H	6.09000948899043	-0.70728344652311	-2.51649289537688
H	5.17497971862154	-2.16463318488665	-2.16322392585222
H	6.14790420548643	-1.15690248776393	-0.07397167455037
H	5.23763262464120	0.31304946097043	-0.38501194501012
H	1.79237394032639	-0.34494060752190	-2.27410954891317
H	3.97119186259169	-0.96727014396776	1.19669929994493
H	4.15510167141482	-2.45220974401449	0.28992846751925
H	3.75728541919462	-0.85722907055025	-3.64060423515692
C	1.46332489967041	-1.35935801123966	0.09991745095548
C	0.24295287425079	-1.27435303564152	-0.57035580645092
C	-0.19474935532974	-1.94372194893576	-1.63721833100001
H	0.38382374806593	-2.81235551901648	-1.95359707285381
O	1.95514951092904	0.50265363291271	2.24895998769246
C	-1.44017835935803	-1.67729884800629	-2.41321325228360
H	-2.15597108740889	-2.49228667042330	-2.28435147812301
H	-1.91706623248342	-0.74625637635748	-2.10723452069745
H	-1.20917001545792	-1.62889275992974	-3.47943845082037
C	1.76681094625620	-0.65118457581037	2.53491060651298
H	1.79197232573005	-0.98863540175935	3.58420062696274
C	1.45878810254950	-1.75137446188314	1.54735045944946
H	2.19716825830310	-2.54262302905042	1.71591489564782
H	0.48803776713676	-2.16260968570059	1.83599741123964
Au	-0.83611191106733	0.30116704786058	0.35754400388122
P	-2.02713635298273	2.03671500462826	1.31249191906380
C	-3.61590995671139	1.54535753274902	2.02362953895102
H	-3.45356445204066	0.79206745137000	2.79347019787535
H	-4.11060126625341	2.41289890582115	2.46188753219960
H	-4.24802283238427	1.12197354520176	1.24406434835460
C	-2.42998120630840	3.37215945100645	0.16093055546419
H	-2.98146614791374	4.15582022420195	0.68175494310082
H	-1.51130197046196	3.78690464175916	-0.25189409615986
H	-3.03754177080128	2.98049405282239	-0.65392790376810
C	-1.13852234367405	2.84334790809902	2.66636726758559
H	-0.92629041047100	2.11483297261602	3.44802653552255
H	-0.19588943631961	3.24288323752552	2.29404422455971
H	-1.74210442907913	3.65365566087050	3.07700014066687

Total DFT Energy (No VdW correction)   -1138.9425452885
Van der Waals Correction: -0.0086947919
Temperature (Kelvin) : 298.1500000000
Pressure (atm) : 1.0000000000
Total Mass (AMU) : 449.3045000000
Spin Degeneracy : 1.0000000000
Electronic Energy (Hartree) : -1138.9512400804
Translational Energy (Hartree) : 0.0014162714
Rotational Energy (Hartree) : 0.0014162714
Vibrational Energy (Hartree) : 0.0198743686
Zero Point Energy (Hartree) : 0.3682612431
Inner Energy (Hartree) : -1138.5602719259
Enthalpy (Hartree) : -1138.5593277168
Electronic entropy : 0.0000000000
Rotational entropy : 0.0163984219
Vibrational entropy : 0.0361195202
Translational entropy : 0.0163984219
Entropy : 0.0735171754
Gibbs Energy (Hartree) : -1138.6328448923
Magnitude of dipole moment (Debye) : 1.2130424763

IV

CARTESIAN COORDINATES

C	5.38008702199715	-1.45423940693172	-0.71283050573580
C	5.14189762519223	-1.79081558259130	-2.17818658888430
C	4.08698742494422	-0.85503355318726	-2.74972658138390
C	2.92184031596447	-0.66683584689733	-1.84897033494170
C	2.87210784929865	-1.10344932216660	-0.56182800800596
C	4.11200156059190	-1.66398968979698	0.10811285445701
H	4.50907360285941	0.13729178697534	-2.94998970083892
H	6.06397396667730	-1.70706406492475	-2.75342952528639
H	4.80068130413829	-2.82647251060114	-2.26440465407221
H	6.18317021982457	-2.06370730892848	-0.29790991492116
H	5.69578585704926	-0.40970279575410	-0.63266331206975
H	2.09922144990229	-0.08100872295127	-2.23868427920680
H	4.23618096881838	-1.19599168651098	1.08595796698263
H	3.95925973905053	-2.73150401071508	0.29504418157460
H	3.71172763703149	-1.20265948883963	-3.71656435186129
C	1.63975574131901	-1.01856589773840	0.16190103357016
C	0.41350967533755	-0.81038554898814	-0.48805932055087
C	0.13843311898218	-1.42847883802374	-1.66288832191280
H	0.76794052981032	-2.27023115632931	-1.95320204951497
O	2.54750210037817	1.07208619063457	1.87651499164984
C	-1.01517225648579	-1.15941366553588	-2.55813627838119
H	-1.72103237526057	-1.99335231942573	-2.52264281405242
H	-1.54005529722407	-0.24374994581182	-2.28878279353556
H	-0.66708876195296	-1.09526772805525	-3.59153887312932
C	2.17617176292711	0.05155596885175	2.39540727040051
H	2.18065819831292	-0.06323396211318	3.49223854067661
C	1.65933207312659	-1.15678674314237	1.65241148764500
H	2.28433491238380	-2.00352215472353	1.95261228392047
H	0.65718715618020	-1.35671107136822	2.03988253597010
Au	-0.94773770494933	0.49282889030977	0.44070247784228
P	-2.40227113342176	1.97741709491897	1.45883930732406
C	-3.91475010918253	1.22618106507104	2.11066196661626
H	-3.65664843136525	0.46800274234489	2.84895222861985
H	-4.53836911335423	1.98994918166114	2.57692070710599
H	-4.46612353065245	0.75321677916756	1.29894747159788
C	-2.98362447479354	3.30420977867591	0.37370831260842
H	-3.65121839665775	3.96859761108300	0.92365464092480
H	-2.13079320676081	3.87184470041665	0.00352427679091
H	-3.51592407292412	2.87567955219680	-0.47460247796161
C	-1.67153649378914	2.83546966426490	2.87573305995325
H	-1.36877968686970	2.10662304719523	3.62671710611439
H	-0.79196947074427	3.39001773398655	2.55051769471193
H	-2.39643955963961	3.52505899952278	3.31002664172124

Total DFT Energy (No VdW correction)   -1138.9435748526
Van der Waals Correction: -0.0086026739
Temperature (Kelvin) : 298.1500000000
Pressure (atm) : 1.0000000000
Total Mass (AMU) : 449.3045000000
Spin Degeneracy : 1.0000000000
Electronic Energy (Hartree) : -1138.9521775265
Translational Energy (Hartree) : 0.0014162714
Rotational Energy (Hartree) : 0.0014162714
Vibrational Energy (Hartree) : 0.0205966120
Zero Point Energy (Hartree) : 0.3685832376
Inner Energy (Hartree) : -1138.5601651341
Enthalpy (Hartree) : -1138.5592209251
Electronic entropy : 0.0000000000
Rotational entropy : 0.0164348882
Vibrational entropy : 0.0380093879
Translational entropy : 0.0164348882

```
       Entropy                          :         0.0754435094
       Gibbs Energy (Hartree)           :     -1138.6346644344
       Magnitude of dipole moment (Debye) :        2.4297368370
-------------------------------------------------------------------------
```

TS4

CARTESIAN COORDINATES

```
  C   5.23478576847202     -1.94550696208802     -1.02058769926205
  C   4.55492032834272     -2.36074310618438     -2.32064070081060
  C   3.68746164249688     -1.21554867741152     -2.82805334283881
  C   2.84004430046154     -0.62566233187298     -1.74770554246184
  C   2.97926847262123     -0.97177758916451     -0.41370253320372
  C   4.19936816801932     -1.70787466180802      0.07541245034608
  H   4.31222552961664     -0.41731934081843     -3.24174743665600
  H   5.29154288776773     -2.62913924085313     -3.07791881588417
  H   3.93704083980955     -3.24658279851043     -2.14337818340279
  H   5.94279061316180     -2.70503840061810     -0.68898233439081
  H   5.80568362937113     -1.02872538101780     -1.19388075765546
  H   2.33817651619791      0.30271236601101     -1.99011968396331
  H   4.63773161774374     -1.13928497111204      0.89983084456262
  H   3.87723553367014     -2.66226609959150      0.50371514379421
  H   3.03616511344894     -1.53308647602540     -3.64746470910462
  C   1.88196865680992     -0.69359572401641      0.43429214634569
  C   0.68183770336072     -0.47315213018008     -0.22704582970957
  C   0.63839877778460     -0.97986491869927     -1.53092982756195
  H   1.00587151666813     -1.99221307652017     -1.67771105802890
  O   3.31704205951772      1.38380082741973      1.75576806586195
  C   -0.22624885401679     -0.46228536639129     -2.62622797046290
  H   -1.16031184920470     -1.03036411186019     -2.64028181024928
  H   -0.46054222819842      0.59345082374282     -2.50045195367115
  H   0.25375470933412     -0.62316220906811     -3.59253219637890
  C   2.80102785628701      0.54007819612535      2.44393051053416
  H   2.86673166240002      0.59385685328718      3.54422378922101
  C   2.03066657264107     -0.64475795085909      1.92226393008094
  H   2.54810737666902     -1.53779437278294      2.29128584498801
  H   1.05744811462143     -0.64025585821863      2.41945746794644
  Au  -0.90095677929762      0.60538251049433      0.57969909796293
  P   -2.65327088546951      1.82479392084959      1.48884143496077
  C   -4.08707076429337      0.82784110944450      1.96621689851468
  H   -3.78748077414196      0.09007452545680      2.70955618839607
  H   -4.86462489916075      1.46872623991519      2.38360047605175
  H   -4.47633069488250      0.30727949446252      1.09202776194182
  C   -3.32645558179684      3.08351387953283      0.37520276047879
  H   -4.13980128239027      3.61904562724231      0.86637022136966
  H   -2.54063426322143      3.78730644559569      0.10350487533777
  H   -3.70023499800100      2.60749096958043     -0.53054574308443
  C   -2.21419195494616      2.73680311956685      2.98978503106741
  H   -1.87951636434205      2.03865331585811      3.75615829007934
  H   -1.40433579436551      3.43144705419218      2.77006391413774
  H   -3.07891186956616      3.29109749689504      3.35649342480146

    Total DFT Energy (No VdW correction)   -1138.9384759944
       Van der Waals Correction:       -0.0086451933
       Temperature (Kelvin)             :       298.1500000000
       Pressure (atm)                   :         1.0000000000
       Total Mass (AMU)                 :       449.3045000000
       Spin Degeneracy                  :         1.0000000000
       Electronic Energy (Hartree)      :     -1138.9471211877
       Translational Energy (Hartree) :         0.0014162714
       Rotational Energy (Hartree)      :         0.0014162714
```

```
        Vibrational Energy (Hartree)   :          0.0197099093
        Zero Point Energy (Hartree)    :          0.3682361248
        Inner Energy (Hartree)         :      -1138.5563426107
        Enthalpy (Hartree)             :      -1138.5553984016
        Electronic entropy             :          0.0000000000
        Rotational entropy             :          0.0164483011
        Vibrational entropy            :          0.0362398364
        Translational entropy          :          0.0164483011
        Entropy                        :          0.0736873708
        Gibbs Energy (Hartree)         :      -1138.6290857724
        Magnitude of dipole moment (Debye) :        3.4290323299
-------------------------------------------------------------------------
```

V

CARTESIAN COORDINATES

C	5.09208582700764	-1.92615564635943	-1.28485183898517
C	4.19049125995211	-2.84216504379857	-2.10966230452784
C	2.89728090379371	-2.14432766312318	-2.57929520299916
C	2.55878314776383	-0.88530074967819	-1.75798639390863
C	2.99687142433047	-0.97350339431625	-0.35217434073049
C	4.35436435959439	-1.46281590383520	-0.03193916004423
H	2.97795743812333	-1.85354568008187	-3.62664574230736
H	4.73451178146173	-3.22890973545577	-2.97099121743225
H	3.93102960327736	-3.70583587864921	-1.49144024725793
H	6.00473971227248	-2.44813948491735	-0.99868661601178
H	5.39431839448145	-1.05850118256812	-1.87726646108167
H	3.13337065171538	-0.04729140023824	-2.17721377828652
H	4.89058811149452	-0.66842788242874	0.49824600793442
H	4.25488511981582	-2.28008830820450	0.69266683676197
H	2.06159816327238	-2.84199370066360	-2.51072448138860
C	2.01474347447639	-0.55168985382782	0.52392843673049
C	0.88067005788912	-0.16714232125335	-0.19554114571858
C	1.09236479476336	-0.45649057020556	-1.64795721313819
H	0.45653539273532	-1.32718896081424	-1.85694989860968
O	3.44477459133498	1.56583880628587	1.73777188287035
C	0.67439251846951	0.67403730163286	-2.58380185390082
H	-0.38830957865469	0.89293340263458	-2.47872594675368
H	1.23580259128286	1.58387754891038	-2.36426505272987
H	0.86844391666446	0.39479722714695	-3.61970946349106
C	2.95040828356937	0.74578239965386	2.47014839818740
H	3.04222150151333	0.84328070720151	3.56552386687167
C	2.18418919486841	-0.46447031765273	2.00724415766211
H	2.71031099654845	-1.33788075574953	2.40824471164412
H	1.21591806572887	-0.44534555241909	2.51488822996573
Au	-0.82009264880886	0.67288900658570	0.57365186024695
P	-2.75377169837804	1.64490438122606	1.44841888362115
C	-4.09421096959591	0.46827398475805	1.75725660364538
H	-3.76112851194889	-0.28576456734661	2.46933521570982
H	-4.96189060520956	0.99134892561418	2.16084292021162
H	-4.36866412998222	-0.02500056762821	0.82560575054116
C	-3.48814116810832	2.90560559091612	0.37719657145514
H	-4.38522629783835	3.31508541369244	0.84286349990859
H	-2.76829656254431	3.70607902771130	0.21066829458355
H	-3.74772137781508	2.46141740613110	-0.58303267737227
C	-2.50367334281290	2.48081591894292	3.03434217080734
H	-2.13818160423815	1.76539625711052	3.77005329306720
H	-1.76420980873476	3.27202356666221	2.91617268008775
H	-3.44375684353115	2.91094126839873	3.38158320416193

Total DFT Energy (No VdW correction)   -1138.9786779792

```
       Van der Waals Correction:       -0.0085337946
       Temperature (Kelvin)           :        298.1500000000
       Pressure (atm)                 :          1.0000000000
       Total Mass (AMU)               :        449.3045000000
       Spin Degeneracy                :          1.0000000000
       Electronic Energy (Hartree)    :      -1138.9872117738
       Translational Energy (Hartree) :          0.0014162714
       Rotational Energy (Hartree)    :          0.0014162714
       Vibrational Energy (Hartree)   :          0.0198685015
       Zero Point Energy (Hartree)    :          0.3706642424
       Inner Energy (Hartree)         :      -1138.5938464870
       Enthalpy (Hartree)             :      -1138.5929022780
       Electronic entropy             :          0.0000000000
       Rotational entropy             :          0.0164373285
       Vibrational entropy            :          0.0368626491
       Translational entropy          :          0.0164373285
       Entropy                        :          0.0742992110
       Gibbs Energy (Hartree)         :      -1138.6672014889
       Magnitude of dipole moment (Debye) :        3.4693968381
-------------------------------------------------------------------------
```

TS-rac

CARTESIAN COORDINATES

```
  C   4.89709094562806     -2.20165895480060     -1.09836502263621
  C   5.10814290538356     -1.30524837848348     -2.31275081919283
  C   4.74716639556819      0.14123535265141     -1.98950519871090
  C   3.47374088457096      0.25186019438756     -1.20328590358307
  C   2.87929341095660     -0.78623681180617     -0.62553532763503
  C   3.43303782671246     -2.19621595717116     -0.67616656665732
  H   5.55025554134680      0.61421435704243     -1.41436098913066
  H   6.14130463517359     -1.36269274646123     -2.65719397118995
  H   4.47772288001849     -1.66254940681980     -3.13272590820098
  H   5.20928511016205     -3.22450219929363     -1.31252698795962
  H   5.51441902785718     -1.84351064311757     -0.26873251193465
  H   3.03302351549595      1.23984687880891     -1.10537233675482
  H   3.30855296294858     -2.68674952435061      0.29412668542420
  H   2.82674034864913     -2.77912751529668     -1.37827932081179
  H   4.65199927768445      0.72841839667957     -2.90542693459731
  C   1.64109009328151     -0.63289980073278      0.17371775699921
  C   0.34255040346900     -0.78988258410300     -0.32876392197383
  C   0.29863842487180     -1.03917671456970     -1.68797933052641
  H   1.22902586017042     -1.10312500928093     -2.24946296692944
  O   3.05236979715466     -1.25012627768571      3.40748286440349
  C   -0.91350247691086     -1.22744649745900     -2.49281945820086
  H   -0.86372745746715     -2.21184359344579     -2.97017060698861
  H   -1.83144592715408     -1.13467342584432     -1.91748724117536
  H   -0.90482669344678     -0.50902636348311     -3.31854036802096
  C   3.08134584178650     -0.81947492204598      2.28569474734742
  H   4.02598281551947     -0.76669236402633      1.71955233456022
  C   1.84229893054496     -0.28094349328260      1.60573974396232
  H   0.96190267169773     -0.48371553057866      2.21354727459221
  H   1.98496913810260      0.81355090967191      1.58516275171435
  Au  -1.38267965183974     -0.65478273523696      0.84064204409246
  P   -3.30855179987719     -0.51977094121705      2.12547073503602
  C   -3.79555445418910     -2.08546439200658      2.89430644842395
  H   -2.99487457202401     -2.43712658329505      3.54393575584272
  H   -4.70466625333719     -1.94710698216859      3.48072758554743
  H   -3.97289668376291     -2.83192744744395      2.12094425414670
  C   -4.77590051312076      0.00417029019351      1.20208269242918
  H   -5.64194113619698      0.03990879131291      1.86395938579187
```

H	-4.60478623275637	0.99163904690661	0.77486849149171
H	-4.96700068169515	-0.69960396320222	0.39277067935543
C	-3.20241485013845	0.64505783569281	3.50775851653515
H	-2.39193465876107	0.34804351100773	4.17242857685380
H	-2.99578616175652	1.64491813062688	3.12785514807593
H	-4.14158152032048	0.65402773372718	4.06213211018476

```
 Total DFT Energy (No VdW correction)   -1138.9241396925
      Van der Waals Correction:       -0.0083762600
      Temperature (Kelvin)           :        298.1500000000
      Pressure (atm)                 :          1.0000000000
      Total Mass (AMU)               :        449.3045000000
      Spin Degeneracy                :          1.0000000000
      Electronic Energy (Hartree)    :      -1138.9325159525
      Translational Energy (Hartree) :          0.0014162714
      Rotational Energy (Hartree)    :          0.0014162714
      Vibrational Energy (Hartree)   :          0.0201333162
      Zero Point Energy (Hartree)    :          0.3673494485
      Inner Energy (Hartree)         :      -1138.5422006450
      Enthalpy (Hartree)             :      -1138.5412564359
      Electronic entropy             :          0.0000000000
      Rotational entropy             :          0.0165529331
      Vibrational entropy            :          0.0370722414
      Translational entropy          :          0.0165529331
      Entropy                        :          0.0746244078
      Gibbs Energy (Hartree)         :      -1138.6158808438
      Magnitude of dipole moment (Debye) :        5.2349730184
--------------------------------------------------------------------------
```

ent-IIIc

CARTESIAN COORDINATES

C	3.475137537230	-3.219157257909	-1.017251702641
C	4.082920239798	-2.553669058296	0.210591974823
C	3.337286842588	-1.319641935945	0.618272982170
C	2.455208429134	-0.688685014060	-0.160998945381
C	2.153964109164	-1.156203636476	-1.565293719714
H	4.110354695760	-3.243827957233	1.056893562813
H	2.548609775551	-3.728620409596	-0.734365753032
H	4.154814693722	-3.979204544228	-1.404910731097
H	4.096241771772	-1.652919547202	-2.345521180541
H	2.791374599551	-2.635730377558	-2.989814241125
H	3.554382947414	-0.944772746590	1.611660522921
H	1.147300180208	-1.590691316101	-1.589394098576
H	2.120506502257	-0.290868103013	-2.230196264039
H	5.126318443017	-2.281720658723	0.015125499442
C	1.737581526846	0.521584561452	0.327035722963
C	0.875557413360	1.193895480639	-0.470466186978
C	0.376817875242	2.043524380088	-1.327617124273
H	1.102843763359	2.688560996571	-1.819237799406
O	3.956627997374	2.230422712127	0.470943441156
C	-1.057398372071	2.224165345867	-1.712066501379
H	-1.188433756200	2.017431941167	-2.775247773868
H	-1.716568370534	1.569407988126	-1.143365763728
H	-1.359029607268	3.258727526912	-1.543290672867
C	3.402029960490	1.984056905386	1.510706069656
H	3.787518912091	2.380189311555	2.465682084149
C	2.141839291353	1.166336739456	1.637199997740
H	2.278038309506	0.443923706618	2.443551069274
H	1.370386645919	1.858908866553	1.983913645749
Au	-0.424935908518	-0.276294649816	0.648216891851

```
 P  -2.263872174763   -1.475635379346    1.368636815823
 C  -2.209975390163   -3.222564938329    0.914827318493
 H  -1.317138305426   -3.681371754285    1.337244678026
 H  -3.097137743480   -3.724403987997    1.302948363779
 H  -2.180972224770   -3.318205326695   -0.169623513163
 C  -3.809528931433   -0.844152456847    0.681447628610
 H  -4.643170576174   -1.445958265265    1.045095742764
 H  -3.947757035900    0.192701843193    0.984704897744
 H  -3.771387537243   -0.894108769238   -0.405906715967
 C  -2.482680463361   -1.453212957631    3.161079853827
 H  -1.608260193702   -1.891944569746    3.639677789197
 H  -2.598605840229   -0.426018028392    3.503487745250
 H  -3.370070443526   -2.029358880941    3.425785654601

  Total DFT Energy (No VdW correction)    -1138.9515023392
       Van der Waals Correction:       -0.0088128470
       Temperature (Kelvin)           :       298.1500000000
       Pressure (atm)                 :         1.0000000000
       Total Mass (AMU)               :       449.3045000000
       Spin Degeneracy                :         1.0000000000
       Electronic Energy (Hartree)    :     -1138.9603151862
       Translational Energy (Hartree) :         0.0014162714
       Rotational Energy (Hartree)    :         0.0014162714
       Vibrational Energy (Hartree)   :         0.0209864566
       Zero Point Energy (Hartree)    :         0.3685076450
       Inner Energy (Hartree)         :     -1138.5679885418
       Enthalpy (Hartree)             :     -1138.5670443327
       Electronic entropy             :         0.0000000000
       Rotational entropy             :         0.0163458869
       Vibrational entropy            :         0.0387743598
       Translational entropy          :         0.0163458869
       Entropy                        :         0.0761194800
       Gibbs Energy (Hartree)         :     -1138.6431638128
       Magnitude of dipole moment (Debye) :        9.0317101117
-------------------------------------------------------------------------
```
